# AS601245, an Anti-Inflammatory JNK Inhibitor, and Clofibrate Have a Synergistic Effect in Inducing Cell Responses and in Affecting the Gene Expression Profile in CaCo-2 Colon Cancer Cells

**DOI:** 10.1155/2012/269751

**Published:** 2012-02-29

**Authors:** Angelo Cerbone, Cristina Toaldo, Stefania Pizzimenti, Piergiorgio Pettazzoni, Chiara Dianzani, Rosalba Minelli, Eric Ciamporcero, Guglielmo Roma, Mario Umberto Dianzani, Roberto Canaparo, Carlo Ferretti, Giuseppina Barrera

**Affiliations:** ^1^MerckSerono Ivrea, Istituto di Ricerche Biomediche “A. Marxer”, RBM S.p.A., 10010 Colleretto Giacosa, Italy; ^2^Department of Medicine and Experimental Oncology, Section of General Pathology, University of Turin, 10125 Turin, Italy; ^3^Department of Anatomy, Pharmacology and Forensic Medicine, Section of Pharmacology and Pharmacognosy, University of Turin,10125 Turin, Italy; ^4^Department of Anatomy, Pharmacology, and Forensic Medicine, Section of Pharmacology and Experimental Therapy, University of Turin, 10125 Turin, Italy

## Abstract

PPAR*α*s are nuclear receptors highly expressed in colon cells. They can be activated by the fibrates (clofibrate, ciprofibrate etc.) used to treat hyperlipidemia. Since PPAR*α* transcriptional activity can be negatively regulated by JNK, the inhibition of JNK activity could increase the effectiveness of PPAR*α* ligands. We analysed the effects of AS601245 (a JNK inhibitor) and clofibrate alone or in association, on proliferation, apoptosis, differentiation and the gene expression profile of CaCo-2 human colon cancer cells. Proliferation was inhibited in a dose-dependent way by clofibrate and AS601245. Combined treatment synergistically reduced cell proliferation, cyclin D1 and PCNA expression and induced apoptosis and differentiation. Reduction of cell proliferation, accompanied by the modulation of p21 expression was observed in HepG2 cells, also. Gene expression analysis revealed that some genes were highly modulated by the combined treatment and 28 genes containing PPRE were up-regulated, while clofibrate alone was ineffective. Moreover, STAT3 signalling was strongly reduced by combined treatment. After combined treatment, the binding of PPAR*α* to PPRE increased and paralleled with the expression of the PPAR coactivator MED1. Results demonstrate that combined treatment increases the effectiveness of both compounds and suggest a positive interaction between PPAR*α* ligands and anti-inflammatory agents in humans.

## 1. Introduction

PPARs are ligand-activated transcription factors belonging to the nuclear receptor superfamily. Three molecular forms of PPAR have been identified, namely, PPAR*α*, PPAR*β*/*δ*, and PPAR*γ*, and all involved in many different biological processes [1]. PPAR*α* is the predominant PPAR subtype highly expressed in liver, heart, proximal tubules of kidney cortex, skeletal muscle, intestinal mucosa, and in brown adipose tissues that are metabolically very active [2].

Endogenous ligands with high specificity for PPAR*α* are long-chain unsaturated fatty acids and fatty acid derivatives [3, 4]. Fibrates, which are hypolipidemic drugs used in the treatment of hyperlipidemia, are among the group of synthetic ligands, which are the most important agonists of PPAR*α*.

Since PPAR*α* is expressed in the digestive tract and mainly localized in the intestinal mucosa in the small intestine and in the colon, it has been proposed that a physiological role of the receptor may be to sense the total flux of dietary fatty acids in key tissues [5]. Colon epithelial cells can be physiologically exposed not only to fatty acids but also to hypolipidemic drugs such as fibrates, all PPAR*α* agonists. For this reason, there is particular interest to study the effect of PPAR*α* ligands in colon cancer cells.

Less is known about the role of PPAR*α* in human tumors. Generally, activation of this PPAR by agonists causes inhibition of tumor cell growth [6, 7]. In contrast, in liver murine cell models, Wy-14,643, clofibrate, ciprofibrate, and DEHP were inducers of c-fos, c-jun, junB egr-1, and NUP475 [8]. Indeed, PPAR*α* has been widely employed in hepatocarcinogenesis protocols for rodents [9, 10]. However, in human cell models, PPAR ligands downregulate oncogenes and upregulate proapoptotic genes also [11, 12]. In particular, the proapoptotic role of PPAR*α* ligands has been outlined by a recent review [13].

 Beside ligand induction, PPAR*α* activity can be regulated by JNK and p38 mitogen-activated protein kinase (MAPK) phosphorylation. The p38 MAPK phosphorylates the A/B domain of PPAR*α* and enhances its ligand-dependent transcriptional activity [14]. On the contrary, the activation of ERK-MAPK decreases PPAR*α* activity [15]. By inhibiting Rho A, a component of Rho family proteins, which regulate the JNK and the p38 MAPK cascades, cerivastatin stimulates PPAR*α* transcriptional activity by reducing its phosphorylation [16].

AS601245[1,3-Benzothiazol-2-yl-(2-{[2-(3-pyridinyl)ethyl]amino}-4-pyrimidinyl) acetonitrile; JNK inhibitor V] has been selected as a potent and selective JNK inhibitor with anti-inflammatory properties [17]. In the present work, we intend to assess its effect on clofibrate action in colon cancer cells. In particular, we examined the effects of AS601245 and clofibrate, alone or in association, on apoptosis, differentiation, and PPRE binding activity of PPAR*α* in CaCo-2 human colon cancer cells and analysed, through microarray analysis (Affymetrix GeneChip), the gene expression pattern in control and drug-treated cells. Moreover, since the liver is the major target organ expressing PPAR*α*, to assess whether combined treatment could produce some toxic effects in liver cells, we tested, through MTT analysis, the acute toxicity of the both substances and the effect of single and combined treatments on cell proliferation and proliferation-related gene expressions in HepG2 human hepatic cells.

## 2. Materials and Methods

### 2.1. Cell Culture and Treatments

CaCo-2 and HepG2 cells, obtained from European Collection of Cell Cultures (ECACC), were cultured at 37°C in a humidified atmosphere of 5% CO_2_-air. For all experiments, cells, from 1 to 10 passages, were used. Cells were grown in D-MEM medium supplemented with 10% fetal bovine serum (HyClone, Italy), 2 mM glutamine, 1% nonessential amino acids solution and 1% antibiotic mixture (penicillin streptomycin) (Sigma, Milano, Italy).

Treatments with clofibrate and AS601245[1,3-benzothiazol-2-yl-(2-{[2-(3-pyridinyl)ethyl]amino}-4-pyrimidinyl) acetonitrile; JNK inhibitor V] (SPRI, Geneva, Switzerland) were performed by suspending the drugs in DMSO. The concentration of vehicle in culture did not exceed 1%. Moreover, cultures, treated with 1% DMSO alone, were performed to exclude the vehicle's effects.

### 2.2. Cell Proliferation and Viability

CaCo-2 cell proliferation was evaluated by using the kit “CellTiter-Glo Luminescent Cell Viability Assay” (Promega, Milano, Italy). This highly sensitive assay detects the luminescence released by the metabolically active cells. Quantification of luminescence was expressed as RLU (relative light unit). For the proliferation experiments, treatments were performed by adding the drugs (at different concentrations) to the CaCo-2 cells seeded at about 4,000 cells/well in a 96-well plate. HepG2 cell proliferation was analysed through the MTT method. Briefly, 1500 cells/well were seeded in 200 *μ*L of serum-supplemented media and the following day treated with the drugs. 20 *μ*L of 5 mg/mL thiazolyl blue tetrazolium bromide (M2128-SIGMA Aldrich) was subsequently added to the cells and removed 2 hours later. 100 *μ*L of DMSO was added to the cells, and the absorbance was recorded at 570 nm through a 96 well plate ELISA reader. Viability was evaluated through Trypan blue (T8154 SIGMA Aldrich) exclusion test.

### 2.3. Detection of Apoptosis

Apoptosis was evaluated, 24 and 48 hours after the treatments, by using a Kit Caspase-GLO 3/7 Assay (Promega, Milano, Italy) and confirmed by TUNEL test. The Caspase-GLO 3/7 assay determines the amount of caspase 3 and 7 cleavage of the luminogenic substrate resulting in luciferase reaction and production of light. The TUNEL method from Promega (Milano, Italy) consists of the terminal deoxynucleotidyl transferase-mediated nick-end labelling of FITC-conjugated deoxyuridine triphosphate. Incorporated fluorescein was detected using a fluorescence microscope (Leitz, Dialux 20, Oberkochen, Germany). The number of apoptotic cells was determined by counting the percentage of green fluorescence-positive cells. At least, 100 cells were counted for each experiment. CaCo-2 cells treated with 10 mM butyrate were used as positive control of apoptosis. 

### 2.4. Detection of Differentiated Cells

Differentiation was determined by counting the dome formation in the control or treated cells. Dome formation, which can be attributed to ion and water transport across polarized epithelial cells [18], was quantified in confluent CaCo-2 monolayers using inverted light microscopy and expressed as the number of domes per square centimetre. Domes were recognised as a cohesive group of approximately 30 cells or more that were in a different plane of view compared to cells attached to the culture plate. 

Preliminary experiments demonstrated that results obtained by dome count were similar to those obtained with two other methods (transepithelial electrical resistance (TEER) determination and phosphates alkaline assay) (data not shown). CaCo-2 cells treated with 2 mM sodium butyrate (Sigma, Milano, Italy) were used as a positive control.

### 2.5. RNA Extraction and Array Hybridization

Total RNA from 3 biological replicates of each treatment was isolated using TRIzol reagent (Invitrogen, Milano, Italy). Samples were treated with DNase in order to avoid any genomic contamination. The quality of the resulting RNA was determined by using the Agilent 2100 Bioanalyzer (Agilent Technologies), and RNA content was normalized by using the Thermo Scientific NanoDrop ND-1000 spectrophotometer. RNA samples of each replicate were analyzed by using Affymetrix GeneChip Human Genome U133A plus 2.0 chips (Affymetrix).

GCOS software version 1.2 (Affymetrix) was used to define the probe cell and to calculate the intensity for each cell. CEL files generated were analyzed for the overall data quality using R/Bioconductor. Then, they were processed into the Rosetta Resolver dataset for data normalization, generation of expression values, and statistical analysis. Expression values of all treatment groups were obtained as a ratio versus the negative control (1% DMSO).

Differential analyses between pairs of groups were performed with 1-way ANOVA followed by the Benjamini-Hochberg multiple testing correction (False Discovery Rate-FDR cut-off of 1%) and a Student-Newman-Keuls post hoc analysis. Finally, differentially expressed genes were analysed in Ingenuity Pathway Analysis software version 7.5 (Ingenuity Systems, http://www.ingenuity.com/).

### 2.6. Real-Time RT-PCR

We selected 4 genes that had shown an approximately 2-fold change in expression for further study by RT-PCR in a separate experiment. Two of these (GANAB and IL6ST) were found to be increased in Affymetrix analysis, whereas the other two were found to be decreased (FGFR2 and CCNG). Total RNA was reverse transcribed into cDNA in a 20 *μ*L reaction using the TaqMan High Capacity cDNA Reverse Transcription Kit provided by Applied Biosystems. 500 ng of total RNA was used as the starting material from each sample. For each Real-Time PCR reaction, the reverse transcribed sample was used as a template. Target mRNA was quantified using an ABI 7900 HT Fast real-time PCR system (Applied Biosystems). Primers were purchased from Applied Biosystems: GANAB (glucosidase, alpha; neutral AB, Hs00929274_m1), IL6ST (interleukin 6 signal transducer (gp130, oncostatin M receptor, Hs00174360_m1), CCNG (cyclin G2, Hs00171119_m1), and FGFR2 (fibroblast growth factor receptor 2, Hs01552926_m1). The TaqMan probes were labelled with a 5′ reporter dye (FAM, 6-carboxyfluorescein) and a 3′ quencher dye (TAMRA, 6-carboxytetramethylrhodamine). Real-time PCR reactions were carried out in triplicate. Data were analyzed by the ABI Sequence Detection System (SDS) software using the relative quantification. The fold changes are determined by the ΔΔCt method as described in Applied Biosystems User Bulletin no. 2.

### 2.7. DNA-Binding Activity of PPAR*α*

The PPAR*α* binding activity assay was performed by using Trans-AM ELISA-based kit from Active Motif (Carlsbad, CA, USA) according to the manufacturer's protocol. Briefly, cell extracts were incubated in a 96-well plate coated with an oligonucleotide containing the PPRE motif (5′-AACTAGGTCAAAGGTCA-3′). PPAR contained in nuclear extract, specifically bound to the immobilized oligonucleotide, was detected by using an antibody anti-PPAR*α* (clone H-98, from Santa Cruz Biotechnology) followed by a secondary HRP- (horseradish-peroxidase-) conjugated antibody (Bio-Rad Laboratories) in an ELISA-like assay.

### 2.8. Western Blot Analysis

Total extracts were prepared by lysis in a buffer containing Tris-HCl buffer, pH 7.4, 150 mM NaCl, 5 mM EDTA, 1% Nonidet P-40, 1 mM sodium orthovanadate, 1 mM phenylmethylsulfonyl fluoride, and 0.05% aprotinin. Insoluble proteins were discarded by high-speed centrifugation at 4°C. Protein concentration in the supernatant was measured in triplicate using a commercially available assay (Bio-Rad Laboratories, Segrate, Italy).

All proteins were separated by SDS-polyacrylamide gel and electroblotted on nitrocellulose membrane (Bio-Rad Laboratories, Segrate, Italy). Membranes were blocked overnight at 4°C in Tris buffered saline (TBS) containing 5% milk plus 0.5% Tween 20 and then incubated at room temperature with primary antibodies (anti-P-PPAR alpha (Ser21) from Thermo Scientific; anti-PPAR alpha from Millipore; anti-STAT3 clone F-2, anti-p-STAT3 (Tyr705) clone B-7, anti-PCNA clone FL-261, anticyclin D1 clone A12 from Santa Cruz Biotechnology; anti-p-STAT3 (Ser727) from ThermoScientific; anti-PPARBP (MED1) clone 2A2; anti-*β*-actin clone AC-1 from Sigma-Aldrich; anti-P-Jun from cell signaling; anti-p21 from Abcam and horseradish-peroxidase-conjugated secondary antibodies (Bio-Rad Laboratories, Segrate, Italy). Detection was carried out by enhanced chemiluminescence (ECL) according to the manufacturer's protocol (Amersham-Pharmacia Biotech, Italy, Cologno Monzese, Italy).

Densitometric analysis was performed by using a software program (Multi-Analyst, version 1.1, Bio-Rad Laboratories, Segrate, Italy). All results were standardized using the signal obtained with *β*-actin.

### 2.9. Statistical Analysis

The 2-way ANOVA was performed in the proliferation, apoptosis, differentiation, and PPRE binding assays.

## 3. Results

### 3.1. Growth of Clofibrate and AS601245-Treated Cells

**Figure 1:**
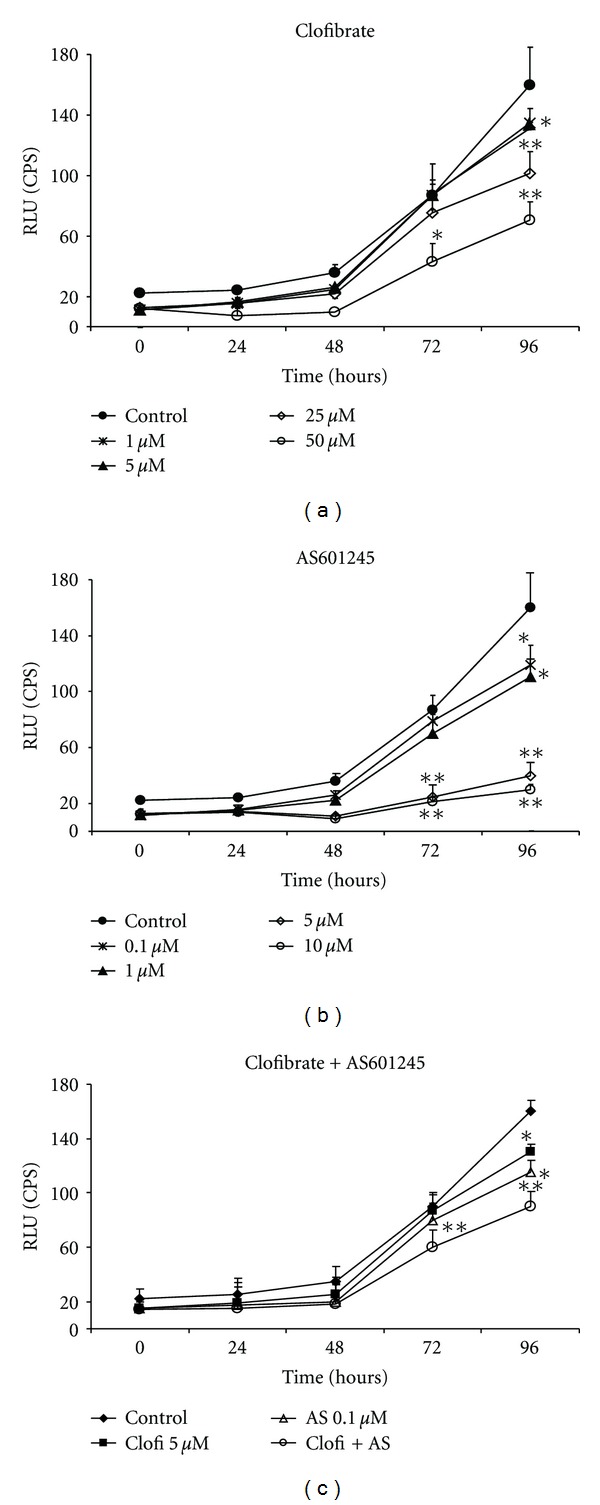
*Caco-2 cell proliferation.* (a) Growth of Caco-2 cells treated with clofibrate at the indicated concentrations (from 1 to 50 *μ*M) and at different times; (b) growth of Caco-2 cells treated with AS601245 at the indicated concentrations (from 0,1 to 10 *μ*M) and at different times; (c) growth of Caco-2 cells treated with 5 *μ*M clofibrate, 0,1 *μ*M AS601245, and the association of these two compounds (Clofi + AS601245). Cell proliferation was detected by measuring the luminescence released by the metabolically active cells. The values expressed in RLU (relative light units) are the means ± SD of three separate experiments. Variance analysis: *P < 0.05, **P < 0.01 versus control.

Both clofibrate and AS601245 were able to inhibit CaCo-2 cell proliferation in a dose-dependent way (Figures 1(a) and 1(b)). To evaluate the efficacy on cell growth of the combination of clofibrate and AS601245, we chose doses able to reduce cell proliferation by 20% (IC20) after 48 hours of treatment. These doses were 5 *μ*M clofibrate (IC 20 : 5 ± 0.4) and 0.1 *μ*M AS601245 (IC 20 : 0.1 ± 0.01). Combined treatment significantly reduced cell growth after 72 and 96 hours from the treatment (Figure 1(c)). The treatment with two compounds synergistically reduced the cell growth after 72 hours, whereas, after 96 hours, the reduction is less pronounced. The doses of 5 *μ*M clofibrate, 0.1 *μ*M AS601245, and the combined treatment with these doses were used for all the subsequent experiments.

### 3.2. Proliferation-Related Genes Expressions

**Figure 2:**
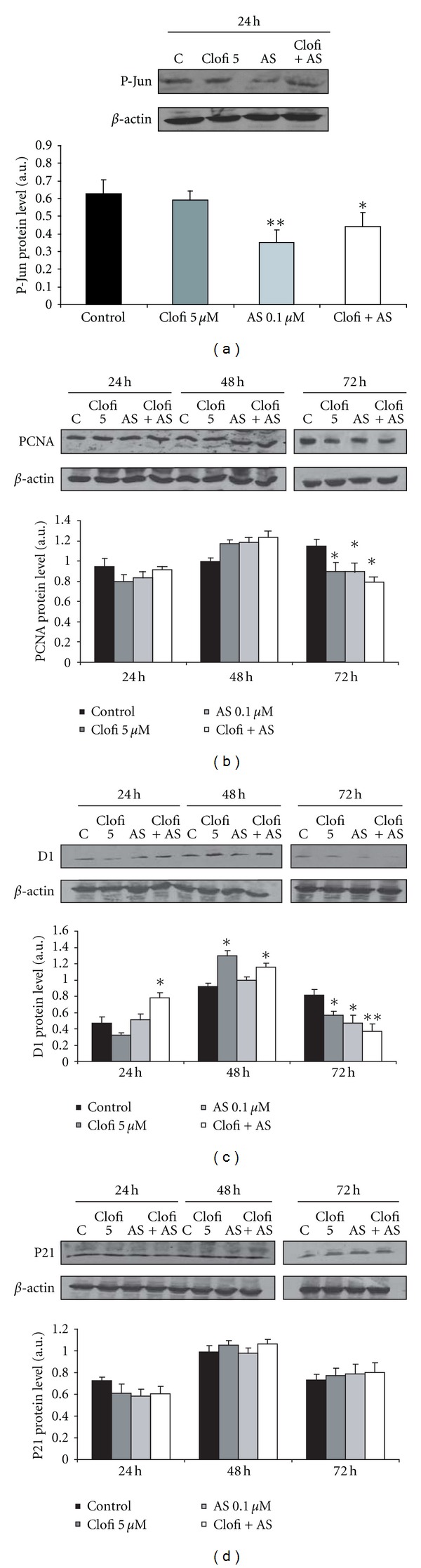
*Proliferation-related protein expressions. *(a) Determination of 0.1 *μ*M AS601245 effect in reducing P-Jun expression; (b) time course of PCNA expression in CaCo-2 cells treated with 5 *μ*M clofibrate, 0.1 *μ*M AS601245, and the association of these two compounds (Clofi + AS601245); (c) time course of cyclin D1 expression in CaCo-2 cells treated with 5 *μ*M clofibrate, 0.1 *μ*M AS601245, and the association of these two compounds (Clofi + AS601245); (d) time course of p21 expression in CaCo-2 cells treated with 5 *μ*M clofibrate, 0.1 *μ*M AS601245, and the association of these two compounds (Clofi + AS601245). Graphics represent the relative quantification of protein products performed by densitometric scanning. Data were normalized by using the *β*-actin signal, expressed as arbitrary densitometric units, and are the mean ± SD of three separate experiments from three different preparations for each condition. Variance analysis: *P < 0.05, **P < 0.01 versus control.

Since the reduction of proliferation can be accompanied by the modulation of specific genes, we determined the expression of proliferating cell nuclear antigen (PCNA), cyclin D1 and p21, in CaCo-2 cells. Moreover, we found that AS601245 at the concentration of 0.1 *μ*M was able to inhibit Jun phosphorylation in CaCo-2 cells. In Figure 2(a), the analysis of P-Jun expression revealed that the amount of P-Jun protein was reduced in cells treated with 0.1 *μ*M AS601245. A similar result was observed in cells treated with AS601245 plus clofibrate.

The PCNA expression was reduced in CaCo-2 cells only after 72 hours from the treatments with clofibrate, AS601245, and their combination (Figure 2(b)). Cyclin D1 expression was induced by clofibrate 48 hours from the treatment, but, in the following hours, its expression decreased particularly in combined treatment (Figure 2(c)). Finally, p21 expression did not vary significantly after the treatments (Figure 2(d)).

### 3.3. Apoptosis and Differentiation after AS601245 and Clofibrate Treatments

**Figure 3:**
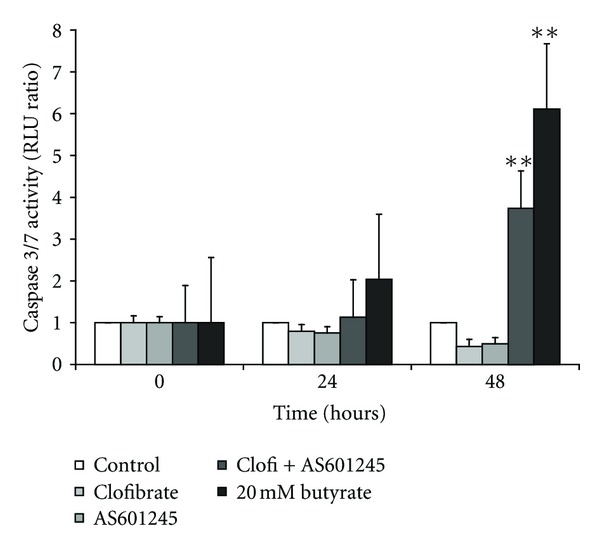
*Induction of apoptosis.* Induction of apoptosis in CaCo-2 cells treated with 5 *μ*M clofibrate, 0.1 *μ*M AS601245, and the association of these two compounds (Clofi + AS601245). Apoptosis was evaluated at 0, 24, and 48 hours after the treatments, by measuring caspase 3/7 activity. 20 mM butyrate was used as a positive control. Results normalized to the respective control value for each experimental time is the mean ± SD of three separate experiments from three different preparations for each condition. Variance analysis: **P < 0.01 versus control.

Apoptosis detection is reported in Figure 3. Both clofibrate and AS601245 were unable to induce apoptosis when added separately to CaCo-2 cells. Conversely, the association of these two compounds induced a significant increase in caspase 3/7 activity, at 24 and 48 hours after the combined treatments. After 48 hours from the combined treatment, the caspase 3/7 activity was about 4-fold higher than that detected in the control cells. These results were confirmed by using TUNEL test (data not shown).

**Figure 4:**
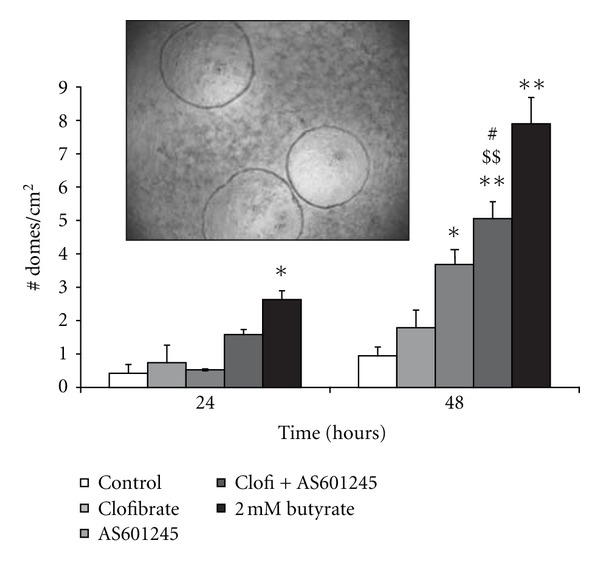
*Induction of differentiation.* Differentiation of CaCo-2 cells treated with 5 *μ*M clofibrate, 0.1 *μ*M AS601245, and the association of these two compounds (Clofi + AS601245). Cell differentiation was evaluated at 24 and 48 hours after the start of treatments, by measuring dome formation. 2 mM butyrate was used as a positive control. Results, expressed as number of domes per cm^2^, are the mean ± SD of three separate experiments from three different preparations for each condition. Variance analysis: *P < 0.05, **P < 0.01 versus control; ^$$^ *P* < 0.01, versus clofibrate-treated cells; ^#^ *P* < 0.05 versus AS601245-treated cells. The picture represents a microscopic image of domes.

Differentiation of CaCo-2 cells was induced by 0.1 *μ*M AS601245 at 48 hours after the treatment, whereas clofibrate alone did not increase the Dome number (Figure 4). Combined treatment increased the Dome number after 24 hours and caused a greater induction of differentiation compared to single treatments, after 48 hours (an increase of about 5-fold with respect to the control value).

### 3.4. Microarray Analysis of Gene Expression in CaCo-2 Cells

To analyze whether the cell responses to the treatments with 5 *μ*M clofibrate, 0.1 *μ*M AS601245, or with both substances were a consequence of a specific gene pathway modulation, we performed microarray analysis by using the Affymetrix GeneChip platform, 24 hours after the treatments. The complete list of the genes modulated by clofibrate, AS601245, and by the combined treatment is reported in the supplementary data (See Table A in Supplementary Material available online at doi:10.1155/2012/269751). To confirm results obtained by microarray analysis, real time-PCR of 4 selected genes (2 upregulated and 2 downregulated) was performed. Results, reported in supplementary data (Table B), indicated that the up or down fold changes obtained by microarray analysis were similar to those obtained in real-time PCR. Some discrepancies were found only for FGFR2 and CCNG that did not change in clofibrate treated cells, if evaluated by microarray analysis, whereas they were decreased by about −1.5-fold, if analysed by real-time PCR. The supplementary data (Table C) also indicates the genes affected by clofibrate, by AS601245, and by the combined treatment with clofibrate and AS601245, arranged with respect to the relative biological functions and listed on the basis of the *P* value. The genes mainly affected by clofibrate, which modulated a limited group of genes, belonged to the “cancer,” “cellular development,” and “gene expression” groups. AS601245 modulated genes belonging to “cancer,” “cell cycle,” “genetic disorders,” and “cell death” groups. The combined treatments affected mainly genes belonging to “cancer” and “genetic disorder” functions.

**Figure 5:**
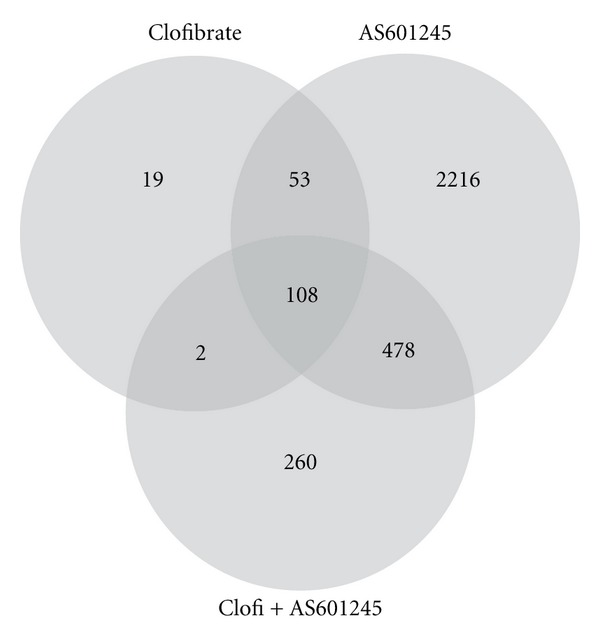
*Microarray analysis of gene expression.* Venn diagram derived from microarray analysis of gene expression in CaCo-2 cells treated with 5 *μ*M clofibrate, 0.1 *μ*M AS601245, and the association of these two compounds (Clofi + AS601245) at 24 hours. The diagram shows the number of genes modulated by the treatments.

The Venn diagram (Figure 5) shows that clofibrate modulated 182 genes, AS601245 modulated 2855 genes, and the combined treatments modulated 848 genes. Among the genes affected by clofibrate alone or AS601245 alone, 161 were common in both of the two groups. The combined treatment affected 108 genes which were present in both the clofibrate and AS601245 groups and 260 genes which were not affected by either clofibrate or AS601245 alone. Among these 260 genes, 65 only were downregulated by the combined treatment, whereas 195 genes were upregulated.

The top ten genes changed the most by the clofibrate treatment, with respect to 1% DMSO treated cells, are reported in Table 1. Clofibrate treatment (Table 1(a)) mainly increased the expression of the GANAB gene (3.8-fold change) which encodes glucosidase alpha, the ATF6B gene which encoded a transcription factor belonging the unfolded protein response (UPR) pathway during ER stress [19, 20], and some genes, belonging to “cancer” function (DLST, IL6ST, MEX3d). Among genes downregulated by clofibrate, the major part belonged mainly to functional groups: “cancer” (STIP1, HNRNPA1, VIL1, and CDH1) and “cellular assembly and organization” (CLASP1 and MACF1).

The genes modulated by the treatments with AS601245 are indicated in Table 1(b). In this case, the gene most upregulated was CYP1A1 (5.3 fold change). The other genes upregulated belonged mainly to the “cancer” and cell death functions (NFAT5, BMO2K, DLST, IL6ST, FAM76B, and MGA). The downregulated genes belonged mainly to the “cancer” biofunction (RPS27A, HNRNPA1, STIP1, and TFDP1).

The top ten genes affected the most by the combined treatment with clofibrate and AS601245 are reported in Table 1(c). Among the genes upregulated by the combined treatment, 6 were upregulated by treatments with the single substances also. CYP1A1 was increased by 7.5-fold, whereas AS601245 alone induced its expression by 5.3-fold. IL6ST was induced by clofibrate (2.0 fold), by AS601245 (2.5-fold change), and, more intensively, by the combined treatment (6.5-fold change). AP3D1 was increased (3.6-fold change) in AS601245 treated cells and with an even greater increase (5.0-fold change) in cells treated with both compounds.

NFAT5 and GANAB genes remained almost unchanged in combined treatment. Finally, SMRCC1 gene expression was increased in AS601245-treated cells by a 2.0-fold change (Table B) and by a 3.1-fold change in cells treated with both substances. The resting genes (WASF2, VAPB, THRA, and BDP1) were not affected in cells treated with a single compound, whereas they were highly increased in cells treated with both substances. Among the downregulated genes, four genes which were unaffected by single treatments (TRUB1, NXT2, FGB, and ACTC1) reached the top ten positions in the combined treatment.

Other genes downregulated with the combined treatment were downregulated also, to a lesser extent, in clofibrate- and AS601245-treated cells (MALAT1, LARP5, and TFRC) or in only AS601245-treated cells (SLC39A14). Finally, two genes were downregulated by a single treatment with AS601245 more than by the combined treatment (HNRNPA1 and RPS27A).

We postulated that the inhibition of JNK could increase the affinity of activated PPAR*α* for the PPRE sequences. For this reason, we investigated, among the genes activated by clofibrate, by AS601245, and by the combined treatment, the genes having PPRE sequences by using the genomewide library of high-confidence predicted PPAR target genes as published by Lemay and collaborators [21] (Table 2). It is noteworthy that, among the genes upregulated by the clofibrate 24 hours after the treatment, none had the PPRE putative sequence in the promoter, indicating that the clofibrate, at this concentration, was not able to induce PPAR*α* binding to PPRE containing genes. After treatment with AS601245 and clofibrate, the number of activated genes, containing PPRE sequences, greatly increased (28 genes were upregulated).

#### 3.4.1. Effect of Clofibrate and AS601245 Treatments on PPAR*α* Binding to PPRE and Med1 Expression

**Figure 6:**
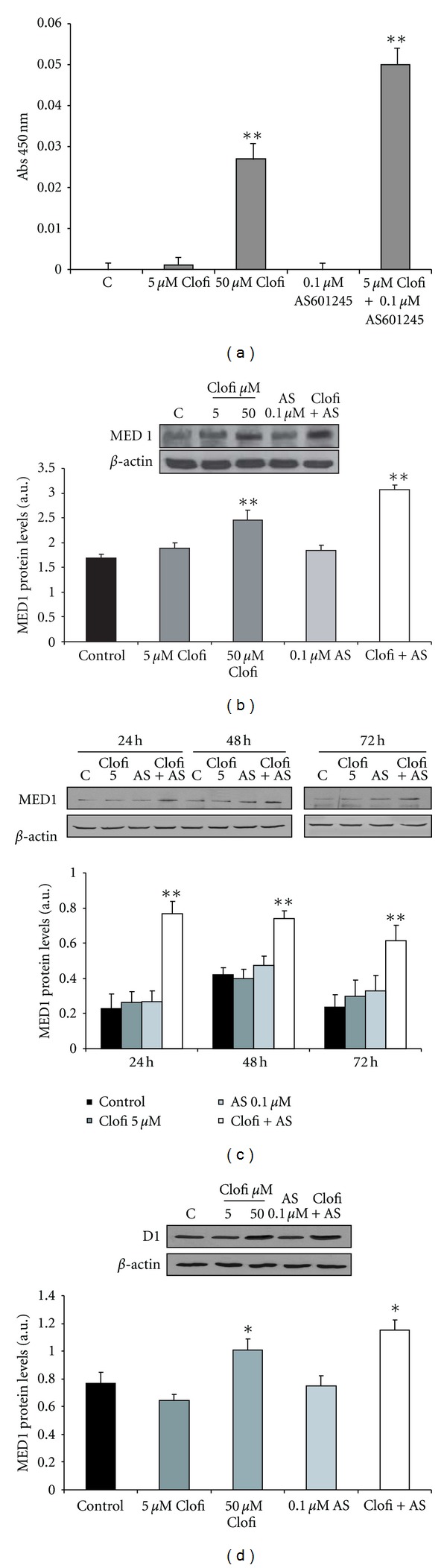
*PPRE binding activity and Western Blot analysis of Med1 and cyclin D1.* (a) PPRE binding activity of PPAR*α*. Results represent the absorbance, detected at 450 nm, subtracted from the control value, and are the mean ± SD of three separate experiments from three different preparations for each condition; (b) Western Blot analysis of Med1 protein detected in CaCo-2 cells treated with 5 *μ*M and 50 *μ*M clofibrate (Clofi), 0.1 *μ*M AS1245, and 5 *μ*M clofibrate plus 0.1 *μ*M AS601245 (Clofi + AS601245), collected at 24 hours after the treatment; (c) Western Blot analysis of Med1 protein detected in CaCo-2 cells treated with 5 *μ*M clofibrate, 0.1 *μ*M AS1245, and 5 *μ*M clofibrate plus 0.1 *μ*M AS601245 (Clofi + AS), collected at 24, 48, and 72 hours after the treatment; (d) Western Blot analysis of cyclin D1 detected in CaCo-2 cells treated with 5 *μ*M and 50 *μ*M clofibrate (Clofi), 0.1 *μ*M AS1245, and 5 *μ*M Clofibrate plus 0.1 *μ*M AS601245 (Clofi + AS601245), collected at 24 hours after the treatment. Graphics represent the relative quantification of protein products performed by densitometric scanning. Data were normalized by using the *β*-actin signal, expressed as arbitrary densitometric units, and are the mean ± SD of three separate experiments from three different preparations for each condition. Variance analysis: *P < 0.05, **P < 0.01 versus control.

Since microarray analysis indicated that clofibrate alone did not induce expression of genes containing PPRE sequences, whereas the treatment with AS601245 and clofibrate does, we examined, through Elisa assay, the binding of nuclear protein extracts from control cells and cells treated with clofibrate alone, AS601245 alone, and combined treatment with clofibrate and AS601245, to PPRE sequences.  Figure 6(a), indicates that 5 *μ*M clofibrate and 0.1 *μ*M AS601245 alone did not increase the PPRE binding, whereas the PPRE binding activity was increased in cells treated with 50 *μ*M clofibrate and, more intensively, in cells treated with 5 *μ*M clofibrate and 0.1 *μ*M AS601245. This increase in binding activity is not linked to the variation of PPAR*α* phosphorylation in serine 21, as demonstrated by the western blot analysis with an anti-phospho-PPAR alpha antibody (data not shown). However, microarray analysis revealed that the PPAR alpha activator, MED1, was increased in combined treated cells, only. To verify if this increase corresponded to an increase in MED1 protein, we analyzed, by Western Blot, the expression of MED1. Results confirmed that MED1 protein was doubled in cells treated with both compounds (Figure 6(b)); moreover, its concentration was increased in cells treated with 50 *μ*M clofibrate also. To assess whether the increase of MED1 expression was maintained in the following days in cells exposed to combined treatment, we performed a time-course analysis of MED1 expression. The results confirmed that the increase of MED1 expression was evident until 72 hours after combined treatment (Figure 6(c)). Among the genes, bearing PPRE sequences in their promoter and activated 24 hours after combined treatment, cyclin D1 has been found. We demonstrated that cyclin D1 expression was increased 24 hours after treatment with 50 *μ*M clofibrate and 5 *μ*M clofibrate plus 0.1 *μ*M AS601245. These variations of cyclin D1 expression at 24 hours were similar to those observed for MED1 expression. (Figure 6(d)). 

### 3.5. Analysis of JAK/STAT Signalling

The most important gene, modulated by combined treatment, was IL6ST (also called gp130).

**Figure 7:**
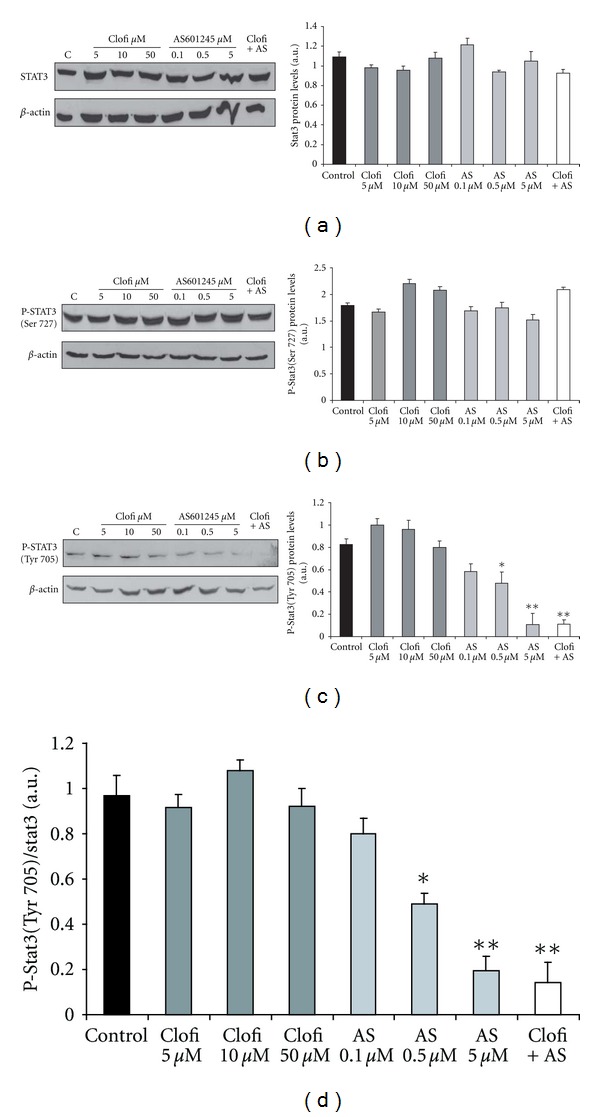
*Analysis of JAK/STAT signalling.* Western Blot analysis of STAT3 (a), P-STAT3 (Ser727) (b), and P-STAT3 (Tyr705) (c) proteins levels analyzed in CaCo-2 cells treated with clofibrate (Clofi) alone, AS601245 alone at the indicated concentrations, and combined treatment with 5 *μ*M clofibrate and 0,1 *μ*M AS601245 (Clofi + AS) and collected at 24 hours after the treatment. Graphics represent the relative quantification of protein products performed by densitometric scanning. Data were normalized by using the *β*-actin signal and expressed as arbitrary densitometric units. (d) Graphics represent the normalization of the protein level of P-STAT3 (Tyr705) by using STAT3 signal. Results are the mean ± SD of three separate experiments from three different preparations for each condition. Variance analysis: *P < 0.05, **P < 0.01 versus control.

This protein is the receptor for IL-6 cytokine. The binding induces the dimerization of gp130 chains resulting in activation of Janus kinases (JAKs). JAKs phosphorylate gp130, leading to the activation and phosphorylation of the STAT1 and STAT3 transcription factors [22, 23]. Although both transcription factors can be involved in gp130 downstream signalling, it has been demonstrated that STAT3 is necessary for the growth of colorectal cancer in mice [24, 25]. For these reasons, we analysed in colon cancer cells the expression of STAT3 protein and the phosphorylation of STAT3 after clofibrate and AS601245 treatments (Figures 7(a), 7(b), 7(c) and 7(d)). STAT 3 expression did not vary after the treatments, and the amount of serine-phosphorylated STAT3 did not vary either. Interestingly, the amount of tyrosine-phosphorylated STAT3 is strongly decreased after treatments with the highest concentration of AS601245 and in particular after combined treatment, which induced a synergistic decrease of phosphorylation.

### 3.6. Effects of AS601245 and Clofibrate in HepG2 Cells

**Figure 8:**
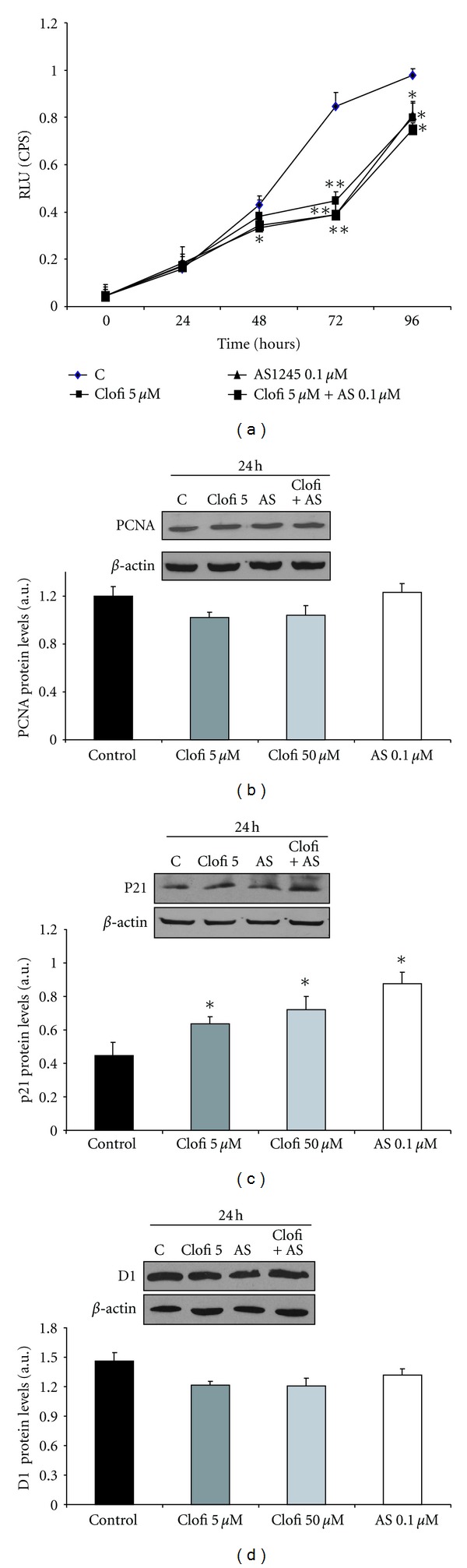
*Effect of clofibrate and AS601245 in HepG-2 human liver cells. *(a) Growth of HepG-2 cells treated with 5 *μ*M clofibrate, 0,1 *μ*M AS601245, and the association of these two compounds (Clofi + AS601245); (b) Western Blot analysis of PCNA detected in CaCo-2 cells treated with 5 *μ*M clofibrate, 0.1 *μ*M AS1245, and 5 *μ*M clofibrate plus 0.1 *μ*M AS601245 (Clofi + AS601245), collected at 24 hours after the treatment; (c) Western Blot analysis of p21, detected in CaCo-2 cells treated with 5 *μ*M clofibrate, 0.1 *μ*M AS1245, and 5 *μ*M clofibrate plus 0.1 *μ*M AS601245 (Clofi+AS601245), collected at 24 hours after the treatment; (d) Western Blot analysis of cyclin D1 detected in CaCo-2 cells treated with 5 *μ*M clofibrate, 0.1 *μ*M AS1245, and 5 *μ*M clofibrate plus 0.1 *μ*M AS601245 (Clofi + AS601245), collected at 24 hours after the treatment. Graphics represent the relative quantification of protein products performed by densitometric scanning. Data were normalized by using the *β*-actin signal, expressed as arbitrary densitometric units, and are the mean ± SD of three separate experiments from three different preparations for each condition. Variance analysis: *P < 0.05, **P < 0.01 versus control.

Since fibrates and other drugs are principally metabolized in liver, we tested the toxic effect of 5 *μ*M clofibrate, 0.1 *μ*M AS601245, and the association of the two substances in a liver cell line: the HepG2 cells, which has been demonstrated to be responsive to hepatotoxic compounds [26]. Acute toxicity was evaluated with the MTT test, performed 2 hours after the chemical additions. The amount of viable cells was determined by using the trypan blue dye exclusion test also. Results confirmed that 5 *μ*M clofibrate, 0.1 *μ*M AS601245, and the combined treatment with the two substances did not cause acute toxicity in HepG2 cells (data not shown). In this cell line, the effect of clofibrate, AS601245, and combined treatment in inhibiting cell proliferation was been evaluated (Figure 8(a)). All treatments reduced cell proliferation starting from 48 hours after the beginning of experiments, and the inhibitory effect reached the maximum at 72 hours. No significant differences were found between the three different treatments. Since the reduction of proliferation can be dependent on the modulation of specific gene expression, we tested PCNA, cyclin D1, and p21 proliferation-related protein expression, 24 hours after the treatments. PCNA and cyclin D1 expression did not change after the treatments (Figures 8(b) and 8(d)). On the contrary, p21 expression, which did not change after the treatments in CaCo-2 cells, was upregulated by clofibrate, AS601245, and combined treatment in HepG2 cells (Figure 8(c)).

## 4. Discussion

Results obtained demonstrated that the combined treatment with clofibrate and AS601245 inhibits proliferation and downregulated the expression of two proliferation-related proteins, PCNA and cyclin D1, 72 hours after the treatment of CaCO-2 human colon cells. Caspase activities and TUNEL test demonstrated that apoptosis increased significantly only in cells treated with both compounds, whereas apoptosis was not induced by such compounds, added separately. The proapoptotic action of PPAR ligands has been reported in many in vitro studies performed in some cancer cell lines [27, 28]. However, at the concentration used in this study, clofibrate alone did not induce a significant increase of caspase activity in CaCo-2 cells. AS601245 was ineffective too, although it modulated the expression of about 511 genes belonging to the “cell death” group. On the other hand, the JNK inhibition can elicit cellular responses, which range from apoptosis induction to increased survival, depending on the stimuli and the cell type [29]. Since the combined treatment only was able to induce apoptosis, we paid particular attention to genes, involved in the apoptotic pathway, which were unaffected or slightly modulated after the treatment with a single compound and highly modulated by the combined treatment. Among the top ten genes highly modulated by the combined treatment, the THRA gene, which codifies for the thyroid hormone receptor, has been shown to be related to the apoptosis induction of hematopoietic progenitor cells [30]. Several other genes, up- or downregulated by the combined treatment only, were involved in apoptosis induction. For example, CASP2 (caspase 2, +2.8-fold change) and KLF6 (Kruppel like factor 6, +1.5 fold change) [31, 32] were upregulated, whereas ARHGEF7 (Rho guanine nucleotide exchange factor-GEF7) [33] and RAB23 (member Ras oncogene family) were down regulated by a 1.9- and 2.2-fold change, respectively (Table A).

However, the most important change in gene expression, after combined treatment regards the IL6ST gene, which was induced about 6 fold, 24 hours after the treatment, as demonstrated by affymetric analysis and confirmed by RT-PCR. IL6ST (or gp130) is a universal signal transducing receptor for all IL-6 family cytokines [34]. The role played by IL-6 signalling in mediating tumour growth is equivocal [35], in certain tumour cells, it inhibits proliferation, whereas, in “in vivo” models, it stimulates cell growth. Moreover, the significance of an increase of gp130 expression also is controversial: on one hand, it may indicate an increase in the response to the IL-6 family cytokine stimuli, on the other hand, the soluble form signalling receptor subunit gp130, which is generated by differential splicing, is the natural inhibitor of IL-6 trans-signalling responses [35]. We analyzed the downstream signalling of gp130, through the analysis of STAT3 phosphorylation. STAT3 is constitutively active in most tumor cells but not in normal cells. Phosphorylation of STAT3 at tyrosine 705 leads to its dimerization, nuclear translocation, DNA binding, and gene transcription. The phosphorylation of STAT3 at serine 727 may regulate its activity negatively or positively [36]. AS601245 and, at higher level, the combined treatment reduced STAT3 phosphorylation at tyrosine 705, indicating a strong inhibition of STA3-induced gene transcription. STAT3 regulates the expression of genes that mediate survival and proliferation and can be involved in apoptosis induction and in reduction of cell proliferation caused by combined treatment. Interestingly, AS601245 alone reduced in a dose-dependent way STAT3 phosphorylation. This result is in agreement with the work by Kim et al. [37] demonstrating that a JNK inhibitor negatively correlated with the viability of cancer cells and reduced STAT3 activity.

As far as it regards differentiation induction, clofibrate alone did not increase differentiation of CaCo-2 cells. AS601245 is more effective than clofibrate in inducing differentiation, and it shows a synergistic effect when added together with clofibrate. As well as for the apoptosis induction, the action of JNK in differentiation is controversial. Ding and collaborators demonstrated that differentiation is associated with increased JNK activity and c-Jun phosphorylation [38]. In agreement with these findings, inhibition of JNK has been shown to attenuate intestinal cell differentiation [39]. On the contrary, other authors demonstrated that the MAP kinase pathway inhibitor U0126, in combination with butyrate, promotes differentiation in some colon cancer cell lines [40]. Our results demonstrated that AS601245, at 24 hours, did not increase the number of Domes, whereas, after 48 hours, differentiation induction was evident. Moreover, the addition of AS601245 to clofibrate increased the differentiation of CaCo-2 cells both at 24 and 48 hours.

By analysing the expression profile of the genes involved in this pathway, we found some genes affected by AS601245, which were further modulated by the association of these two compounds (Table A). A role, in the differentiation-process, may be played by the MGA gene, activated by 2.361-fold in AS601245-treated cells and by 3.089-fold in cells treated with AS601245 and clofibrate. This gene codifies a member of MAD family proteins belonging to the Max/Mad network which is involved in the control of various aspects of cell behaviour, including proliferation, differentiation, and apoptosis [41].

Clofibrate alone, at the concentration of 5 *μ*M, did not activate genes containing PPRE sequences, probably because of its low concentration. However, when added together with AS601245, 28 genes containing putative PPRE sequences were upregulated. This datum is in agreement with the hypothesis of an increase of PPAR transcriptional activity after JNK inhibition [42] and is further sustained by the results obtained in PPRE binding activity, which demonstrated a strong increase in DNA binding of cells treated with both AS601245 and clofibrate. The gene expression profile revealed that the expression of the PPAR*α* activator MED1 was increased by 1.8-fold in cells treated with both compounds only. MED1 (also referred as PBP/TRAP220/DRIP205) was shown as a critical component of the TRAP/DRIP/ARC/Mediator complex [43] which facilitates interaction of ligand-activated PPAR with RNA polymerase and the general basal transcription machinery to enhance the transcription of a specific set of genes [44–46]. Western Blot analysis confirmed the increase of MED1 protein in cells treated with 5 *μ*M clofibrate plus 0.1 *μ*M AS691245. This increase remained elevated in the cells treated with both compounds until 72 hours after the treatment. Moreover, an increase of MED1 expression occurred even in 50 *μ*M clofibrate-treated cells. These results paralleled with the increase of PPRE binding activity and may suggest that MED1 expression was involved in increasing the number of genes, containing PPRE sequences, activated by combined treatment. According to this hypothesis, we demonstrated that the expression of cyclin D1 protein, codified by a gene containing PPRE sequences that was upregulated after combined treatment, was increased, not only after combined treatment, but also after treatment with 50 *μ*M clofibrate.

The experiments performed in HepG2 cells showed that the single and combined treatment did not induce hepatotoxicity in human liver cells. Moreover, the analysis of the effect of these compounds on HepG2 cells proliferation indicated that there was no synergistic effect between clofibrate and AS601245. In this cell line, the pattern of proliferation-related protein expression was different from that detected in CaCO-2 cells. Indeed, in HepG-2 cells, the p21 expression was increased in clofibrate, AS601245 and clofibrate plus AS601245, treated cells. This result may be related to the fact that HepG2 cells have a wild-type p53 gene [47], whereas, in CaCo-2 cells, p53 is not expressed [48].

## 5. Conclusions

Taken together, our data demonstrate the effectiveness of combined treatments with PPAR*α* agonists and a JNK inhibitor in inducing apoptosis, differentiation, and PPRE binding activity in colon cancer cells and support the data suggesting a positive interaction between PPAR*α* ligands and anti-inflammatory agents in humans [49, 50]. The positive interaction originates from the modulation of several signalling pathways which were affected by the association of clofibrate and AS601245.

## Supplementary Material

Table A shows the complete affimetrix analysis of genes modulated by 5 *µ*CM Clofibrate, 0.1 *µ*M AS601245 and by the combined treatment with Clofibrate and AS601245 and listed on the basis of the p-value. Table B indicates gene relative expression detected by affymetrix and quantitative real-time reverse transcription PCR in Caco-2 cells treated with 5 *µ*M Clofibrate, 0.1 *µ*M AS601245 and combined treatment. Table C lists the genes affected by Clofibrate, by AS601245, and by the combined treatment, arranged with respect to the relative biological functions and listed on the basis of the p-value.

## Figures and Tables

**Table:** (a) Clofibrate

Gene ID	Gene name	Fold change
GANAB*	Glucosidase, alpha; neutral AB	+3.8
ATF6B	Activating transcription factor 6 beta	+2.8
SETD5	SET domain containing 5	+2.6
NFAT5	Nuclear factor of activated T cells 5, tonicity-responsive	+2.5
DLST	Dihydrolipoamide S-succinyltransferase (E2 component of 2-oxoglutarate complex)	+2.3
IL6ST	Interleukin 6 signal transducer (gp130, oncostatin M receptor)	+2.1
HNRNPA0	Heterogeneous nuclear ribonucleoprotein A0	+2.0
MEX3D	Mex-3 homolog D (*C. elegans*)	+1.6
CTSZ	Cathepsin Z	+1.5

STIP 1	Stress-induced phosphoprotein 1	−3.1
CLASP 1	Cytoplasmic linker-associated protein 1	−3.1
MACF 1	Microtubule-actin cross-linking factor 1	−2.9
COPA	Coatomer protein complex, subunit alpha	−2.7
HNRNPA1	Heterogeneous nuclear ribonucleoprotein A1	−2.6
SPSB2	SplA/ryanodine receptor domain and SOCS box containing 2	−2.3
TSR1	TSR1, 20S rRNA accumulation, homolog (*S. cerevisiae*)	−2.3
VIL1*	Villin 1	−2.3
CDH1	Cadherin 1, type 1, E-cadherin (epithelial)	−2.2
CRTAP	Cartilage-associated protein	−2.2

**Table:** (b) AS601245

Gene ID	Gene name	Fold change
CYP1A1	Cytochrome P450, family 1, subfamily A, polypeptide 1	+5.3
AP3D1	Adaptor-related protein complex 3, delta 1 subunit	+3.6
NFAT5	Nuclear factor of activated T cells 5, tonicity-responsive	+3.1
BMP2K	Bone morphogenetic protein 2 inducible kinase	+2.9
DLST	Dihydrolipoamide S-succinyltransferase (E2 component of 2-oxoglutarate complex)	+2.8
IL6ST	Interleukin 6 signal transducer (gp130, oncostatin M receptor)	+2.8
FAM76B	Family with sequence similarity 76, member B	+2.4
MGA	MAX gene associated	+2.4
THRAP3	Thyroid hormone receptor-associated protein 3	+2.4
GANAB	Glucosidase, alpha; neutral AB	+2.3

RPS27A	Ribosomal protein S27a	−4.2
HNRNPA1	Heterogeneous nuclear ribonucleoprotein A1	−4.1
WDR33	WD repeat domain 33	−3.9
EPN1	Epsin 1	−3.7
KLC2	Kinesin light chain 2	−3.6
STIP1	Stress-induced phosphoprotein 1	−3.6
SEPT9	Septin 9	−3.4
TFDP1	Transcription factor Dp-1	−3.1
LAD1	Ladinin 1	−3.1
CABIN 1	Calcineurin-binding protein 1	−3.1

**Table:** (c) Clofibrate + AS601245

Gene ID	Gene name	Fold change
CYP1A1	Cytochrome P450, family 1, subfamily A, polypeptide 1	+7.5
IL6ST	Interleukin 6 signal transducer (gp130, oncostatin M receptor)	+6.4
AP3D1	Adaptor-related protein complex 3, delta 1 subunit	+5.1
WASF2	WAS protein family, member 2	+4.1
VAPB	VAMP- (vesicle-associated membrane-protein-)associated protein B and C	+3.9
NFAT5*	Nuclear factor of activated T cells 5, tonicity-responsive	+3.8
THRA	Thyroid hormone receptor, alpha (erythroblastic leukemia viral (v-erb-a) oncogene homolog, avian)	+3.6
GANAB*	Glucosidase, alpha; neutral AB	+3.6
BDP1	B double prime 1, subunit of RNA polymerase III transcription initiation factor IIIB	+3.4
SMARCC1	SWI/SNF related, matrix associated, actin-dependent regulator of chromatin, subfamily c, member 1	+3.2

TRUB1	TruB pseudouridine (psi) synthase homolog 1 (*E. coli*)	−4.4
HNRNPA1	Heterogeneous nuclear ribonucleoprotein A1	−3.9
SLC39A14	Solute carrier family 39 (zinc transporter), member 14	−3.6
NXT2	Nuclear transport factor-2-like export factor 2	−3.4
MALAT1	Metastasis-associated lung adenocarcinoma transcript 1 (nonprotein coding)	−3.2
LARP5	La ribonucleoprotein domain family, member 5	−3.0
FGB	Fibrinogen beta chain	−2.8
ACTC1	Actin, alpha, cardiac muscle 1	−2.8
TFRC	Transferrin receptor (p90, CD71)	−2.8
RPS27A	Ribosomal protein S27a	−2.7

^b^The top ten genes upregulated and downregulated the most by 5 *μ*M Clofibrate (Table 1(a)), 0.1 *μ*M AS601245 (Table 1(b)), and combined treatment (Table 1(c)) with respect to DMSO-treated Caco-2 cells at 24 h.

**Table 2:** Upregulated genes containing PPRE sequences^b^.

	Gene ID	Gene name	Fold change
Clofibrate	None		

AS601245	CYP1A1	Cytochrome P450, family 1, subfamily A, polypeptide 1	5.3
	ITGB	Integrin, beta 1 (fibronectin receptor, beta polypeptide, antigen CD29 includes MDF2, MSK12)	2.9

Clofibrate + 601245	AMFR	Autocrine motility factor receptor	2.3
	ANKRD12	Ankyrin repeat domain 11	2.2
	CASP2	Caspase 2, apoptosis-related cysteine peptidase	2.7
	CBX5	Chromobox homolog 5 (HP1 alpha homolog, *Drosophila*)	1.4
	CCND1	Cyclin D1	1.9
	CYP1A1	Cytochrome P450, family 1, subfamily A, polypeptide 1	7.5
	DDX17	DEAD (Asp-Glu-Ala-Asp) box polypeptide 17	
	DST	Dystonin	1.8
	DUSP6	Dual specificity phosphatase 6	2.2
	EHBP1	EH domain-binding protein 1	2.4
	EWSR1	Ewing sarcoma breakpoint region 1	1.8
	FOSL1	FOS-like antigen 1	1.7
	LMCD	LIM and cysteine-rich domains 1	1.8
	MARCKS	MARCKS-like 1	2.5
	MCAM	Melanoma cell adhesion molecule	1.6
	MLL3	Myeloid/lymphoid or mixed-lineage leukemia 3	2.7
	NR2F2	Nuclear receptor subfamily 2, group F, member 2	2.4
	PCYOX1	Prenylcysteine oxidase 1	1.3
	RBM8A	RNA-binding motif protein 8A	2.5
	SPEN	Spen homolog, transcriptional regulator (Drosophila)	2.2
	TBL1XR1	Transducin (beta)-like 1 X-linked receptor 1	2.5
	TTC3	Tetratricopeptide repeat domain 32	1.8
	USP47	Ubiquitin specific peptidase 47	1.7
	ZNF281	Zinc finger protein 281	1.5
	ZNF294	Ring finger protein 160	1.9

^b^The genes harbouring PPRE sequences among the upregulated genes by treatment with 5 *μ*M clofibrate, 0.1 *μ*M AS601245, and combined treatment versus DMSO-treated Caco-2 cells at 24 h.
