# Supplementary material for: AS601245, an Anti-Inflammatory JNK Inhibitor, and Clofibrate Have a Synergistic Effect in Inducing Cell Responses and in Affecting the Gene Expression Profile in CaCo-2 Colon Cancer Cells
Source: PPAR Res. 2012 Feb 29;2012:269751. doi: 10.1155/2012/269751 (PMC3349252; doi:10.1155/2012/269751)
Supplement: Supplementary file 1 — Table A shows the complete affimetrix analysis of genes modulated by 5 µCM Clofibrate, 0.1 µM AS601245 and by the combined treatment with Clofibrate and AS601245 and listed on the basis of the p-value. Table B indicates gene relative expression detected by affymetrix and quantitative real-time reverse transcription PCR in Caco-2 cells treated with 5 µM Clofibrate, 0.1 µM AS601245 and combined treatment. Table C lists the genes affected by Clofibrate, by AS601245, and by the combined treatment, arranged with respect to the relative biological functions and listed on the basis of the p-value. [file 269751.f1.doc]

| Tab A AFFIMETRIX ANALYSIS OF GENES MODULATED BY CLOFIBRATE, AS601245 AND COMBINED TREATMENT | | | | | | | |
| --- | --- | --- | --- | --- | --- | --- | --- |
| **Gene Symbol** | **Gene Function- Entrez Gene Name** | **p-value Clofi** | **Fold Change Clofi** | **p-value 1245** | **Fold Change 1245** | **p-value Clofi+1245** | **Fold Change Clofi+1245** |
| A1CF | APOBEC1 complementation factor |  |  | 4,08E-08 | -1,785 |  |  |
| AAMP | angio-associated, migratory cell protein |  |  | 6,70E-12 | -2,225 |  |  |
| AARS | alanyl-tRNA synthetase |  |  | 4,49E-10 | -1,837 |  |  |
| AATF | apoptosis antagonizing transcription factor |  |  | 4,88E-13 | -2,052 |  |  |
| ABCB6 | ATP-binding cassette, sub-family B (MDR/TAP), member 6 |  |  | 1,12E-09 | -1,851 |  |  |
| ABCC1 | ATP-binding cassette, sub-family C (CFTR/MRP), member 1 |  |  | 5,69E-10 | -1,903 |  |  |
| ABCC3 | ATP-binding cassette, sub-family C (CFTR/MRP), member 3 |  |  | 4,19E-08 | -1,830 |  |  |
| ABCC6 | ATP-binding cassette, sub-family C (CFTR/MRP), member 6 |  |  | 2,69E-11 | -1,755 | 2,69E-11 | -1,608 |
| ABCC9 | ATP-binding cassette, sub-family C (CFTR/MRP), member 9 |  |  | 8,06E-10 | -2,073 | 8,06E-10 | -1,957 |
| ABCD3 | ATP-binding cassette, sub-family D (ALD), member 3 |  |  | 1,69E-14 | -1,841 | 1,69E-14 | -2,373 |
| ABCF1 | ATP-binding cassette, sub-family F (GCN20), member 1 |  |  | 7,16E-10 | -1,654 |  |  |
| ABCF2 | ATP-binding cassette, sub-family F (GCN20), member 2 |  |  | 1,95E-09 | -1,852 |  |  |
| ABHD12 | abhydrolase domain containing 12 |  |  | 1,69E-14 | -2,247 |  |  |
| ABHD14A | abhydrolase domain containing 14A |  |  | 1,38E-09 | -2,234 |  |  |
| ABHD14B | abhydrolase domain containing 14B |  |  | 1,89E-09 | -2,015 |  |  |
| ABHD2 | abhydrolase domain containing 2 |  |  |  |  | 1,17E-08 | 1,793 |
| ABHD3 | abhydrolase domain containing 3 |  |  | 2,06E-10 | -1,757 |  |  |
| ABHD4 | abhydrolase domain containing 4 |  |  | 7,39E-11 | -2,368 | 7,39E-11 | -2,153 |
| ABI1 | abl-interactor 1 |  |  | 1,14E-08 | -1,530 |  |  |
| ABLIM1 | actin binding LIM protein 1 |  |  | 1,69E-14 | -1,465 |  |  |
| ACAD8 | acyl-Coenzyme A dehydrogenase family, member 8 |  |  | 1,35E-08 | -1,604 |  |  |
| ACADM | acyl-Coenzyme A dehydrogenase, C-4 to C-12 straight chain |  |  | 9,51E-14 | -1,772 | 9,51E-14 | -1,582 |
| ACADVL | acyl-Coenzyme A dehydrogenase, very long chain |  |  | 1,65E-12 | -1,910 |  |  |
| ACAT2 | acetyl-Coenzyme A acetyltransferase 2 |  |  | 3,78E-13 | -1,707 | 3,78E-13 | -1,519 |
| ACLY | ATP citrate lyase |  |  | 1,69E-14 | -1,896 | 1,69E-14 | -1,841 |
| ACO2 | aconitase 2, mitochondrial |  |  | 2,87E-09 | -1,781 |  |  |
| ACOT2 | acyl-CoA thioesterase 2 | 1,69E-14 | -1,608 | 1,69E-14 | -2,383 | 1,69E-14 | -1,680 |
| ACOT7 | acyl-CoA thioesterase 7 | 4,12E-13 | -1,358 | 4,12E-13 | -1,981 |  |  |
| ACP1 | acid phosphatase 1, soluble |  |  | 9,31E-11 | -1,582 | 9,31E-11 | -1,516 |
| ACP1 | acid phosphatase 1, soluble |  |  | 1,01E-12 | -1,775 | 1,01E-12 | -1,498 |
| ACSS2 | acyl-CoA synthetase short-chain family member 2 |  |  | 7,61E-08 | -1,888 |  |  |
| ACTC1 | actin, alpha, cardiac muscle 1 |  |  |  |  | 2,28E-10 | -2,848 |
| ACTN1 | actinin, alpha 1 |  |  | 7,12E-11 | -1,701 |  |  |
| ACTN1 | actinin, alpha 1 |  |  | 2,53E-13 | -2,381 |  |  |
| ACTN4 | actinin, alpha 4 |  |  | 7,12E-10 | -1,721 |  |  |
| ACTR1A | ARP1 actin-related protein 1 homolog A, centractin alpha (yeast) |  |  | 1,51E-08 | -1,772 |  |  |
| ACTR1A | ARP1 actin-related protein 1 homolog A, centractin alpha (yeast) |  |  | 2,18E-09 | -1,826 |  |  |
| ACTR1B (includes EG:10120) | ARP1 actin-related protein 1 homolog B, centractin beta (yeast) |  |  | 4,85E-09 | -2,046 |  |  |
| ACTR3 | ARP3 actin-related protein 3 homolog (yeast) |  |  | 7,45E-08 | -1,438 |  |  |
| ACY1 | aminoacylase 1 |  |  | 1,69E-14 | -2,188 | 1,69E-14 | -1,488 |
| ACYP1 | acylphosphatase 1, erythrocyte (common) type |  |  | 1,62E-09 | -1,824 |  |  |
| ADAM10 | ADAM metallopeptidase domain 10 |  |  | 4,07E-11 | -1,566 |  |  |
| ADAM17 | ADAM metallopeptidase domain 17 |  |  | 6,00E-12 | -1,921 |  |  |
| ADD1 | adducin 1 (alpha) |  |  | 2,44E-13 | -2,070 |  |  |
| ADD3 | adducin 3 (gamma) |  |  | 8,29E-10 | -1,555 |  |  |
| ADD3 | adducin 3 (gamma) |  |  | 4,33E-10 | -1,568 |  |  |
| ADFP | adipose differentiation-related protein |  |  | 5,56E-08 | -1,592 |  |  |
| ADH5 (includes EG:128) | alcohol dehydrogenase 5 (class III), chi polypeptide |  |  | 7,66E-10 | -1,607 | 7,66E-10 | -1,587 |
| ADI1 | acireductone dioxygenase 1 |  |  | 7,27E-08 | -1,477 |  |  |
| ADK | adenosine kinase |  |  | 5,45E-11 | -1,645 |  |  |
| ADNP | activity-dependent neuroprotector homeobox |  |  | 8,68E-11 | -1,510 |  |  |
| ADNP | activity-dependent neuroprotector homeobox |  |  | 1,07E-13 | -2,017 |  |  |
| ADNP2 | ADNP homeobox 2 |  |  | 1,57E-11 | -1,983 |  |  |
| ADRM1 | adhesion regulating molecule 1 |  |  | 7,52E-11 | -1,664 |  |  |
| ADSL | adenylosuccinate lyase |  |  | 5,65E-09 | -1,638 |  |  |
| AGA | aspartylglucosaminidase |  |  | 3,11E-12 | -1,870 | 3,11E-12 | -1,838 |
| AGL | amylo-1, 6-glucosidase, 4-alpha-glucanotransferase |  |  | 1,30E-11 | -1,540 |  |  |
| AGPAT1 | 1-acylglycerol-3-phosphate O-acyltransferase 1 (lysophosphatidic acid acyltransferase, alpha) |  |  | 1,40E-08 | -1,896 |  |  |
| AGPAT1 | 1-acylglycerol-3-phosphate O-acyltransferase 1 (lysophosphatidic acid acyltransferase, alpha) |  |  | 8,14E-11 | -2,016 |  |  |
| AGPAT2 | 1-acylglycerol-3-phosphate O-acyltransferase 2 (lysophosphatidic acid acyltransferase, beta) | 2,36E-12 | -1,618 | 2,36E-12 | -2,189 |  |  |
| AGPS | alkylglycerone phosphate synthase |  |  | 2,53E-13 | -1,641 | 2,53E-13 | -1,373 |
| AHCY | S-adenosylhomocysteine hydrolase |  |  | 3,18E-10 | -1,690 |  |  |
| AHCYL1 | S-adenosylhomocysteine hydrolase-like 1 |  |  | 3,85E-10 | -1,606 |  |  |
| AHNAK | AHNAK nucleoprotein |  |  | 8,53E-09 | -1,484 |  |  |
| AIDA | axin interactor, dorsalization associated |  |  | 1,63E-13 | -1,657 | 1,63E-13 | -1,623 |
| AIFM1 | apoptosis-inducing factor, mitochondrion-associated, 1 |  |  | 2,37E-10 | -1,816 |  |  |
| AIG1 | androgen-induced 1 |  |  | 6,26E-09 | -1,752 |  |  |
| AIP | aryl hydrocarbon receptor interacting protein |  |  | 5,65E-09 | -1,958 |  |  |
| AIP | aryl hydrocarbon receptor interacting protein |  |  | 2,62E-08 | -1,870 |  |  |
| AK1 | adenylate kinase 1 |  |  | 2,68E-10 | -1,978 |  |  |
| AK2 | adenylate kinase 2 |  |  | 1,26E-08 | -1,574 |  |  |
| AK2 | adenylate kinase 2 |  |  | 8,75E-09 | -1,889 |  |  |
| AK3 | adenylate kinase 3 |  |  | 4,12E-11 | -1,652 |  |  |
| AKAP1 | A kinase (PRKA) anchor protein 1 |  |  | 1,18E-10 | -1,571 |  |  |
| AKAP13 | A kinase (PRKA) anchor protein 13 |  |  |  |  | 1,63E-08 | 2,822 |
| AKAP13 | A kinase (PRKA) anchor protein 13 |  |  |  |  | 2,22E-11 | 2,234 |
| AKAP9 | A kinase (PRKA) anchor protein (yotiao) 9 |  |  | 3,69E-08 | -1,562 |  |  |
| AKIRIN1 | akirin 1 |  |  |  |  | 1,88E-12 | 1,836 |
| AKR1B1 | aldo-keto reductase family 1, member B1 (aldose reductase) |  |  | 3,57E-11 | -1,814 |  |  |
| AKR1C3 | aldo-keto reductase family 1, member C3 (3-alpha hydroxysteroid dehydrogenase, type II) |  |  | 8,87E-09 | -1,510 |  |  |
| AKR7A2 | aldo-keto reductase family 7, member A2 (aflatoxin aldehyde reductase) |  |  | 2,07E-12 | -1,804 |  |  |
| AKT1 | v-akt murine thymoma viral oncogene homolog 1 |  |  | 5,76E-13 | -2,351 |  |  |
| AKTIP | AKT interacting protein |  |  | 9,29E-12 | -1,802 | 9,29E-12 | -1,922 |
| ALAS1 | aminolevulinate, delta-, synthase 1 |  |  | 7,12E-09 | -1,722 |  |  |
| ALDH5A1 | aldehyde dehydrogenase 5 family, member A1 |  |  | 1,78E-10 | -1,650 |  |  |
| ALDH7A1 | aldehyde dehydrogenase 7 family, member A1 |  |  | 1,44E-08 | -1,600 |  |  |
| ALDH9A1 | aldehyde dehydrogenase 9 family, member A1 |  |  | 9,68E-10 | -1,690 |  |  |
| ALDOA | aldolase A, fructose-bisphosphate |  |  | 1,12E-08 | -1,674 |  |  |
| ALG13 (includes EG:79868) | asparagine-linked glycosylation 13 homolog (S. cerevisiae) |  |  | 2,44E-13 | -1,683 | 2,44E-13 | -1,843 |
| ALG14 | asparagine-linked glycosylation 14 homolog (S. cerevisiae) |  |  | 1,36E-07 | -1,832 |  |  |
| ALG5 | asparagine-linked glycosylation 5, dolichyl-phosphate beta-glucosyltransferase homolog (S. cerevisiae) |  |  | 4,04E-08 | -1,526 |  |  |
| ALKBH5 | alkB, alkylation repair homolog 5 (E. coli) |  |  | 5,38E-10 | -1,657 |  |  |
| ALKBH7 | alkB, alkylation repair homolog 7 (E. coli) |  |  | 2,44E-08 | -1,942 |  |  |
| ALKBH7 | alkB, alkylation repair homolog 7 (E. coli) |  |  | 1,52E-08 | -2,002 |  |  |
| AMFR | autocrine motility factor receptor |  |  |  |  | 1,69E-14 | 2,345 |
| AMMECR1 | Alport syndrome, mental retardation, midface hypoplasia and elliptocytosis chromosomal region gene 1 |  |  | 7,55E-10 | -1,781 |  |  |
| AMZ2 | archaelysin family metallopeptidase 2 |  |  | 2,38E-10 | -1,686 |  |  |
| ANAPC1 | anaphase promoting complex subunit 1 |  |  | 4,14E-09 | -1,859 |  |  |
| ANAPC1 | anaphase promoting complex subunit 1 |  |  | 5,37E-10 | -1,790 |  |  |
| ANAPC10 | anaphase promoting complex subunit 10 |  |  |  |  | 3,05E-09 | -1,485 |
| ANAPC13 | anaphase promoting complex subunit 13 |  |  | 1,17E-08 | -1,499 |  |  |
| ANAPC5 | anaphase promoting complex subunit 5 |  |  | 1,16E-08 | -1,566 |  |  |
| ANK3 | ankyrin 3, node of Ranvier (ankyrin G) |  |  | 1,19E-08 | -1,623 |  |  |
| ANKRD10 | ankyrin repeat domain 10 |  |  | 1,69E-14 | -1,568 | 1,69E-14 | -2,446 |
| ANKRD11 | ankyrin repeat domain 11 |  |  |  |  | 1,80E-12 | 1,728 |
| ANKRD12 | ankyrin repeat domain 12 |  |  |  |  | 8,43E-11 | 2,204 |
| ANKRD16 | ankyrin repeat domain 16 |  |  | 6,94E-08 | -2,036 |  |  |
| ANKRD27 | ankyrin repeat domain 27 (VPS9 domain) |  |  | 3,53E-10 | -1,535 |  |  |
| ANKRD39 | ankyrin repeat domain 39 |  |  | 4,54E-08 | -1,739 |  |  |
| ANLN | anillin, actin binding protein |  |  | 1,02E-09 | -1,478 |  |  |
| ANP32E | acidic (leucine-rich) nuclear phosphoprotein 32 family, member E |  |  | 1,69E-14 | -1,958 | 1,69E-14 | -2,574 |
| ANXA1 | annexin A1 |  |  | 8,38E-10 | -1,645 |  |  |
| ANXA3 | annexin A3 |  |  | 7,47E-11 | -1,545 |  |  |
| ANXA4 | annexin A4 |  |  | 5,57E-10 | -1,514 | 5,57E-10 | -1,425 |
| ANXA4 | annexin A4 |  |  | 9,28E-12 | -1,592 |  |  |
| ANXA9 | annexin A9 |  |  | 3,29E-10 | -2,492 | 3,29E-10 | -2,147 |
| ANXA9 | annexin A9 | 1,69E-14 | -1,502 | 1,69E-14 | -2,409 | 1,69E-14 | -2,207 |
| AP1M1 | adaptor-related protein complex 1, mu 1 subunit |  |  | 1,69E-10 | -1,909 |  |  |
| AP1M2 | adaptor-related protein complex 1, mu 2 subunit |  |  | 3,01E-11 | -2,117 |  |  |
| AP1S2 | adaptor-related protein complex 1, sigma 2 subunit |  |  | 4,59E-08 | -1,732 |  |  |
| AP1S3 (includes EG:130340) | adaptor-related protein complex 1, sigma 3 subunit | 1,69E-14 | -1,375 | 1,69E-14 | -1,763 | 1,69E-14 | -1,946 |
| AP1S3 (includes EG:130340) | adaptor-related protein complex 1, sigma 3 subunit | 1,73E-13 | -1,332 | 1,73E-13 | -1,519 | 1,73E-13 | -1,791 |
| AP1S3 (includes EG:130340) | adaptor-related protein complex 1, sigma 3 subunit |  |  | 2,41E-12 | -1,623 | 2,41E-12 | -1,851 |
| AP1S3 (includes EG:130340) | adaptor-related protein complex 1, sigma 3 subunit |  |  | 1,65E-12 | -1,926 |  |  |
| AP2A2 | adaptor-related protein complex 2, alpha 2 subunit |  |  | 8,77E-09 | -1,639 |  |  |
| AP2B1 | adaptor-related protein complex 2, beta 1 subunit |  |  | 3,19E-09 | -1,568 |  |  |
| AP2M1 | adaptor-related protein complex 2, mu 1 subunit |  |  | 6,12E-12 | -1,800 |  |  |
| AP3D1 | adaptor-related protein complex 3, delta 1 subunit |  |  | 1,69E-14 | 3,634 | 1,69E-14 | 5,056 |
| AP3M1 | adaptor-related protein complex 3, mu 1 subunit |  |  | 8,52E-08 | -1,592 |  |  |
| APEH | N-acylaminoacyl-peptide hydrolase |  |  | 1,41E-10 | -1,805 |  |  |
| API5 | apoptosis inhibitor 5 |  |  |  |  | 2,16E-10 | 1,699 |
| APLP2 | amyloid beta (A4) precursor-like protein 2 |  |  | 2,93E-09 | -1,440 |  |  |
| APOA1 | apolipoprotein A-I |  |  | 2,17E-09 | -1,730 |  |  |
| APOA1BP | apolipoprotein A-I binding protein |  |  | 5,35E-11 | -1,798 |  |  |
| APOA2 | apolipoprotein A-II |  |  | 7,00E-09 | -1,461 |  |  |
| APOBEC3B | apolipoprotein B mRNA editing enzyme, catalytic polypeptide-like 3B |  |  | 1,52E-12 | -1,591 |  |  |
| APOE | apolipoprotein E |  |  | 1,42E-08 | -1,764 |  |  |
| APOE | apolipoprotein E |  |  | 5,64E-13 | -2,196 |  |  |
| APOH | apolipoprotein H (beta-2-glycoprotein I) |  |  | 2,98E-12 | -1,829 | 2,98E-12 | -1,564 |
| APOOL | apolipoprotein O-like |  |  |  |  | 3,20E-08 | -2,318 |
| APPBP2 | amyloid beta precursor protein (cytoplasmic tail) binding protein 2 |  |  | 5,59E-08 | -1,649 |  |  |
| APPL2 | adaptor protein, phosphotyrosine interaction, PH domain and leucine zipper containing 2 |  |  | 2,05E-08 | -1,634 |  |  |
| APRT | adenine phosphoribosyltransferase |  |  | 6,07E-11 | -1,919 |  |  |
| APRT | adenine phosphoribosyltransferase |  |  | 5,47E-08 | -1,608 |  |  |
| AQP11 | aquaporin 11 |  |  | 6,08E-10 | -1,678 | 6,08E-10 | -1,668 |
| AQR | aquarius homolog (mouse) |  |  | 2,43E-09 | -1,589 |  |  |
| ARD1A | ARD1 homolog A, N-acetyltransferase (S. cerevisiae) |  |  | 4,67E-09 | -1,900 |  |  |
| AREGB | amphiregulin B |  |  |  |  | 1,07E-09 | 1,627 |
| ARF1 | ADP-ribosylation factor 1 | 1,69E-14 | -1,546 | 1,69E-14 | -1,998 | 1,69E-14 | -1,903 |
| ARF3 | ADP-ribosylation factor 3 |  |  | 1,64E-10 | -1,693 |  |  |
| ARF3 | ADP-ribosylation factor 3 |  |  | 9,30E-09 | -1,647 |  |  |
| ARF4 | ADP-ribosylation factor 4 |  |  | 1,16E-08 | -1,481 |  |  |
| ARFGAP2 | ADP-ribosylation factor GTPase activating protein 2 |  |  | 2,67E-09 | -1,828 |  |  |
| ARGLU1 | arginine and glutamate rich 1 |  |  | 2,18E-09 | -1,535 |  |  |
| ARHGAP18 | Rho GTPase activating protein 18 |  |  |  |  | 1,69E-14 | 2,077 |
| ARHGAP27 | Rho GTPase activating protein 27 |  |  | 1,02E-11 | -2,425 | 1,02E-11 | -1,586 |
| ARHGAP29 | Rho GTPase activating protein 29 |  |  | 8,08E-10 | -1,598 |  |  |
| ARHGAP5 | Rho GTPase activating protein 5 |  |  |  |  | 4,18E-10 | -1,684 |
| ARHGAP8 | Rho GTPase activating protein 8 |  |  | 1,28E-08 | -1,575 |  |  |
| ARHGDIB | Rho GDP dissociation inhibitor (GDI) beta |  |  | 5,39E-09 | -2,065 |  |  |
| ARHGEF7 | Rho guanine nucleotide exchange factor (GEF) 7 |  |  | 3,22E-08 | -1,661 |  |  |
| ARHGEF7 | Rho guanine nucleotide exchange factor (GEF) 7 |  |  |  |  | 9,78E-07 | -1,919 |
| ARID1B | AT rich interactive domain 1B (SWI1-like) |  |  | 8,03E-09 | -1,530 |  |  |
| ARID3A | AT rich interactive domain 3A (BRIGHT-like) |  |  | 6,22E-09 | -1,793 |  |  |
| ARID5B | AT rich interactive domain 5B (MRF1-like) |  |  | 8,48E-10 | -1,609 |  |  |
| ARL1 | ADP-ribosylation factor-like 1 |  |  | 1,76E-09 | -1,441 | 1,76E-09 | -1,494 |
| ARL3 | ADP-ribosylation factor-like 3 |  |  | 2,97E-08 | -1,625 |  |  |
| ARL4C | ADP-ribosylation factor-like 4C |  |  | 4,51E-11 | -1,881 |  |  |
| ARL5A | ADP-ribosylation factor-like 5A |  |  | 1,69E-14 | -1,964 | 1,69E-14 | -1,382 |
| ARL8B | ADP-ribosylation factor-like 8B |  |  | 3,63E-08 | -1,512 |  |  |
| ARL8B | ADP-ribosylation factor-like 8B |  |  | 4,20E-10 | -1,624 | 4,20E-10 | -1,456 |
| ARMCX6 | armadillo repeat containing, X-linked 6 |  |  | 1,49E-08 | -1,680 |  |  |
| ARPC1A | actin related protein 2/3 complex, subunit 1A, 41kDa |  |  | 9,92E-09 | -1,503 |  |  |
| ARPC3 | actin related protein 2/3 complex, subunit 3, 21kDa |  |  | 1,69E-14 | -1,840 | 1,69E-14 | -1,441 |
| ARPP-19 | cyclic AMP phosphoprotein, 19 kD |  |  | 6,79E-11 | -1,565 |  |  |
| ARRB1 | arrestin, beta 1 |  |  | 2,15E-08 | -2,556 |  |  |
| ASAP1 | ArfGAP with SH3 domain, ankyrin repeat and PH domain 1 |  |  | 2,53E-12 | -1,699 |  |  |
| ASB8 | ankyrin repeat and SOCS box-containing 8 |  |  | 7,39E-11 | -1,733 | 7,39E-11 | -1,464 |
| ASF1A | ASF1 anti-silencing function 1 homolog A (S. cerevisiae) |  |  | 4,93E-12 | -1,633 | 4,93E-12 | -1,659 |
| ASF1B | ASF1 anti-silencing function 1 homolog B (S. cerevisiae) |  |  | 8,27E-09 | -1,711 |  |  |
| ASH1L | ash1 (absent, small, or homeotic)-like (Drosophila) |  |  | 9,33E-12 | -1,543 |  |  |
| ASH1L | ash1 (absent, small, or homeotic)-like (Drosophila) |  |  | 1,44E-07 | -1,654 |  |  |
| ASL | argininosuccinate lyase |  |  | 3,44E-10 | -1,778 |  |  |
| ASNA1 | arsA arsenite transporter, ATP-binding, homolog 1 (bacterial) |  |  | 1,13E-12 | -2,167 |  |  |
| ASPM | asp (abnormal spindle) homolog, microcephaly associated (Drosophila) |  |  |  |  | 2,71E-13 | 1,538 |
| ASS1 | argininosuccinate synthetase 1 |  |  | 3,40E-09 | -1,866 |  |  |
| ATAD2 | ATPase family, AAA domain containing 2 |  |  | 4,96E-11 | -1,721 | 4,96E-11 | -1,562 |
| ATAD3A | ATPase family, AAA domain containing 3A |  |  | 2,01E-09 | -2,446 |  |  |
| ATF1 | activating transcription factor 1 |  |  | 2,82E-08 | -1,630 |  |  |
| ATF6B | activating transcription factor 6 beta | 4,97E-08 | 2,839 |  |  |  |  |
| ATF7IP2 | activating transcription factor 7 interacting protein 2 |  |  | 1,07E-08 | -1,904 |  |  |
| ATG3 | ATG3 autophagy related 3 homolog (S. cerevisiae) |  |  | 6,44E-08 | -1,438 |  |  |
| ATG4B | ATG4 autophagy related 4 homolog B (S. cerevisiae) |  |  | 6,39E-09 | -2,070 |  |  |
| ATG5 | ATG5 autophagy related 5 homolog (S. cerevisiae) |  |  | 1,69E-14 | -1,690 | 1,69E-14 | -1,810 |
| ATL3 | atlastin GTPase 3 |  |  | 7,91E-09 | -1,753 |  |  |
| ATM | ataxia telangiectasia mutated |  |  | 9,94E-10 | -1,910 |  |  |
| ATOX1 | ATX1 antioxidant protein 1 homolog (yeast) |  |  | 1,57E-12 | -1,942 |  |  |
| ATP1A1 | ATPase, Na+/K+ transporting, alpha 1 polypeptide |  |  | 5,77E-08 | -1,604 |  |  |
| ATP2C1 | ATPase, Ca++ transporting, type 2C, member 1 |  |  | 7,07E-14 | -1,805 |  |  |
| ATP5C1 | ATP synthase, H+ transporting, mitochondrial F1 complex, gamma polypeptide 1 |  |  | 3,96E-09 | -1,479 |  |  |
| ATP5C1 | ATP synthase, H+ transporting, mitochondrial F1 complex, gamma polypeptide 1 |  |  | 1,08E-08 | -1,455 |  |  |
| ATP5C1 | ATP synthase, H+ transporting, mitochondrial F1 complex, gamma polypeptide 1 |  |  | 1,06E-10 | -1,518 | 1,06E-10 | -1,422 |
| ATP5D | ATP synthase, H+ transporting, mitochondrial F1 complex, delta subunit |  |  | 1,69E-14 | -2,519 |  |  |
| ATP5F1 | ATP synthase, H+ transporting, mitochondrial F0 complex, subunit B1 |  |  | 4,92E-09 | -1,524 |  |  |
| ATP5G1 | ATP synthase, H+ transporting, mitochondrial F0 complex, subunit C1 (subunit 9) |  |  | 6,71E-08 | -1,661 |  |  |
| ATP5G2 | ATP synthase, H+ transporting, mitochondrial F0 complex, subunit C2 (subunit 9) |  |  | 1,70E-12 | -1,837 |  |  |
| ATP5H (includes EG:10476) | ATP synthase, H+ transporting, mitochondrial F0 complex, subunit d |  |  | 1,50E-10 | -1,524 |  |  |
| ATP5I | ATP synthase, H+ transporting, mitochondrial F0 complex, subunit E |  |  | 1,12E-09 | -1,710 |  |  |
| ATP5J | ATP synthase, H+ transporting, mitochondrial F0 complex, subunit F6 |  |  | 1,55E-08 | -1,474 |  |  |
| ATP5J2 | ATP synthase, H+ transporting, mitochondrial F0 complex, subunit F2 |  |  | 2,02E-11 | -1,836 |  |  |
| ATP5S | ATP synthase, H+ transporting, mitochondrial F0 complex, subunit s (factor B) |  |  | 4,21E-09 | -1,747 |  |  |
| ATP6AP1 | ATPase, H+ transporting, lysosomal accessory protein 1 |  |  | 2,32E-09 | -1,753 |  |  |
| ATP6AP2 | ATPase, H+ transporting, lysosomal accessory protein 2 |  |  | 4,98E-09 | -1,454 |  |  |
| ATP6V0C | ATPase, H+ transporting, lysosomal 16kDa, V0 subunit c |  |  | 9,72E-11 | -1,817 |  |  |
| ATP6V0E2 | ATPase, H+ transporting V0 subunit e2 |  |  | 2,33E-09 | -1,787 |  |  |
| ATP6V1A | ATPase, H+ transporting, lysosomal 70kDa, V1 subunit A | 2,41E-09 | -1,537 | 2,41E-09 | -1,498 | 2,41E-09 | -1,508 |
| ATP6V1D | ATPase, H+ transporting, lysosomal 34kDa, V1 subunit D |  |  | 2,24E-09 | -1,590 |  |  |
| ATP6V1F | ATPase, H+ transporting, lysosomal 14kDa, V1 subunit F |  |  | 6,44E-10 | -1,779 |  |  |
| ATP7B | ATPase, Cu++ transporting, beta polypeptide |  |  | 1,69E-14 | -2,330 | 1,69E-14 | -1,955 |
| ATP8B1 | ATPase, class I, type 8B, member 1 |  |  | 2,68E-10 | -1,670 |  |  |
| ATPAF2 | ATP synthase mitochondrial F1 complex assembly factor 2 |  |  | 1,81E-10 | -2,232 |  |  |
| ATPIF1 | ATPase inhibitory factor 1 |  |  | 4,36E-09 | -1,841 |  |  |
| ATRX | alpha thalassemia/mental retardation syndrome X-linked (RAD54 homolog, S. cerevisiae) |  |  |  |  | 1,69E-14 | 2,276 |
| ATXN1L | ataxin 1-like |  |  | 1,51E-11 | -1,670 | 1,51E-11 | -1,408 |
| AURKA | aurora kinase A |  |  | 7,35E-11 | -1,774 |  |  |
| AURKA | aurora kinase A |  |  | 1,21E-11 | -1,634 | 1,21E-11 | -1,398 |
| AURKAIP1 | aurora kinase A interacting protein 1 |  |  | 3,50E-09 | -1,623 |  |  |
| AURKAIP1 | aurora kinase A interacting protein 1 |  |  | 3,78E-09 | -1,600 |  |  |
| AVL9 | AVL9 homolog (S. cerevisiase) |  |  | 1,75E-10 | -1,472 |  |  |
| AXIN2 | axin 2 |  |  | 3,94E-13 | -1,852 | 3,94E-13 | -1,579 |
| AZI2 | 5-azacytidine induced 2 | 3,14E-14 | -1,432 | 3,14E-14 | -2,219 | 3,14E-14 | -1,912 |
| B2M | beta-2-microglobulin |  |  | 6,60E-10 | -1,442 |  |  |
| B3GNT1 | UDP-GlcNAc:betaGal beta-1,3-N-acetylglucosaminyltransferase 1 |  |  | 7,92E-12 | -1,923 |  |  |
| B4GALT1 | UDP-Gal:betaGlcNAc beta 1,4- galactosyltransferase, polypeptide 1 |  |  |  |  | 1,69E-14 | 1,417 |
| B4GALT1 | UDP-Gal:betaGlcNAc beta 1,4- galactosyltransferase, polypeptide 1 |  |  | 1,94E-12 | -1,572 | 1,94E-12 | -1,885 |
| B4GALT3 | UDP-Gal:betaGlcNAc beta 1,4- galactosyltransferase, polypeptide 3 |  |  | 1,01E-11 | -2,071 |  |  |
| BAG2 | BCL2-associated athanogene 2 |  |  | 4,96E-13 | -1,565 | 4,96E-13 | -1,505 |
| BAG5 | BCL2-associated athanogene 5 |  |  | 4,52E-08 | -1,587 |  |  |
| BANF1 | barrier to autointegration factor 1 |  |  | 7,56E-11 | -1,851 |  |  |
| BAT2D1 | BAT2 domain containing 1 |  |  |  |  | 1,07E-07 | 1,341 |
| BAT2L | HLA-B associated transcript 2-like |  |  | 4,88E-10 | -2,207 |  |  |
| BAT3 | HLA-B associated transcript 3 |  |  | 3,33E-10 | -1,795 |  |  |
| BAT4 | HLA-B associated transcript 4 |  |  | 4,25E-10 | -1,756 |  |  |
| BBS1 | Bardet-Biedl syndrome 1 |  |  | 6,18E-08 | -1,612 |  |  |
| BCAP29 | B-cell receptor-associated protein 29 | 9,51E-14 | -1,619 | 9,51E-14 | -2,147 |  |  |
| BCAS2 | breast carcinoma amplified sequence 2 |  |  | 2,02E-09 | -1,658 |  |  |
| BCAT1 | branched chain aminotransferase 1, cytosolic |  |  | 1,69E-14 | -1,867 | 1,69E-14 | -1,520 |
| BCAT2 | branched chain aminotransferase 2, mitochondrial |  |  | 1,28E-12 | -2,060 |  |  |
| BCCIP | BRCA2 and CDKN1A interacting protein |  |  | 1,42E-07 | -1,744 |  |  |
| BCL2L1 | BCL2-like 1 |  |  | 1,31E-07 | -1,928 |  |  |
| BCL2L1 | BCL2-like 1 |  |  | 2,41E-08 | -2,157 |  |  |
| BCL2L13 | BCL2-like 13 (apoptosis facilitator) |  |  | 2,66E-09 | -1,876 |  |  |
| BCL7C | B-cell CLL/lymphoma 7C | 2,44E-13 | -1,876 | 2,44E-13 | -2,416 |  |  |
| BCLAF1 | BCL2-associated transcription factor 1 |  |  | 1,07E-10 | -1,492 |  |  |
| BDP1 | B double prime 1, subunit of RNA polymerase III transcription initiation factor IIIB |  |  |  |  | 1,35E-09 | 3,371 |
| BECN1 | beclin 1, autophagy related |  |  | 2,32E-08 | -1,681 |  |  |
| BICD1 | bicaudal D homolog 1 (Drosophila) |  |  | 3,64E-09 | -1,627 |  |  |
| BICD2 | bicaudal D homolog 2 (Drosophila) |  |  | 2,10E-07 | -1,575 |  |  |
| BID | BH3 interacting domain death agonist |  |  | 6,96E-13 | -2,073 |  |  |
| BIN1 | bridging integrator 1 |  |  | 2,44E-09 | -2,083 |  |  |
| BIN1 | bridging integrator 1 |  |  | 1,04E-08 | -1,879 |  |  |
| BIRC3 | baculoviral IAP repeat-containing 3 |  |  |  |  | 1,69E-14 | 2,054 |
| BLCAP | bladder cancer associated protein |  |  | 6,20E-08 | -1,645 |  |  |
| BLMH | bleomycin hydrolase |  |  | 1,72E-08 | -2,061 |  |  |
| BLVRB | biliverdin reductase B (flavin reductase (NADPH)) |  |  | 2,77E-08 | -1,723 |  |  |
| BMI1 | BMI1 polycomb ring finger oncogene |  |  | 2,41E-09 | -1,642 |  |  |
| BMP2 | bone morphogenetic protein 2 |  |  | 5,28E-09 | -1,741 |  |  |
| BMP2K (includes EG:55589) | BMP2 inducible kinase |  |  | 1,69E-14 | 2,889 | 1,69E-14 | 3,177 |
| BMPR1A | bone morphogenetic protein receptor, type IA |  |  | 9,92E-13 | -2,009 |  |  |
| BNIP3L | BCL2/adenovirus E1B 19kDa interacting protein 3-like |  |  | 1,77E-11 | -1,803 |  |  |
| BOP1 | block of proliferation 1 |  |  | 2,55E-11 | -2,326 |  |  |
| BPTF | bromodomain PHD finger transcription factor |  |  | 3,21E-10 | -1,806 |  |  |
| BRCA2 | breast cancer 2, early onset |  |  |  |  | 1,31E-06 | -1,527 |
| BRCC3 | BRCA1/BRCA2-containing complex, subunit 3 |  |  | 7,07E-14 | -1,991 |  |  |
| BRD2 | bromodomain containing 2 |  |  |  |  | 2,72E-12 | 1,486 |
| BRD4 | bromodomain containing 4 |  |  |  |  | 1,66E-08 | 1,409 |
| BRD4 | bromodomain containing 4 |  |  | 4,49E-14 | -2,235 |  |  |
| BRD4 | bromodomain containing 4 |  |  |  |  | 3,22E-09 | 1,901 |
| BRD7 | bromodomain containing 7 |  |  | 7,75E-11 | -1,610 |  |  |
| BRD8 | bromodomain containing 8 |  |  | 4,18E-09 | -1,555 |  |  |
| BRD9 | bromodomain containing 9 |  |  | 4,39E-12 | -1,782 |  |  |
| BRI3 | brain protein I3 |  |  | 1,69E-14 | -1,959 | 1,69E-14 | -1,412 |
| BRMS1 | breast cancer metastasis suppressor 1 |  |  | 5,04E-10 | -1,907 |  |  |
| BST2 | bone marrow stromal cell antigen 2 | 1,69E-14 | -1,768 | 1,69E-14 | -2,694 | 1,69E-14 | -1,449 |
| BTBD1 | BTB (POZ) domain containing 1 |  |  | 3,54E-10 | -1,693 |  |  |
| BTBD3 | BTB (POZ) domain containing 3 |  |  | 5,84E-09 | -1,812 |  |  |
| BTBD7 | BTB (POZ) domain containing 7 |  |  |  |  | 6,71E-10 | 1,735 |
| BTG1 | B-cell translocation gene 1, anti-proliferative | 1,69E-14 | -1,301 | 1,69E-14 | -2,006 | 1,69E-14 | -1,709 |
| BTG3 | BTG family, member 3 |  |  | 1,70E-08 | -1,541 |  |  |
| BTG3 | BTG family, member 3 |  |  | 1,08E-09 | -1,963 |  |  |
| BUB1 | budding uninhibited by benzimidazoles 1 homolog (yeast) |  |  | 3,14E-14 | -1,643 | 3,14E-14 | -1,508 |
| BUB1 | budding uninhibited by benzimidazoles 1 homolog (yeast) |  |  | 4,35E-09 | -1,583 | 4,35E-09 | -1,714 |
| BUB1B | budding uninhibited by benzimidazoles 1 homolog beta (yeast) |  |  | 2,38E-10 | -1,677 |  |  |
| BUB3 | budding uninhibited by benzimidazoles 3 homolog (yeast) |  |  | 3,96E-09 | -1,562 |  |  |
| BUD13 | BUD13 homolog (S. cerevisiae) | 8,58E-10 | -1,407 | 8,58E-10 | -1,770 |  |  |
| BUD31 (includes EG:8896) | BUD31 homolog (S. cerevisiae) |  |  | 3,06E-08 | -1,606 |  |  |
| BXDC5 | brix domain containing 5 | 5,37E-11 | -1,370 | 5,37E-11 | -1,815 |  |  |
| C10ORF104 | chromosome 10 open reading frame 104 |  |  | 1,22E-07 | -1,492 |  |  |
| C10ORF104 | chromosome 10 open reading frame 104 |  |  | 1,93E-08 | -1,615 |  |  |
| C10ORF119 | chromosome 10 open reading frame 119 |  |  | 8,97E-11 | -1,618 |  |  |
| C10ORF2 | chromosome 10 open reading frame 2 |  |  | 2,31E-08 | -1,806 |  |  |
| C10ORF32 | chromosome 10 open reading frame 32 |  |  | 1,34E-11 | -1,754 |  |  |
| C10ORF47 | chromosome 10 open reading frame 47 |  |  | 1,89E-08 | -1,808 |  |  |
| C10ORF58 | chromosome 10 open reading frame 58 | 8,35E-09 | -1,428 | 8,35E-09 | -1,487 |  |  |
| C10ORF84 | chromosome 10 open reading frame 84 |  |  | 6,07E-11 | -2,051 |  |  |
| C11ORF10 | chromosome 11 open reading frame 10 |  |  | 2,32E-12 | -1,663 |  |  |
| C11ORF52 | chromosome 11 open reading frame 52 |  |  | 1,73E-08 | -2,185 |  |  |
| C11ORF59 | chromosome 11 open reading frame 59 |  |  | 1,69E-14 | -1,936 |  |  |
| C11ORF73 | chromosome 11 open reading frame 73 |  |  | 1,69E-14 | -2,128 | 1,69E-14 | -1,505 |
| C11ORF73 | chromosome 11 open reading frame 73 |  |  | 1,92E-09 | -1,584 |  |  |
| C11ORF75 | chromosome 11 open reading frame 75 |  |  | 1,32E-09 | -1,639 | 1,32E-09 | -1,352 |
| C11ORF83 | chromosome 11 open reading frame 83 |  |  | 4,50E-12 | -1,974 |  |  |
| C12ORF10 | chromosome 12 open reading frame 10 |  |  | 2,06E-11 | -1,936 |  |  |
| C12ORF24 | chromosome 12 open reading frame 24 |  |  | 3,72E-10 | -1,870 |  |  |
| C12ORF32 | chromosome 12 open reading frame 32 |  |  | 1,48E-10 | -1,513 | 1,48E-10 | -1,764 |
| C12ORF32 | chromosome 12 open reading frame 32 |  |  | 9,23E-12 | -1,999 |  |  |
| C12ORF34 | chromosome 12 open reading frame 34 |  |  | 1,89E-09 | -2,079 |  |  |
| C12ORF47 | chromosome 12 open reading frame 47 |  |  | 1,69E-14 | -2,140 | 1,69E-14 | -1,842 |
| C12ORF47 | chromosome 12 open reading frame 47 |  |  | 5,81E-09 | -1,667 |  |  |
| C12ORF59 | chromosome 12 open reading frame 59 |  |  | 1,16E-11 | -2,071 |  |  |
| C12ORF62 | chromosome 12 open reading frame 62 |  |  | 4,34E-08 | -1,704 |  |  |
| C13ORF15 | chromosome 13 open reading frame 15 |  |  | 6,20E-13 | -1,899 |  |  |
| C13ORF23 | chromosome 13 open reading frame 23 |  |  | 1,02E-08 | -1,559 |  |  |
| C14ORF1 | chromosome 14 open reading frame 1 |  |  | 2,39E-11 | -1,700 |  |  |
| C14ORF108 | MU-2/AP1M2 domain containing, death-inducing |  |  | 1,90E-09 | -1,463 | 1,90E-09 | -1,873 |
| C14ORF109 | chromosome 14 open reading frame 109 |  |  | 2,93E-12 | -1,860 | 2,93E-12 | -1,576 |
| C14ORF142 | chromosome 14 open reading frame 142 |  |  | 1,69E-14 | -1,778 | 1,69E-14 | -1,961 |
| C14ORF147 | chromosome 14 open reading frame 147 |  |  | 8,05E-12 | -1,700 |  |  |
| C14ORF2 | chromosome 14 open reading frame 2 |  |  | 8,03E-09 | -1,598 |  |  |
| C14ORF94 | chromosome 14 open reading frame 94 |  |  | 4,44E-09 | -1,799 |  |  |
| C15ORF15 | chromosome 15 open reading frame 15 |  |  | 4,82E-09 | -1,642 |  |  |
| C15ORF23 | chromosome 15 open reading frame 23 |  |  | 2,13E-09 | -1,638 |  |  |
| C15ORF61 | chromosome 15 open reading frame 61 |  |  | 4,86E-11 | -1,629 | 4,86E-11 | -1,594 |
| C16ORF14 | chromosome 16 open reading frame 14 |  |  | 3,79E-09 | -1,844 |  |  |
| C16ORF35 | chromosome 16 open reading frame 35 |  |  | 2,37E-10 | -2,233 |  |  |
| C16ORF58 | chromosome 16 open reading frame 58 |  |  | 4,21E-13 | -3,121 | 4,21E-13 | -2,587 |
| C16ORF68 | chromosome 16 open reading frame 68 |  |  | 2,70E-09 | -2,152 |  |  |
| C16ORF70 | chromosome 16 open reading frame 70 |  |  | 1,43E-07 | -1,739 |  |  |
| C16ORF75 | chromosome 16 open reading frame 75 |  |  | 2,48E-09 | -1,796 |  |  |
| C16ORF79 | chromosome 16 open reading frame 79 | 1,75E-08 | -1,901 | 1,75E-08 | -2,900 |  |  |
| C16ORF88 | chromosome 16 open reading frame 88 |  |  |  |  | 5,79E-08 | 1,880 |
| C17ORF101 | chromosome 17 open reading frame 101 |  |  | 2,81E-12 | -1,635 |  |  |
| C17ORF37 | chromosome 17 open reading frame 37 |  |  | 4,67E-10 | -1,916 |  |  |
| C17ORF80 | chromosome 17 open reading frame 80 |  |  | 1,08E-10 | -1,935 |  |  |
| C17ORF81 | chromosome 17 open reading frame 81 |  |  | 3,14E-11 | -1,757 |  |  |
| C17ORF90 | chromosome 17 open reading frame 90 |  |  | 5,20E-09 | -1,661 |  |  |
| C17ORF95 | chromosome 17 open reading frame 95 |  |  | 3,14E-14 | -1,797 | 3,14E-14 | -1,522 |
| C18ORF10 | chromosome 18 open reading frame 10 |  |  | 2,39E-10 | -1,861 |  |  |
| C18ORF10 | chromosome 18 open reading frame 10 |  |  | 9,71E-13 | -1,961 | 9,71E-13 | -1,683 |
| C18ORF24 | chromosome 18 open reading frame 24 |  |  | 5,50E-13 | -2,042 | 5,50E-13 | -1,520 |
| C19ORF10 | chromosome 19 open reading frame 10 | 1,69E-14 | -1,485 | 1,69E-14 | -1,962 | 1,69E-14 | -1,359 |
| C19ORF10 | chromosome 19 open reading frame 10 | 1,91E-12 | -1,618 | 1,91E-12 | -1,849 |  |  |
| C19ORF22 | chromosome 19 open reading frame 22 |  |  | 2,28E-08 | -1,859 |  |  |
| C19ORF33 | chromosome 19 open reading frame 33 |  |  | 6,47E-11 | -1,758 |  |  |
| C19ORF42 | chromosome 19 open reading frame 42 |  |  | 7,24E-09 | -1,664 |  |  |
| C19ORF43 | chromosome 19 open reading frame 43 |  |  | 6,55E-12 | -1,778 |  |  |
| C19ORF60 | chromosome 19 open reading frame 60 |  |  | 5,68E-08 | -1,793 |  |  |
| C19ORF62 | chromosome 19 open reading frame 62 |  |  | 4,80E-11 | -1,917 |  |  |
| C19ORF70 | chromosome 19 open reading frame 70 |  |  | 3,43E-09 | -1,720 |  |  |
| C1GALT1C1 | C1GALT1-specific chaperone 1 |  |  | 1,73E-08 | -1,575 |  |  |
| C1ORF103 | chromosome 1 open reading frame 103 |  |  | 5,77E-14 | -1,706 |  |  |
| C1ORF122 | chromosome 1 open reading frame 122 |  |  | 3,51E-12 | -2,142 |  |  |
| C1ORF131 | chromosome 1 open reading frame 131 |  |  | 1,59E-11 | -1,708 |  |  |
| C1ORF151 | chromosome 1 open reading frame 151 |  |  | 2,24E-09 | -1,629 |  |  |
| C1ORF43 | chromosome 1 open reading frame 43 |  |  | 2,06E-08 | -1,711 |  |  |
| C1ORF43 | chromosome 1 open reading frame 43 |  |  |  |  | 2,89E-10 | -1,557 |
| C1ORF51 | chromosome 1 open reading frame 51 |  |  | 9,63E-09 | -1,746 |  |  |
| C1ORF53 | chromosome 1 open reading frame 53 |  |  | 1,31E-10 | -2,280 | 1,31E-10 | -1,792 |
| C1ORF59 | chromosome 1 open reading frame 59 |  |  | 1,47E-08 | -1,614 |  |  |
| C1ORF63 | chromosome 1 open reading frame 63 |  |  |  |  | 3,39E-08 | -1,756 |
| C1ORF77 | chromosome 1 open reading frame 77 |  |  | 2,30E-09 | -1,720 |  |  |
| C1ORF85 | chromosome 1 open reading frame 85 |  |  | 5,84E-09 | -1,959 |  |  |
| C1ORF85 | chromosome 1 open reading frame 85 |  |  | 1,79E-08 | -1,837 |  |  |
| C1QBP | complement component 1, q subcomponent binding protein |  |  | 3,94E-13 | -1,604 | 3,94E-13 | -1,456 |
| C1RL | complement component 1, r subcomponent-like |  |  | 3,10E-10 | -2,139 |  |  |
| C20ORF108 | chromosome 20 open reading frame 108 |  |  | 6,69E-08 | -1,589 |  |  |
| C20ORF111 | chromosome 20 open reading frame 111 |  |  | 3,43E-09 | -1,600 |  |  |
| C20ORF177 | chromosome 20 open reading frame 177 |  |  | 1,07E-12 | -1,708 |  |  |
| C20ORF199 | chromosome 20 open reading frame 199 |  |  | 1,69E-14 | -1,634 | 1,69E-14 | -1,451 |
| C20ORF20 | chromosome 20 open reading frame 20 |  |  | 9,13E-11 | -1,743 |  |  |
| C20ORF30 | chromosome 20 open reading frame 30 |  |  | 3,78E-13 | -1,551 | 3,78E-13 | -1,872 |
| C20ORF4 | chromosome 20 open reading frame 4 |  |  | 1,13E-10 | -1,805 |  |  |
| C20ORF7 | chromosome 20 open reading frame 7 |  |  | 4,97E-09 | -1,917 |  |  |
| C20ORF72 | chromosome 20 open reading frame 72 |  |  | 6,31E-08 | -1,489 |  |  |
| C21ORF45 | chromosome 21 open reading frame 45 |  |  | 4,00E-11 | -1,861 |  |  |
| C21ORF57 | chromosome 21 open reading frame 57 |  |  | 4,90E-09 | -2,068 |  |  |
| C21ORF66 | chromosome 21 open reading frame 66 |  |  | 7,03E-10 | -1,716 |  |  |
| C21ORF66 | chromosome 21 open reading frame 66 |  |  | 7,82E-10 | -1,642 |  |  |
| C22ORF28 | chromosome 22 open reading frame 28 |  |  | 7,21E-09 | -1,674 |  |  |
| C22ORF32 | chromosome 22 open reading frame 32 |  |  | 9,14E-10 | -1,814 |  |  |
| C22ORF32 | chromosome 22 open reading frame 32 |  |  | 9,51E-14 | -2,314 |  |  |
| C22ORF39 | chromosome 22 open reading frame 39 |  |  | 7,72E-09 | -1,959 |  |  |
| C2ORF12 | chromosome 2 open reading frame 12 |  |  |  |  | 1,69E-14 | 1,464 |
| C2ORF28 | chromosome 2 open reading frame 28 |  |  | 3,14E-11 | -1,791 |  |  |
| C2ORF30 | chromosome 2 open reading frame 30 |  |  | 3,58E-11 | -1,609 |  |  |
| C2ORF30 | chromosome 2 open reading frame 30 |  |  | 2,50E-09 | -1,594 |  |  |
| C2ORF79 | chromosome 2 open reading frame 79 |  |  | 8,23E-12 | -1,695 | 8,23E-12 | -1,623 |
| C3ORF10 | chromosome 3 open reading frame 10 |  |  | 5,27E-11 | -1,809 |  |  |
| C3ORF31 | chromosome 3 open reading frame 31 |  |  | 8,52E-12 | -2,665 | 8,52E-12 | -2,208 |
| C3ORF38 | chromosome 3 open reading frame 38 |  |  | 3,51E-11 | -1,619 |  |  |
| C4BPB | complement component 4 binding protein, beta |  |  | 3,90E-09 | -1,678 |  |  |
| C4ORF27 | chromosome 4 open reading frame 27 |  |  | 2,74E-08 | -1,594 |  |  |
| C4ORF30 | chromosome 4 open reading frame 30 |  |  | 1,31E-13 | -1,687 | 1,31E-13 | -1,556 |
| C4ORF41 | chromosome 4 open reading frame 41 |  |  |  |  | 1,07E-08 | -1,777 |
| C5ORF22 | chromosome 5 open reading frame 22 |  |  |  |  | 8,94E-08 | -1,917 |
| C5ORF22 | chromosome 5 open reading frame 22 |  |  | 2,17E-12 | -1,765 |  |  |
| C5ORF24 | chromosome 5 open reading frame 24 |  |  |  |  | 1,69E-14 | 2,383 |
| C5ORF25 | chromosome 5 open reading frame 25 |  |  | 2,61E-10 | -1,620 |  |  |
| C5ORF26 | chromosome 5 open reading frame 26 |  |  | 1,02E-08 | -1,539 |  |  |
| C5ORF35 | chromosome 5 open reading frame 35 |  |  | 1,20E-12 | -2,009 | 1,20E-12 | -1,691 |
| C6ORF108 | chromosome 6 open reading frame 108 |  |  | 1,80E-09 | -1,957 |  |  |
| C6ORF125 | chromosome 6 open reading frame 125 |  |  | 1,69E-14 | -2,045 |  |  |
| C6ORF130 | chromosome 6 open reading frame 130 |  |  | 6,67E-09 | -1,714 |  |  |
| C6ORF173 | chromosome 6 open reading frame 173 |  |  | 2,87E-08 | -1,548 |  |  |
| C6ORF211 | chromosome 6 open reading frame 211 |  |  | 2,09E-12 | -1,595 | 2,09E-12 | -1,392 |
| C6ORF64 | chromosome 6 open reading frame 64 |  |  | 4,73E-09 | -1,823 |  |  |
| C6ORF66 | chromosome 6 open reading frame 66 |  |  | 5,09E-09 | -1,592 |  |  |
| C7ORF11 | chromosome 7 open reading frame 11 |  |  | 1,33E-11 | -1,608 | 1,33E-11 | -1,631 |
| C7ORF23 | chromosome 7 open reading frame 23 |  |  | 1,11E-09 | -1,633 |  |  |
| C7ORF28A | chromosome 7 open reading frame 28A |  |  | 3,30E-10 | -1,669 |  |  |
| C7ORF28A | chromosome 7 open reading frame 28A |  |  | 6,11E-13 | -1,629 | 6,11E-13 | -1,887 |
| C7ORF30 | chromosome 7 open reading frame 30 |  |  | 1,17E-07 | -1,519 |  |  |
| C7ORF44 | chromosome 7 open reading frame 44 |  |  | 5,66E-09 | -1,616 |  |  |
| C7ORF44 | chromosome 7 open reading frame 44 |  |  | 3,18E-10 | -2,024 |  |  |
| C7ORF50 | chromosome 7 open reading frame 50 | 8,63E-12 | -1,425 | 8,63E-12 | -2,192 |  |  |
| C7ORF59 | chromosome 7 open reading frame 59 |  |  | 1,69E-08 | -1,736 |  |  |
| C8ORF33 | chromosome 8 open reading frame 33 |  |  | 1,29E-09 | -1,869 |  |  |
| C8ORF38 | chromosome 8 open reading frame 38 |  |  | 9,14E-08 | -1,580 |  |  |
| C8ORF59 | chromosome 8 open reading frame 59 |  |  | 8,99E-12 | -1,653 |  |  |
| C9ORF140 | chromosome 9 open reading frame 140 |  |  | 2,71E-08 | -1,721 |  |  |
| C9ORF16 | chromosome 9 open reading frame 16 |  |  | 9,61E-08 | -1,750 |  |  |
| C9ORF30 | chromosome 9 open reading frame 30 |  |  | 9,25E-11 | -1,974 |  |  |
| C9ORF5 | chromosome 9 open reading frame 5 |  |  | 1,78E-09 | -1,524 | 1,78E-09 | -1,560 |
| C9ORF5 | chromosome 9 open reading frame 5 |  |  | 2,29E-11 | -1,671 | 2,29E-11 | -1,398 |
| C9ORF5 | chromosome 9 open reading frame 5 |  |  | 5,89E-10 | -1,604 |  |  |
| C9ORF80 | chromosome 9 open reading frame 80 |  |  | 1,80E-08 | -1,541 |  |  |
| C9ORF86 | chromosome 9 open reading frame 86 |  |  |  |  | 1,69E-14 | 1,751 |
| CAB39 | calcium binding protein 39 |  |  | 1,48E-10 | -1,674 |  |  |
| CABC1 | chaperone, ABC1 activity of bc1 complex homolog (S. pombe) |  |  | 1,10E-08 | -1,873 |  |  |
| CABIN1 | calcineurin binding protein 1 |  |  | 5,80E-11 | -3,110 |  |  |
| CALD1 | caldesmon 1 |  |  | 1,69E-14 | -1,757 | 1,69E-14 | -1,704 |
| CALM3 | calmodulin 3 (phosphorylase kinase, delta) |  |  | 1,69E-09 | -2,013 |  |  |
| CALM3 | calmodulin 3 (phosphorylase kinase, delta) |  |  | 7,46E-08 | -1,570 |  |  |
| CALML4 | calmodulin-like 4 |  |  | 4,52E-09 | -1,681 |  |  |
| CALR | calreticulin |  |  | 1,84E-08 | -1,628 |  |  |
| CAMK2G | calcium/calmodulin-dependent protein kinase II gamma |  |  | 1,95E-11 | -1,729 | 1,95E-11 | -1,521 |
| CAMK2N1 | calcium/calmodulin-dependent protein kinase II inhibitor 1 |  |  | 1,69E-14 | -2,217 | 1,69E-14 | -1,732 |
| CAMTA1 (includes EG:23261) | calmodulin binding transcription activator 1 |  |  | 1,32E-08 | -1,753 |  |  |
| CAND1 | cullin-associated and neddylation-dissociated 1 |  |  | 1,77E-09 | -1,651 |  |  |
| CANT1 | calcium activated nucleotidase 1 |  |  | 6,63E-11 | -1,820 |  |  |
| CAP1 | CAP, adenylate cyclase-associated protein 1 (yeast) |  |  | 2,06E-13 | -2,091 |  |  |
| CAPN1 | calpain 1, (mu/I) large subunit |  |  | 2,38E-09 | -2,686 |  |  |
| CAPNS1 | calpain, small subunit 1 |  |  | 1,85E-13 | -1,964 |  |  |
| CAPRIN1 | cell cycle associated protein 1 |  |  | 1,43E-13 | -1,665 | 1,43E-13 | -1,851 |
| CAPRIN1 | cell cycle associated protein 1 |  |  | 1,59E-09 | -1,525 |  |  |
| CAPZB | capping protein (actin filament) muscle Z-line, beta |  |  | 1,22E-11 | -1,762 |  |  |
| CAPZB | capping protein (actin filament) muscle Z-line, beta |  |  | 5,87E-12 | -1,938 |  |  |
| CAPZB | capping protein (actin filament) muscle Z-line, beta |  |  | 1,69E-14 | -1,678 |  |  |
| CASC4 | cancer susceptibility candidate 4 |  |  | 6,43E-09 | -1,532 |  |  |
| CASC5 | cancer susceptibility candidate 5 |  |  |  |  | 2,08E-11 | 2,300 |
| CASP2 | caspase 2, apoptosis-related cysteine peptidase |  |  |  |  | 3,43E-11 | 2,752 |
| CASP8AP2 | caspase 8 associated protein 2 |  |  | 1,07E-13 | -1,879 |  |  |
| CAST | calpastatin |  |  | 3,39E-09 | -1,447 |  |  |
| CAST | calpastatin |  |  | 1,21E-12 | -1,696 |  |  |
| CBARA1 | calcium binding atopy-related autoantigen 1 |  |  | 9,89E-08 | -1,774 |  |  |
| CBL | Cas-Br-M (murine) ecotropic retroviral transforming sequence |  |  |  |  | 1,69E-14 | 2,305 |
| CBX1 | chromobox homolog 1 (HP1 beta homolog Drosophila ) |  |  | 1,34E-11 | -1,561 | 1,34E-11 | -1,396 |
| CBX3 | chromobox homolog 3 (HP1 gamma homolog, Drosophila) | 1,53E-12 | -1,552 | 1,53E-12 | -1,914 |  |  |
| CBX4 | chromobox homolog 4 (Pc class homolog, Drosophila) |  |  | 6,47E-11 | -1,744 |  |  |
| CBX5 | chromobox homolog 5 (HP1 alpha homolog, Drosophila) |  |  |  |  | 1,34E-07 | 1,404 |
| CCDC109A | coiled-coil domain containing 109A |  |  | 1,83E-10 | -1,873 |  |  |
| CCDC34 | coiled-coil domain containing 34 |  |  | 3,00E-13 | -1,912 |  |  |
| CCDC5 | coiled-coil domain containing 5 (spindle associated) |  |  | 1,63E-12 | -1,779 |  |  |
| CCDC50 | coiled-coil domain containing 50 |  |  | 2,69E-11 | -1,699 | 2,69E-11 | -1,477 |
| CCDC52 | coiled-coil domain containing 52 |  |  | 1,66E-09 | -1,807 |  |  |
| CCDC56 | coiled-coil domain containing 56 |  |  | 3,57E-11 | -1,567 |  |  |
| CCDC90B | coiled-coil domain containing 90B |  |  | 1,63E-07 | -1,494 |  |  |
| CCDC99 | coiled-coil domain containing 99 |  |  | 5,44E-11 | -1,603 |  |  |
| CCNA2 | cyclin A2 |  |  | 1,69E-14 | -1,577 | 1,69E-14 | -1,719 |
| CCNB1 | cyclin B1 |  |  | 8,69E-11 | -1,534 |  |  |
| CCNC | cyclin C |  |  | 4,57E-09 | -1,446 |  |  |
| CCND1 | cyclin D1 |  |  |  |  | 1,69E-14 | 1,905 |
| CCND1 | cyclin D1 |  |  |  |  | 1,69E-14 | 1,556 |
| CCNE1 | cyclin E1 |  |  | 2,83E-11 | -2,107 |  |  |
| CCNF | cyclin F |  |  | 6,05E-10 | -2,150 |  |  |
| CCNG1 | cyclin G1 |  |  | 5,46E-10 | -1,516 |  |  |
| CCNG2 | cyclin G2 |  |  | 6,41E-09 | -1,502 |  |  |
| CCNG2 | cyclin G2 |  |  | 1,69E-14 | -1,659 | 1,69E-14 | -2,141 |
| CCNG2 | cyclin G2 |  |  | 1,69E-14 | -1,882 | 1,69E-14 | -3,211 |
| CCNH | cyclin H |  |  | 6,08E-12 | -1,911 |  |  |
| CCNI | cyclin I |  |  | 3,49E-13 | -1,796 |  |  |
| CCNJ | cyclin J |  |  | 5,49E-09 | -1,969 |  |  |
| CCNK | cyclin K |  |  | 5,77E-14 | -1,777 | 5,77E-14 | -1,375 |
| CCNY | cyclin Y |  |  | 1,08E-10 | -1,738 |  |  |
| CCPG1 | cell cycle progression 1 |  |  | 1,69E-09 | -2,111 |  |  |
| CD164 | CD164 molecule, sialomucin |  |  | 1,76E-11 | -1,445 | 1,76E-11 | -1,780 |
| CD164 | CD164 molecule, sialomucin |  |  | 3,50E-11 | -1,403 | 3,50E-11 | -1,484 |
| CD2AP | CD2-associated protein |  |  | 2,32E-12 | -1,835 |  |  |
| CD46 | CD46 molecule, complement regulatory protein |  |  | 4,45E-11 | -1,586 | 4,45E-11 | -1,924 |
| CD46 | CD46 molecule, complement regulatory protein |  |  | 3,14E-14 | -1,724 | 3,14E-14 | -2,004 |
| CD55 | CD55 molecule, decay accelerating factor for complement (Cromer blood group) |  |  | 1,03E-10 | -1,704 |  |  |
| CD59 | CD59 molecule, complement regulatory protein |  |  | 3,70E-08 | -1,561 |  |  |
| CD99 (includes EG:4267) | CD99 molecule |  |  | 7,55E-10 | -1,602 |  |  |
| CDC16 | cell division cycle 16 homolog (S. cerevisiae) |  |  | 7,24E-12 | -1,764 |  |  |
| CDC2 | cell division cycle 2, G1 to S and G2 to M |  |  | 2,08E-12 | -1,447 | 2,08E-12 | -1,505 |
| CDC20 | cell division cycle 20 homolog (S. cerevisiae) |  |  | 9,44E-08 | -1,835 |  |  |
| CDC26 | cell division cycle 26 homolog (S. cerevisiae) |  |  | 1,71E-08 | -1,567 |  |  |
| CDC27 | cell division cycle 27 homolog (S. cerevisiae) |  |  | 5,24E-12 | -1,723 |  |  |
| CDC2L2 | cell division cycle 2-like 2 (PITSLRE proteins) |  |  | 9,49E-10 | -1,695 |  |  |
| CDC34 (includes EG:997) | cell division cycle 34 homolog (S. cerevisiae) |  |  | 6,76E-11 | -1,961 |  |  |
| CDC40 | cell division cycle 40 homolog (S. cerevisiae) |  |  | 4,15E-11 | -1,705 | 4,15E-11 | -1,408 |
| CDC42 | cell division cycle 42 (GTP binding protein, 25kDa) |  |  | 1,69E-14 | -2,007 | 1,69E-14 | -2,270 |
| CDC42BPA | CDC42 binding protein kinase alpha (DMPK-like) |  |  |  |  | 1,69E-14 | 2,136 |
| CDC42EP3 | CDC42 effector protein (Rho GTPase binding) 3 |  |  | 1,51E-12 | -1,720 |  |  |
| CDC45L | CDC45 cell division cycle 45-like (S. cerevisiae) |  |  | 1,13E-09 | -2,183 |  |  |
| CDCA4 | cell division cycle associated 4 |  |  | 1,69E-14 | -2,294 |  |  |
| CDCA8 | cell division cycle associated 8 |  |  | 1,69E-14 | -2,113 | 1,69E-14 | -1,695 |
| CDH1 | cadherin 1, type 1, E-cadherin (epithelial) | 5,77E-14 | -2,223 | 5,77E-14 | -1,641 | 5,77E-14 | -1,688 |
| CDH1 | cadherin 1, type 1, E-cadherin (epithelial) |  |  | 3,12E-09 | -1,551 |  |  |
| CDK2 | cyclin-dependent kinase 2 |  |  | 2,44E-13 | -1,861 |  |  |
| CDK2 | cyclin-dependent kinase 2 | 6,53E-12 | -1,840 | 6,53E-12 | -1,918 | 6,53E-12 | -1,835 |
| CDK4 | cyclin-dependent kinase 4 |  |  | 2,00E-10 | -1,612 |  |  |
| CDK5RAP3 (includes EG:80279) | CDK5 regulatory subunit associated protein 3 |  |  | 3,20E-08 | -1,681 |  |  |
| CDK7 | cyclin-dependent kinase 7 |  |  | 5,84E-09 | -1,488 |  |  |
| CDK8 | cyclin-dependent kinase 8 |  |  | 4,61E-11 | -1,732 | 4,61E-11 | -1,703 |
| CDKN1B | cyclin-dependent kinase inhibitor 1B (p27, Kip1) |  |  | 4,79E-12 | -1,444 | 4,79E-12 | -1,414 |
| CDKN2AIPNL | CDKN2A interacting protein N-terminal like |  |  | 1,19E-08 | -1,844 |  |  |
| CDKN3 | cyclin-dependent kinase inhibitor 3 |  |  | 1,69E-14 | -2,033 | 1,69E-14 | -1,875 |
| CDKN3 | cyclin-dependent kinase inhibitor 3 |  |  | 1,69E-14 | -1,999 | 1,69E-14 | -1,531 |
| CDS2 | CDP-diacylglycerol synthase (phosphatidate cytidylyltransferase) 2 | 1,69E-14 | -1,630 | 1,69E-14 | -2,272 |  |  |
| CDS2 | CDP-diacylglycerol synthase (phosphatidate cytidylyltransferase) 2 |  |  | 6,28E-09 | -1,936 |  |  |
| CDV3 | CDV3 homolog (mouse) | 1,73E-13 | -1,834 | 1,73E-13 | -2,309 | 1,73E-13 | -1,924 |
| CDV3 | CDV3 homolog (mouse) | 1,36E-11 | 1,556 |  |  |  |  |
| CENPA | centromere protein A |  |  | 3,96E-09 | -1,872 |  |  |
| CENPJ | centromere protein J |  |  | 5,64E-09 | -1,737 |  |  |
| CENPN | centromere protein N |  |  | 8,01E-12 | -1,815 |  |  |
| CENPO | centromere protein O |  |  | 4,87E-09 | -1,742 |  |  |
| CENPQ | centromere protein Q |  |  | 1,69E-14 | -1,707 | 1,69E-14 | -2,001 |
| CEP290 | centrosomal protein 290kDa |  |  |  |  | 1,56E-08 | 1,737 |
| CEP57 | centrosomal protein 57kDa |  |  | 2,09E-11 | -1,616 |  |  |
| CEP57 | centrosomal protein 57kDa |  |  | 9,92E-13 | -1,781 | 9,92E-13 | -1,402 |
| CEP57 | centrosomal protein 57kDa |  |  | 6,79E-11 | -1,668 | 6,79E-11 | -1,503 |
| CEP70 | centrosomal protein 70kDa |  |  | 2,66E-08 | -1,709 |  |  |
| CEP76 | centrosomal protein 76kDa |  |  | 4,47E-10 | -1,554 |  |  |
| CES2 (includes EG:8824) | carboxylesterase 2 (intestine, liver) |  |  | 2,24E-08 | -2,187 |  |  |
| CETN2 | centrin, EF-hand protein, 2 |  |  | 9,25E-11 | -1,718 |  |  |
| CETN3 | centrin, EF-hand protein, 3 (CDC31 homolog, yeast) |  |  | 2,35E-10 | -1,537 |  |  |
| CFL2 | cofilin 2 (muscle) |  |  | 1,69E-14 | -2,034 |  |  |
| CFL2 | cofilin 2 (muscle) |  |  | 2,04E-12 | -1,730 | 2,04E-12 | -1,486 |
| CFLAR | CASP8 and FADD-like apoptosis regulator |  |  |  |  | 1,06E-08 | 1,470 |
| CGN | cingulin |  |  | 5,19E-10 | -1,860 |  |  |
| CHCHD1 | coiled-coil-helix-coiled-coil-helix domain containing 1 |  |  | 1,44E-12 | -1,730 |  |  |
| CHCHD2 | coiled-coil-helix-coiled-coil-helix domain containing 2 |  |  | 6,30E-08 | -1,475 |  |  |
| CHCHD5 | coiled-coil-helix-coiled-coil-helix domain containing 5 |  |  | 1,75E-08 | -1,826 |  |  |
| CHD2 | chromodomain helicase DNA binding protein 2 |  |  | 2,16E-13 | -1,725 |  |  |
| CHD4 | chromodomain helicase DNA binding protein 4 |  |  |  |  | 1,69E-14 | 2,551 |
| CHD4 | chromodomain helicase DNA binding protein 4 |  |  |  |  | 5,17E-10 | 1,768 |
| CHD9 (includes EG:80205) | chromodomain helicase DNA binding protein 9 |  |  | 7,59E-09 | -1,628 |  |  |
| CHEK1 | CHK1 checkpoint homolog (S. pombe) |  |  | 5,26E-09 | -1,638 |  |  |
| CHEK1 | CHK1 checkpoint homolog (S. pombe) |  |  | 4,86E-10 | -1,529 |  |  |
| CHERP | calcium homeostasis endoplasmic reticulum protein |  |  | 4,31E-08 | -1,527 |  |  |
| CHIC2 | cysteine-rich hydrophobic domain 2 |  |  | 1,75E-09 | -1,781 |  |  |
| CHKA | choline kinase alpha |  |  | 3,04E-09 | -1,773 |  |  |
| CHM | choroideremia (Rab escort protein 1) |  |  | 5,98E-11 | -1,733 |  |  |
| CHML | choroideremia-like (Rab escort protein 2) |  |  | 1,04E-10 | -1,648 |  |  |
| CHMP2A | chromatin modifying protein 2A |  |  | 1,71E-10 | -1,724 |  |  |
| CHMP2B | chromatin modifying protein 2B |  |  | 2,97E-09 | -1,601 |  |  |
| CHMP4A | chromatin modifying protein 4A |  |  | 2,36E-11 | -1,772 |  |  |
| CHMP4B | chromatin modifying protein 4B |  |  | 1,67E-10 | -1,686 |  |  |
| CHTF18 | CTF18, chromosome transmission fidelity factor 18 homolog (S. cerevisiae) |  |  | 5,58E-10 | -1,999 |  |  |
| CHUK | conserved helix-loop-helix ubiquitous kinase |  |  | 5,92E-09 | -1,656 |  |  |
| CIAO1 | cytosolic iron-sulfur protein assembly 1 homolog (S. cerevisiae) |  |  | 5,15E-10 | -1,919 |  |  |
| CIAPIN1 | cytokine induced apoptosis inhibitor 1 |  |  | 2,16E-10 | -1,684 |  |  |
| CIAPIN1 | cytokine induced apoptosis inhibitor 1 |  |  | 1,13E-09 | -1,664 |  |  |
| CIB1 | calcium and integrin binding 1 (calmyrin) |  |  | 1,23E-08 | -1,635 |  |  |
| CIRBP | cold inducible RNA binding protein |  |  | 1,69E-09 | -1,737 |  |  |
| CIRBP | cold inducible RNA binding protein |  |  | 2,06E-10 | -1,786 |  |  |
| CIRH1A | cirrhosis, autosomal recessive 1A (cirhin) |  |  | 4,49E-14 | -1,837 |  |  |
| CIRH1A | cirrhosis, autosomal recessive 1A (cirhin) |  |  | 8,23E-08 | -1,523 |  |  |
| CKAP2 | cytoskeleton associated protein 2 |  |  | 1,14E-08 | -1,506 |  |  |
| CKAP5 | cytoskeleton associated protein 5 |  |  | 1,26E-09 | -1,657 |  |  |
| CKLF | chemokine-like factor |  |  | 6,81E-11 | -2,022 |  |  |
| CKS1B | CDC28 protein kinase regulatory subunit 1B |  |  | 1,19E-09 | -1,538 |  |  |
| CLASP1 | cytoplasmic linker associated protein 1 | 3,18E-10 | -3,009 | 3,18E-10 | -1,965 | 3,18E-10 | -2,401 |
| CLCN5 | chloride channel 5 |  |  |  |  | 3,70E-11 | 1,473 |
| CLCN5 | chloride channel 5 |  |  | 3,45E-10 | -1,660 |  |  |
| CLCN5 | chloride channel 5 |  |  |  |  | 3,11E-12 | 2,151 |
| CLDND1 | claudin domain containing 1 |  |  | 3,24E-09 | -1,504 |  |  |
| CLIC4 | chloride intracellular channel 4 |  |  | 1,85E-13 | -1,704 | 1,85E-13 | -2,470 |
| CLIP1 | CAP-GLY domain containing linker protein 1 |  |  |  |  | 7,07E-09 | 2,128 |
| CLPTM1 | cleft lip and palate associated transmembrane protein 1 |  |  | 5,76E-12 | -2,114 |  |  |
| CLTA | clathrin, light chain (Lca) |  |  | 4,44E-08 | -1,524 |  |  |
| CLTB | clathrin, light chain (Lcb) |  |  | 2,49E-11 | -1,956 |  |  |
| CLTB | clathrin, light chain (Lcb) |  |  | 1,20E-08 | -1,776 |  |  |
| CLU | clusterin |  |  | 5,26E-11 | -1,710 |  |  |
| CMBL | carboxymethylenebutenolidase homolog (Pseudomonas) |  |  | 1,34E-12 | -1,886 | 1,34E-12 | -1,683 |
| CMPK1 | cytidine monophosphate (UMP-CMP) kinase 1, cytosolic |  |  | 9,97E-10 | -1,808 |  |  |
| CMTM4 | CKLF-like MARVEL transmembrane domain containing 4 |  |  | 2,03E-11 | -1,742 |  |  |
| CMTM6 | CKLF-like MARVEL transmembrane domain containing 6 |  |  | 1,98E-08 | -1,611 |  |  |
| CNIH | cornichon homolog (Drosophila) |  |  | 1,63E-13 | -1,579 | 1,63E-13 | -1,412 |
| CNIH4 | cornichon homolog 4 (Drosophila) |  |  | 7,90E-10 | -1,494 |  |  |
| CNOT1 | CCR4-NOT transcription complex, subunit 1 |  |  | 2,76E-09 | -2,102 |  |  |
| CNOT2 | CCR4-NOT transcription complex, subunit 2 |  |  | 4,96E-09 | -1,620 |  |  |
| CNOT7 | CCR4-NOT transcription complex, subunit 7 |  |  | 1,05E-08 | -1,885 |  |  |
| CNOT7 | CCR4-NOT transcription complex, subunit 7 |  |  | 1,53E-08 | -1,538 |  |  |
| CNP | 2',3'-cyclic nucleotide 3' phosphodiesterase |  |  | 1,33E-07 | -1,869 |  |  |
| CNPY2 | canopy 2 homolog (zebrafish) |  |  | 3,92E-12 | -1,645 |  |  |
| CNPY3 | canopy 3 homolog (zebrafish) |  |  | 1,41E-08 | -2,002 |  |  |
| COASY | Coenzyme A synthase |  |  | 2,47E-09 | -1,917 |  |  |
| COBRA1 | cofactor of BRCA1 |  |  | 1,01E-09 | -1,774 |  |  |
| COCH | coagulation factor C homolog, cochlin (Limulus polyphemus) |  |  | 1,69E-14 | -1,419 | 1,69E-14 | -2,155 |
| COCH | coagulation factor C homolog, cochlin (Limulus polyphemus) |  |  | 2,71E-13 | -1,864 | 2,71E-13 | -1,498 |
| COCH | coagulation factor C homolog, cochlin (Limulus polyphemus) |  |  | 1,33E-11 | -1,649 |  |  |
| COG7 | component of oligomeric golgi complex 7 |  |  | 2,20E-07 | -2,339 |  |  |
| COL12A1 | collagen, type XII, alpha 1 |  |  | 6,28E-09 | -1,618 |  |  |
| COL1A1 | collagen, type I, alpha 1 |  |  | 6,52E-11 | -1,979 |  |  |
| COL4A3BP | collagen, type IV, alpha 3 (Goodpasture antigen) binding protein |  |  |  |  | 1,65E-09 | 1,663 |
| COL5A2 | collagen, type V, alpha 2 |  |  | 1,08E-08 | -1,538 |  |  |
| COMMD10 | COMM domain containing 10 |  |  | 9,74E-10 | -1,517 |  |  |
| COMMD2 | COMM domain containing 2 |  |  | 4,29E-09 | -1,679 |  |  |
| COMMD4 | COMM domain containing 4 |  |  | 4,72E-13 | -1,992 |  |  |
| COMMD5 | COMM domain containing 5 |  |  | 1,22E-09 | -2,416 |  |  |
| COMMD7 | COMM domain containing 7 |  |  | 7,63E-09 | -1,603 |  |  |
| COMT | catechol-O-methyltransferase |  |  | 1,07E-11 | -1,820 | 1,07E-11 | -1,662 |
| COMT | catechol-O-methyltransferase |  |  | 5,30E-11 | -1,780 | 5,30E-11 | -1,499 |
| COPA | coatomer protein complex, subunit alpha | 3,50E-11 | -2,724 | 3,50E-11 | -2,193 | 3,50E-11 | -2,229 |
| COPB1 | coatomer protein complex, subunit beta 1 |  |  |  |  | 4,19E-09 | -1,429 |
| COPS4 | COP9 constitutive photomorphogenic homolog subunit 4 (Arabidopsis) |  |  | 1,80E-09 | -1,563 |  |  |
| COPS5 | COP9 constitutive photomorphogenic homolog subunit 5 (Arabidopsis) |  |  | 4,62E-09 | -1,576 |  |  |
| COPS6 | COP9 constitutive photomorphogenic homolog subunit 6 (Arabidopsis) |  |  | 8,13E-09 | -1,693 |  |  |
| COPS6 | COP9 constitutive photomorphogenic homolog subunit 6 (Arabidopsis) |  |  | 3,08E-11 | -1,938 |  |  |
| COPS7A | COP9 constitutive photomorphogenic homolog subunit 7A (Arabidopsis) |  |  | 1,24E-07 | -1,724 |  |  |
| COPS8 | COP9 constitutive photomorphogenic homolog subunit 8 (Arabidopsis) |  |  | 1,80E-10 | -1,653 | 1,80E-10 | -1,692 |
| COPZ1 | coatomer protein complex, subunit zeta 1 |  |  | 7,62E-12 | -1,765 |  |  |
| COPZ1 | coatomer protein complex, subunit zeta 1 |  |  | 4,40E-11 | -1,639 | 4,40E-11 | -1,438 |
| COQ2 | coenzyme Q2 homolog, prenyltransferase (yeast) |  |  | 1,95E-13 | -2,060 |  |  |
| COQ3 | coenzyme Q3 homolog, methyltransferase (S. cerevisiae) |  |  | 5,68E-08 | -1,799 |  |  |
| COQ4 | coenzyme Q4 homolog (S. cerevisiae) |  |  | 1,33E-08 | -1,888 |  |  |
| CORO1C | coronin, actin binding protein, 1C |  |  | 4,35E-08 | -1,453 |  |  |
| COTL1 | coactosin-like 1 (Dictyostelium) |  |  | 4,68E-11 | -1,634 |  |  |
| COTL1 | coactosin-like 1 (Dictyostelium) |  |  | 3,71E-12 | -1,605 |  |  |
| COX1 | cytochrome c oxidase I | 9,06E-08 | -1,271 | 9,06E-08 | -1,404 |  |  |
| COX15 | COX15 homolog, cytochrome c oxidase assembly protein (yeast) |  |  | 7,43E-11 | -1,808 |  |  |
| COX4NB | COX4 neighbor |  |  | 2,16E-13 | -2,373 |  |  |
| COX4NB | COX4 neighbor |  |  | 7,41E-09 | -1,618 |  |  |
| COX5A | cytochrome c oxidase subunit Va |  |  | 3,95E-09 | -1,498 |  |  |
| COX5B | cytochrome c oxidase subunit Vb |  |  | 4,67E-09 | -1,702 |  |  |
| COX7A2L | cytochrome c oxidase subunit VIIa polypeptide 2 like |  |  | 4,53E-08 | -1,550 |  |  |
| COX7C (includes EG:1350) | cytochrome c oxidase subunit VIIc |  |  | 7,34E-08 | -1,642 |  |  |
| CPD | carboxypeptidase D |  |  | 1,70E-08 | -1,580 |  |  |
| CPNE1 | copine I |  |  | 2,40E-08 | -1,595 |  |  |
| CPNE1 | copine I |  |  | 9,97E-09 | -1,501 |  |  |
| CPSF2 | cleavage and polyadenylation specific factor 2, 100kDa |  |  | 9,26E-09 | -2,006 |  |  |
| CPSF3 | cleavage and polyadenylation specific factor 3, 73kDa |  |  | 1,06E-09 | -1,606 |  |  |
| CPSF3L | cleavage and polyadenylation specific factor 3-like |  |  | 8,51E-08 | -1,923 |  |  |
| CPSF4 | cleavage and polyadenylation specific factor 4, 30kDa |  |  | 1,92E-09 | -1,821 |  |  |
| CPSF6 | cleavage and polyadenylation specific factor 6, 68kDa |  |  | 1,69E-14 | -1,937 | 1,69E-14 | -1,400 |
| CRBN | cereblon |  |  | 5,31E-10 | -1,701 |  |  |
| CRELD2 | cysteine-rich with EGF-like domains 2 |  |  | 4,60E-11 | -1,924 |  |  |
| CRIM1 | cysteine rich transmembrane BMP regulator 1 (chordin-like) |  |  | 4,49E-10 | -1,472 |  |  |
| CRIM1 | cysteine rich transmembrane BMP regulator 1 (chordin-like) |  |  |  |  | 2,15E-08 | -1,427 |
| CRK | v-crk sarcoma virus CT10 oncogene homolog (avian) |  |  | 6,58E-09 | -1,462 |  |  |
| CRKL | v-crk sarcoma virus CT10 oncogene homolog (avian)-like |  |  | 3,51E-10 | -1,824 |  |  |
| CRLS1 | cardiolipin synthase 1 |  |  | 1,05E-08 | -1,715 |  |  |
| CROP | cisplatin resistance-associated overexpressed protein |  |  |  |  | 1,64E-09 | 1,490 |
| CRTAP | cartilage associated protein | 1,69E-14 | -2,172 | 1,69E-14 | -1,684 | 1,69E-14 | -1,787 |
| CRTAP | cartilage associated protein |  |  | 2,75E-08 | -1,600 |  |  |
| CS | citrate synthase |  |  | 1,22E-08 | -1,576 |  |  |
| CSDE1 | cold shock domain containing E1, RNA-binding |  |  | 9,12E-11 | -1,702 |  |  |
| CSNK1D | casein kinase 1, delta |  |  | 3,92E-08 | -1,768 |  |  |
| CSNK1D | casein kinase 1, delta |  |  | 2,66E-11 | -2,014 |  |  |
| CSNK2A2 | casein kinase 2, alpha prime polypeptide |  |  | 8,23E-08 | -1,742 |  |  |
| CSNK2A2 | casein kinase 2, alpha prime polypeptide |  |  | 2,51E-08 | -1,503 |  |  |
| CSPP1 | centrosome and spindle pole associated protein 1 |  |  | 6,41E-10 | -1,627 |  |  |
| CSRP1 | cysteine and glycine-rich protein 1 |  |  | 5,76E-12 | -1,894 |  |  |
| CSRP2BP | CSRP2 binding protein | 1,09E-11 | -1,458 | 1,09E-11 | -2,107 |  |  |
| CST2 | cystatin SA |  |  | 9,71E-13 | -1,811 |  |  |
| CSTF2 (includes EG:1478) | cleavage stimulation factor, 3' pre-RNA, subunit 2, 64kDa |  |  | 6,64E-13 | -1,970 | 6,64E-13 | -1,610 |
| CSTF3 | cleavage stimulation factor, 3' pre-RNA, subunit 3, 77kDa |  |  | 1,38E-10 | -1,666 |  |  |
| CTBP1 | C-terminal binding protein 1 |  |  | 2,36E-13 | -1,838 |  |  |
| CTBP1 | C-terminal binding protein 1 |  |  | 8,36E-12 | -1,872 |  |  |
| CTBP1 | C-terminal binding protein 1 |  |  | 4,49E-13 | -2,070 |  |  |
| CTDSPL | CTD (carboxy-terminal domain, RNA polymerase II, polypeptide A) small phosphatase-like |  |  | 1,10E-08 | -1,688 |  |  |
| CTDSPL2 | CTD (carboxy-terminal domain, RNA polymerase II, polypeptide A) small phosphatase like 2 |  |  |  |  | 2,61E-11 | -2,293 |
| CTDSPL2 | CTD (carboxy-terminal domain, RNA polymerase II, polypeptide A) small phosphatase like 2 |  |  | 1,97E-07 | -1,651 |  |  |
| CTF8 | chromosome transmission fidelity factor 8 homolog (S. cerevisiae) |  |  | 3,04E-08 | -1,855 |  |  |
| CTNNA1 | catenin (cadherin-associated protein), alpha 1, 102kDa |  |  | 1,35E-12 | -2,102 | 1,35E-12 | -1,852 |
| CTNNB1 | catenin (cadherin-associated protein), beta 1, 88kDa |  |  | 1,69E-14 | -2,085 |  |  |
| CTR9 | Ctr9, Paf1/RNA polymerase II complex component, homolog (S. cerevisiae) |  |  | 4,29E-13 | -1,628 |  |  |
| CTSA | cathepsin A |  |  | 1,19E-11 | -1,826 |  |  |
| CTSB | cathepsin B |  |  | 1,36E-11 | -1,757 |  |  |
| CTSC | cathepsin C |  |  | 1,69E-14 | -1,692 | 1,69E-14 | -1,827 |
| CTSH | cathepsin H |  |  | 6,47E-10 | -1,701 |  |  |
| CTSL1 | cathepsin L1 |  |  | 7,86E-08 | -1,758 |  |  |
| CTSZ (includes EG:1522) | cathepsin Z | 1,69E-14 | 1,470 |  |  |  |  |
| CTTN | cortactin |  |  | 2,75E-10 | -1,641 |  |  |
| CUEDC2 | CUE domain containing 2 |  |  | 1,86E-10 | -1,867 |  |  |
| CUL1 | cullin 1 |  |  | 2,51E-10 | -1,808 |  |  |
| CXADR | coxsackie virus and adenovirus receptor |  |  |  |  | 4,05E-08 | -1,817 |
| CXCL16 | chemokine (C-X-C motif) ligand 16 |  |  | 5,94E-08 | -1,612 |  |  |
| CXORF26 | chromosome X open reading frame 26 |  |  | 3,38E-08 | -1,410 |  |  |
| CXORF39 | chromosome X open reading frame 39 |  |  | 3,55E-09 | -1,690 |  |  |
| CXXC5 | CXXC finger 5 |  |  | 1,40E-09 | -1,838 |  |  |
| CYB561 | cytochrome b-561 |  |  | 1,69E-14 | -2,441 |  |  |
| CYB5A | cytochrome b5 type A (microsomal) |  |  | 1,18E-11 | -1,634 | 1,18E-11 | -1,572 |
| CYB5A | cytochrome b5 type A (microsomal) |  |  | 6,25E-11 | -1,552 | 6,25E-11 | -1,532 |
| CYB5A | cytochrome b5 type A (microsomal) |  |  | 1,69E-14 | -1,812 | 1,69E-14 | -1,768 |
| CYB5R1 | cytochrome b5 reductase 1 |  |  | 1,03E-08 | -1,982 |  |  |
| CYB5R3 | cytochrome b5 reductase 3 | 7,81E-11 | -1,500 | 7,81E-11 | -2,031 | 7,81E-11 | -1,571 |
| CYBA | cytochrome b-245, alpha polypeptide |  |  | 1,26E-09 | -1,825 |  |  |
| CYC1 | cytochrome c-1 |  |  | 1,75E-09 | -1,717 |  |  |
| CYP1A1 | cytochrome P450, family 1, subfamily A, polypeptide 1 |  |  | 1,69E-14 | 5,329 | 1,69E-14 | 7,487 |
| CYP2R1 | cytochrome P450, family 2, subfamily R, polypeptide 1 |  |  | 1,96E-11 | -1,815 |  |  |
| CYP51A1 | cytochrome P450, family 51, subfamily A, polypeptide 1 |  |  | 1,78E-08 | -1,559 |  |  |
| CYP51A1 | cytochrome P450, family 51, subfamily A, polypeptide 1 |  |  |  |  | 4,90E-08 | -1,416 |
| DAB2 | disabled homolog 2, mitogen-responsive phosphoprotein (Drosophila) |  |  | 3,69E-13 | -1,724 | 3,69E-13 | -1,393 |
| DAB2 | disabled homolog 2, mitogen-responsive phosphoprotein (Drosophila) |  |  | 9,97E-10 | -1,725 | 9,97E-10 | -1,688 |
| DAB2 | disabled homolog 2, mitogen-responsive phosphoprotein (Drosophila) |  |  | 7,15E-11 | -1,580 | 7,15E-11 | -1,533 |
| DAB2 | disabled homolog 2, mitogen-responsive phosphoprotein (Drosophila) |  |  |  |  | 1,71E-09 | -1,521 |
| DAG1 | dystroglycan 1 (dystrophin-associated glycoprotein 1) |  |  | 2,29E-12 | -1,958 |  |  |
| DAPK1 | death-associated protein kinase 1 |  |  | 4,20E-09 | -1,804 |  |  |
| DARS | aspartyl-tRNA synthetase |  |  | 3,33E-11 | -1,635 |  |  |
| DAZAP2 (includes EG:9802) | DAZ associated protein 2 |  |  | 1,45E-10 | -1,570 | 1,45E-10 | -1,676 |
| DBF4 | DBF4 homolog (S. cerevisiae) |  |  | 4,84E-09 | -1,540 |  |  |
| DBN1 | drebrin 1 |  |  | 4,50E-08 | -2,185 |  |  |
| DBNDD2 | dysbindin (dystrobrevin binding protein 1) domain containing 2 |  |  | 1,74E-08 | -1,853 |  |  |
| DBR1 | debranching enzyme homolog 1 (S. cerevisiae) |  |  | 3,80E-10 | -1,622 |  |  |
| DC2 | oligosaccharyltransferase complex subunit |  |  | 1,95E-09 | -1,538 | 1,95E-09 | -1,448 |
| DCK | deoxycytidine kinase |  |  | 7,16E-10 | -1,821 |  |  |
| DCTD | dCMP deaminase |  |  | 2,92E-09 | -1,649 | 2,92E-09 | -1,581 |
| DCTD | dCMP deaminase |  |  | 1,08E-07 | -1,720 |  |  |
| DCTN1 | dynactin 1 (p150, glued homolog, Drosophila) |  |  | 1,85E-12 | -1,872 |  |  |
| DCTN3 | dynactin 3 (p22) |  |  | 6,87E-13 | -1,779 |  |  |
| DCTN4 | dynactin 4 (p62) |  |  | 1,01E-08 | -1,547 |  |  |
| DCTN6 | dynactin 6 |  |  | 1,29E-09 | -1,619 |  |  |
| DCUN1D5 | DCN1, defective in cullin neddylation 1, domain containing 5 (S. cerevisiae) |  |  | 4,07E-12 | -1,622 |  |  |
| DDAH2 | dimethylarginine dimethylaminohydrolase 2 |  |  | 2,31E-10 | -2,297 | 2,31E-10 | -1,625 |
| DDB1 | damage-specific DNA binding protein 1, 127kDa |  |  | 2,55E-08 | -1,647 |  |  |
| DDOST | dolichyl-diphosphooligosaccharide-protein glycosyltransferase |  |  | 6,17E-08 | -1,602 |  |  |
| DDR1 | discoidin domain receptor tyrosine kinase 1 |  |  | 1,69E-14 | -2,220 | 1,69E-14 | -1,589 |
| DDR1 | discoidin domain receptor tyrosine kinase 1 |  |  | 5,37E-09 | -1,785 |  |  |
| DDR1 | discoidin domain receptor tyrosine kinase 1 |  |  | 1,69E-14 | -2,163 |  |  |
| DDT | D-dopachrome tautomerase |  |  | 1,39E-08 | -1,678 |  |  |
| DDX11 | DEAD/H (Asp-Glu-Ala-Asp/His) box polypeptide 11 (CHL1-like helicase homolog, S. cerevisiae) |  |  | 5,05E-09 | -1,892 |  |  |
| DDX11 | DEAD/H (Asp-Glu-Ala-Asp/His) box polypeptide 11 (CHL1-like helicase homolog, S. cerevisiae) |  |  | 1,37E-11 | -2,566 |  |  |
| DDX17 | DEAD (Asp-Glu-Ala-Asp) box polypeptide 17 |  |  |  |  | 2,15E-09 | 2,013 |
| DDX18 | DEAD (Asp-Glu-Ala-Asp) box polypeptide 18 |  |  | 3,17E-09 | -1,568 |  |  |
| DDX19A | DEAD (Asp-Glu-Ala-As) box polypeptide 19A |  |  | 2,21E-09 | -1,809 |  |  |
| DDX23 | DEAD (Asp-Glu-Ala-Asp) box polypeptide 23 |  |  | 6,05E-12 | -2,035 |  |  |
| DDX23 | DEAD (Asp-Glu-Ala-Asp) box polypeptide 23 |  |  | 1,32E-10 | -1,716 |  |  |
| DDX24 | DEAD (Asp-Glu-Ala-Asp) box polypeptide 24 |  |  | 7,73E-12 | -1,828 |  |  |
| DDX24 | DEAD (Asp-Glu-Ala-Asp) box polypeptide 24 |  |  |  |  | 9,71E-13 | 1,616 |
| DDX3X | DEAD (Asp-Glu-Ala-Asp) box polypeptide 3, X-linked |  |  | 3,26E-11 | -1,540 |  |  |
| DDX3X | DEAD (Asp-Glu-Ala-Asp) box polypeptide 3, X-linked |  |  |  |  | 1,59E-10 | -1,893 |
| DDX3X | DEAD (Asp-Glu-Ala-Asp) box polypeptide 3, X-linked |  |  | 9,15E-13 | -1,736 | 9,15E-13 | -1,650 |
| DDX41 | DEAD (Asp-Glu-Ala-Asp) box polypeptide 41 |  |  | 5,90E-11 | -1,827 |  |  |
| DDX42 | DEAD (Asp-Glu-Ala-Asp) box polypeptide 42 |  |  | 1,89E-09 | -1,877 | 1,89E-09 | -1,610 |
| DDX47 | DEAD (Asp-Glu-Ala-Asp) box polypeptide 47 |  |  | 1,54E-10 | -1,662 |  |  |
| DDX52 | DEAD (Asp-Glu-Ala-Asp) box polypeptide 52 |  |  | 1,69E-14 | -1,978 | 1,69E-14 | -1,725 |
| DDX56 | DEAD (Asp-Glu-Ala-Asp) box polypeptide 56 |  |  | 1,60E-07 | -1,561 |  |  |
| DECR1 | 2,4-dienoyl CoA reductase 1, mitochondrial |  |  | 3,37E-09 | -1,892 |  |  |
| DEFB1 | defensin, beta 1 |  |  | 1,55E-10 | -2,669 | 1,55E-10 | -2,232 |
| DENND4C | DENN/MADD domain containing 4C |  |  | 1,13E-10 | -1,723 |  |  |
| DENR (includes EG:8562) | density-regulated protein |  |  | 3,30E-10 | -1,660 |  |  |
| DEPDC1 | DEP domain containing 1 |  |  | 2,24E-08 | -1,581 |  |  |
| DEPDC1 | DEP domain containing 1 |  |  | 3,20E-12 | -1,948 | 3,20E-12 | -1,868 |
| DEPDC1B | DEP domain containing 1B |  |  | 1,64E-09 | -1,564 |  |  |
| DERA | 2-deoxyribose-5-phosphate aldolase homolog (C. elegans) |  |  | 1,99E-08 | -1,451 |  |  |
| DGCR6 | DiGeorge syndrome critical region gene 6 |  |  | 3,36E-08 | -2,056 |  |  |
| DGCR8 | DiGeorge syndrome critical region gene 8 |  |  | 1,88E-09 | -1,989 |  |  |
| DGKZ | diacylglycerol kinase, zeta 104kDa |  |  | 4,46E-10 | -1,927 |  |  |
| DGUOK | deoxyguanosine kinase |  |  | 1,57E-08 | -1,546 |  |  |
| DHCR24 | 24-dehydrocholesterol reductase |  |  | 1,95E-11 | -1,780 |  |  |
| DHCR7 | 7-dehydrocholesterol reductase |  |  | 7,15E-11 | -1,933 |  |  |
| DHCR7 | 7-dehydrocholesterol reductase |  |  | 1,69E-14 | -2,112 | 1,69E-14 | -1,535 |
| DHFR | dihydrofolate reductase |  |  | 1,69E-14 | -1,893 |  |  |
| DHFR | dihydrofolate reductase |  |  | 1,76E-10 | -1,748 |  |  |
| DHPS | deoxyhypusine synthase |  |  | 1,65E-07 | -2,065 |  |  |
| DHRS11 | dehydrogenase/reductase (SDR family) member 11 |  |  | 1,24E-07 | -1,874 |  |  |
| DHRS2 (includes EG:10202) | dehydrogenase/reductase (SDR family) member 2 |  |  | 1,00E-11 | -1,853 | 1,00E-11 | -1,630 |
| DHRS7 (includes EG:51635) | dehydrogenase/reductase (SDR family) member 7 |  |  |  |  | 2,77E-08 | -1,550 |
| DHTKD1 | dehydrogenase E1 and transketolase domain containing 1 |  |  | 5,73E-09 | -1,879 |  |  |
| DHX29 | DEAH (Asp-Glu-Ala-His) box polypeptide 29 |  |  | 1,72E-07 | -1,549 |  |  |
| DHX33 | DEAH (Asp-Glu-Ala-His) box polypeptide 33 | 6,11E-13 | -1,523 | 6,11E-13 | -1,973 | 6,11E-13 | -1,376 |
| DHX40 | DEAH (Asp-Glu-Ala-His) box polypeptide 40 |  |  | 4,29E-09 | -1,531 |  |  |
| DHX9 | DEAH (Asp-Glu-Ala-His) box polypeptide 9 | 7,41E-08 | -1,727 |  |  |  |  |
| DIAPH1 | diaphanous homolog 1 (Drosophila) |  |  | 6,90E-10 | -1,648 |  |  |
| DIAPH3 | diaphanous homolog 3 (Drosophila) |  |  | 1,34E-12 | -1,828 |  |  |
| DICER1 | dicer 1, ribonuclease type III |  |  | 3,00E-10 | -1,627 |  |  |
| DIDO1 | death inducer-obliterator 1 |  |  | 6,18E-10 | -1,819 |  |  |
| DIMT1L | DIM1 dimethyladenosine transferase 1-like (S. cerevisiae) |  |  | 6,78E-09 | -1,905 |  |  |
| DIRC2 | disrupted in renal carcinoma 2 |  |  | 1,33E-11 | -1,750 |  |  |
| DIS3L | DIS3 mitotic control homolog (S. cerevisiae)-like |  |  | 1,98E-08 | -1,659 |  |  |
| DKC1 | dyskeratosis congenita 1, dyskerin |  |  | 1,48E-10 | -1,878 |  |  |
| DKC1 | dyskeratosis congenita 1, dyskerin |  |  | 3,35E-12 | -1,734 |  |  |
| DKC1 | dyskeratosis congenita 1, dyskerin |  |  |  |  | 1,04E-07 | 1,490 |
| DKK3 | dickkopf homolog 3 (Xenopus laevis) |  |  | 5,96E-11 | -1,861 | 5,96E-11 | -1,991 |
| DLAT | dihydrolipoamide S-acetyltransferase |  |  | 1,85E-11 | -1,585 | 1,85E-11 | -1,538 |
| DLEU2 | deleted in lymphocytic leukemia 2 (non-protein coding) |  |  | 1,93E-07 | -1,755 |  |  |
| DLG1 | discs, large homolog 1 (Drosophila) |  |  | 2,63E-13 | -1,580 | 2,63E-13 | -1,926 |
| DLGAP5 | discs, large (Drosophila) homolog-associated protein 5 |  |  | 1,69E-14 | -1,927 | 1,69E-14 | -1,518 |
| DLST | dihydrolipoamide S-succinyltransferase (E2 component of 2-oxo-glutarate complex) | 1,69E-14 | 2,345 | 1,69E-14 | 2,839 |  |  |
| DMKN | dermokine |  |  | 1,21E-09 | -1,627 |  |  |
| DNA2 | DNA replication helicase 2 homolog (yeast) |  |  | 1,53E-13 | -1,800 |  |  |
| DNAJA1 | DnaJ (Hsp40) homolog, subfamily A, member 1 |  |  | 4,40E-11 | -1,670 |  |  |
| DNAJA3 | DnaJ (Hsp40) homolog, subfamily A, member 3 |  |  | 1,54E-07 | -1,631 |  |  |
| DNAJA3 | DnaJ (Hsp40) homolog, subfamily A, member 3 |  |  | 5,65E-08 | -1,796 |  |  |
| DNAJB12 | DnaJ (Hsp40) homolog, subfamily B, member 12 |  |  | 3,19E-10 | -2,158 | 3,19E-10 | -1,519 |
| DNAJB6 | DnaJ (Hsp40) homolog, subfamily B, member 6 |  |  | 1,32E-08 | -1,662 |  |  |
| DNAJB9 | DnaJ (Hsp40) homolog, subfamily B, member 9 |  |  | 1,16E-12 | -1,715 | 1,16E-12 | -1,858 |
| DNAJC15 | DnaJ (Hsp40) homolog, subfamily C, member 15 |  |  | 3,87E-10 | -1,688 |  |  |
| DNAJC19 | DnaJ (Hsp40) homolog, subfamily C, member 19 |  |  | 1,78E-08 | -1,660 |  |  |
| DNAJC19 | DnaJ (Hsp40) homolog, subfamily C, member 19 |  |  | 5,31E-13 | -1,961 |  |  |
| DNAJC8 | DnaJ (Hsp40) homolog, subfamily C, member 8 |  |  | 3,25E-08 | -1,772 |  |  |
| DNMBP | dynamin binding protein |  |  | 6,68E-12 | -2,012 |  |  |
| DNMT3B | DNA (cytosine-5-)-methyltransferase 3 beta |  |  | 1,10E-09 | -1,651 |  |  |
| DNPEP | aspartyl aminopeptidase |  |  | 2,05E-08 | -1,816 |  |  |
| DNPEP | aspartyl aminopeptidase |  |  | 3,93E-09 | -1,678 |  |  |
| DNTTIP1 | deoxynucleotidyltransferase, terminal, interacting protein 1 |  |  | 1,16E-10 | -1,983 | 1,16E-10 | -1,636 |
| DNTTIP1 | deoxynucleotidyltransferase, terminal, interacting protein 1 |  |  | 2,82E-10 | -1,681 |  |  |
| DNTTIP2 | deoxynucleotidyltransferase, terminal, interacting protein 2 |  |  | 5,77E-14 | -1,880 |  |  |
| DOK4 | docking protein 4 |  |  | 5,39E-09 | -1,734 | 5,39E-09 | -1,533 |
| DOLPP1 | dolichyl pyrophosphate phosphatase 1 |  |  | 9,38E-12 | -1,949 |  |  |
| DONSON | downstream neighbor of SON |  |  | 3,66E-09 | -1,509 |  |  |
| DPF2 | D4, zinc and double PHD fingers family 2 |  |  | 3,27E-09 | -1,784 |  |  |
| DPH1 | DPH1 homolog (S. cerevisiae) |  |  | 1,21E-11 | -2,008 |  |  |
| DPH3 | DPH3, KTI11 homolog (S. cerevisiae) |  |  | 2,08E-09 | -1,563 |  |  |
| DPM2 | dolichyl-phosphate mannosyltransferase polypeptide 2, regulatory subunit | 6,19E-11 | -1,623 | 6,19E-11 | -2,046 | 6,19E-11 | -1,575 |
| DPP4 | dipeptidyl-peptidase 4 |  |  | 2,44E-13 | -1,785 |  |  |
| DPP4 | dipeptidyl-peptidase 4 |  |  | 9,32E-11 | -1,767 |  |  |
| DPY30 | dpy-30 homolog (C. elegans) |  |  | 4,12E-11 | -1,577 | 4,12E-11 | -1,656 |
| DPYSL2 | dihydropyrimidinase-like 2 |  |  | 5,94E-10 | -1,573 |  |  |
| DPYSL3 | dihydropyrimidinase-like 3 |  |  | 8,49E-12 | -2,048 | 8,49E-12 | -1,877 |
| DSN1 | DSN1, MIND kinetochore complex component, homolog (S. cerevisiae) |  |  | 4,31E-09 | -1,678 |  |  |
| DST | dystonin |  |  | 2,94E-06 | -1,422 |  |  |
| DST | dystonin |  |  | 9,92E-11 | -1,553 |  |  |
| DST | dystonin |  |  |  |  | 2,11E-09 | 1,855 |
| DSTN | destrin (actin depolymerizing factor) |  |  | 3,74E-09 | -1,449 |  |  |
| DUSP11 | dual specificity phosphatase 11 (RNA/RNP complex 1-interacting) |  |  | 1,65E-10 | -1,588 |  |  |
| DUSP16 | dual specificity phosphatase 16 |  |  | 1,14E-08 | -1,758 |  |  |
| DUSP22 | dual specificity phosphatase 22 |  |  | 9,44E-08 | -1,988 |  |  |
| DUSP3 | dual specificity phosphatase 3 |  |  | 5,10E-11 | -1,962 |  |  |
| DUSP3 | dual specificity phosphatase 3 |  |  | 5,77E-14 | -2,376 |  |  |
| DUSP5 | dual specificity phosphatase 5 |  |  |  |  | 1,31E-13 | 2,007 |
| DUSP6 | dual specificity phosphatase 6 |  |  |  |  | 1,69E-14 | 1,484 |
| DUSP6 | dual specificity phosphatase 6 |  |  |  |  | 1,69E-14 | 2,196 |
| DUT (includes EG:1854) | deoxyuridine triphosphatase |  |  | 3,12E-10 | -1,933 |  |  |
| DUT (includes EG:1854) | deoxyuridine triphosphatase |  |  | 4,20E-10 | -1,631 |  |  |
| DVL2 (includes EG:1856) | dishevelled, dsh homolog 2 (Drosophila) |  |  | 1,85E-12 | -2,020 |  |  |
| DVL3 | dishevelled, dsh homolog 3 (Drosophila) |  |  | 1,25E-11 | -1,904 |  |  |
| DYM | dymeclin |  |  | 2,47E-09 | -1,880 |  |  |
| DYNC1H1 | dynein, cytoplasmic 1, heavy chain 1 |  |  | 5,28E-11 | -1,853 |  |  |
| DYNLRB1 | dynein, light chain, roadblock-type 1 |  |  | 2,25E-10 | -1,648 |  |  |
| DYNLT1 | dynein, light chain, Tctex-type 1 |  |  | 1,64E-09 | -1,560 |  |  |
| EAF1 | ELL associated factor 1 |  |  | 6,16E-09 | -1,688 |  |  |
| EAPP | E2F-associated phosphoprotein |  |  | 1,69E-14 | -2,045 |  |  |
| EBP | emopamil binding protein (sterol isomerase) |  |  | 8,68E-11 | -1,864 |  |  |
| ECHDC1 | enoyl Coenzyme A hydratase domain containing 1 |  |  |  |  | 6,56E-09 | -1,691 |
| ECHDC3 | enoyl Coenzyme A hydratase domain containing 3 |  |  | 5,37E-11 | -1,944 |  |  |
| ECHS1 | enoyl Coenzyme A hydratase, short chain, 1, mitochondrial |  |  | 2,33E-09 | -1,573 |  |  |
| ECOP | EGFR-coamplified and overexpressed protein |  |  | 9,80E-09 | -1,789 |  |  |
| ECT2 | epithelial cell transforming sequence 2 oncogene |  |  | 1,04E-09 | -1,533 |  |  |
| EDEM2 | ER degradation enhancer, mannosidase alpha-like 2 |  |  | 1,68E-11 | -1,811 |  |  |
| EDN1 | endothelin 1 |  |  | 1,57E-08 | -1,833 |  |  |
| EEA1 | early endosome antigen 1 |  |  |  |  | 2,14E-09 | 2,279 |
| EED | embryonic ectoderm development |  |  | 3,38E-11 | -1,578 | 3,38E-11 | -1,483 |
| EEF1D | eukaryotic translation elongation factor 1 delta (guanine nucleotide exchange protein) | 1,69E-14 | -1,378 | 1,69E-14 | -1,839 |  |  |
| EFCAB2 | EF-hand calcium binding domain 2 |  |  | 1,72E-10 | -1,793 |  |  |
| EFCAB7 | EF-hand calcium binding domain 7 |  |  | 1,49E-08 | -2,257 |  |  |
| EFNB2 | ephrin-B2 |  |  | 2,86E-10 | -1,404 |  |  |
| EFNB2 | ephrin-B2 |  |  |  |  | 6,72E-13 | 2,150 |
| EFTUD2 | elongation factor Tu GTP binding domain containing 2 |  |  | 2,20E-12 | -1,908 |  |  |
| EGLN1 | egl nine homolog 1 (C. elegans) |  |  | 1,53E-09 | -1,651 |  |  |
| EGR1 | early growth response 1 |  |  |  |  | 2,17E-12 | 2,951 |
| EGR1 | early growth response 1 |  |  |  |  | 1,08E-09 | 1,507 |
| EHBP1 | EH domain binding protein 1 |  |  |  |  | 1,69E-14 | 2,384 |
| EI24 | etoposide induced 2.4 mRNA |  |  | 5,04E-09 | -1,541 |  |  |
| EID1 | EP300 interacting inhibitor of differentiation 1 |  |  | 1,75E-11 | -1,891 | 1,75E-11 | -1,583 |
| EIF1AX | eukaryotic translation initiation factor 1A, X-linked |  |  | 2,18E-09 | -1,467 |  |  |
| EIF2AK1 | eukaryotic translation initiation factor 2-alpha kinase 1 |  |  | 2,22E-09 | -1,608 |  |  |
| EIF2AK4 | eukaryotic translation initiation factor 2 alpha kinase 4 |  |  | 2,57E-11 | -2,036 |  |  |
| EIF2C2 | eukaryotic translation initiation factor 2C, 2 |  |  | 2,81E-13 | -2,045 |  |  |
| EIF2S1 | eukaryotic translation initiation factor 2, subunit 1 alpha, 35kDa |  |  | 2,63E-13 | -1,906 |  |  |
| EIF2S3 | eukaryotic translation initiation factor 2, subunit 3 gamma, 52kDa |  |  |  |  | 1,63E-13 | 1,659 |
| EIF3A | eukaryotic translation initiation factor 3, subunit A |  |  | 2,81E-13 | -1,526 |  |  |
| EIF3B | eukaryotic translation initiation factor 3, subunit B |  |  | 3,30E-11 | -1,648 |  |  |
| EIF3B | eukaryotic translation initiation factor 3, subunit B |  |  | 6,35E-09 | -1,600 |  |  |
| EIF3C | eukaryotic translation initiation factor 3, subunit C |  |  | 9,44E-09 | -1,484 |  |  |
| EIF3C | eukaryotic translation initiation factor 3, subunit C |  |  | 6,82E-12 | -1,634 |  |  |
| EIF3C | eukaryotic translation initiation factor 3, subunit C |  |  |  |  | 2,86E-09 | 1,658 |
| EIF3D | eukaryotic translation initiation factor 3, subunit D |  |  | 1,53E-11 | -1,699 |  |  |
| EIF3I (includes EG:8668) | eukaryotic translation initiation factor 3, subunit I |  |  | 1,06E-08 | -1,592 |  |  |
| EIF3J | eukaryotic translation initiation factor 3, subunit J |  |  | 2,99E-09 | -1,636 |  |  |
| EIF3K | eukaryotic translation initiation factor 3, subunit K |  |  | 1,69E-09 | -1,600 |  |  |
| EIF3K | eukaryotic translation initiation factor 3, subunit K |  |  | 2,92E-09 | -1,657 |  |  |
| EIF4B | eukaryotic translation initiation factor 4B | 3,46E-11 | -1,567 | 3,46E-11 | -1,822 | 3,46E-11 | -2,306 |
| EIF4E | eukaryotic translation initiation factor 4E |  |  |  |  | 3,55E-08 | -1,423 |
| EIF4E2 | eukaryotic translation initiation factor 4E family member 2 |  |  | 1,52E-07 | -1,544 |  |  |
| EIF4EBP1 | eukaryotic translation initiation factor 4E binding protein 1 |  |  | 3,01E-08 | -1,810 |  |  |
| EIF4G1 | eukaryotic translation initiation factor 4 gamma, 1 | 1,69E-14 | -1,827 | 1,69E-14 | -1,518 |  |  |
| EIF4G1 | eukaryotic translation initiation factor 4 gamma, 1 |  |  | 1,42E-10 | -1,783 |  |  |
| EIF5 | eukaryotic translation initiation factor 5 |  |  | 1,16E-11 | -1,707 |  |  |
| EIF5A | eukaryotic translation initiation factor 5A |  |  |  |  | 6,24E-10 | 1,901 |
| EIF5A | eukaryotic translation initiation factor 5A |  |  |  |  | 1,69E-14 | 2,059 |
| EIF5B | eukaryotic translation initiation factor 5B |  |  | 1,69E-14 | -2,038 |  |  |
| EIF5B | eukaryotic translation initiation factor 5B |  |  | 4,60E-12 | -1,865 |  |  |
| ELF1 | E74-like factor 1 (ets domain transcription factor) |  |  |  |  | 6,80E-09 | 1,709 |
| ELMO2 | engulfment and cell motility 2 |  |  | 1,48E-08 | -1,502 |  |  |
| ELMOD2 | ELMO/CED-12 domain containing 2 |  |  | 8,21E-10 | -1,761 |  |  |
| EMG1 | EMG1 nucleolar protein homolog (S. cerevisiae) |  |  | 3,05E-09 | -1,581 |  |  |
| EML4 | echinoderm microtubule associated protein like 4 |  |  | 3,50E-12 | -1,625 | 3,50E-12 | -1,688 |
| EMP2 | epithelial membrane protein 2 |  |  | 1,18E-10 | -1,770 |  |  |
| EMP2 | epithelial membrane protein 2 |  |  | 1,69E-14 | -2,191 | 1,69E-14 | -1,512 |
| EMP2 | epithelial membrane protein 2 |  |  | 6,53E-10 | -1,962 |  |  |
| ENC1 | ectodermal-neural cortex (with BTB-like domain) |  |  |  |  | 1,69E-14 | 2,019 |
| ENDOD1 | endonuclease domain containing 1 |  |  |  |  | 1,38E-11 | 1,435 |
| ENDOD1 | endonuclease domain containing 1 |  |  | 7,33E-10 | -1,635 |  |  |
| ENO1 | enolase 1, (alpha) |  |  | 1,15E-10 | -1,602 | 1,15E-10 | -1,435 |
| ENOSF1 | enolase superfamily member 1 |  |  | 7,36E-10 | -1,698 |  |  |
| ENSA | endosulfine alpha |  |  | 1,16E-08 | -1,753 |  |  |
| ENSA | endosulfine alpha |  |  | 1,00E-08 | -1,774 |  |  |
| ENY2 | enhancer of yellow 2 homolog (Drosophila) |  |  | 3,14E-14 | -1,889 | 3,14E-14 | -1,545 |
| EPB41L4B | erythrocyte membrane protein band 4.1 like 4B |  |  | 6,71E-10 | -1,542 |  |  |
| EPB41L5 | erythrocyte membrane protein band 4.1 like 5 |  |  | 1,22E-12 | -1,896 | 1,22E-12 | -1,656 |
| EPB41L5 | erythrocyte membrane protein band 4.1 like 5 |  |  | 5,37E-09 | -1,711 |  |  |
| EPHA4 | EPH receptor A4 | 4,79E-13 | -1,369 | 4,79E-13 | -1,931 | 4,79E-13 | -1,632 |
| EPHB4 | EPH receptor B4 |  |  | 3,10E-13 | -2,608 |  |  |
| EPM2AIP1 | EPM2A (laforin) interacting protein 1 |  |  | 1,95E-13 | -1,933 |  |  |
| EPR1 | effector cell peptidase receptor 1 (non-protein coding) |  |  | 3,47E-12 | -2,205 | 3,47E-12 | -2,277 |
| EPRS | glutamyl-prolyl-tRNA synthetase |  |  | 1,94E-09 | -1,631 |  |  |
| EPS8L2 | EPS8-like 2 |  |  | 1,33E-09 | -1,942 |  |  |
| EPSTI1 | epithelial stromal interaction 1 (breast) |  |  | 8,35E-14 | -2,115 | 8,35E-14 | -1,956 |
| ERBB2 | v-erb-b2 erythroblastic leukemia viral oncogene homolog 2, neuro/glioblastoma derived oncogene homolog (avian) |  |  | 6,61E-10 | -2,175 |  |  |
| ERBB3 | v-erb-b2 erythroblastic leukemia viral oncogene homolog 3 (avian) |  |  | 1,31E-13 | -1,878 | 1,31E-13 | -1,681 |
| ERBB3 | v-erb-b2 erythroblastic leukemia viral oncogene homolog 3 (avian) |  |  | 1,69E-14 | -2,219 | 1,69E-14 | -1,631 |
| ERGIC1 | endoplasmic reticulum-golgi intermediate compartment (ERGIC) 1 |  |  | 3,51E-12 | -1,689 |  |  |
| ERGIC1 | endoplasmic reticulum-golgi intermediate compartment (ERGIC) 1 |  |  | 4,07E-11 | -1,947 |  |  |
| ERGIC3 | ERGIC and golgi 3 |  |  | 1,69E-14 | -2,060 |  |  |
| ERLIN1 | ER lipid raft associated 1 |  |  | 3,74E-11 | -1,756 | 3,74E-11 | -1,888 |
| ERLIN2 | ER lipid raft associated 2 |  |  | 1,36E-11 | -1,701 |  |  |
| ESCO1 | establishment of cohesion 1 homolog 1 (S. cerevisiae) |  |  |  |  | 1,63E-10 | 2,549 |
| ESF1 | ESF1, nucleolar pre-rRNA processing protein, homolog (S. cerevisiae) |  |  |  |  | 1,69E-14 | 1,692 |
| ESPL1 | extra spindle pole bodies homolog 1 (S. cerevisiae) |  |  | 5,96E-12 | -1,979 |  |  |
| ETAA1 | Ewing tumor-associated antigen 1 | 5,94E-07 | -1,447 |  |  |  |  |
| ETFA | electron-transfer-flavoprotein, alpha polypeptide |  |  | 2,95E-10 | -1,564 |  |  |
| ETHE1 | ethylmalonic encephalopathy 1 |  |  | 1,82E-09 | -1,768 |  |  |
| ETS1 | v-ets erythroblastosis virus E26 oncogene homolog 1 (avian) |  |  |  |  | 1,69E-14 | 1,762 |
| EVI1 | ecotropic viral integration site 1 |  |  | 1,53E-13 | -2,164 |  |  |
| EWSR1 | Ewing sarcoma breakpoint region 1 |  |  | 6,82E-12 | -2,127 |  |  |
| EWSR1 | Ewing sarcoma breakpoint region 1 |  |  |  |  | 7,62E-07 | 1,832 |
| EXOC4 | exocyst complex component 4 |  |  | 4,96E-11 | -2,030 |  |  |
| EXOC6 | exocyst complex component 6 |  |  | 6,17E-08 | -1,807 |  |  |
| EXOC7 | exocyst complex component 7 |  |  | 3,24E-09 | -1,759 |  |  |
| EXOSC1 | exosome component 1 |  |  | 1,81E-07 | -1,615 |  |  |
| EXOSC3 | exosome component 3 |  |  | 4,21E-06 | -1,577 |  |  |
| EXOSC4 | exosome component 4 |  |  | 9,23E-08 | -1,697 |  |  |
| EXOSC4 | exosome component 4 |  |  | 4,03E-11 | -2,016 |  |  |
| EXOSC8 | exosome component 8 |  |  | 2,33E-10 | -1,624 |  |  |
| EZR | ezrin |  |  | 4,38E-08 | -1,658 |  |  |
| EZR | ezrin |  |  | 5,77E-14 | -2,013 |  |  |
| F12 | coagulation factor XII (Hageman factor) |  |  | 1,94E-08 | -2,159 |  |  |
| F7 | coagulation factor VII (serum prothrombin conversion accelerator) |  |  | 2,01E-09 | -2,413 |  |  |
| F8A1 | coagulation factor VIII-associated (intronic transcript) 1 |  |  | 9,94E-09 | -1,744 |  |  |
| FABP1 | fatty acid binding protein 1, liver |  |  | 1,69E-14 | -2,118 |  |  |
| FAF2 | Fas associated factor family member 2 |  |  |  |  | 1,49E-12 | 1,845 |
| FAHD1 | fumarylacetoacetate hydrolase domain containing 1 |  |  |  |  | 5,79E-07 | -1,375 |
| FAHD1 | fumarylacetoacetate hydrolase domain containing 1 |  |  | 2,31E-09 | -1,644 |  |  |
| FAHD2A | fumarylacetoacetate hydrolase domain containing 2A |  |  | 1,42E-11 | -2,247 |  |  |
| FAIM | Fas apoptotic inhibitory molecule |  |  | 1,69E-14 | -2,061 | 1,69E-14 | -1,636 |
| FAM103A1 | family with sequence similarity 103, member A1 |  |  | 4,98E-10 | -1,511 |  |  |
| FAM108A1 | family with sequence similarity 108, member A1 |  |  | 6,32E-11 | -2,012 |  |  |
| FAM111B | family with sequence similarity 111, member B |  |  |  |  | 2,32E-10 | 1,774 |
| FAM115A | family with sequence similarity 115, member A |  |  | 1,69E-14 | -2,010 | 1,69E-14 | -2,042 |
| FAM116A | family with sequence similarity 116, member A |  |  | 4,20E-09 | -1,618 |  |  |
| FAM119A | family with sequence similarity 119, member A |  |  | 2,18E-08 | -1,613 |  |  |
| FAM120A | family with sequence similarity 120A |  |  | 3,14E-14 | -2,274 |  |  |
| FAM120A | family with sequence similarity 120A |  |  | 1,67E-11 | -1,602 |  |  |
| FAM120AOS | family with sequence similarity 120A opposite strand |  |  | 4,07E-11 | -1,864 |  |  |
| FAM126B | family with sequence similarity 126, member B |  |  | 3,75E-06 | -1,403 |  |  |
| FAM128A | family with sequence similarity 128, member A |  |  | 3,19E-09 | -1,696 |  |  |
| FAM128B | family with sequence similarity 128, member B |  |  | 1,37E-07 | -1,659 |  |  |
| FAM134A | family with sequence similarity 134, member A |  |  | 7,58E-08 | -1,610 |  |  |
| FAM134A | family with sequence similarity 134, member A |  |  | 5,79E-09 | -1,581 |  |  |
| FAM134C | family with sequence similarity 134, member C |  |  | 3,18E-08 | -1,589 |  |  |
| FAM13A1 | family with sequence similarity 13, member A1 |  |  | 1,31E-07 | -2,008 |  |  |
| FAM152A | family with sequence similarity 152, member A |  |  | 1,69E-14 | -1,923 |  |  |
| FAM171B | family with sequence similarity 171, member B |  |  | 6,50E-08 | -1,655 |  |  |
| FAM32A | family with sequence similarity 32, member A |  |  | 2,33E-09 | -1,700 |  |  |
| FAM33A | family with sequence similarity 33, member A |  |  | 1,69E-14 | -1,809 |  |  |
| FAM36A | family with sequence similarity 36, member A |  |  | 1,36E-08 | -1,691 |  |  |
| FAM36A | family with sequence similarity 36, member A |  |  | 3,17E-11 | -1,726 |  |  |
| FAM38A | family with sequence similarity 38, member A |  |  | 7,60E-11 | -1,745 |  |  |
| FAM3B | family with sequence similarity 3, member B |  |  | 1,69E-14 | -2,540 | 1,69E-14 | -1,899 |
| FAM44B | family with sequence similarity 44, member B |  |  | 3,93E-09 | -1,572 |  |  |
| FAM45A | family with sequence similarity 45, member A |  |  | 3,60E-11 | -1,741 |  |  |
| FAM53C | family with sequence similarity 53, member C |  |  | 5,68E-08 | -1,694 |  |  |
| FAM62A | family with sequence similarity 62 (C2 domain containing), member A |  |  | 1,75E-11 | -2,119 |  |  |
| FAM64A | family with sequence similarity 64, member A |  |  | 3,25E-09 | -1,653 |  |  |
| FAM76B | family with sequence similarity 76, member B |  |  | 3,12E-11 | 2,363 |  |  |
| FAM82B | family with sequence similarity 82, member B |  |  | 4,27E-12 | -1,800 |  |  |
| FAM83D | family with sequence similarity 83, member D |  |  | 1,96E-10 | -1,468 | 1,96E-10 | -1,448 |
| FAM83H | family with sequence similarity 83, member H |  |  | 2,07E-09 | -1,607 |  |  |
| FAM86B1 | family with sequence similarity 86, member B1 |  |  | 1,21E-07 | -1,746 |  |  |
| FAM89A | family with sequence similarity 89, member A |  |  | 1,93E-08 | -2,122 |  |  |
| FAM91A1 | family with sequence similarity 91, member A1 |  |  | 1,69E-14 | -1,945 | 1,69E-14 | -1,513 |
| FAM96A | family with sequence similarity 96, member A |  |  | 1,69E-08 | -1,451 |  |  |
| FAM98B | family with sequence similarity 98, member B |  |  | 2,44E-09 | -1,634 |  |  |
| FANCI | Fanconi anemia, complementation group I |  |  | 4,96E-10 | -1,889 |  |  |
| FAR1 | fatty acyl CoA reductase 1 |  |  | 1,81E-12 | -1,620 | 1,81E-12 | -1,770 |
| FARS2 | phenylalanyl-tRNA synthetase 2, mitochondrial |  |  | 2,62E-10 | -2,037 |  |  |
| FARSA | phenylalanyl-tRNA synthetase, alpha subunit |  |  | 2,94E-08 | -2,124 |  |  |
| FASN | fatty acid synthase |  |  | 6,41E-09 | -1,860 |  |  |
| FASTKD3 | FAST kinase domains 3 |  |  | 2,10E-10 | -1,575 |  |  |
| FBL | fibrillarin |  |  | 1,35E-08 | -1,631 |  |  |
| FBXL14 | F-box and leucine-rich repeat protein 14 |  |  | 9,37E-13 | -2,006 |  |  |
| FBXL3 | F-box and leucine-rich repeat protein 3 |  |  | 1,55E-11 | -1,772 |  |  |
| FBXO2 | F-box protein 2 |  |  | 1,69E-14 | -2,406 | 1,69E-14 | -1,758 |
| FBXO21 | F-box protein 21 |  |  | 1,19E-13 | -1,994 | 1,19E-13 | -1,547 |
| FBXO22 | F-box protein 22 | 5,56E-12 | -1,436 | 5,56E-12 | -1,837 | 5,56E-12 | -1,706 |
| FBXO28 | F-box protein 28 |  |  | 5,58E-10 | -1,636 | 5,58E-10 | -1,524 |
| FBXO33 | F-box protein 33 |  |  | 8,01E-09 | -2,507 |  |  |
| FBXO41 | F-box protein 41 |  |  | 8,21E-10 | -2,012 |  |  |
| FBXO45 | F-box protein 45 |  |  | 1,85E-10 | -1,525 |  |  |
| FBXO5 | F-box protein 5 |  |  | 4,55E-13 | -1,559 | 4,55E-13 | -1,751 |
| FBXO6 | F-box protein 6 |  |  | 1,13E-09 | -2,026 |  |  |
| FBXW4 | F-box and WD repeat domain containing 4 |  |  | 5,52E-09 | -2,271 |  |  |
| FDFT1 | farnesyl-diphosphate farnesyltransferase 1 | 1,69E-14 | -1,313 | 1,69E-14 | -1,928 | 1,69E-14 | -1,611 |
| FDX1 | ferredoxin 1 |  |  | 6,72E-09 | -1,646 |  |  |
| FDX1 | ferredoxin 1 |  |  | 1,39E-08 | -1,601 |  |  |
| FEN1 | flap structure-specific endonuclease 1 |  |  | 2,38E-11 | -1,795 |  |  |
| FERMT1 | fermitin family homolog 1 (Drosophila) |  |  | 1,55E-11 | -1,777 | 1,55E-11 | -1,333 |
| FEZ2 | fasciculation and elongation protein zeta 2 (zygin II) |  |  | 6,44E-12 | -1,792 |  |  |
| FGB | fibrinogen beta chain |  |  |  |  | 5,00E-11 | -2,853 |
| FGFR1OP2 | FGFR1 oncogene partner 2 |  |  | 9,60E-13 | -1,750 |  |  |
| FGFR3 | fibroblast growth factor receptor 3 |  |  | 1,87E-08 | -1,649 |  |  |
| FGG | fibrinogen gamma chain |  |  | 4,32E-09 | -1,951 |  |  |
| FH | fumarate hydratase |  |  |  |  | 5,67E-09 | -1,702 |
| FIGNL1 | fidgetin-like 1 |  |  | 2,64E-10 | -1,640 | 2,64E-10 | -1,733 |
| FILIP1L | filamin A interacting protein 1-like |  |  | 2,71E-13 | -1,785 | 2,71E-13 | -1,662 |
| FIS1 | fission 1 (mitochondrial outer membrane) homolog (S. cerevisiae) |  |  | 9,15E-13 | -1,988 |  |  |
| FJX1 | four jointed box 1 (Drosophila) |  |  |  |  | 3,50E-11 | 2,197 |
| FKBP11 | FK506 binding protein 11, 19 kDa |  |  | 2,58E-12 | -1,815 |  |  |
| FKBP11 | FK506 binding protein 11, 19 kDa |  |  | 1,32E-07 | -1,827 |  |  |
| FKBP1A | FK506 binding protein 1A, 12kDa |  |  | 1,49E-11 | -1,674 |  |  |
| FKBP3 | FK506 binding protein 3, 25kDa |  |  | 4,91E-08 | -1,497 |  |  |
| FKBP4 | FK506 binding protein 4, 59kDa |  |  | 2,90E-10 | -1,949 |  |  |
| FKBP9 | FK506 binding protein 9, 63 kDa |  |  | 1,77E-11 | -1,691 |  |  |
| FLII | flightless I homolog (Drosophila) |  |  | 3,38E-11 | -2,006 |  |  |
| FLII | flightless I homolog (Drosophila) |  |  | 1,69E-14 | -2,097 |  |  |
| FLJ10404 | hypothetical protein FLJ10404 |  |  | 4,63E-09 | -2,290 |  |  |
| FLJ11151 | hypothetical protein FLJ11151 |  |  | 3,51E-10 | -2,270 |  |  |
| FLJ11151 | hypothetical protein FLJ11151 |  |  | 3,28E-08 | -1,842 |  |  |
| FLJ31306 | hypothetical protein FLJ31306 |  |  | 6,88E-10 | -1,821 | 6,88E-10 | -1,665 |
| FLNA | filamin A, alpha (actin binding protein 280) |  |  | 9,03E-12 | -1,716 |  |  |
| FLNA | filamin A, alpha (actin binding protein 280) |  |  | 1,69E-14 | -1,996 |  |  |
| FLNA | filamin A, alpha (actin binding protein 280) |  |  | 4,52E-10 | -2,029 |  |  |
| FLNB | filamin B, beta (actin binding protein 278) |  |  | 3,72E-10 | -1,677 |  |  |
| FLOT1 | flotillin 1 |  |  | 2,15E-10 | -1,737 |  |  |
| FLOT1 | flotillin 1 |  |  | 4,42E-09 | -1,712 |  |  |
| FLOT2 | flotillin 2 |  |  | 1,32E-10 | -1,942 |  |  |
| FLOT2 | flotillin 2 |  |  | 1,15E-08 | -2,144 | 1,15E-08 | -1,869 |
| FMC1 | formation of mitochondrial complexes 1 homolog (S. cerevisiae) |  |  | 7,50E-08 | -2,249 |  |  |
| FNBP4 | formin binding protein 4 |  |  |  |  | 5,28E-08 | 1,981 |
| FNTA | farnesyltransferase, CAAX box, alpha |  |  | 1,15E-08 | -1,525 |  |  |
| FNTA | farnesyltransferase, CAAX box, alpha |  |  | 1,13E-11 | -1,715 |  |  |
| FOLR1 | folate receptor 1 (adult) |  |  | 5,19E-10 | -2,366 |  |  |
| FOLR1 | folate receptor 1 (adult) |  |  | 1,69E-14 | 1,680 | 1,69E-14 | 1,902 |
| FOSL1 | FOS-like antigen 1 |  |  |  |  | 2,78E-08 | 1,725 |
| FOSL2 | FOS-like antigen 2 |  |  | 4,16E-09 | -1,779 |  |  |
| FOXA2 | forkhead box A2 |  |  | 3,70E-08 | -1,799 |  |  |
| FOXK1 | forkhead box K1 |  |  | 2,02E-12 | -1,998 |  |  |
| FOXM1 | forkhead box M1 |  |  | 5,00E-10 | -1,895 |  |  |
| FOXP1 | forkhead box P1 |  |  | 2,19E-11 | -1,782 |  |  |
| FOXRED1 | FAD-dependent oxidoreductase domain containing 1 |  |  | 8,31E-08 | -1,892 |  |  |
| FOXRED2 | FAD-dependent oxidoreductase domain containing 2 |  |  | 6,69E-08 | -2,068 |  |  |
| FTSJ2 | FtsJ homolog 2 (E. coli) |  |  | 3,55E-08 | -1,533 |  |  |
| FUBP1 | far upstream element (FUSE) binding protein 1 |  |  | 1,76E-10 | -1,531 | 1,76E-10 | -1,483 |
| FUNDC2 (includes EG:65991) | FUN14 domain containing 2 |  |  | 7,04E-13 | -1,783 |  |  |
| FUS | fusion (involved in t(12;16) in malignant liposarcoma) |  |  | 1,19E-08 | -1,688 |  |  |
| FUS | fusion (involved in t(12;16) in malignant liposarcoma) |  |  | 6,31E-09 | -1,631 |  |  |
| FUSIP1 | FUS interacting protein (serine/arginine-rich) 1 |  |  | 1,41E-09 | -1,531 |  |  |
| FUSIP1 | FUS interacting protein (serine/arginine-rich) 1 |  |  | 1,21E-07 | -1,549 |  |  |
| FUT4 | fucosyltransferase 4 (alpha (1,3) fucosyltransferase, myeloid-specific) |  |  | 1,33E-11 | -1,928 |  |  |
| FXYD5 | FXYD domain containing ion transport regulator 5 |  |  | 6,05E-10 | -1,930 |  |  |
| FXYD5 | FXYD domain containing ion transport regulator 5 |  |  | 1,15E-11 | -2,333 |  |  |
| FZD2 | frizzled homolog 2 (Drosophila) |  |  | 2,76E-12 | -1,853 | 2,76E-12 | -1,511 |
| FZD7 | frizzled homolog 7 (Drosophila) |  |  | 9,16E-09 | -1,581 |  |  |
| G0S2 | G0/G1switch 2 |  |  |  |  | 5,94E-10 | -1,926 |
| G3BP1 | GTPase activating protein (SH3 domain) binding protein 1 |  |  | 2,82E-12 | -1,637 |  |  |
| G3BP1 | GTPase activating protein (SH3 domain) binding protein 1 |  |  |  |  | 1,89E-06 | -1,928 |
| G3BP1 | GTPase activating protein (SH3 domain) binding protein 1 |  |  |  |  | 2,06E-08 | 2,250 |
| G3BP2 | GTPase activating protein (SH3 domain) binding protein 2 |  |  | 1,12E-10 | -1,533 |  |  |
| G6PC3 | glucose 6 phosphatase, catalytic, 3 |  |  | 2,71E-09 | -2,653 |  |  |
| G6PC3 | glucose 6 phosphatase, catalytic, 3 |  |  | 2,86E-09 | -1,730 |  |  |
| GABARAP | GABA(A) receptor-associated protein | 4,34E-12 | -1,385 | 4,34E-12 | -1,804 |  |  |
| GABARAPL1 | GABA(A) receptor-associated protein like 1 |  |  | 2,38E-12 | -1,950 |  |  |
| GAL | galanin prepropeptide |  |  | 1,69E-14 | -1,990 |  |  |
| GALC | galactosylceramidase |  |  | 1,58E-09 | -1,723 |  |  |
| GALNT1 | UDP-N-acetyl-alpha-D-galactosamine:polypeptide N-acetylgalactosaminyltransferase 1 (GalNAc-T1) | 1,69E-14 | -1,586 | 1,69E-14 | -1,559 |  |  |
| GALNT1 | UDP-N-acetyl-alpha-D-galactosamine:polypeptide N-acetylgalactosaminyltransferase 1 (GalNAc-T1) |  |  | 1,08E-07 | -1,552 |  |  |
| GALNT1 | UDP-N-acetyl-alpha-D-galactosamine:polypeptide N-acetylgalactosaminyltransferase 1 (GalNAc-T1) |  |  | 7,16E-10 | -1,629 |  |  |
| GALNT2 | UDP-N-acetyl-alpha-D-galactosamine:polypeptide N-acetylgalactosaminyltransferase 2 (GalNAc-T2) |  |  | 5,44E-11 | -2,519 |  |  |
| GALNT6 | UDP-N-acetyl-alpha-D-galactosamine:polypeptide N-acetylgalactosaminyltransferase 6 (GalNAc-T6) |  |  |  |  | 1,63E-12 | 1,573 |
| GANAB | glucosidase, alpha; neutral AB | 1,69E-14 | 3,788 | 1,69E-14 | 2,321 | 1,69E-14 | 3,561 |
| GANAB | glucosidase, alpha; neutral AB | 1,69E-14 | 4,021 | 1,69E-14 | 2,390 | 1,69E-14 | 3,032 |
| GAPVD1 | GTPase activating protein and VPS9 domains 1 |  |  | 7,91E-09 | -1,646 |  |  |
| GARNL4 | GTPase activating Rap/RanGAP domain-like 4 |  |  | 2,29E-11 | -1,990 |  |  |
| GAS2L3 | growth arrest-specific 2 like 3 |  |  | 4,00E-10 | -1,659 |  |  |
| GAS5 | growth arrest-specific 5 (non-protein coding) |  |  | 1,09E-08 | -1,515 |  |  |
| GATAD2A | GATA zinc finger domain containing 2A |  |  | 2,63E-10 | -1,841 |  |  |
| GATAD2A | GATA zinc finger domain containing 2A |  |  | 8,87E-12 | -2,151 |  |  |
| GBAS | glioblastoma amplified sequence |  |  | 1,69E-14 | -1,727 | 1,69E-14 | -1,450 |
| GBL | G protein beta subunit-like |  |  | 1,93E-11 | -2,299 |  |  |
| GCLM | glutamate-cysteine ligase, modifier subunit |  |  | 4,38E-12 | -1,952 |  |  |
| GCN1L1 | GCN1 general control of amino-acid synthesis 1-like 1 (yeast) |  |  | 1,73E-08 | -1,803 |  |  |
| GCSH | glycine cleavage system protein H (aminomethyl carrier) |  |  | 4,34E-11 | -1,621 | 4,34E-11 | -1,482 |
| GDE1 | glycerophosphodiester phosphodiesterase 1 |  |  | 1,07E-13 | -1,639 | 1,07E-13 | -1,774 |
| GDE1 | glycerophosphodiester phosphodiesterase 1 |  |  | 3,79E-12 | -2,002 |  |  |
| GEMIN7 | gem (nuclear organelle) associated protein 7 |  |  | 2,68E-10 | -2,083 |  |  |
| GEMIN7 | gem (nuclear organelle) associated protein 7 |  |  | 4,08E-09 | -1,853 |  |  |
| GFM1 | G elongation factor, mitochondrial 1 |  |  | 1,42E-09 | -1,597 |  |  |
| GFM1 | G elongation factor, mitochondrial 1 |  |  | 1,45E-12 | -1,653 | 1,45E-12 | -1,475 |
| GFM2 | G elongation factor, mitochondrial 2 |  |  | 5,17E-10 | -1,616 | 5,17E-10 | -1,685 |
| GGA1 | golgi associated, gamma adaptin ear containing, ARF binding protein 1 | 1,84E-10 | -2,127 | 1,84E-10 | -2,887 | 1,84E-10 | -1,832 |
| GGCT | gamma-glutamyl cyclotransferase |  |  | 9,08E-08 | -1,470 |  |  |
| GGH | gamma-glutamyl hydrolase (conjugase, folylpolygammaglutamyl hydrolase) |  |  | 6,55E-09 | -1,509 |  |  |
| GGNBP2 | gametogenetin binding protein 2 |  |  | 2,10E-10 | -1,743 |  |  |
| GINS2 | GINS complex subunit 2 (Psf2 homolog) |  |  | 1,14E-07 | -1,520 |  |  |
| GLB1 | galactosidase, beta 1 |  |  | 2,92E-09 | -1,602 |  |  |
| GLG1 | golgi apparatus protein 1 |  |  |  |  | 1,19E-13 | 1,317 |
| GLRX3 | glutaredoxin 3 |  |  | 2,69E-11 | -1,565 | 2,69E-11 | -1,425 |
| GLT25D1 | glycosyltransferase 25 domain containing 1 |  |  | 1,80E-09 | -1,867 |  |  |
| GLT8D1 | glycosyltransferase 8 domain containing 1 |  |  | 1,66E-08 | -1,686 |  |  |
| GLT8D3 | glycosyltransferase 8 domain containing 3 |  |  | 4,04E-09 | -1,738 |  |  |
| GLTSCR2 | glioma tumor suppressor candidate region gene 2 | 4,27E-10 | -1,620 | 4,27E-10 | -2,279 |  |  |
| GLUD2 | glutamate dehydrogenase 2 | 5,90E-10 | -1,341 | 5,90E-10 | -1,587 | 5,90E-10 | -1,641 |
| GLUL | glutamate-ammonia ligase (glutamine synthetase) |  |  | 3,30E-09 | -1,688 | 3,30E-09 | -1,649 |
| GLUL | glutamate-ammonia ligase (glutamine synthetase) |  |  | 4,79E-09 | -1,512 |  |  |
| GLUL | glutamate-ammonia ligase (glutamine synthetase) |  |  | 2,26E-13 | -1,790 | 2,26E-13 | -2,101 |
| GM2A | GM2 ganglioside activator |  |  | 7,67E-10 | -1,783 |  |  |
| GM2A | GM2 ganglioside activator |  |  | 1,17E-08 | -1,792 |  |  |
| GMFB | glia maturation factor, beta |  |  | 7,09E-11 | -1,730 |  |  |
| GMPR2 | guanosine monophosphate reductase 2 |  |  | 5,63E-09 | -1,835 |  |  |
| GNB1 | guanine nucleotide binding protein (G protein), beta polypeptide 1 |  |  | 9,66E-10 | -1,673 |  |  |
| GNB2 | guanine nucleotide binding protein (G protein), beta polypeptide 2 |  |  | 2,73E-11 | -1,875 |  |  |
| GNG5 | guanine nucleotide binding protein (G protein), gamma 5 |  |  | 1,35E-12 | -1,722 | 1,35E-12 | -1,447 |
| GNPDA1 (includes EG:10007) | glucosamine-6-phosphate deaminase 1 |  |  | 2,10E-08 | -1,683 |  |  |
| GNS | glucosamine (N-acetyl)-6-sulfatase |  |  | 2,30E-07 | -1,473 |  |  |
| GOLGA5 | golgi autoantigen, golgin subfamily a, 5 |  |  | 1,12E-07 | -1,537 |  |  |
| GOLGA7 | golgi autoantigen, golgin subfamily a, 7 |  |  | 1,69E-14 | -1,650 | 1,69E-14 | -1,921 |
| GOLT1B | golgi transport 1 homolog B (S. cerevisiae) | 2,74E-09 | -1,446 | 2,74E-09 | -1,505 | 2,74E-09 | -1,668 |
| GOT2 | glutamic-oxaloacetic transaminase 2, mitochondrial (aspartate aminotransferase 2) |  |  | 4,18E-08 | -1,569 |  |  |
| GPAA1 | glycosylphosphatidylinositol anchor attachment protein 1 homolog (yeast) |  |  | 3,15E-08 | -1,952 |  |  |
| GPAM | glycerol-3-phosphate acyltransferase, mitochondrial |  |  | 4,67E-10 | -1,709 |  |  |
| GPATCH8 | G patch domain containing 8 |  |  | 2,73E-08 | -1,736 |  |  |
| GPBP1 | GC-rich promoter binding protein 1 |  |  | 2,20E-12 | -1,618 |  |  |
| GPC3 | glypican 3 |  |  | 7,84E-08 | -1,539 |  |  |
| GPI | glucose phosphate isomerase |  |  | 1,95E-11 | -1,804 |  |  |
| GPN1 | GPN-loop GTPase 1 |  |  | 3,02E-10 | -1,802 |  |  |
| GPR107 | G protein-coupled receptor 107 |  |  | 1,69E-14 | -2,853 | 1,69E-14 | -1,843 |
| GPR126 | G protein-coupled receptor 126 |  |  |  |  | 7,65E-11 | 1,622 |
| GPR160 | G protein-coupled receptor 160 |  |  | 1,69E-14 | -1,698 | 1,69E-14 | -1,666 |
| GPR172A | G protein-coupled receptor 172A |  |  | 1,26E-11 | -2,403 |  |  |
| GPRC5B | G protein-coupled receptor, family C, group 5, member B |  |  | 6,12E-12 | -1,983 |  |  |
| GPS1 (includes EG:2873) | G protein pathway suppressor 1 |  |  | 2,77E-09 | -2,013 |  |  |
| GPSN2 | glycoprotein, synaptic 2 |  |  | 9,90E-08 | -1,597 |  |  |
| GPX3 | glutathione peroxidase 3 (plasma) |  |  | 5,01E-09 | -1,551 |  |  |
| GPX3 | glutathione peroxidase 3 (plasma) |  |  | 5,68E-11 | -1,745 |  |  |
| GREM1 | gremlin 1, cysteine knot superfamily, homolog (Xenopus laevis) |  |  |  |  | 3,01E-09 | 1,938 |
| GRHPR | glyoxylate reductase/hydroxypyruvate reductase |  |  | 9,98E-10 | -1,836 |  |  |
| GRN | granulin |  |  | 1,62E-09 | -1,877 |  |  |
| GRPEL1 | GrpE-like 1, mitochondrial (E. coli) |  |  | 8,06E-10 | -1,920 |  |  |
| GRSF1 | G-rich RNA sequence binding factor 1 |  |  | 3,81E-12 | -1,834 |  |  |
| GSPT1 | G1 to S phase transition 1 |  |  | 4,04E-09 | -1,593 |  |  |
| GSPT2 | G1 to S phase transition 2 |  |  | 6,31E-09 | -1,692 |  |  |
| GSS | glutathione synthetase |  |  | 6,97E-12 | -1,732 |  |  |
| GSS | glutathione synthetase |  |  | 1,69E-14 | -2,074 |  |  |
| GSTCD | glutathione S-transferase, C-terminal domain containing |  |  | 3,03E-10 | -2,030 | 3,03E-10 | -1,627 |
| GSTM1 | glutathione S-transferase mu 1 |  |  | 5,51E-09 | -1,874 |  |  |
| GSTM3 (includes EG:2947) | glutathione S-transferase mu 3 (brain) |  |  | 1,38E-08 | -1,598 |  |  |
| GSTM4 | glutathione S-transferase mu 4 |  |  | 1,86E-10 | -1,831 | 1,86E-10 | -1,809 |
| GSTO1 | glutathione S-transferase omega 1 |  |  | 2,56E-12 | -1,615 |  |  |
| GTF3A | general transcription factor IIIA |  |  | 8,94E-09 | -1,625 |  |  |
| GTF3A | general transcription factor IIIA |  |  | 8,20E-11 | -1,646 |  |  |
| GTF3C3 | general transcription factor IIIC, polypeptide 3, 102kDa |  |  | 4,69E-12 | -1,818 |  |  |
| GTPBP6 | GTP binding protein 6 (putative) |  |  | 1,10E-11 | -2,050 |  |  |
| GTSE1 | G-2 and S-phase expressed 1 |  |  |  |  | 3,14E-14 | 2,320 |
| GUF1 | GUF1 GTPase homolog (S. cerevisiae) |  |  | 6,34E-09 | -1,715 |  |  |
| GUSB | glucuronidase, beta |  |  | 1,01E-09 | -1,645 |  |  |
| GYG1 | glycogenin 1 |  |  | 3,24E-10 | -1,590 |  |  |
| GYG2 | glycogenin 2 |  |  | 8,47E-09 | -2,021 |  |  |
| GYS1 | glycogen synthase 1 (muscle) |  |  | 3,76E-08 | -2,066 |  |  |
| H1FX | H1 histone family, member X |  |  | 3,54E-11 | -1,915 |  |  |
| H2AFV (includes EG:94239) | H2A histone family, member V |  |  | 1,01E-11 | -1,783 | 1,01E-11 | -1,455 |
| H2AFV (includes EG:94239) | H2A histone family, member V |  |  | 5,22E-10 | -1,558 |  |  |
| H2AFV (includes EG:94239) | H2A histone family, member V |  |  | 3,45E-10 | -1,972 |  |  |
| H3F3A (includes EG:3020) | H3 histone, family 3A |  |  | 6,41E-09 | -1,496 |  |  |
| HADH | hydroxyacyl-Coenzyme A dehydrogenase |  |  | 2,94E-08 | -1,663 |  |  |
| HADH | hydroxyacyl-Coenzyme A dehydrogenase |  |  | 1,30E-11 | -1,804 |  |  |
| HADHA | hydroxyacyl-Coenzyme A dehydrogenase/3-ketoacyl-Coenzyme A thiolase/enoyl-Coenzyme A hydratase |  |  | 6,39E-08 | -1,624 |  |  |
| HAX1 | HCLS1 associated protein X-1 |  |  | 2,04E-09 | -1,580 |  |  |
| HBXIP | hepatitis B virus x interacting protein |  |  | 1,69E-14 | -1,841 | 1,69E-14 | -1,540 |
| HBXIP | hepatitis B virus x interacting protein |  |  | 2,69E-09 | -1,610 |  |  |
| HCCS | holocytochrome c synthase (cytochrome c heme-lyase) |  |  | 2,80E-09 | -1,779 |  |  |
| HCG 16001 | similar to ribosomal protein L23A |  |  | 1,69E-14 | 2,146 | 1,69E-14 | 1,845 |
| HCG 1815491 | hCG1815491 |  |  | 1,59E-11 | -1,957 |  |  |
| HCG 20857 | thiosulfate sulfurtransferase KAT, putative |  |  | 4,00E-08 | -1,819 |  |  |
| HDAC3 | histone deacetylase 3 |  |  | 1,08E-08 | -1,731 |  |  |
| HDDC2 | HD domain containing 2 |  |  | 2,49E-10 | -1,655 |  |  |
| HDGF | hepatoma-derived growth factor (high-mobility group protein 1-like) |  |  | 1,89E-09 | -1,674 |  |  |
| HDGFRP3 | hepatoma-derived growth factor, related protein 3 |  |  | 5,81E-11 | -1,695 | 5,81E-11 | -1,702 |
| HDHD1A | haloacid dehalogenase-like hydrolase domain containing 1A |  |  | 4,97E-08 | -1,634 |  |  |
| HDLBP | high density lipoprotein binding protein |  |  | 2,35E-09 | -1,983 |  |  |
| HEBP2 | heme binding protein 2 |  |  | 2,19E-08 | -1,598 |  |  |
| HECW1 | HECT, C2 and WW domain containing E3 ubiquitin protein ligase 1 |  |  | 1,69E-14 | -2,635 |  |  |
| HECW2 | HECT, C2 and WW domain containing E3 ubiquitin protein ligase 2 |  |  | 1,69E-14 | -1,561 | 1,69E-14 | -1,980 |
| HERPUD1 | homocysteine-inducible, endoplasmic reticulum stress-inducible, ubiquitin-like domain member 1 |  |  | 1,16E-09 | -1,540 |  |  |
| HES1 | hairy and enhancer of split 1, (Drosophila) | 1,48E-10 | -1,650 | 1,48E-10 | -1,467 |  |  |
| HEXA | hexosaminidase A (alpha polypeptide) |  |  | 9,51E-14 | -2,367 |  |  |
| HGD | homogentisate 1,2-dioxygenase (homogentisate oxidase) |  |  | 2,86E-08 | -1,897 |  |  |
| HGS | hepatocyte growth factor-regulated tyrosine kinase substrate |  |  | 6,81E-12 | -1,950 |  |  |
| HHEX | hematopoietically expressed homeobox |  |  | 5,60E-11 | -1,894 | 5,60E-11 | -1,908 |
| HIATL1 | hippocampus abundant transcript-like 1 |  |  | 3,23E-12 | -1,678 |  |  |
| HIBCH | 3-hydroxyisobutyryl-Coenzyme A hydrolase |  |  | 6,02E-10 | -1,620 |  |  |
| HIC2 | hypermethylated in cancer 2 |  |  | 1,02E-09 | -1,772 |  |  |
| HIF1AN | hypoxia inducible factor 1, alpha subunit inhibitor |  |  | 2,28E-10 | -1,875 |  |  |
| HINT1 (includes EG:3094) | histidine triad nucleotide binding protein 1 |  |  | 1,29E-10 | -1,599 |  |  |
| HINT2 | histidine triad nucleotide binding protein 2 |  |  | 1,51E-12 | -1,917 |  |  |
| HISPPD1 | histidine acid phosphatase domain containing 1 |  |  | 2,24E-09 | -1,542 |  |  |
| HIST1H2BK | histone cluster 1, H2bk |  |  | 2,44E-09 | -1,511 |  |  |
| HIST2H2AA3 | histone cluster 2, H2aa3 |  |  |  |  | 1,15E-09 | 1,643 |
| HKDC1 | hexokinase domain containing 1 |  |  | 2,98E-10 | -1,512 |  |  |
| HLA-B | major histocompatibility complex, class I, B |  |  |  |  | 4,29E-13 | -1,853 |
| HLA-B | major histocompatibility complex, class I, B |  |  | 3,49E-08 | -1,662 |  |  |
| HLA-B | major histocompatibility complex, class I, B |  |  | 3,36E-09 | -1,695 |  |  |
| HLA-B | major histocompatibility complex, class I, B |  |  | 2,60E-12 | -1,960 |  |  |
| HLA-B | major histocompatibility complex, class I, B |  |  | 1,69E-14 | -1,981 | 1,69E-14 | -1,449 |
| HLA-C | major histocompatibility complex, class I, C |  |  | 1,42E-12 | -1,907 |  |  |
| HLA-C | major histocompatibility complex, class I, C |  |  | 3,99E-09 | -2,139 |  |  |
| HLA-E | major histocompatibility complex, class I, E |  |  | 2,71E-11 | -1,896 |  |  |
| HLA-F | major histocompatibility complex, class I, F | 1,43E-13 | -1,696 | 1,43E-13 | -2,658 | 1,43E-13 | -1,738 |
| HLA-F | major histocompatibility complex, class I, F |  |  | 1,69E-14 | -2,390 | 1,69E-14 | -1,834 |
| HLA-G | major histocompatibility complex, class I, G |  |  | 2,89E-10 | -2,084 |  |  |
| HLA-G | major histocompatibility complex, class I, G |  |  | 9,37E-13 | -1,901 |  |  |
| HLA-G | major histocompatibility complex, class I, G |  |  | 4,20E-11 | -1,839 |  |  |
| HLA-G | major histocompatibility complex, class I, G |  |  | 1,39E-11 | -2,057 |  |  |
| HM13 | histocompatibility (minor) 13 |  |  | 1,73E-13 | -2,269 |  |  |
| HM13 | histocompatibility (minor) 13 |  |  | 7,16E-10 | -2,027 |  |  |
| HMGA1 | high mobility group AT-hook 1 |  |  | 2,53E-13 | -2,051 |  |  |
| HMGB2 | high-mobility group box 2 |  |  | 1,69E-14 | -1,488 | 1,69E-14 | -1,578 |
| HMGCR | 3-hydroxy-3-methylglutaryl-Coenzyme A reductase |  |  | 1,49E-08 | -1,487 |  |  |
| HMGN4 | high mobility group nucleosomal binding domain 4 |  |  | 1,49E-07 | -1,548 |  |  |
| HMGN4 | high mobility group nucleosomal binding domain 4 |  |  | 1,23E-08 | -1,641 |  |  |
| HMMR | hyaluronan-mediated motility receptor (RHAMM) |  |  | 1,77E-12 | -1,633 | 1,77E-12 | -1,509 |
| HMOX2 | heme oxygenase (decycling) 2 |  |  | 1,95E-13 | -1,998 | 1,95E-13 | -1,488 |
| HN1 | hematological and neurological expressed 1 |  |  | 7,16E-10 | -1,894 |  |  |
| HNMT | histamine N-methyltransferase |  |  | 5,57E-10 | -1,534 | 5,57E-10 | -1,471 |
| HNRNPA0 | heterogeneous nuclear ribonucleoprotein A0 |  |  | 8,44E-08 | -1,454 |  |  |
| HNRNPA0 | heterogeneous nuclear ribonucleoprotein A0 | 1,63E-12 | 1,991 |  |  |  |  |
| HNRNPA1 | heterogeneous nuclear ribonucleoprotein A1 | 1,69E-14 | -2,639 | 1,69E-14 | -4,084 | 1,69E-14 | -3,958 |
| HNRNPA2B1 | heterogeneous nuclear ribonucleoprotein A2/B1 |  |  | 2,91E-09 | -1,489 |  |  |
| HNRNPA3 | heterogeneous nuclear ribonucleoprotein A3 | 3,16E-10 | -1,712 | 3,16E-10 | -1,738 |  |  |
| HNRNPA3 | heterogeneous nuclear ribonucleoprotein A3 |  |  | 5,47E-12 | -1,618 |  |  |
| HNRNPC | heterogeneous nuclear ribonucleoprotein C (C1/C2) |  |  | 2,53E-10 | -1,674 |  |  |
| HNRNPC | heterogeneous nuclear ribonucleoprotein C (C1/C2) |  |  | 1,50E-08 | -1,519 |  |  |
| HNRNPC | heterogeneous nuclear ribonucleoprotein C (C1/C2) |  |  | 4,35E-08 | -1,493 |  |  |
| HNRNPD | heterogeneous nuclear ribonucleoprotein D (AU-rich element RNA binding protein 1, 37kDa) |  |  | 1,69E-14 | -1,922 |  |  |
| HNRNPF | heterogeneous nuclear ribonucleoprotein F |  |  | 1,69E-09 | -1,583 | 1,69E-09 | -1,457 |
| HNRNPH1 | heterogeneous nuclear ribonucleoprotein H1 (H) |  |  | 1,69E-14 | -1,365 | 1,69E-14 | -1,564 |
| HNRNPH3 | heterogeneous nuclear ribonucleoprotein H3 (2H9) |  |  | 1,59E-08 | -1,448 |  |  |
| HNRNPK | heterogeneous nuclear ribonucleoprotein K |  |  | 2,39E-10 | -1,605 |  |  |
| HNRNPM | heterogeneous nuclear ribonucleoprotein M |  |  | 3,17E-08 | -1,503 |  |  |
| HNRNPR | heterogeneous nuclear ribonucleoprotein R |  |  | 5,39E-10 | -1,498 |  |  |
| HNRNPUL1 | heterogeneous nuclear ribonucleoprotein U-like 1 |  |  | 1,69E-14 | -2,158 |  |  |
| HOOK1 | hook homolog 1 (Drosophila) |  |  |  |  | 3,49E-08 | 1,515 |
| HOOK1 | hook homolog 1 (Drosophila) |  |  | 2,22E-09 | -1,613 |  |  |
| HOXB7 | homeobox B7 |  |  | 6,82E-12 | -1,787 |  |  |
| HOXB7 | homeobox B7 |  |  | 9,28E-11 | -1,786 |  |  |
| HOXC6 | homeobox C6 |  |  | 1,80E-09 | -1,686 |  |  |
| HP1BP3 | heterochromatin protein 1, binding protein 3 |  |  | 5,24E-08 | -1,543 |  |  |
| HPDL | 4-hydroxyphenylpyruvate dioxygenase-like |  |  | 7,33E-13 | -2,008 | 7,33E-13 | -1,608 |
| HPGD | hydroxyprostaglandin dehydrogenase 15-(NAD) |  |  | 2,39E-09 | -1,570 |  |  |
| HPGD | hydroxyprostaglandin dehydrogenase 15-(NAD) |  |  | 1,77E-12 | -1,728 | 1,77E-12 | -1,826 |
| HRAS | v-Ha-ras Harvey rat sarcoma viral oncogene homolog |  |  | 2,94E-11 | -2,133 |  |  |
| HRSP12 | heat-responsive protein 12 |  |  | 1,69E-14 | -1,913 | 1,69E-14 | -1,475 |
| HS2ST1 | heparan sulfate 2-O-sulfotransferase 1 |  |  | 1,55E-09 | -1,524 |  |  |
| HSBP1 | heat shock factor binding protein 1 |  |  | 1,30E-10 | -1,670 |  |  |
| HSD17B10 | hydroxysteroid (17-beta) dehydrogenase 10 |  |  | 9,88E-11 | -1,710 |  |  |
| HSDL2 | hydroxysteroid dehydrogenase like 2 |  |  | 1,07E-10 | -1,713 |  |  |
| HSP90AB1 | heat shock protein 90kDa alpha (cytosolic), class B member 1 | 7,46E-12 | -1,402 | 7,46E-12 | -1,455 | 7,46E-12 | -1,542 |
| HSP90AB1 | heat shock protein 90kDa alpha (cytosolic), class B member 1 |  |  | 8,47E-09 | -1,707 |  |  |
| HSPA4 | heat shock 70kDa protein 4 |  |  | 6,05E-10 | -1,595 | 6,05E-10 | -1,650 |
| HSPA5 | heat shock 70kDa protein 5 (glucose-regulated protein, 78kDa) |  |  | 1,34E-11 | -1,588 | 1,34E-11 | -1,391 |
| HSPA9 | heat shock 70kDa protein 9 (mortalin) |  |  | 6,18E-10 | -1,850 |  |  |
| HSPB11 | heat shock protein family B (small), member 11 |  |  | 2,52E-11 | -1,794 | 2,52E-11 | -1,552 |
| HSPB11 | heat shock protein family B (small), member 11 |  |  | 9,82E-11 | -1,664 |  |  |
| HSPB8 | heat shock 22kDa protein 8 |  |  | 6,52E-09 | -1,713 |  |  |
| HSPG2 (includes EG:3339) | heparan sulfate proteoglycan 2 |  |  | 8,11E-09 | -2,106 |  |  |
| HTATSF1 | HIV-1 Tat specific factor 1 |  |  | 7,07E-14 | -1,835 | 7,07E-14 | -1,489 |
| HTRA2 | HtrA serine peptidase 2 |  |  | 2,45E-08 | -1,728 |  |  |
| HUWE1 | HECT, UBA and WWE domain containing 1 |  |  | 3,02E-08 | -1,583 |  |  |
| HYAL2 | hyaluronoglucosaminidase 2 |  |  | 7,46E-12 | -2,214 |  |  |
| IAH1 | isoamyl acetate-hydrolyzing esterase 1 homolog (S. cerevisiae) |  |  | 1,69E-14 | -1,793 | 1,69E-14 | -2,081 |
| IARS2 | isoleucyl-tRNA synthetase 2, mitochondrial |  |  | 8,63E-12 | -1,708 |  |  |
| ID2 | inhibitor of DNA binding 2, dominant negative helix-loop-helix protein |  |  | 1,07E-10 | -1,682 |  |  |
| IDE | insulin-degrading enzyme |  |  | 1,19E-11 | -1,740 |  |  |
| IDE | insulin-degrading enzyme |  |  | 1,69E-14 | -1,933 | 1,69E-14 | -1,799 |
| IDE | insulin-degrading enzyme |  |  | 9,64E-10 | -1,614 | 9,64E-10 | -1,462 |
| IDH1 | isocitrate dehydrogenase 1 (NADP+), soluble |  |  | 1,02E-11 | -1,466 | 1,02E-11 | -1,440 |
| IDH3B | isocitrate dehydrogenase 3 (NAD+) beta |  |  | 9,16E-10 | -1,761 |  |  |
| IDH3B | isocitrate dehydrogenase 3 (NAD+) beta |  |  | 2,29E-12 | -1,940 |  |  |
| IDH3B | isocitrate dehydrogenase 3 (NAD+) beta |  |  | 2,22E-11 | -1,907 |  |  |
| IDH3G | isocitrate dehydrogenase 3 (NAD+) gamma |  |  | 7,70E-10 | -1,831 |  |  |
| IDS | iduronate 2-sulfatase |  |  |  |  | 1,93E-06 | 1,468 |
| IER3IP1 | immediate early response 3 interacting protein 1 |  |  | 3,94E-08 | -1,519 |  |  |
| IFNGR1 | interferon gamma receptor 1 |  |  |  |  | 1,69E-14 | 2,727 |
| IFNGR2 | interferon gamma receptor 2 (interferon gamma transducer 1) |  |  | 1,52E-09 | -1,679 |  |  |
| IFT52 | intraflagellar transport 52 homolog (Chlamydomonas) |  |  | 1,68E-09 | -1,618 |  |  |
| IFT52 | intraflagellar transport 52 homolog (Chlamydomonas) |  |  | 2,99E-12 | -2,493 |  |  |
| IFT80 | intraflagellar transport 80 homolog (Chlamydomonas) |  |  | 6,45E-09 | -1,472 |  |  |
| IGF2 | insulin-like growth factor 2 (somatomedin A) |  |  | 1,69E-14 | -2,330 | 1,69E-14 | -1,435 |
| IGF2BP1 | insulin-like growth factor 2 mRNA binding protein 1 |  |  | 1,69E-14 | -1,925 |  |  |
| IGF2BP3 | insulin-like growth factor 2 mRNA binding protein 3 |  |  | 3,65E-12 | -1,569 | 3,65E-12 | -1,374 |
| IGF2R | insulin-like growth factor 2 receptor |  |  | 3,93E-09 | -1,815 |  |  |
| IGF2R | insulin-like growth factor 2 receptor |  |  | 1,27E-08 | -1,585 |  |  |
| IGFBP6 | insulin-like growth factor binding protein 6 |  |  | 5,80E-08 | -2,253 |  |  |
| IGFBP7 | insulin-like growth factor binding protein 7 |  |  |  |  | 1,98E-10 | -2,153 |
| IHH | Indian hedgehog homolog (Drosophila) |  |  | 9,32E-13 | -2,593 |  |  |
| IKIP | IKK interacting protein |  |  | 1,84E-10 | -2,141 |  |  |
| IL13RA1 | interleukin 13 receptor, alpha 1 |  |  | 1,01E-10 | -1,714 | 1,01E-10 | -1,567 |
| IL6ST | interleukin 6 signal transducer (gp130, oncostatin M receptor) |  |  |  |  | 1,69E-14 | 6,456 |
| IL6ST | interleukin 6 signal transducer (gp130, oncostatin M receptor) | 1,69E-14 | 2,028 | 1,69E-14 | 2,502 | 1,69E-14 | 4,022 |
| ILF2 (includes EG:3608) | interleukin enhancer binding factor 2, 45kDa |  |  | 4,43E-10 | -1,590 |  |  |
| ILF3 | interleukin enhancer binding factor 3, 90kDa |  |  | 1,69E-14 | -1,829 |  |  |
| ILVBL | ilvB (bacterial acetolactate synthase)-like |  |  | 7,21E-08 | -2,256 |  |  |
| ILVBL | ilvB (bacterial acetolactate synthase)-like |  |  | 7,83E-10 | -2,025 |  |  |
| IMMP1L | IMP1 inner mitochondrial membrane peptidase-like (S. cerevisiae) |  |  | 9,24E-08 | -1,661 |  |  |
| IMP4 | IMP4, U3 small nucleolar ribonucleoprotein, homolog (yeast) |  |  | 1,03E-12 | -1,918 |  |  |
| INO80 | INO80 homolog (S. cerevisiae) |  |  | 1,98E-09 | -2,358 |  |  |
| INO80 | INO80 homolog (S. cerevisiae) |  |  | 2,07E-09 | -1,847 |  |  |
| INO80E | INO80 complex subunit E |  |  | 4,09E-11 | -2,271 |  |  |
| INPP5A | inositol polyphosphate-5-phosphatase, 40kDa |  |  | 2,04E-10 | -1,702 |  |  |
| INTS3 | integrator complex subunit 3 |  |  | 4,07E-10 | -1,980 |  |  |
| INTS4 | integrator complex subunit 4 |  |  | 1,23E-11 | -2,043 | 1,23E-11 | -2,088 |
| INTS4 | integrator complex subunit 4 | 4,75E-09 | -1,993 |  |  |  |  |
| INTS5 | integrator complex subunit 5 |  |  | 1,75E-09 | -1,691 |  |  |
| IPO11 | importin 11 |  |  | 1,62E-06 | -1,613 |  |  |
| IPO5 | importin 5 |  |  | 1,15E-11 | -1,583 |  |  |
| IPO7 | importin 7 |  |  | 4,39E-10 | -1,462 |  |  |
| IPO7 | importin 7 |  |  | 5,90E-11 | -1,495 |  |  |
| IPO7 | importin 7 |  |  | 4,24E-11 | -1,549 |  |  |
| IPO8 | importin 8 |  |  | 4,97E-12 | -1,840 |  |  |
| IQGAP1 | IQ motif containing GTPase activating protein 1 |  |  | 2,32E-12 | -1,831 |  |  |
| IQGAP1 | IQ motif containing GTPase activating protein 1 |  |  |  |  | 1,69E-14 | 2,419 |
| IQGAP2 | IQ motif containing GTPase activating protein 2 |  |  | 4,97E-12 | -1,771 | 4,97E-12 | -1,525 |
| IQWD1 | IQ motif and WD repeats 1 |  |  | 1,69E-14 | -1,929 | 1,69E-14 | -1,477 |
| IREB2 | iron-responsive element binding protein 2 |  |  | 7,39E-11 | -1,625 |  |  |
| IRF2 | interferon regulatory factor 2 |  |  | 5,71E-13 | -2,543 |  |  |
| IRF2BP2 | interferon regulatory factor 2 binding protein 2 |  |  | 2,19E-10 | -1,805 |  |  |
| ISCA1 | iron-sulfur cluster assembly 1 homolog (S. cerevisiae) |  |  |  |  | 8,47E-09 | -1,814 |
| ISCA2 | iron-sulfur cluster assembly 2 homolog (S. cerevisiae) | 1,69E-12 | -1,359 | 1,69E-12 | -1,775 | 1,69E-12 | -1,505 |
| ISOC1 | isochorismatase domain containing 1 |  |  | 5,79E-10 | -1,480 |  |  |
| ISOC2 | isochorismatase domain containing 2 |  |  | 3,87E-09 | -2,420 |  |  |
| ISX | intestine-specific homeobox |  |  | 5,69E-10 | -2,471 | 5,69E-10 | -2,092 |
| ITFG3 | integrin alpha FG-GAP repeat containing 3 |  |  | 4,82E-11 | -2,253 |  |  |
| ITGAE | integrin, alpha E (antigen CD103, human mucosal lymphocyte antigen 1; alpha polypeptide) |  |  | 4,43E-11 | -1,729 |  |  |
| ITGAV | integrin, alpha V (vitronectin receptor, alpha polypeptide, antigen CD51) |  |  | 5,59E-07 | -1,442 |  |  |
| ITGB1 | integrin, beta 1 (fibronectin receptor, beta polypeptide, antigen CD29 includes MDF2, MSK12) |  |  | 1,49E-12 | -1,676 | 1,49E-12 | -1,414 |
| ITGB1 | integrin, beta 1 (fibronectin receptor, beta polypeptide, antigen CD29 includes MDF2, MSK12) |  |  | 3,05E-09 | -1,459 | 3,05E-09 | -1,377 |
| ITGB1 | integrin, beta 1 (fibronectin receptor, beta polypeptide, antigen CD29 includes MDF2, MSK12) |  |  | 3,42E-08 | 2,891 |  |  |
| ITGB3BP | integrin beta 3 binding protein (beta3-endonexin) |  |  | 4,17E-10 | -1,619 |  |  |
| ITM2B | integral membrane protein 2B |  |  | 1,84E-10 | -1,527 |  |  |
| ITM2C | integral membrane protein 2C |  |  | 7,35E-11 | -2,027 |  |  |
| ITPA | inosine triphosphatase (nucleoside triphosphate pyrophosphatase) |  |  | 1,34E-12 | -1,984 |  |  |
| ITPR3 | inositol 1,4,5-triphosphate receptor, type 3 |  |  | 1,42E-11 | -2,012 |  |  |
| ITSN1 | intersectin 1 (SH3 domain protein) |  |  | 2,44E-09 | -1,627 |  |  |
| ITSN1 | intersectin 1 (SH3 domain protein) |  |  | 7,65E-09 | -1,694 |  |  |
| IVD | isovaleryl Coenzyme A dehydrogenase |  |  | 6,89E-09 | -1,867 |  |  |
| IVNS1ABP | influenza virus NS1A binding protein |  |  | 1,08E-07 | -1,608 |  |  |
| JAG1 | jagged 1 (Alagille syndrome) |  |  | 1,26E-08 | -1,404 |  |  |
| JAGN1 | jagunal homolog 1 (Drosophila) |  |  | 6,53E-12 | -1,785 |  |  |
| JAK1 | Janus kinase 1 (a protein tyrosine kinase) |  |  | 9,49E-10 | -1,702 | 9,49E-10 | -1,879 |
| JAK1 | Janus kinase 1 (a protein tyrosine kinase) |  |  |  |  | 1,43E-12 | 2,471 |
| JAK1 | Janus kinase 1 (a protein tyrosine kinase) |  |  | 5,75E-09 | -1,657 |  |  |
| JDP2 | Jun dimerization protein 2 |  |  | 3,13E-10 | -2,448 |  |  |
| JTB | jumping translocation breakpoint |  |  | 8,35E-10 | -1,549 |  |  |
| JUB | jub, ajuba homolog (Xenopus laevis) |  |  | 6,04E-13 | -1,475 | 6,04E-13 | -1,746 |
| JUND | jun D proto-oncogene |  |  | 2,49E-08 | -1,574 |  |  |
| JUP | junction plakoglobin |  |  | 6,07E-11 | -1,706 |  |  |
| KANK2 | KN motif and ankyrin repeat domains 2 |  |  | 1,69E-14 | -2,179 | 1,69E-14 | -1,665 |
| KCNE3 | potassium voltage-gated channel, Isk-related family, member 3 |  |  | 1,56E-10 | -1,914 |  |  |
| KCTD14 | potassium channel tetramerisation domain containing 14 |  |  | 1,92E-08 | -1,662 |  |  |
| KCTD2 | potassium channel tetramerisation domain containing 2 |  |  | 5,46E-09 | -2,022 |  |  |
| KCTD5 | potassium channel tetramerisation domain containing 5 |  |  | 2,79E-08 | -1,657 |  |  |
| KCTD5 | potassium channel tetramerisation domain containing 5 | 4,52E-10 | -2,022 | 4,52E-10 | -2,341 | 4,52E-10 | -1,646 |
| KCTD9 | potassium channel tetramerisation domain containing 9 |  |  | 1,55E-11 | -1,621 |  |  |
| KHSRP | KH-type splicing regulatory protein |  |  | 3,86E-09 | -1,654 |  |  |
| KIAA0141 | KIAA0141 |  |  | 1,16E-07 | -1,921 |  |  |
| KIAA0368 | KIAA0368 |  |  | 3,44E-10 | -1,628 |  |  |
| KIAA0368 | KIAA0368 |  |  |  |  | 3,54E-11 | 1,867 |
| KIAA0406 | KIAA0406 |  |  | 1,94E-11 | -1,849 |  |  |
| KIAA0652 | KIAA0652 |  |  | 1,65E-07 | -2,105 |  |  |
| KIAA0652 | KIAA0652 |  |  | 4,32E-09 | -1,852 |  |  |
| KIAA0776 | KIAA0776 |  |  | 8,57E-13 | -1,680 | 8,57E-13 | -1,595 |
| KIAA0859 | KIAA0859 |  |  | 3,18E-09 | -2,416 |  |  |
| KIAA0922 | KIAA0922 |  |  | 7,79E-08 | -1,927 |  |  |
| KIAA0999 | KIAA0999 protein |  |  | 3,03E-08 | -1,806 |  |  |
| KIAA1191 | KIAA1191 |  |  | 2,87E-12 | -1,641 |  |  |
| KIAA1219 | KIAA1219 |  |  | 3,88E-09 | -1,688 |  |  |
| KIAA1333 | KIAA1333 |  |  | 1,37E-10 | -2,044 |  |  |
| KIAA1524 | KIAA1524 |  |  | 1,54E-09 | -1,730 |  |  |
| KIAA1627 | KIAA1627 protein | 4,25E-12 | -1,432 | 4,25E-12 | -2,207 | 4,25E-12 | -1,750 |
| KIAA1737 | KIAA1737 |  |  | 4,78E-10 | -1,910 |  |  |
| KIAA2013 | KIAA2013 | 5,58E-12 | -1,526 | 5,58E-12 | -2,273 |  |  |
| KIF11 | kinesin family member 11 |  |  | 3,32E-12 | -1,609 | 3,32E-12 | -1,365 |
| KIF14 | kinesin family member 14 |  |  | 5,77E-14 | -1,998 | 5,77E-14 | -1,522 |
| KIF15 | kinesin family member 15 |  |  | 8,03E-11 | -1,770 |  |  |
| KIF20B | kinesin family member 20B |  |  | 6,14E-10 | -1,650 |  |  |
| KIF21A | kinesin family member 21A |  |  |  |  | 4,49E-14 | 2,104 |
| KIF22 | kinesin family member 22 |  |  | 3,41E-12 | -1,860 |  |  |
| KIF22 | kinesin family member 22 |  |  |  |  | 2,06E-08 | -2,069 |
| KIF2C | kinesin family member 2C |  |  | 1,12E-09 | -1,866 |  |  |
| KIF3A | kinesin family member 3A |  |  | 1,69E-14 | -2,040 | 1,69E-14 | -1,479 |
| KIF3B | kinesin family member 3B |  |  | 5,26E-11 | -1,699 |  |  |
| KIF3B | kinesin family member 3B |  |  | 1,48E-11 | -1,856 |  |  |
| KIF4A | kinesin family member 4A |  |  | 3,41E-12 | -1,764 |  |  |
| KIF5B | kinesin family member 5B |  |  | 9,85E-12 | -1,671 |  |  |
| KIN | KIN, antigenic determinant of recA protein homolog (mouse) |  |  | 1,13E-10 | -1,762 |  |  |
| KITLG | KIT ligand | 1,69E-14 | -1,405 | 1,69E-14 | -1,727 | 1,69E-14 | -2,407 |
| KITLG | KIT ligand |  |  | 1,69E-14 | -1,997 | 1,69E-14 | -2,727 |
| KLC2 | kinesin light chain 2 |  |  | 3,61E-10 | -3,657 |  |  |
| KLF10 | Kruppel-like factor 10 |  |  | 1,38E-11 | -1,530 |  |  |
| KLF6 | Kruppel-like factor 6 |  |  |  |  | 1,69E-14 | 1,520 |
| KLF6 | Kruppel-like factor 6 |  |  |  |  | 5,77E-14 | 1,699 |
| KLF6 | Kruppel-like factor 6 |  |  |  |  | 5,47E-12 | 1,565 |
| KLHDC2 | kelch domain containing 2 |  |  | 2,12E-10 | -1,653 |  |  |
| KLHDC3 | kelch domain containing 3 | 3,32E-12 | -1,775 | 3,32E-12 | -2,512 |  |  |
| KLHL12 | kelch-like 12 (Drosophila) |  |  | 1,77E-11 | -1,820 |  |  |
| KLHL15 | kelch-like 15 (Drosophila) |  |  | 1,68E-08 | -1,809 |  |  |
| KLHL20 | kelch-like 20 (Drosophila) | 1,75E-06 | -1,776 |  |  |  |  |
| KLHL5 | kelch-like 5 (Drosophila) |  |  | 3,76E-08 | -1,569 |  |  |
| KLHL7 | kelch-like 7 (Drosophila) |  |  | 3,14E-14 | -1,959 | 3,14E-14 | -1,749 |
| KLHL7 | kelch-like 7 (Drosophila) |  |  | 2,26E-13 | -1,809 | 2,26E-13 | -1,441 |
| KLHL8 | kelch-like 8 (Drosophila) |  |  | 3,81E-09 | -1,618 |  |  |
| KLK6 | kallikrein-related peptidase 6 |  |  | 9,51E-14 | -2,103 | 9,51E-14 | -1,693 |
| KPNA3 | karyopherin alpha 3 (importin alpha 4) |  |  | 1,37E-11 | -1,679 |  |  |
| KPNA4 | karyopherin alpha 4 (importin alpha 3) |  |  | 2,97E-09 | -1,602 |  |  |
| KPNA6 | karyopherin alpha 6 (importin alpha 7) |  |  | 1,66E-09 | -1,909 |  |  |
| KPNB1 | karyopherin (importin) beta 1 |  |  | 7,14E-10 | -1,636 |  |  |
| KPNB1 | karyopherin (importin) beta 1 |  |  | 2,41E-09 | -1,561 |  |  |
| KRAS | v-Ki-ras2 Kirsten rat sarcoma viral oncogene homolog |  |  | 1,72E-09 | -1,519 |  |  |
| KRCC1 | lysine-rich coiled-coil 1 |  |  | 2,14E-07 | -1,812 |  |  |
| KRT10 | keratin 10 |  |  | 1,80E-10 | -1,678 | 1,80E-10 | -1,500 |
| KRT10 | keratin 10 |  |  | 1,69E-14 | -1,931 | 1,69E-14 | -1,725 |
| KRTCAP2 | keratinocyte associated protein 2 |  |  | 5,06E-11 | -1,622 |  |  |
| L1CAM | L1 cell adhesion molecule |  |  |  |  | 1,07E-11 | 1,706 |
| LACTB2 | lactamase, beta 2 |  |  | 1,69E-14 | -1,789 | 1,69E-14 | -1,652 |
| LAD1 | ladinin 1 |  |  | 1,44E-08 | -3,134 |  |  |
| LAGE3 | L antigen family, member 3 |  |  | 4,84E-09 | -1,829 |  |  |
| LAMB1 | laminin, beta 1 |  |  | 1,81E-11 | -1,650 |  |  |
| LAMB3 | laminin, beta 3 |  |  | 1,69E-14 | -2,668 | 1,69E-14 | -1,450 |
| LAMP1 | lysosomal-associated membrane protein 1 |  |  | 1,04E-07 | -1,554 |  |  |
| LAMP2 | lysosomal-associated membrane protein 2 |  |  | 9,08E-09 | -1,523 |  |  |
| LAP3 | leucine aminopeptidase 3 |  |  | 4,05E-08 | -1,517 |  |  |
| LAPTM4B | lysosomal protein transmembrane 4 beta |  |  | 1,69E-14 | -1,729 | 1,69E-14 | -2,027 |
| LAPTM4B | lysosomal protein transmembrane 4 beta |  |  | 7,12E-11 | -1,658 |  |  |
| LAPTM4B | lysosomal protein transmembrane 4 beta |  |  | 3,30E-12 | -1,585 | 3,30E-12 | -1,533 |
| LAPTM4B | lysosomal protein transmembrane 4 beta |  |  | 1,51E-08 | -1,431 |  |  |
| LARP1 | La ribonucleoprotein domain family, member 1 |  |  | 1,56E-08 | -1,614 |  |  |
| LARP1 | La ribonucleoprotein domain family, member 1 |  |  | 1,69E-14 | -1,959 |  |  |
| LARP5 | La ribonucleoprotein domain family, member 5 |  |  | 3,05E-08 | -1,634 |  |  |
| LARP5 | La ribonucleoprotein domain family, member 5 | 3,92E-12 | -1,682 | 3,92E-12 | -1,861 | 3,92E-12 | -2,968 |
| LARP6 | La ribonucleoprotein domain family, member 6 |  |  | 4,15E-11 | -2,151 |  |  |
| LARP7 (includes EG:51574) | La ribonucleoprotein domain family, member 7 |  |  | 1,30E-08 | -1,775 |  |  |
| LARS2 | leucyl-tRNA synthetase 2, mitochondrial |  |  | 7,67E-08 | -1,459 |  |  |
| LASS2 | LAG1 homolog, ceramide synthase 2 |  |  | 1,69E-14 | -1,871 | 1,69E-14 | -1,484 |
| LASS5 | LAG1 homolog, ceramide synthase 5 |  |  | 5,64E-13 | -2,078 |  |  |
| LBH | limb bud and heart development homolog (mouse) |  |  | 1,07E-07 | -1,546 |  |  |
| LCMT1 | leucine carboxyl methyltransferase 1 |  |  | 1,43E-09 | -1,814 |  |  |
| LEPROTL1 | leptin receptor overlapping transcript-like 1 | 7,35E-12 | -1,356 | 7,35E-12 | -1,682 | 7,35E-12 | -1,616 |
| LETMD1 | LETM1 domain containing 1 |  |  | 1,76E-07 | -1,644 |  |  |
| LGALS2 | lectin, galactoside-binding, soluble, 2 |  |  | 8,35E-14 | -1,975 |  |  |
| LGALS3BP | lectin, galactoside-binding, soluble, 3 binding protein | 1,69E-14 | -1,701 | 1,69E-14 | -2,673 | 1,69E-14 | -1,625 |
| LGMN | legumain |  |  | 3,05E-08 | -1,903 |  |  |
| LIMA1 | LIM domain and actin binding 1 |  |  | 1,40E-10 | -1,623 |  |  |
| LIMA1 | LIM domain and actin binding 1 |  |  |  |  | 1,69E-14 | 1,467 |
| LIMK2 | LIM domain kinase 2 |  |  | 4,03E-08 | -1,984 |  |  |
| LIN28B | lin-28 homolog B (C. elegans) |  |  | 3,28E-10 | -1,602 |  |  |
| LIPA | lipase A, lysosomal acid, cholesterol esterase |  |  | 7,34E-12 | -1,611 | 7,34E-12 | -1,491 |
| LIPG | lipase, endothelial |  |  | 4,21E-09 | -1,613 |  |  |
| LITAF | lipopolysaccharide-induced TNF factor |  |  | 3,34E-12 | -1,949 |  |  |
| LLGL1 | lethal giant larvae homolog 1 (Drosophila) |  |  | 1,62E-11 | -2,116 |  |  |
| LMAN2 | lectin, mannose-binding 2 |  |  | 7,08E-08 | -1,602 |  |  |
| LMCD1 | LIM and cysteine-rich domains 1 |  |  |  |  | 2,48E-10 | 1,765 |
| LMNA | lamin A/C |  |  | 6,59E-10 | -1,865 |  |  |
| LMO4 | LIM domain only 4 |  |  |  |  | 1,58E-06 | 1,636 |
| LMO7 | LIM domain 7 |  |  |  |  | 2,36E-13 | 1,913 |
| LOC100129361 | hypothetical protein LOC100129361 |  |  | 4,76E-09 | -1,559 |  |  |
| LOC100130506 | hypothetical protein LOC100130506 |  |  | 4,96E-09 | -1,943 |  |  |
| LOC100131402 | hypothetical protein LOC100131402 |  |  |  |  | 2,26E-13 | 3,481 |
| LOC100132181 | hypothetical protein LOC100132181 |  |  | 4,19E-08 | -1,525 |  |  |
| LOC100132181 | hypothetical protein LOC100132181 |  |  | 1,82E-09 | -1,491 | 1,82E-09 | -1,615 |
| LOC100132815 | hypothetical protein LOC100132815 |  |  | 2,30E-09 | -2,091 |  |  |
| LOC100132910 | PRO1477 |  |  | 7,06E-09 | -1,873 |  |  |
| LOC146346 | hypothetical protein LOC146346 |  |  |  |  | 1,69E-14 | 1,604 |
| LOC158402 | hypothetical protein LOC158402 |  |  | 1,49E-08 | -1,732 |  |  |
| LOC203547 | hypothetical protein LOC203547 |  |  | 4,12E-09 | -1,690 |  |  |
| LOC221710 | hypothetical protein LOC221710 |  |  | 9,28E-08 | -2,388 |  |  |
| LOC23117 | PI-3-kinase-related kinase SMG-1 isoform 1 homolog |  |  |  |  | 1,69E-14 | 1,900 |
| LOC254057 | hypothetical protein LOC254057 |  |  | 7,83E-09 | -2,398 |  |  |
| LOC26010 | viral DNA polymerase-transactivated protein 6 |  |  | 4,31E-09 | -1,505 |  |  |
| LOC284422 | similar to HSPC323 |  |  | 5,64E-13 | -2,009 |  |  |
| LOC285074 | hypothetical protein LOC285074 |  |  | 1,44E-07 | -1,663 |  |  |
| LOC285733 | hypothetical LOC285733 |  |  | 1,80E-10 | -1,804 | 1,80E-10 | -1,646 |
| LOC387882 | overexpressed in colon carcinoma-1 |  |  | 1,84E-10 | -1,530 |  |  |
| LOC388789 | hypothetical LOC388789 |  |  | 3,59E-12 | -1,617 |  |  |
| LOC388796 | hypothetical LOC388796 |  |  | 3,14E-14 | -1,694 |  |  |
| LOC400027 | hypothetical gene supported by BC047417 |  |  | 3,00E-09 | -1,535 | 3,00E-09 | -1,618 |
| LOC401152 | chromosome 4 open reading frame 3 |  |  | 4,22E-09 | -1,633 |  |  |
| LOC401152 | chromosome 4 open reading frame 3 |  |  | 1,15E-07 | -1,590 |  |  |
| LOC440895 | LIM and senescent cell antigen-like domains 3-like |  |  | 2,78E-09 | -1,616 |  |  |
| LOC440995 | hypothetical gene supported by BC034933; BC068085 | 1,69E-14 | -1,612 | 1,69E-14 | -2,281 |  |  |
| LOC441453 | similar to olfactory receptor, family 7, subfamily A, member 17 |  |  | 1,12E-08 | -2,417 |  |  |
| LOC442175 | similar to hCG1811681 | 1,69E-14 | -1,808 | 1,69E-14 | -3,256 | 1,69E-14 | -2,954 |
| LOC550643 | hypothetical LOC550643 |  |  | 4,47E-09 | -1,731 |  |  |
| LOC552889 | hypothetical protein LOC552889 |  |  | 1,56E-09 | -1,743 |  |  |
| LOC57228 | small trans-membrane and glycosylated protein |  |  | 4,03E-13 | -2,151 |  |  |
| LOC654433 | hypothetical LOC654433 |  |  | 3,68E-10 | -1,630 |  |  |
| LOC728449 | hypothetical protein LOC728449 | 1,69E-14 | -2,888 | 1,69E-14 | -5,156 | 1,69E-14 | -3,406 |
| LOC728453 | similar to 40S ribosomal protein S28 |  |  | 3,40E-13 | -1,928 | 3,40E-13 | -1,621 |
| LOC729580 | hypothetical LOC729580 |  |  | 3,38E-09 | -2,324 |  |  |
| LOC729659 | S100 calcium binding protein A11 pseudogene |  |  | 1,69E-14 | -2,387 | 1,69E-14 | -1,762 |
| LOC93622 | hypothetical LOC93622 |  |  | 1,11E-09 | -1,820 |  |  |
| LONP1 | lon peptidase 1, mitochondrial |  |  | 5,81E-10 | -1,822 |  |  |
| LPGAT1 | lysophosphatidylglycerol acyltransferase 1 |  |  | 6,46E-12 | 1,927 | 6,46E-12 | 2,212 |
| LPIN1 | lipin 1 |  |  | 6,80E-09 | -1,863 |  |  |
| LPP | LIM domain containing preferred translocation partner in lipoma |  |  |  |  | 3,80E-08 | 2,101 |
| LRPPRC | leucine-rich PPR-motif containing |  |  | 1,71E-09 | -1,474 |  |  |
| LRRC16A | leucine rich repeat containing 16A |  |  | 3,61E-11 | -1,540 | 3,61E-11 | -1,730 |
| LRRC40 | leucine rich repeat containing 40 |  |  | 3,68E-12 | -1,609 |  |  |
| LRRC47 | leucine rich repeat containing 47 |  |  | 7,66E-12 | -1,788 |  |  |
| LRRC58 | leucine rich repeat containing 58 |  |  | 1,77E-10 | -1,696 |  |  |
| LRRC59 | leucine rich repeat containing 59 |  |  | 3,87E-09 | -1,495 |  |  |
| LRRC8D | leucine rich repeat containing 8 family, member D |  |  | 7,17E-09 | -1,869 |  |  |
| LRRFIP1 | leucine rich repeat (in FLII) interacting protein 1 |  |  | 6,30E-10 | -1,619 |  |  |
| LSG1 | large subunit GTPase 1 homolog (S. cerevisiae) |  |  | 3,14E-14 | -2,064 |  |  |
| LSG1 | large subunit GTPase 1 homolog (S. cerevisiae) |  |  | 1,31E-13 | -1,968 |  |  |
| LSM14A | LSM14A, SCD6 homolog A (S. cerevisiae) |  |  | 1,69E-14 | -1,892 |  |  |
| LSM2 | LSM2 homolog, U6 small nuclear RNA associated (S. cerevisiae) |  |  | 5,84E-08 | -1,588 |  |  |
| LSM4 | LSM4 homolog, U6 small nuclear RNA associated (S. cerevisiae) | 6,43E-12 | -1,371 | 6,43E-12 | -1,752 | 6,43E-12 | -1,411 |
| LSM4 | LSM4 homolog, U6 small nuclear RNA associated (S. cerevisiae) |  |  | 5,96E-11 | -1,672 |  |  |
| LSM5 | LSM5 homolog, U6 small nuclear RNA associated (S. cerevisiae) |  |  | 1,88E-09 | -1,852 | 1,88E-09 | -1,609 |
| LSM5 | LSM5 homolog, U6 small nuclear RNA associated (S. cerevisiae) |  |  | 3,87E-13 | -1,806 |  |  |
| LSM5 | LSM5 homolog, U6 small nuclear RNA associated (S. cerevisiae) |  |  | 1,73E-13 | -1,586 | 1,73E-13 | -1,340 |
| LSM7 | LSM7 homolog, U6 small nuclear RNA associated (S. cerevisiae) |  |  | 1,98E-09 | -1,657 |  |  |
| LSM8 | LSM8 homolog, U6 small nuclear RNA associated (S. cerevisiae) |  |  | 2,42E-12 | -1,684 | 2,42E-12 | -1,471 |
| LSMD1 | LSM domain containing 1 |  |  | 1,43E-09 | -1,818 |  |  |
| LSR | lipolysis stimulated lipoprotein receptor | 1,69E-14 | -1,416 | 1,69E-14 | -2,417 | 1,69E-14 | -1,528 |
| LSS | lanosterol synthase (2,3-oxidosqualene-lanosterol cyclase) |  |  | 4,03E-13 | -1,914 |  |  |
| LTBP1 | latent transforming growth factor beta binding protein 1 |  |  | 1,78E-12 | -1,823 |  |  |
| LTBR | lymphotoxin beta receptor (TNFR superfamily, member 3) |  |  | 8,52E-11 | -2,381 |  |  |
| LUC7L2 | LUC7-like 2 (S. cerevisiae) |  |  | 9,08E-10 | -1,937 |  |  |
| LY6G5B | lymphocyte antigen 6 complex, locus G5B |  |  | 3,73E-09 | -1,593 |  |  |
| LYCAT | lysocardiolipin acyltransferase 1 |  |  | 2,26E-09 | -1,583 |  |  |
| LYRM1 | LYR motif containing 1 |  |  | 1,75E-10 | -1,703 |  |  |
| LYRM2 | LYR motif containing 2 |  |  | 1,46E-08 | -1,533 | 1,46E-08 | -1,321 |
| M6PR | mannose-6-phosphate receptor (cation dependent) |  |  | 1,69E-10 | -1,624 |  |  |
| MACF1 | microtubule-actin crosslinking factor 1 |  |  | 1,75E-11 | -1,633 |  |  |
| MACF1 | microtubule-actin crosslinking factor 1 | 3,29E-10 | -2,870 |  |  |  |  |
| MAD2L1 | MAD2 mitotic arrest deficient-like 1 (yeast) |  |  | 6,69E-11 | -1,497 | 6,69E-11 | -1,594 |
| MAEA | macrophage erythroblast attacher |  |  | 3,38E-12 | -1,923 |  |  |
| MAFF | v-maf musculoaponeurotic fibrosarcoma oncogene homolog F (avian) |  |  |  |  | 1,69E-14 | 1,735 |
| MAGED1 | melanoma antigen family D, 1 |  |  | 2,26E-08 | -1,639 |  |  |
| MAGED2 | melanoma antigen family D, 2 | 1,73E-13 | -1,464 | 1,73E-13 | -2,339 | 1,73E-13 | -1,589 |
| MAGI1 | membrane associated guanylate kinase, WW and PDZ domain containing 1 |  |  | 1,22E-08 | -1,772 |  |  |
| MAGOH | mago-nashi homolog, proliferation-associated (Drosophila) | 1,69E-14 | -1,691 | 1,69E-14 | -2,750 | 1,69E-14 | -2,213 |
| MAGOH | mago-nashi homolog, proliferation-associated (Drosophila) |  |  | 1,69E-14 | -2,573 | 1,69E-14 | -1,649 |
| MAGT1 | magnesium transporter 1 |  |  | 1,99E-10 | -1,655 |  |  |
| MAL2 | mal, T-cell differentiation protein 2 |  |  | 2,16E-12 | -1,703 |  |  |
| MALAT1 | metastasis associated lung adenocarcinoma transcript 1 (non-protein coding) | 1,32E-12 | 1,394 | 1,32E-12 | 1,482 | 1,32E-12 | 1,684 |
| MALAT1 | metastasis associated lung adenocarcinoma transcript 1 (non-protein coding) | 1,69E-14 | -1,934 | 1,69E-14 | -2,277 | 1,69E-14 | -3,166 |
| MALAT1 | metastasis associated lung adenocarcinoma transcript 1 (non-protein coding) |  |  |  |  | 1,69E-14 | 2,098 |
| MALAT1 | metastasis associated lung adenocarcinoma transcript 1 (non-protein coding) |  |  |  |  | 1,69E-14 | 2,027 |
| MALAT1 | metastasis associated lung adenocarcinoma transcript 1 (non-protein coding) |  |  |  |  | 1,69E-14 | 2,316 |
| MALAT1 | metastasis associated lung adenocarcinoma transcript 1 (non-protein coding) |  |  | 1,69E-14 | 1,766 | 1,69E-14 | 2,632 |
| MALAT1 | metastasis associated lung adenocarcinoma transcript 1 (non-protein coding) | 1,69E-14 | -2,928 | 1,69E-14 | -2,540 | 1,69E-14 | -2,742 |
| MALAT1 | metastasis associated lung adenocarcinoma transcript 1 (non-protein coding) | 1,69E-14 | -1,979 | 1,69E-14 | -2,257 | 1,69E-14 | -3,135 |
| MAML1 | mastermind-like 1 (Drosophila) |  |  | 4,77E-12 | -1,805 |  |  |
| MAN1B1 | mannosidase, alpha, class 1B, member 1 |  |  | 3,11E-09 | -2,002 |  |  |
| MANBAL | mannosidase, beta A, lysosomal-like |  |  | 1,59E-08 | -1,667 |  |  |
| MANEAL | mannosidase, endo-alpha-like |  |  | 1,05E-10 | -1,814 |  |  |
| MAP1LC3B | microtubule-associated protein 1 light chain 3 beta |  |  | 6,48E-11 | -1,600 | 6,48E-11 | -1,503 |
| MAP1LC3B | microtubule-associated protein 1 light chain 3 beta |  |  | 4,08E-08 | -1,681 |  |  |
| MAP2K1 | mitogen-activated protein kinase kinase 1 |  |  | 2,75E-08 | -1,689 |  |  |
| MAP3K13 | mitogen-activated protein kinase kinase kinase 13 |  |  | 4,34E-11 | -1,579 |  |  |
| MAP3K13 | mitogen-activated protein kinase kinase kinase 13 |  |  | 8,15E-10 | -1,763 |  |  |
| MAP3K7IP2 | mitogen-activated protein kinase kinase kinase 7 interacting protein 2 |  |  | 1,89E-08 | -1,582 |  |  |
| MAP4 | microtubule-associated protein 4 |  |  | 1,28E-09 | -1,726 |  |  |
| MAP4K3 | mitogen-activated protein kinase kinase kinase kinase 3 |  |  | 2,37E-09 | -1,573 |  |  |
| MAPK1 | mitogen-activated protein kinase 1 |  |  | 1,05E-08 | -1,607 |  |  |
| MAPK13 | mitogen-activated protein kinase 13 |  |  | 2,70E-09 | -1,765 |  |  |
| MAPK14 | mitogen-activated protein kinase 14 |  |  | 1,02E-08 | -1,731 |  |  |
| MAPK14 | mitogen-activated protein kinase 14 |  |  | 1,73E-13 | -1,934 | 1,73E-13 | -1,851 |
| MAPRE1 | microtubule-associated protein, RP/EB family, member 1 |  |  | 1,04E-08 | -1,518 |  |  |
| MARCH5 | membrane-associated ring finger (C3HC4) 5 |  |  | 4,29E-10 | -1,640 |  |  |
| MARCH6 | membrane-associated ring finger (C3HC4) 6 |  |  | 5,55E-09 | -1,567 |  |  |
| MARCKS (includes EG:4082) | myristoylated alanine-rich protein kinase C substrate |  |  |  |  | 1,69E-14 | 2,529 |
| MARCKS (includes EG:4082) | myristoylated alanine-rich protein kinase C substrate |  |  | 1,69E-14 | -1,698 | 1,69E-14 | -1,410 |
| MARCKS (includes EG:4082) | myristoylated alanine-rich protein kinase C substrate |  |  |  |  | 7,72E-10 | 1,565 |
| MARCKSL1 | MARCKS-like 1 |  |  | 1,32E-10 | -1,765 |  |  |
| MARS | methionyl-tRNA synthetase |  |  | 3,04E-09 | -1,629 |  |  |
| MARVELD2 | MARVEL domain containing 2 |  |  |  |  | 2,61E-09 | 1,733 |
| MATR3 | matrin 3 |  |  |  |  | 8,93E-09 | -1,628 |
| MATR3 | matrin 3 |  |  | 7,26E-13 | -1,626 | 7,26E-13 | -1,485 |
| MATR3 | matrin 3 |  |  | 1,90E-08 | -1,517 |  |  |
| MBD4 | methyl-CpG binding domain protein 4 |  |  | 1,25E-11 | -1,717 |  |  |
| MBD6 | methyl-CpG binding domain protein 6 |  |  | 2,38E-09 | -2,800 |  |  |
| MBD6 | methyl-CpG binding domain protein 6 |  |  | 4,44E-09 | -1,792 |  |  |
| MBIP | MAP3K12 binding inhibitory protein 1 |  |  | 2,85E-09 | -1,633 |  |  |
| MBOAT1 | membrane bound O-acyltransferase domain containing 1 |  |  |  |  | 2,30E-08 | -1,779 |
| MBTD1 | mbt domain containing 1 |  |  | 5,01E-09 | -1,634 |  |  |
| MCAM | melanoma cell adhesion molecule |  |  |  |  | 3,91E-08 | 1,610 |
| MCAT | malonyl CoA:ACP acyltransferase (mitochondrial) |  |  | 1,29E-09 | -2,195 |  |  |
| MCCC2 | methylcrotonoyl-Coenzyme A carboxylase 2 (beta) |  |  | 1,69E-14 | -1,843 | 1,69E-14 | -1,673 |
| MCCC2 | methylcrotonoyl-Coenzyme A carboxylase 2 (beta) |  |  | 5,71E-13 | -2,032 | 5,71E-13 | -1,531 |
| MCM2 | minichromosome maintenance complex component 2 |  |  | 1,78E-09 | -1,715 |  |  |
| MCM5 | minichromosome maintenance complex component 5 |  |  | 6,66E-08 | -1,615 |  |  |
| MCM6 | minichromosome maintenance complex component 6 |  |  | 3,89E-12 | -1,667 |  |  |
| MCM7 | minichromosome maintenance complex component 7 |  |  | 5,77E-14 | -1,851 |  |  |
| MCRS1 | microspherule protein 1 |  |  | 2,33E-09 | -1,910 |  |  |
| MCTS1 | malignant T cell amplified sequence 1 |  |  | 9,31E-10 | -1,623 |  |  |
| MDH1 | malate dehydrogenase 1, NAD (soluble) |  |  | 3,91E-09 | -1,470 |  |  |
| MDH2 | malate dehydrogenase 2, NAD (mitochondrial) |  |  | 2,80E-08 | -1,487 |  |  |
| MDK | midkine (neurite growth-promoting factor 2) |  |  | 2,93E-10 | -1,696 |  |  |
| ME2 | malic enzyme 2, NAD(+)-dependent, mitochondrial |  |  | 2,26E-10 | -1,607 | 2,26E-10 | -1,623 |
| MEA1 | male-enhanced antigen 1 |  |  | 1,15E-08 | -1,647 |  |  |
| MED1 | mediator complex subunit 1 |  |  |  |  | 1,89E-11 | 1,748 |
| MED16 | mediator complex subunit 16 |  |  | 3,69E-08 | -1,741 |  |  |
| MED19 | mediator complex subunit 19 |  |  | 8,03E-10 | -1,756 |  |  |
| MED21 | mediator complex subunit 21 |  |  | 3,54E-12 | -1,681 | 3,54E-12 | -1,487 |
| MED21 | mediator complex subunit 21 |  |  | 2,33E-10 | -1,508 | 2,33E-10 | -1,694 |
| MED23 | mediator complex subunit 23 |  |  | 3,10E-10 | -1,712 |  |  |
| MED25 | mediator complex subunit 25 |  |  | 5,21E-08 | -1,691 |  |  |
| MED30 | mediator complex subunit 30 |  |  | 5,80E-11 | -1,910 |  |  |
| MED4 | mediator complex subunit 4 |  |  | 4,83E-12 | -1,927 |  |  |
| MED4 | mediator complex subunit 4 |  |  | 6,10E-12 | -2,139 |  |  |
| MED7 (includes EG:9443) | mediator complex subunit 7 |  |  | 1,67E-08 | -1,570 |  |  |
| MEN1 | multiple endocrine neoplasia I |  |  | 2,23E-09 | -1,896 |  |  |
| MEPCE | methylphosphate capping enzyme |  |  | 1,12E-08 | -1,821 |  |  |
| MERTK | c-mer proto-oncogene tyrosine kinase |  |  | 2,26E-09 | -1,654 |  |  |
| MESDC1 | mesoderm development candidate 1 |  |  | 6,10E-12 | -1,949 |  |  |
| METAP2 (includes EG:10988) | methionyl aminopeptidase 2 |  |  | 2,22E-11 | -1,613 |  |  |
| METAP2 (includes EG:10988) | methionyl aminopeptidase 2 |  |  | 7,45E-08 | -1,691 |  |  |
| METT11D1 | methyltransferase 11 domain containing 1 |  |  | 2,51E-08 | -1,643 |  |  |
| METTL10 | methyltransferase like 10 |  |  | 1,70E-08 | -1,752 |  |  |
| METTL5 | methyltransferase like 5 |  |  |  |  | 3,35E-08 | 2,769 |
| METTL5 | methyltransferase like 5 |  |  | 7,04E-09 | -1,504 |  |  |
| METTL7A | methyltransferase like 7A |  |  | 1,92E-08 | -2,061 |  |  |
| METTL9 | methyltransferase like 9 |  |  | 1,34E-11 | -2,109 |  |  |
| MEX3D | mex-3 homolog D (C. elegans) | 4,96E-13 | 1,578 |  |  |  |  |
| MFAP1 | microfibrillar-associated protein 1 |  |  | 9,32E-08 | -1,486 |  |  |
| MFAP3 | microfibrillar-associated protein 3 |  |  | 2,25E-08 | -1,709 |  |  |
| MFF | mitochondrial fission factor |  |  | 4,66E-09 | -1,560 |  |  |
| MFN1 | mitofusin 1 |  |  | 3,34E-08 | -1,541 |  |  |
| MFN2 | mitofusin 2 |  |  | 1,25E-11 | -2,172 |  |  |
| MFSD11 | major facilitator superfamily domain containing 11 |  |  | 2,44E-11 | -1,854 |  |  |
| MGA (includes EG:23269) | MAX gene associated |  |  | 1,69E-14 | 2,361 | 1,69E-14 | 3,089 |
| MGAT1 | mannosyl (alpha-1,3-)-glycoprotein beta-1,2-N-acetylglucosaminyltransferase |  |  | 2,80E-09 | -2,293 |  |  |
| MGAT2 | mannosyl (alpha-1,6-)-glycoprotein beta-1,2-N-acetylglucosaminyltransferase |  |  | 5,11E-12 | -1,742 | 5,11E-12 | -1,687 |
| MGAT4B | mannosyl (alpha-1,3-)-glycoprotein beta-1,4-N-acetylglucosaminyltransferase, isozyme B |  |  | 1,69E-14 | -1,892 |  |  |
| MGAT4B | mannosyl (alpha-1,3-)-glycoprotein beta-1,4-N-acetylglucosaminyltransferase, isozyme B |  |  | 2,34E-08 | -1,529 |  |  |
| MGC16385 | hypothetical protein MGC16385 | 7,57E-08 | -1,623 |  |  |  |  |
| MGC3032 | hypothetical protein MGC3032 |  |  | 3,14E-14 | -2,219 |  |  |
| MGC3196 | hypothetical protein MGC3196 |  |  | 3,10E-09 | -1,655 |  |  |
| MGC70870 | C-terminal binding protein 2 pseudogene |  |  |  |  | 3,14E-14 | -2,114 |
| MGST1 | microsomal glutathione S-transferase 1 |  |  | 5,13E-13 | -1,569 | 5,13E-13 | -1,550 |
| MGST1 | microsomal glutathione S-transferase 1 |  |  | 2,37E-09 | -1,594 |  |  |
| MGST2 | microsomal glutathione S-transferase 2 |  |  | 3,59E-13 | -1,988 |  |  |
| MIB1 | mindbomb homolog 1 (Drosophila) |  |  |  |  | 3,65E-10 | 1,737 |
| MICAL2 | microtubule associated monoxygenase, calponin and LIM domain containing 2 |  |  | 1,01E-09 | -1,728 |  |  |
| MICALL1 | MICAL-like 1 |  |  | 1,10E-07 | -1,632 |  |  |
| MIDN | midnolin |  |  | 9,51E-14 | -2,211 |  |  |
| MIER1 | mesoderm induction early response 1 homolog (Xenopus laevis) |  |  | 2,91E-10 | -1,969 |  |  |
| MIF4GD | MIF4G domain containing |  |  | 7,94E-09 | -1,757 |  |  |
| MIF4GD | MIF4G domain containing |  |  | 3,42E-08 | -1,877 |  |  |
| MIPEP | mitochondrial intermediate peptidase |  |  | 6,92E-10 | -1,741 |  |  |
| MIRN21 (includes EG:406991) | microRNA 21 |  |  |  |  | 2,87E-10 | 1,485 |
| MITD1 | MIT, microtubule interacting and transport, domain containing 1 |  |  | 1,69E-14 | -1,924 | 1,69E-14 | -1,654 |
| MKI67 | antigen identified by monoclonal antibody Ki-67 |  |  | 1,67E-12 | -2,152 |  |  |
| MKNK2 | MAP kinase interacting serine/threonine kinase 2 |  |  | 4,52E-09 | -1,629 |  |  |
| MKRN1 | makorin ring finger protein 1 |  |  | 4,08E-08 | -1,671 |  |  |
| MLEC | malectin |  |  |  |  | 3,85E-10 | 1,447 |
| MLEC | malectin |  |  | 1,70E-11 | -1,712 |  |  |
| MLF2 | myeloid leukemia factor 2 |  |  | 1,69E-14 | -2,103 |  |  |
| MLL | myeloid/lymphoid or mixed-lineage leukemia (trithorax homolog, Drosophila) |  |  | 5,83E-10 | -1,932 |  |  |
| MLL3 | myeloid/lymphoid or mixed-lineage leukemia 3 |  |  |  |  | 8,57E-13 | 2,678 |
| MLXIP | MLX interacting protein |  |  | 1,20E-09 | -1,743 |  |  |
| MND1 | meiotic nuclear divisions 1 homolog (S. cerevisiae) |  |  | 5,25E-10 | -1,673 |  |  |
| MNS1 | meiosis-specific nuclear structural 1 |  |  | 1,91E-08 | -1,690 |  |  |
| MOBKL3 | MOB1, Mps One Binder kinase activator-like 3 (yeast) |  |  | 4,10E-08 | -1,453 |  |  |
| MORC4 | MORC family CW-type zinc finger 4 |  |  | 7,00E-12 | -1,786 |  |  |
| MPDU1 | mannose-P-dolichol utilization defect 1 | 6,92E-12 | -1,693 | 6,92E-12 | -2,386 | 6,92E-12 | -1,468 |
| MPHOSPH9 | M-phase phosphoprotein 9 |  |  | 5,45E-08 | -1,825 |  |  |
| MPRIP | myosin phosphatase Rho interacting protein |  |  | 6,23E-09 | -1,429 |  |  |
| MPZL1 | myelin protein zero-like 1 | 1,69E-14 | -1,353 | 1,69E-14 | -2,005 | 1,69E-14 | -1,425 |
| MPZL1 | myelin protein zero-like 1 |  |  |  |  | 6,11E-09 | -1,562 |
| MREG | melanoregulin |  |  | 4,49E-14 | -2,132 |  |  |
| MRP63 | mitochondrial ribosomal protein 63 |  |  | 1,72E-10 | -1,630 |  |  |
| MRPL10 | mitochondrial ribosomal protein L10 |  |  | 3,29E-10 | -1,771 |  |  |
| MRPL13 | mitochondrial ribosomal protein L13 |  |  | 5,14E-12 | -1,611 |  |  |
| MRPL14 | mitochondrial ribosomal protein L14 |  |  | 4,57E-09 | -1,657 |  |  |
| MRPL28 | mitochondrial ribosomal protein L28 |  |  | 1,58E-10 | -1,880 |  |  |
| MRPL3 | mitochondrial ribosomal protein L3 |  |  | 1,33E-09 | -1,417 |  |  |
| MRPL30 | mitochondrial ribosomal protein L30 |  |  | 7,16E-10 | -1,562 | 7,16E-10 | -1,483 |
| MRPL30 | mitochondrial ribosomal protein L30 | 1,69E-14 | -1,531 | 1,69E-14 | -2,729 | 1,69E-14 | -2,725 |
| MRPL34 | mitochondrial ribosomal protein L34 |  |  | 7,86E-09 | -1,748 |  |  |
| MRPL35 | mitochondrial ribosomal protein L35 |  |  | 2,24E-09 | -1,760 |  |  |
| MRPL37 | mitochondrial ribosomal protein L37 |  |  | 1,68E-11 | -1,871 |  |  |
| MRPL38 | mitochondrial ribosomal protein L38 |  |  | 9,97E-12 | -1,898 |  |  |
| MRPL39 | mitochondrial ribosomal protein L39 |  |  | 6,05E-12 | -1,614 | 6,05E-12 | -1,532 |
| MRPL4 | mitochondrial ribosomal protein L4 |  |  | 1,25E-11 | -2,025 |  |  |
| MRPL40 | mitochondrial ribosomal protein L40 |  |  | 1,89E-09 | -1,684 |  |  |
| MRPL41 | mitochondrial ribosomal protein L41 |  |  | 6,28E-11 | -1,662 |  |  |
| MRPL43 | mitochondrial ribosomal protein L43 |  |  | 3,72E-10 | -1,703 |  |  |
| MRPL48 | mitochondrial ribosomal protein L48 |  |  | 5,61E-09 | -1,663 |  |  |
| MRPL49 | mitochondrial ribosomal protein L49 |  |  | 2,27E-10 | -1,689 |  |  |
| MRPL50 | mitochondrial ribosomal protein L50 |  |  | 1,19E-13 | -1,638 | 1,19E-13 | -1,437 |
| MRPL9 | mitochondrial ribosomal protein L9 |  |  | 1,66E-09 | -1,548 |  |  |
| MRPS10 | mitochondrial ribosomal protein S10 |  |  | 6,38E-13 | -1,780 |  |  |
| MRPS15 | mitochondrial ribosomal protein S15 |  |  | 3,94E-13 | -1,904 | 3,94E-13 | -1,513 |
| MRPS15 | mitochondrial ribosomal protein S15 |  |  | 1,42E-10 | -1,647 |  |  |
| MRPS16 | mitochondrial ribosomal protein S16 |  |  | 3,34E-11 | -2,004 |  |  |
| MRPS18A | mitochondrial ribosomal protein S18A |  |  | 3,19E-10 | -1,813 |  |  |
| MRPS18B | mitochondrial ribosomal protein S18B |  |  | 7,07E-14 | -2,065 |  |  |
| MRPS2 | mitochondrial ribosomal protein S2 |  |  | 1,69E-14 | -2,158 |  |  |
| MRPS24 | mitochondrial ribosomal protein S24 |  |  | 5,53E-10 | -1,595 |  |  |
| MRPS28 | mitochondrial ribosomal protein S28 |  |  | 3,21E-09 | -1,605 |  |  |
| MRPS30 | mitochondrial ribosomal protein S30 |  |  | 6,81E-09 | -1,619 |  |  |
| MRPS31 | mitochondrial ribosomal protein S31 |  |  | 9,52E-09 | -1,654 |  |  |
| MRPS33 | mitochondrial ribosomal protein S33 |  |  | 2,62E-09 | -1,683 |  |  |
| MRPS36 | mitochondrial ribosomal protein S36 |  |  | 5,11E-12 | -1,640 | 5,11E-12 | -1,451 |
| MRPS5 | mitochondrial ribosomal protein S5 |  |  | 1,84E-10 | -1,680 |  |  |
| MRPS7 | mitochondrial ribosomal protein S7 |  |  | 1,40E-10 | -1,653 |  |  |
| MRS2 | MRS2 magnesium homeostasis factor homolog (S. cerevisiae) |  |  | 2,09E-10 | -1,596 |  |  |
| MSH2 | mutS homolog 2, colon cancer, nonpolyposis type 1 (E. coli) |  |  | 2,16E-13 | -1,585 | 2,16E-13 | -1,664 |
| MSL1 | male-specific lethal 1 homolog (Drosophila) |  |  | 2,53E-13 | -1,837 | 2,53E-13 | -1,601 |
| MSRB2 | methionine sulfoxide reductase B2 |  |  | 2,56E-08 | -1,673 |  |  |
| MSX2 | msh homeobox 2 |  |  | 2,90E-11 | -2,196 |  |  |
| MT1F | metallothionein 1F |  |  |  |  | 9,14E-09 | -1,942 |
| MTCP1 | mature T-cell proliferation 1 |  |  | 5,07E-08 | -1,787 |  |  |
| MTDH | metadherin |  |  | 6,19E-11 | -1,660 |  |  |
| MTDH | metadherin |  |  | 4,68E-12 | -1,844 |  |  |
| MTF2 | metal response element binding transcription factor 2 |  |  | 1,32E-10 | -1,949 | 1,32E-10 | -1,744 |
| MTFR1 | mitochondrial fission regulator 1 |  |  | 4,38E-09 | -1,712 |  |  |
| MTHFS | 5,10-methenyltetrahydrofolate synthetase (5-formyltetrahydrofolate cyclo-ligase) |  |  | 1,58E-09 | -1,929 |  |  |
| MTIF3 | mitochondrial translational initiation factor 3 |  |  | 2,07E-12 | -2,045 |  |  |
| MTMR12 | myotubularin related protein 12 |  |  | 1,89E-09 | -1,538 |  |  |
| MTMR4 | myotubularin related protein 4 | 1,69E-14 | -1,600 | 1,69E-14 | -2,505 | 1,69E-14 | -1,749 |
| MTO1 | mitochondrial translation optimization 1 homolog (S. cerevisiae) |  |  | 2,88E-09 | -1,714 |  |  |
| MTRF1L | mitochondrial translational release factor 1-like |  |  | 1,12E-12 | -2,329 |  |  |
| MTUS1 | mitochondrial tumor suppressor 1 |  |  |  |  | 1,15E-08 | -2,221 |
| MTUS1 | mitochondrial tumor suppressor 1 |  |  | 1,69E-14 | -1,852 | 1,69E-14 | -1,438 |
| MTX1 (includes EG:4580) | metaxin 1 |  |  | 1,49E-08 | -1,681 |  |  |
| MUM1 | melanoma associated antigen (mutated) 1 |  |  | 1,08E-10 | -2,620 |  |  |
| MUTYH | mutY homolog (E. coli) |  |  |  |  | 8,64E-08 | -1,682 |
| MXRA7 | matrix-remodelling associated 7 |  |  | 2,80E-10 | -1,625 |  |  |
| MYADM | myeloid-associated differentiation marker |  |  | 1,69E-14 | -1,845 |  |  |
| MYBL2 | v-myb myeloblastosis viral oncogene homolog (avian)-like 2 |  |  | 1,69E-14 | -2,112 |  |  |
| MYCBP | c-myc binding protein |  |  | 1,66E-09 | -1,635 |  |  |
| MYCBP | c-myc binding protein |  |  | 2,67E-10 | -1,693 | 2,67E-10 | -1,583 |
| MYCN | v-myc myelocytomatosis viral related oncogene, neuroblastoma derived (avian) |  |  | 6,86E-09 | -1,669 | 6,86E-09 | -1,786 |
| MYEOV2 | myeloma overexpressed 2 |  |  | 1,01E-11 | -1,931 |  |  |
| MYH10 | myosin, heavy chain 10, non-muscle |  |  | 2,87E-08 | -1,585 |  |  |
| MYH10 | myosin, heavy chain 10, non-muscle | 1,69E-14 | -1,997 |  |  |  |  |
| MYL6B | myosin, light chain 6B, alkali, smooth muscle and non-muscle |  |  | 8,74E-10 | -2,043 |  |  |
| MYL9 (includes EG:10398) | myosin, light chain 9, regulatory |  |  | 4,49E-14 | -1,965 |  |  |
| MYLK | myosin light chain kinase |  |  | 5,84E-09 | -1,590 |  |  |
| MYNN | myoneurin |  |  | 1,69E-12 | -1,923 | 1,69E-12 | -1,517 |
| MYO1D | myosin ID |  |  | 1,69E-14 | -2,118 |  |  |
| MYO6 | myosin VI |  |  |  |  | 1,69E-14 | 2,066 |
| MYST3 | MYST histone acetyltransferase (monocytic leukemia) 3 |  |  | 2,05E-09 | -1,734 |  |  |
| NAALAD2 | N-acetylated alpha-linked acidic dipeptidase 2 |  |  | 4,96E-13 | -1,584 | 4,96E-13 | -1,754 |
| NAB1 | NGFI-A binding protein 1 (EGR1 binding protein 1) |  |  |  |  | 5,50E-09 | -1,926 |
| NAB1 | NGFI-A binding protein 1 (EGR1 binding protein 1) |  |  | 5,83E-10 | -1,628 |  |  |
| NACC2 | NACC family member 2, BEN and BTB (POZ) domain containing |  |  | 1,64E-09 | -1,647 |  |  |
| NADK (includes EG:65220) | NAD kinase |  |  | 8,83E-09 | -1,546 |  |  |
| NADK (includes EG:65220) | NAD kinase |  |  | 7,11E-13 | -2,008 |  |  |
| NADSYN1 | NAD synthetase 1 |  |  | 5,04E-08 | -1,805 |  |  |
| NAGA | N-acetylgalactosaminidase, alpha- |  |  | 2,02E-08 | -1,934 |  |  |
| NAMPT | nicotinamide phosphoribosyltransferase |  |  | 2,27E-09 | -1,569 |  |  |
| NAP1L1 | nucleosome assembly protein 1-like 1 |  |  | 2,61E-08 | -1,439 |  |  |
| NAP1L1 | nucleosome assembly protein 1-like 1 |  |  | 3,14E-09 | -1,518 |  |  |
| NAP1L1 | nucleosome assembly protein 1-like 1 |  |  | 3,27E-11 | -1,572 |  |  |
| NAP1L1 | nucleosome assembly protein 1-like 1 |  |  | 3,91E-09 | -1,471 |  |  |
| NAP1L1 | nucleosome assembly protein 1-like 1 |  |  | 4,15E-09 | -1,474 |  |  |
| NAPA | N-ethylmaleimide-sensitive factor attachment protein, alpha |  |  | 2,41E-09 | -2,087 |  |  |
| NARG1 | NMDA receptor regulated 1 |  |  |  |  | 1,53E-13 | 1,955 |
| NARS2 | asparaginyl-tRNA synthetase 2, mitochondrial (putative) |  |  | 3,18E-10 | -1,580 | 3,18E-10 | -1,505 |
| NASP | nuclear autoantigenic sperm protein (histone-binding) |  |  |  |  | 5,46E-11 | 2,050 |
| NAT1 | N-acetyltransferase 1 (arylamine N-acetyltransferase) |  |  | 3,99E-10 | -1,532 |  |  |
| NAT11 | N-acetyltransferase 11 (GCN5-related, putative) |  |  | 7,26E-13 | -1,651 | 7,26E-13 | -1,626 |
| NAT15 | N-acetyltransferase 15 (GCN5-related, putative) |  |  | 2,63E-10 | -1,842 |  |  |
| NAT5 | N-acetyltransferase 5 (GCN5-related, putative) |  |  | 3,35E-08 | -1,525 |  |  |
| NAV2 (includes EG:89797) | neuron navigator 2 |  |  | 1,49E-08 | -1,993 |  |  |
| NBPF8 | neuroblastoma breakpoint family, member 8 |  |  | 2,39E-08 | -1,438 |  |  |
| NBPF8 | neuroblastoma breakpoint family, member 8 | 1,69E-14 | -1,965 | 1,69E-14 | -2,410 | 1,69E-14 | -1,702 |
| NBPF8 | neuroblastoma breakpoint family, member 8 | 5,31E-10 | -1,940 | 5,31E-10 | -1,707 | 5,31E-10 | -1,688 |
| NBPF8 | neuroblastoma breakpoint family, member 8 |  |  | 1,99E-08 | -1,432 |  |  |
| NCAPD3 | non-SMC condensin II complex, subunit D3 |  |  | 4,27E-12 | -1,751 |  |  |
| NCAPG2 | non-SMC condensin II complex, subunit G2 |  |  | 9,43E-13 | -2,176 |  |  |
| NCBP1 (includes EG:4686) | nuclear cap binding protein subunit 1, 80kDa |  |  | 2,05E-09 | -1,659 |  |  |
| NCK1 | NCK adaptor protein 1 |  |  | 5,72E-08 | -1,367 |  |  |
| NCKAP1 | NCK-associated protein 1 |  |  | 1,53E-13 | -1,488 | 1,53E-13 | -1,702 |
| NCOA7 | nuclear receptor coactivator 7 |  |  |  |  | 1,69E-14 | 1,593 |
| NCRNA00084 | non-protein coding RNA 84 |  |  | 8,53E-08 | -1,615 |  |  |
| NCRNA00084 | non-protein coding RNA 84 |  |  | 1,19E-11 | -2,068 | 1,19E-11 | -1,649 |
| NCRNA00084 | non-protein coding RNA 84 |  |  |  |  | 6,51E-09 | 3,159 |
| NCRNA00084 | non-protein coding RNA 84 |  |  |  |  | 7,24E-12 | 1,476 |
| NCRNA00094 | non-protein coding RNA 94 |  |  | 1,20E-09 | -2,237 |  |  |
| NCRNA00152 | non-protein coding RNA 152 |  |  | 5,50E-08 | -1,710 |  |  |
| NDEL1 | nudE nuclear distribution gene E homolog (A. nidulans)-like 1 |  |  | 8,38E-09 | -1,847 |  |  |
| NDFIP1 | Nedd4 family interacting protein 1 |  |  | 1,69E-14 | -1,907 |  |  |
| NDFIP1 | Nedd4 family interacting protein 1 |  |  | 1,35E-08 | -1,562 |  |  |
| NDUFA10 (includes EG:4705) | NADH dehydrogenase (ubiquinone) 1 alpha subcomplex, 10, 42kDa |  |  | 8,74E-13 | -1,956 |  |  |
| NDUFA11 | NADH dehydrogenase (ubiquinone) 1 alpha subcomplex, 11, 14.7kDa |  |  | 1,69E-14 | -1,734 |  |  |
| NDUFA11 | NADH dehydrogenase (ubiquinone) 1 alpha subcomplex, 11, 14.7kDa |  |  | 2,03E-12 | -1,771 |  |  |
| NDUFA13 | NADH dehydrogenase (ubiquinone) 1 alpha subcomplex, 13 |  |  | 5,73E-11 | -1,740 |  |  |
| NDUFA5 | NADH dehydrogenase (ubiquinone) 1 alpha subcomplex, 5, 13kDa |  |  | 2,81E-10 | -1,614 |  |  |
| NDUFAB1 | NADH dehydrogenase (ubiquinone) 1, alpha/beta subcomplex, 1, 8kDa |  |  | 1,29E-10 | -1,585 |  |  |
| NDUFAF2 | NADH dehydrogenase (ubiquinone) 1 alpha subcomplex, assembly factor 2 |  |  | 1,11E-10 | -1,700 |  |  |
| NDUFB1 | NADH dehydrogenase (ubiquinone) 1 beta subcomplex, 1, 7kDa |  |  | 1,03E-12 | -1,704 | 1,03E-12 | -1,402 |
| NDUFB10 (includes EG:4716) | NADH dehydrogenase (ubiquinone) 1 beta subcomplex, 10, 22kDa |  |  | 3,14E-14 | -1,890 |  |  |
| NDUFB11 | NADH dehydrogenase (ubiquinone) 1 beta subcomplex, 11, 17.3kDa |  |  | 3,83E-08 | -1,711 |  |  |
| NDUFB2 | NADH dehydrogenase (ubiquinone) 1 beta subcomplex, 2, 8kDa |  |  | 3,15E-09 | -1,610 |  |  |
| NDUFB2 | NADH dehydrogenase (ubiquinone) 1 beta subcomplex, 2, 8kDa |  |  | 7,53E-12 | -1,901 |  |  |
| NDUFB6 | NADH dehydrogenase (ubiquinone) 1 beta subcomplex, 6, 17kDa |  |  | 7,07E-14 | -1,902 |  |  |
| NDUFB7 | NADH dehydrogenase (ubiquinone) 1 beta subcomplex, 7, 18kDa |  |  | 3,05E-08 | -1,691 |  |  |
| NDUFC1 | NADH dehydrogenase (ubiquinone) 1, subcomplex unknown, 1, 6kDa |  |  | 3,31E-10 | -1,526 |  |  |
| NDUFS3 | NADH dehydrogenase (ubiquinone) Fe-S protein 3, 30kDa (NADH-coenzyme Q reductase) |  |  | 9,23E-08 | -1,501 |  |  |
| NDUFS8 | NADH dehydrogenase (ubiquinone) Fe-S protein 8, 23kDa (NADH-coenzyme Q reductase) |  |  | 5,01E-08 | -1,704 |  |  |
| NDUFS8 | NADH dehydrogenase (ubiquinone) Fe-S protein 8, 23kDa (NADH-coenzyme Q reductase) |  |  | 8,12E-12 | -1,872 |  |  |
| NDUFV2 | NADH dehydrogenase (ubiquinone) flavoprotein 2, 24kDa |  |  | 7,96E-12 | -1,764 |  |  |
| NDUFV3 | NADH dehydrogenase (ubiquinone) flavoprotein 3, 10kDa |  |  | 1,17E-12 | -1,818 |  |  |
| NEDD8 | neural precursor cell expressed, developmentally down-regulated 8 |  |  | 4,21E-09 | -1,649 |  |  |
| NEK2 | NIMA (never in mitosis gene a)-related kinase 2 |  |  | 4,46E-09 | -1,664 |  |  |
| NEK2 | NIMA (never in mitosis gene a)-related kinase 2 |  |  | 6,84E-08 | -1,654 |  |  |
| NEK6 | NIMA (never in mitosis gene a)-related kinase 6 |  |  | 4,30E-08 | -1,765 |  |  |
| NENF | neuron derived neurotrophic factor |  |  | 7,44E-10 | -1,805 | 7,44E-10 | -1,640 |
| NENF | neuron derived neurotrophic factor |  |  | 3,56E-10 | -2,003 |  |  |
| NES | nestin |  |  | 5,21E-09 | -1,801 |  |  |
| NETO2 | neuropilin (NRP) and tolloid (TLL)-like 2 |  |  | 8,31E-08 | -1,730 |  |  |
| NEU1 | sialidase 1 (lysosomal sialidase) |  |  | 5,33E-10 | -1,588 |  |  |
| NF2 | neurofibromin 2 (merlin) |  |  | 2,60E-08 | -1,972 | 2,60E-08 | -1,495 |
| NFAT5 | nuclear factor of activated T-cells 5, tonicity-responsive | 1,69E-14 | 2,479 | 1,69E-14 | 3,048 | 1,69E-14 | 3,781 |
| NFAT5 | nuclear factor of activated T-cells 5, tonicity-responsive |  |  |  |  | 1,45E-09 | 1,950 |
| NFATC2IP | nuclear factor of activated T-cells, cytoplasmic, calcineurin-dependent 2 interacting protein | 1,80E-10 | -1,374 | 1,80E-10 | -1,737 | 1,80E-10 | -1,444 |
| NFATC2IP | nuclear factor of activated T-cells, cytoplasmic, calcineurin-dependent 2 interacting protein |  |  | 2,46E-08 | -1,649 |  |  |
| NFKBIZ | nuclear factor of kappa light polypeptide gene enhancer in B-cells inhibitor, zeta |  |  | 2,78E-11 | -1,668 |  |  |
| NFS1 | NFS1 nitrogen fixation 1 homolog (S. cerevisiae) |  |  | 5,84E-09 | -1,992 |  |  |
| NFU1 | NFU1 iron-sulfur cluster scaffold homolog (S. cerevisiae) |  |  | 1,44E-09 | -1,555 |  |  |
| NGDN | neuroguidin, EIF4E binding protein |  |  | 1,94E-09 | -1,528 |  |  |
| NGRN | neugrin, neurite outgrowth associated |  |  | 1,87E-10 | -1,587 |  |  |
| NHP2 | NHP2 ribonucleoprotein homolog (yeast) |  |  | 2,02E-11 | -1,790 |  |  |
| NHP2L1 | NHP2 non-histone chromosome protein 2-like 1 (S. cerevisiae) |  |  | 4,34E-10 | -1,588 |  |  |
| NID1 | nidogen 1 |  |  | 5,42E-11 | -1,724 |  |  |
| NIPBL | Nipped-B homolog (Drosophila) |  |  |  |  | 1,69E-14 | 2,036 |
| NIPSNAP3A | nipsnap homolog 3A (C. elegans) |  |  | 1,43E-13 | -1,591 | 1,43E-13 | -1,571 |
| NLK | nemo-like kinase |  |  |  |  | 2,42E-08 | 1,317 |
| NMD3 | NMD3 homolog (S. cerevisiae) |  |  | 5,47E-12 | -1,612 | 5,47E-12 | -1,677 |
| NME4 | non-metastatic cells 4, protein expressed in |  |  | 1,34E-10 | -1,883 |  |  |
| NME7 | non-metastatic cells 7, protein expressed in (nucleoside-diphosphate kinase) |  |  |  |  | 1,69E-14 | -1,661 |
| NMRAL1 | NmrA-like family domain containing 1 |  |  | 2,31E-09 | -1,779 |  |  |
| NMT1 | N-myristoyltransferase 1 |  |  | 4,49E-14 | -1,856 | 4,49E-14 | -1,311 |
| NOL7 | nucleolar protein 7, 27kDa |  |  | 8,22E-08 | -1,440 |  |  |
| NOLC1 | nucleolar and coiled-body phosphoprotein 1 |  |  | 7,92E-09 | -1,695 |  |  |
| NONO | non-POU domain containing, octamer-binding | 3,57E-07 | -1,427 |  |  |  |  |
| NOSIP | nitric oxide synthase interacting protein |  |  | 1,21E-08 | -1,782 |  |  |
| NP | nucleoside phosphorylase |  |  | 3,49E-08 | -1,751 |  |  |
| N-PAC | cytokine-like nuclear factor n-pac |  |  | 7,55E-09 | -1,738 |  |  |
| NPEPL1 | aminopeptidase-like 1 |  |  | 3,11E-12 | -2,074 |  |  |
| NPEPPS | aminopeptidase puromycin sensitive |  |  | 2,19E-10 | -1,871 |  |  |
| NPM3 | nucleophosmin/nucleoplasmin, 3 |  |  | 5,39E-09 | -1,739 |  |  |
| NR2F2 | nuclear receptor subfamily 2, group F, member 2 |  |  |  |  | 4,61E-12 | 2,433 |
| NR2F2 | nuclear receptor subfamily 2, group F, member 2 |  |  | 1,07E-13 | -1,634 | 1,07E-13 | -1,490 |
| NR2F2 | nuclear receptor subfamily 2, group F, member 2 |  |  | 1,69E-14 | -2,113 | 1,69E-14 | -1,706 |
| NRAS | neuroblastoma RAS viral (v-ras) oncogene homolog | 1,71E-08 | -1,355 | 1,71E-08 | -1,518 |  |  |
| NRBF2 | nuclear receptor binding factor 2 |  |  | 1,33E-10 | -1,736 |  |  |
| NRBF2 | nuclear receptor binding factor 2 |  |  | 5,77E-14 | -1,859 | 5,77E-14 | -1,609 |
| NRM | nurim (nuclear envelope membrane protein) |  |  | 7,24E-08 | -1,900 |  |  |
| NSF | N-ethylmaleimide-sensitive factor |  |  | 9,26E-09 | -1,649 |  |  |
| NSFL1C | NSFL1 (p97) cofactor (p47) |  |  | 9,37E-13 | -2,139 |  |  |
| NSFL1C | NSFL1 (p97) cofactor (p47) |  |  | 6,58E-10 | -1,611 |  |  |
| NSMCE1 | non-SMC element 1 homolog (S. cerevisiae) |  |  | 1,14E-07 | -1,539 |  |  |
| NSMCE2 | non-SMC element 2, MMS21 homolog (S. cerevisiae) |  |  | 2,57E-12 | -1,805 | 2,57E-12 | -1,695 |
| NSMCE4A | non-SMC element 4 homolog A (S. cerevisiae) |  |  | 1,69E-14 | -1,967 | 1,69E-14 | -1,674 |
| NSMCE4A | non-SMC element 4 homolog A (S. cerevisiae) |  |  | 3,59E-08 | -1,644 |  |  |
| NSUN5 | NOL1/NOP2/Sun domain family, member 5 |  |  | 1,58E-10 | -1,649 |  |  |
| NT5DC1 | 5'-nucleotidase domain containing 1 |  |  | 5,33E-10 | -1,645 |  |  |
| NT5DC2 | 5'-nucleotidase domain containing 2 |  |  | 1,69E-14 | -2,208 |  |  |
| NTN4 | netrin 4 | 2,63E-13 | -1,308 | 2,63E-13 | -1,748 | 2,63E-13 | -1,659 |
| NUBP1 | nucleotide binding protein 1 (MinD homolog, E. coli) |  |  | 7,47E-08 | -1,760 |  |  |
| NUCB2 | nucleobindin 2 |  |  | 2,44E-13 | -1,872 |  |  |
| NUCKS1 | nuclear casein kinase and cyclin-dependent kinase substrate 1 | 1,69E-14 | -1,851 | 1,69E-14 | -2,829 | 1,69E-14 | -1,865 |
| NUCKS1 | nuclear casein kinase and cyclin-dependent kinase substrate 1 |  |  | 9,41E-10 | -1,554 |  |  |
| NUDCD2 | NudC domain containing 2 |  |  | 4,00E-11 | -1,642 |  |  |
| NUDT13 (includes EG:25961) | nudix (nucleoside diphosphate linked moiety X)-type motif 13 |  |  |  |  | 7,61E-07 | -1,431 |
| NUDT4 | nudix (nucleoside diphosphate linked moiety X)-type motif 4 |  |  | 4,59E-10 | -1,571 |  |  |
| NUDT7 | nudix (nucleoside diphosphate linked moiety X)-type motif 7 |  |  | 1,34E-08 | -1,927 |  |  |
| NUF2 | NUF2, NDC80 kinetochore complex component, homolog (S. cerevisiae) |  |  | 6,62E-11 | -1,691 |  |  |
| NUMA1 | nuclear mitotic apparatus protein 1 |  |  | 2,48E-09 | -2,130 |  |  |
| NUMB | numb homolog (Drosophila) |  |  | 1,69E-14 | -2,357 | 1,69E-14 | -1,706 |
| NUP107 | nucleoporin 107kDa |  |  | 1,46E-09 | -1,539 |  |  |
| NUP133 | nucleoporin 133kDa |  |  | 7,02E-10 | -1,550 |  |  |
| NUP153 | nucleoporin 153kDa |  |  | 5,74E-11 | -1,582 |  |  |
| NUP160 | nucleoporin 160kDa |  |  | 3,14E-14 | -1,851 |  |  |
| NUP210 | nucleoporin 210kDa |  |  | 5,65E-11 | -2,213 |  |  |
| NUP62 | nucleoporin 62kDa |  |  | 3,43E-11 | -2,010 |  |  |
| NUP85 | nucleoporin 85kDa |  |  | 5,13E-09 | -1,591 |  |  |
| NUP88 | nucleoporin 88kDa |  |  | 3,56E-09 | -1,621 |  |  |
| NUTF2 | nuclear transport factor 2 |  |  |  |  | 5,38E-09 | 1,448 |
| NXT2 | nuclear transport factor 2-like export factor 2 |  |  |  |  | 4,85E-08 | -3,444 |
| OBFC2A | oligonucleotide/oligosaccharide-binding fold containing 2A |  |  | 1,03E-06 | -1,427 |  |  |
| OCIAD1 | OCIA domain containing 1 |  |  | 3,14E-14 | -1,818 | 3,14E-14 | -1,516 |
| OCIAD1 | OCIA domain containing 1 |  |  | 1,14E-09 | -1,980 |  |  |
| OGFOD2 | 2-oxoglutarate and iron-dependent oxygenase domain containing 2 |  |  | 1,63E-10 | -2,403 |  |  |
| OGG1 | 8-oxoguanine DNA glycosylase |  |  | 1,61E-11 | -2,533 | 1,61E-11 | -2,085 |
| OIP5 | Opa interacting protein 5 |  |  | 1,58E-10 | -1,618 |  |  |
| OPRS1 | sigma non-opioid intracellular receptor 1 |  |  | 7,48E-10 | -1,983 |  |  |
| OPRS1 | sigma non-opioid intracellular receptor 1 |  |  | 1,43E-13 | -2,268 | 1,43E-13 | -1,748 |
| ORC5L | origin recognition complex, subunit 5-like (yeast) |  |  | 3,66E-09 | -1,699 |  |  |
| ORC5L | origin recognition complex, subunit 5-like (yeast) | 1,63E-13 | -1,436 | 1,63E-13 | -2,006 | 1,63E-13 | -1,944 |
| ORMDL2 | ORM1-like 2 (S. cerevisiae) |  |  | 4,04E-09 | -1,672 |  |  |
| ORMDL3 | ORM1-like 3 (S. cerevisiae) | 3,18E-12 | -1,791 | 3,18E-12 | -2,735 |  |  |
| OS9 | amplified in osteosarcoma |  |  | 1,69E-14 | -2,122 |  |  |
| OS9 | amplified in osteosarcoma |  |  | 2,62E-12 | -1,941 |  |  |
| OSBPL2 | oxysterol binding protein-like 2 |  |  | 9,00E-13 | -2,008 |  |  |
| OSBPL8 | oxysterol binding protein-like 8 |  |  | 2,20E-11 | -1,585 |  |  |
| OSBPL8 | oxysterol binding protein-like 8 |  |  |  |  | 1,69E-14 | 1,970 |
| OSBPL9 | oxysterol binding protein-like 9 |  |  | 3,74E-09 | -1,515 | 3,74E-09 | -1,413 |
| OTUD4 | OTU domain containing 4 |  |  |  |  | 3,98E-08 | 3,055 |
| OXCT1 | 3-oxoacid CoA transferase 1 |  |  | 2,51E-08 | -2,216 |  |  |
| OXR1 | oxidation resistance 1 |  |  | 1,96E-10 | -1,809 |  |  |
| P4HB | prolyl 4-hydroxylase, beta polypeptide | 1,63E-13 | -1,457 | 1,63E-13 | -1,893 | 1,63E-13 | -1,513 |
| P4HB | prolyl 4-hydroxylase, beta polypeptide |  |  | 1,02E-09 | -1,712 |  |  |
| PA2G4 | proliferation-associated 2G4, 38kDa |  |  | 1,71E-10 | -1,885 |  |  |
| PAIP1 | poly(A) binding protein interacting protein 1 |  |  | 3,95E-09 | -1,530 |  |  |
| PAIP1 | poly(A) binding protein interacting protein 1 |  |  | 3,18E-09 | -1,611 |  |  |
| PAIP2 | poly(A) binding protein interacting protein 2 | 3,95E-11 | -1,304 | 3,95E-11 | -1,605 | 3,95E-11 | -1,567 |
| PAK2 | p21 protein (Cdc42/Rac)-activated kinase 2 |  |  | 2,44E-13 | -1,895 |  |  |
| PANK4 | pantothenate kinase 4 |  |  | 8,41E-11 | -2,270 |  |  |
| PANX1 | pannexin 1 |  |  |  |  | 9,12E-11 | 1,400 |
| PAPD1 | PAP associated domain containing 1 |  |  | 2,61E-10 | -1,660 | 2,61E-10 | -2,064 |
| PAPD4 | PAP associated domain containing 4 |  |  | 6,52E-10 | -1,675 |  |  |
| PAPD5 | PAP associated domain containing 5 |  |  |  |  | 8,38E-10 | 1,461 |
| PAPSS1 (includes EG:9061) | 3'-phosphoadenosine 5'-phosphosulfate synthase 1 |  |  | 1,83E-08 | -1,528 |  |  |
| PARD6B | par-6 partitioning defective 6 homolog beta (C. elegans) |  |  | 1,69E-14 | -2,190 | 1,69E-14 | -1,549 |
| PARL | presenilin associated, rhomboid-like |  |  | 3,00E-10 | -1,613 |  |  |
| PARP2 | poly (ADP-ribose) polymerase 2 |  |  | 1,09E-10 | -1,880 |  |  |
| PARP2 | poly (ADP-ribose) polymerase 2 |  |  | 5,14E-11 | -1,927 |  |  |
| PARP6 | poly (ADP-ribose) polymerase family, member 6 |  |  | 1,78E-09 | -1,857 |  |  |
| PATL1 | protein associated with topoisomerase II homolog 1 (yeast) |  |  |  |  | 6,56E-09 | -1,559 |
| PATL1 | protein associated with topoisomerase II homolog 1 (yeast) |  |  | 2,28E-10 | -2,020 | 2,28E-10 | -2,244 |
| PAXIP1 | PAX interacting (with transcription-activation domain) protein 1 |  |  | 2,45E-08 | -1,512 |  |  |
| PBK | PDZ binding kinase |  |  | 1,69E-14 | -1,817 | 1,69E-14 | -1,584 |
| PBLD | phenazine biosynthesis-like protein domain containing |  |  | 3,28E-10 | -1,600 |  |  |
| PBX1 | pre-B-cell leukemia homeobox 1 |  |  | 9,26E-09 | -1,889 |  |  |
| PCBD1 | pterin-4 alpha-carbinolamine dehydratase/dimerization cofactor of hepatocyte nuclear factor 1 alpha |  |  | 8,33E-11 | -1,659 |  |  |
| PCBP1 (includes EG:5093) | poly(rC) binding protein 1 |  |  | 8,26E-09 | -1,586 |  |  |
| PCBP2 | poly(rC) binding protein 2 |  |  | 7,52E-11 | -1,775 |  |  |
| PCBP2 | poly(rC) binding protein 2 |  |  | 2,77E-08 | -1,762 |  |  |
| PCBP2 | poly(rC) binding protein 2 |  |  | 4,14E-10 | -1,916 |  |  |
| PCBP2 | poly(rC) binding protein 2 |  |  |  |  | 2,50E-10 | 1,810 |
| PCCB | propionyl Coenzyme A carboxylase, beta polypeptide |  |  | 1,48E-12 | -1,878 |  |  |
| PCGF2 | polycomb group ring finger 2 |  |  | 1,70E-08 | -1,803 |  |  |
| PCGF5 | polycomb group ring finger 5 |  |  | 1,92E-10 | -1,899 |  |  |
| PCGF6 | polycomb group ring finger 6 |  |  | 1,89E-10 | -1,757 |  |  |
| PCM1 | pericentriolar material 1 |  |  | 9,77E-13 | -1,801 |  |  |
| PCM1 | pericentriolar material 1 |  |  | 1,46E-08 | -1,764 |  |  |
| PCMT1 | protein-L-isoaspartate (D-aspartate) O-methyltransferase |  |  | 1,69E-14 | -1,852 | 1,69E-14 | -1,582 |
| PCMTD2 | protein-L-isoaspartate (D-aspartate) O-methyltransferase domain containing 2 |  |  | 1,69E-14 | -1,813 | 1,69E-14 | -1,610 |
| PCNP | PEST proteolytic signal containing nuclear protein |  |  | 4,76E-12 | -1,597 | 4,76E-12 | -1,620 |
| PCNP | PEST proteolytic signal containing nuclear protein |  |  | 1,69E-14 | -1,768 | 1,69E-14 | -1,606 |
| PCTP | phosphatidylcholine transfer protein |  |  | 8,92E-09 | -1,725 |  |  |
| PCYOX1 | prenylcysteine oxidase 1 |  |  |  |  | 3,63E-10 | 1,319 |
| PDAP1 | PDGFA associated protein 1 | 1,69E-14 | -1,686 | 1,69E-14 | -2,541 | 1,69E-14 | -1,733 |
| PDCD5 | programmed cell death 5 |  |  | 2,13E-09 | -1,744 |  |  |
| PDCL | phosducin-like | 1,28E-12 | -1,478 | 1,28E-12 | -2,231 |  |  |
| PDHA1 (includes EG:5160) | pyruvate dehydrogenase (lipoamide) alpha 1 |  |  | 9,80E-11 | -1,722 |  |  |
| PDHA1 (includes EG:5160) | pyruvate dehydrogenase (lipoamide) alpha 1 |  |  | 1,07E-07 | -1,732 |  |  |
| PDHB | pyruvate dehydrogenase (lipoamide) beta |  |  | 3,12E-09 | -1,565 |  |  |
| PDIA4 | protein disulfide isomerase family A, member 4 |  |  | 1,95E-11 | -1,900 |  |  |
| PDIA5 | protein disulfide isomerase family A, member 5 |  |  | 1,20E-08 | -1,657 |  |  |
| PDIA6 | protein disulfide isomerase family A, member 6 |  |  | 2,37E-08 | -1,512 |  |  |
| PDLIM1 | PDZ and LIM domain 1 |  |  | 1,83E-12 | -1,626 |  |  |
| PDPR | pyruvate dehydrogenase phosphatase regulatory subunit |  |  | 3,83E-08 | -1,658 |  |  |
| PDS5A | PDS5, regulator of cohesion maintenance, homolog A (S. cerevisiae) |  |  | 6,04E-08 | -1,883 |  |  |
| PDS5B | PDS5, regulator of cohesion maintenance, homolog B (S. cerevisiae) |  |  | 1,69E-14 | -2,024 | 1,69E-14 | -2,505 |
| PDXK | pyridoxal (pyridoxine, vitamin B6) kinase |  |  | 4,49E-14 | -1,997 |  |  |
| PDXK | pyridoxal (pyridoxine, vitamin B6) kinase |  |  | 9,28E-09 | -1,569 |  |  |
| PDZD11 | PDZ domain containing 11 |  |  | 1,35E-08 | -1,581 |  |  |
| PDZD8 | PDZ domain containing 8 |  |  | 1,88E-11 | -2,033 |  |  |
| PDZRN3 | PDZ domain containing ring finger 3 |  |  | 1,69E-11 | -2,084 |  |  |
| PEA15 | phosphoprotein enriched in astrocytes 15 |  |  | 2,74E-12 | -2,037 |  |  |
| PEA15 | phosphoprotein enriched in astrocytes 15 |  |  | 3,85E-11 | -1,681 |  |  |
| PEBP1 | phosphatidylethanolamine binding protein 1 |  |  | 4,82E-12 | -1,707 |  |  |
| PECI | peroxisomal D3,D2-enoyl-CoA isomerase |  |  | 2,51E-10 | -1,643 |  |  |
| PEG10 | paternally expressed 10 |  |  | 4,67E-10 | -1,583 |  |  |
| PELI1 | pellino homolog 1 (Drosophila) |  |  | 1,30E-11 | -1,720 |  |  |
| PERP | PERP, TP53 apoptosis effector |  |  | 9,51E-14 | -1,576 | 9,51E-14 | -1,409 |
| PERP | PERP, TP53 apoptosis effector |  |  | 1,30E-08 | -1,488 |  |  |
| PEX11B | peroxisomal biogenesis factor 11 beta |  |  | 1,24E-08 | -1,684 |  |  |
| PEX3 | peroxisomal biogenesis factor 3 |  |  | 2,71E-13 | -1,820 | 2,71E-13 | -1,487 |
| PEX3 | peroxisomal biogenesis factor 3 |  |  | 3,23E-09 | -1,610 |  |  |
| PFDN4 | prefoldin subunit 4 |  |  | 1,16E-09 | -1,583 |  |  |
| PFDN5 | prefoldin subunit 5 |  |  | 7,72E-08 | -1,513 |  |  |
| PFKM | phosphofructokinase, muscle |  |  | 2,89E-08 | -1,572 |  |  |
| PFN1 | profilin 1 |  |  | 1,12E-09 | -1,572 |  |  |
| PGD | phosphogluconate dehydrogenase |  |  | 9,93E-12 | -1,866 |  |  |
| PGGT1B | protein geranylgeranyltransferase type I, beta subunit |  |  | 3,93E-08 | -1,555 |  |  |
| PGK1 | phosphoglycerate kinase 1 | 1,69E-14 | -1,484 | 1,69E-14 | -1,885 | 1,69E-14 | -1,922 |
| PGK1 | phosphoglycerate kinase 1 |  |  | 1,53E-13 | -1,890 |  |  |
| PHACTR2 | phosphatase and actin regulator 2 |  |  | 1,69E-14 | -1,597 |  |  |
| PHAX | phosphorylated adaptor for RNA export |  |  | 5,13E-13 | -1,763 | 5,13E-13 | -1,581 |
| PHB (includes EG:5245) | prohibitin |  |  | 2,03E-08 | -1,614 |  |  |
| PHB2 | prohibitin 2 |  |  | 2,14E-08 | -1,488 |  |  |
| PHF10 | PHD finger protein 10 |  |  | 5,54E-08 | -1,517 |  |  |
| PHF13 | PHD finger protein 13 |  |  | 8,15E-08 | -1,690 |  |  |
| PHF15 | PHD finger protein 15 |  |  | 3,49E-08 | -1,743 |  |  |
| PHF16 | PHD finger protein 16 |  |  | 3,09E-08 | -1,746 |  |  |
| PHF19 | PHD finger protein 19 |  |  | 9,13E-11 | -2,181 |  |  |
| PHF19 | PHD finger protein 19 |  |  | 1,73E-13 | -2,100 | 1,73E-13 | -1,825 |
| PHF20L1 | PHD finger protein 20-like 1 |  |  | 1,97E-07 | -1,808 |  |  |
| PHF3 | PHD finger protein 3 |  |  | 1,69E-14 | -2,208 | 1,69E-14 | -1,898 |
| PHF5A | PHD finger protein 5A |  |  | 3,55E-09 | -1,539 |  |  |
| PHF8 | PHD finger protein 8 |  |  | 3,64E-10 | -2,607 |  |  |
| PHLDA1 | pleckstrin homology-like domain, family A, member 1 |  |  | 1,69E-14 | -1,869 | 1,69E-14 | -1,401 |
| PHPT1 | phosphohistidine phosphatase 1 |  |  | 9,29E-12 | -1,868 |  |  |
| PI4K2A | phosphatidylinositol 4-kinase type 2 alpha |  |  | 2,90E-10 | -1,917 |  |  |
| PI4KA | phosphatidylinositol 4-kinase, catalytic, alpha |  |  | 1,20E-09 | -2,074 |  |  |
| PI4KB | phosphatidylinositol 4-kinase, catalytic, beta |  |  | 3,91E-11 | -2,647 |  |  |
| PICALM | phosphatidylinositol binding clathrin assembly protein |  |  | 1,69E-14 | -1,794 | 1,69E-14 | -2,091 |
| PIGF | phosphatidylinositol glycan anchor biosynthesis, class F |  |  | 3,39E-08 | -1,681 |  |  |
| PIGY (includes EG:84992) | phosphatidylinositol glycan anchor biosynthesis, class Y |  |  | 1,69E-14 | -1,844 | 1,69E-14 | -1,509 |
| PIK3C2A | phosphoinositide-3-kinase, class 2, alpha polypeptide |  |  |  |  | 6,97E-10 | -1,798 |
| PIK3C2A | phosphoinositide-3-kinase, class 2, alpha polypeptide |  |  | 1,75E-12 | -1,949 |  |  |
| PIN4 | protein (peptidylprolyl cis/trans isomerase) NIMA-interacting, 4 (parvulin) |  |  | 1,07E-11 | -1,975 |  |  |
| PITPNA | phosphatidylinositol transfer protein, alpha |  |  | 8,52E-10 | -1,768 |  |  |
| PITX2 | paired-like homeodomain 2 |  |  |  |  | 2,08E-09 | -1,769 |
| PKP2 | plakophilin 2 |  |  | 1,69E-14 | -1,599 | 1,69E-14 | -1,515 |
| PKP4 | plakophilin 4 |  |  | 1,64E-12 | -1,823 |  |  |
| PL-5283 | PL-5283 protein |  |  | 1,69E-14 | -1,783 | 1,69E-14 | -1,571 |
| PL-5283 | PL-5283 protein |  |  | 2,71E-13 | -1,817 | 2,71E-13 | -1,476 |
| PLA2G2A | phospholipase A2, group IIA (platelets, synovial fluid) |  |  | 1,02E-09 | -1,895 |  |  |
| PLAGL2 | pleiomorphic adenoma gene-like 2 |  |  | 1,98E-09 | -1,785 |  |  |
| PLAUR | plasminogen activator, urokinase receptor |  |  |  |  | 5,52E-12 | 2,368 |
| PLDN | pallidin homolog (mouse) | 1,69E-14 | -1,940 | 1,69E-14 | -1,894 | 1,69E-14 | -1,310 |
| PLDN | pallidin homolog (mouse) |  |  | 1,12E-08 | -1,555 |  |  |
| PLEKHF2 (includes EG:79666) | pleckstrin homology domain containing, family F (with FYVE domain) member 2 |  |  | 5,60E-11 | -1,707 |  |  |
| PLLP | plasma membrane proteolipid (plasmolipin) |  |  | 6,31E-09 | -1,843 |  |  |
| PLOD2 | procollagen-lysine, 2-oxoglutarate 5-dioxygenase 2 |  |  | 1,37E-11 | -1,672 |  |  |
| PLP2 | proteolipid protein 2 (colonic epithelium-enriched) |  |  | 1,25E-08 | -1,658 |  |  |
| PLS3 | plastin 3 (T isoform) |  |  | 5,47E-09 | -1,440 |  |  |
| PLSCR1 | phospholipid scramblase 1 |  |  | 7,40E-10 | -1,546 |  |  |
| PLSCR3 | phospholipid scramblase 3 |  |  | 6,32E-09 | -1,757 |  |  |
| PLXNC1 | plexin C1 |  |  | 1,61E-11 | -1,785 | 1,61E-11 | -1,699 |
| PM20D2 | peptidase M20 domain containing 2 |  |  | 1,69E-14 | -2,047 | 1,69E-14 | -1,517 |
| PMM2 | phosphomannomutase 2 |  |  | 6,60E-09 | -2,004 |  |  |
| PMP22 | peripheral myelin protein 22 |  |  | 1,69E-14 | -2,238 | 1,69E-14 | -1,961 |
| PMPCA | peptidase (mitochondrial processing) alpha |  |  | 5,26E-09 | -1,734 |  |  |
| PMS2L1 | postmeiotic segregation increased 2-like 1 pseudogene |  |  | 2,76E-09 | -2,128 |  |  |
| PMS2L1 | postmeiotic segregation increased 2-like 1 pseudogene |  |  | 7,37E-12 | -2,301 | 7,37E-12 | -1,691 |
| PMS2L1 | postmeiotic segregation increased 2-like 1 pseudogene |  |  | 9,77E-09 | -2,011 | 9,77E-09 | -1,558 |
| PMS2L5 | postmeiotic segregation increased 2-like 5 |  |  | 3,86E-09 | -1,969 |  |  |
| PNKD (includes EG:25953) | paroxysmal nonkinesigenic dyskinesia |  |  | 8,35E-14 | -2,025 | 8,35E-14 | -1,602 |
| PNN | pinin, desmosome associated protein |  |  | 1,64E-06 | -1,389 |  |  |
| PNPO | pyridoxamine 5'-phosphate oxidase |  |  | 5,24E-09 | -1,795 |  |  |
| POLD2 | polymerase (DNA directed), delta 2, regulatory subunit 50kDa |  |  | 1,64E-10 | -1,893 |  |  |
| POLDIP2 | polymerase (DNA-directed), delta interacting protein 2 |  |  | 1,43E-09 | -1,625 |  |  |
| POLDIP2 | polymerase (DNA-directed), delta interacting protein 2 |  |  | 4,79E-11 | -1,819 |  |  |
| POLE3 | polymerase (DNA directed), epsilon 3 (p17 subunit) |  |  | 6,16E-08 | -1,533 | 6,16E-08 | -1,328 |
| POLE4 | polymerase (DNA-directed), epsilon 4 (p12 subunit) |  |  | 6,47E-09 | -1,707 |  |  |
| POLE4 | polymerase (DNA-directed), epsilon 4 (p12 subunit) |  |  | 2,87E-10 | -1,759 |  |  |
| POLR1D | polymerase (RNA) I polypeptide D, 16kDa |  |  | 2,33E-07 | -1,574 |  |  |
| POLR1E | polymerase (RNA) I polypeptide E, 53kDa |  |  | 7,06E-09 | -1,984 |  |  |
| POLR2C | polymerase (RNA) II (DNA directed) polypeptide C, 33kDa | 5,77E-14 | -1,360 | 5,77E-14 | -1,829 | 5,77E-14 | -1,522 |
| POLR2D | polymerase (RNA) II (DNA directed) polypeptide D |  |  | 1,40E-10 | -1,761 |  |  |
| POLR2E | polymerase (RNA) II (DNA directed) polypeptide E, 25kDa | 1,01E-11 | -1,454 | 1,01E-11 | -1,782 |  |  |
| POLR2I | polymerase (RNA) II (DNA directed) polypeptide I, 14.5kDa |  |  | 4,33E-09 | -1,598 |  |  |
| POLR2J | polymerase (RNA) II (DNA directed) polypeptide J, 13.3kDa |  |  | 3,11E-08 | -1,614 |  |  |
| POLR2J2 | polymerase (RNA) II (DNA directed) polypeptide J2 | 9,84E-09 | -2,002 |  |  |  |  |
| POLR2K | polymerase (RNA) II (DNA directed) polypeptide K, 7.0kDa |  |  | 5,19E-09 | -1,585 |  |  |
| POLR2L (includes EG:5441) | polymerase (RNA) II (DNA directed) polypeptide L, 7.6kDa |  |  | 1,69E-14 | -1,859 |  |  |
| POLR3E | polymerase (RNA) III (DNA directed) polypeptide E (80kD) |  |  | 1,99E-10 | -1,644 |  |  |
| POLR3K | polymerase (RNA) III (DNA directed) polypeptide K, 12.3 kDa |  |  | 4,18E-09 | -1,600 |  |  |
| POM121 | POM121 membrane glycoprotein (rat) |  |  | 1,80E-10 | -1,684 |  |  |
| PON2 | paraoxonase 2 |  |  | 2,10E-09 | -1,536 |  |  |
| POP5 | processing of precursor 5, ribonuclease P/MRP subunit (S. cerevisiae) |  |  | 1,52E-10 | -1,620 |  |  |
| PPA2 | pyrophosphatase (inorganic) 2 |  |  | 1,69E-14 | -1,853 | 1,69E-14 | -1,561 |
| PPAP2B | phosphatidic acid phosphatase type 2B |  |  | 1,69E-14 | -2,362 | 1,69E-14 | -2,188 |
| PPAP2C | phosphatidic acid phosphatase type 2C |  |  | 1,64E-08 | -1,944 |  |  |
| PPFIA1 | protein tyrosine phosphatase, receptor type, f polypeptide (PTPRF), interacting protein (liprin), alpha 1 |  |  | 2,92E-09 | -1,579 |  |  |
| PPFIA1 | protein tyrosine phosphatase, receptor type, f polypeptide (PTPRF), interacting protein (liprin), alpha 1 |  |  | 2,38E-09 | -1,852 | 2,38E-09 | -1,656 |
| PPFIBP1 | PTPRF interacting protein, binding protein 1 (liprin beta 1) |  |  |  |  | 2,71E-13 | 3,003 |
| PPFIBP1 | PTPRF interacting protein, binding protein 1 (liprin beta 1) |  |  |  |  | 2,38E-09 | 2,303 |
| PPIA (includes EG:5478) | peptidylprolyl isomerase A (cyclophilin A) |  |  | 6,75E-10 | -1,798 | 6,75E-10 | -1,670 |
| PPIA (includes EG:5478) | peptidylprolyl isomerase A (cyclophilin A) |  |  | 3,80E-09 | -1,673 |  |  |
| PPIC | peptidylprolyl isomerase C (cyclophilin C) |  |  | 2,33E-12 | -1,570 |  |  |
| PPID | peptidylprolyl isomerase D |  |  | 1,25E-08 | -1,522 |  |  |
| PPIE | peptidylprolyl isomerase E (cyclophilin E) |  |  | 3,23E-09 | -1,749 |  |  |
| PPIL4 | peptidylprolyl isomerase (cyclophilin)-like 4 |  |  | 3,49E-08 | -1,667 |  |  |
| PPIL5 | peptidylprolyl isomerase (cyclophilin)-like 5 |  |  | 4,38E-11 | -1,648 |  |  |
| PPME1 | protein phosphatase methylesterase 1 |  |  | 4,03E-10 | -1,863 |  |  |
| PPP1CA | protein phosphatase 1, catalytic subunit, alpha isoform |  |  | 1,59E-08 | -1,609 |  |  |
| PPP1CB | protein phosphatase 1, catalytic subunit, beta isoform |  |  | 1,69E-14 | -1,786 | 1,69E-14 | -2,549 |
| PPP1R11 | protein phosphatase 1, regulatory (inhibitor) subunit 11 |  |  | 2,36E-11 | -1,820 |  |  |
| PPP1R14C | protein phosphatase 1, regulatory (inhibitor) subunit 14C |  |  | 6,20E-09 | -1,858 |  |  |
| PPP1R2 | protein phosphatase 1, regulatory (inhibitor) subunit 2 |  |  | 1,42E-10 | -1,744 |  |  |
| PPP1R2 | protein phosphatase 1, regulatory (inhibitor) subunit 2 |  |  | 4,49E-14 | -1,558 | 4,49E-14 | -1,497 |
| PPP1R7 | protein phosphatase 1, regulatory (inhibitor) subunit 7 |  |  | 7,84E-08 | -1,640 |  |  |
| PPP1R7 | protein phosphatase 1, regulatory (inhibitor) subunit 7 |  |  | 2,69E-08 | -1,681 |  |  |
| PPP2R3C | protein phosphatase 2 (formerly 2A), regulatory subunit B'', gamma |  |  | 3,72E-09 | -1,633 |  |  |
| PPP2R5C | protein phosphatase 2, regulatory subunit B', gamma isoform |  |  | 3,72E-10 | -1,593 | 3,72E-10 | -1,586 |
| PPP2R5D | protein phosphatase 2, regulatory subunit B', delta isoform | 2,88E-10 | -1,572 | 2,88E-10 | -2,301 |  |  |
| PPP2R5E | protein phosphatase 2, regulatory subunit B', epsilon isoform |  |  | 1,90E-10 | -1,640 |  |  |
| PPP3CA | protein phosphatase 3 (formerly 2B), catalytic subunit, alpha isoform |  |  | 1,94E-10 | -1,677 |  |  |
| PPP3CA | protein phosphatase 3 (formerly 2B), catalytic subunit, alpha isoform |  |  | 7,59E-09 | -1,684 |  |  |
| PPP6C | protein phosphatase 6, catalytic subunit |  |  | 1,44E-12 | -1,834 |  |  |
| PQBP1 | polyglutamine binding protein 1 |  |  | 2,16E-10 | -1,698 |  |  |
| PRC1 | protein regulator of cytokinesis 1 |  |  | 2,34E-10 | -1,578 |  |  |
| PRCC | papillary renal cell carcinoma (translocation-associated) |  |  | 2,51E-09 | -2,067 |  |  |
| PRDX2 | peroxiredoxin 2 |  |  | 1,90E-12 | -1,930 | 1,90E-12 | -1,564 |
| PRDX4 | peroxiredoxin 4 |  |  | 1,13E-08 | -1,496 |  |  |
| PRDX5 | peroxiredoxin 5 |  |  | 1,69E-14 | -1,807 |  |  |
| PRDX5 | peroxiredoxin 5 | 1,69E-14 | -1,401 | 1,69E-14 | -1,984 | 1,69E-14 | -1,509 |
| PREB | prolactin regulatory element binding |  |  | 1,06E-08 | -1,824 |  |  |
| PRELID1 | PRELI domain containing 1 |  |  | 2,02E-11 | -1,634 |  |  |
| PRELID1 | PRELI domain containing 1 |  |  | 5,13E-11 | -1,654 |  |  |
| PRIM1 | primase, DNA, polypeptide 1 (49kDa) |  |  | 6,42E-10 | -1,641 |  |  |
| PRKAR2A | protein kinase, cAMP-dependent, regulatory, type II, alpha |  |  | 4,00E-09 | -1,514 |  |  |
| PRKCSH | protein kinase C substrate 80K-H |  |  | 3,14E-11 | -1,970 |  |  |
| PRKCZ | protein kinase C, zeta |  |  |  |  | 4,89E-08 | 2,322 |
| PRKD3 | protein kinase D3 |  |  | 6,80E-10 | -1,587 |  |  |
| PRKDC | protein kinase, DNA-activated, catalytic polypeptide |  |  | 2,59E-10 | -1,551 |  |  |
| PRKDC | protein kinase, DNA-activated, catalytic polypeptide |  |  | 9,51E-14 | -1,846 | 9,51E-14 | -1,473 |
| PRKRA | protein kinase, interferon-inducible double stranded RNA dependent activator |  |  | 1,40E-08 | -1,517 |  |  |
| PRKRIP1 | PRKR interacting protein 1 (IL11 inducible) |  |  | 8,50E-09 | -2,052 |  |  |
| PRMT1 | protein arginine methyltransferase 1 | 1,69E-14 | -1,760 | 1,69E-14 | -2,471 | 1,69E-14 | -1,631 |
| PRMT3 | protein arginine methyltransferase 3 |  |  | 2,32E-12 | -1,676 | 2,32E-12 | -1,414 |
| PRMT5 | protein arginine methyltransferase 5 |  |  | 4,68E-12 | -2,009 |  |  |
| PRNP | prion protein |  |  | 5,11E-09 | -1,662 | 5,11E-09 | -1,757 |
| PROCR | protein C receptor, endothelial (EPCR) |  |  | 1,27E-11 | -1,661 |  |  |
| PROM1 | prominin 1 |  |  | 1,02E-07 | -1,491 |  |  |
| PROSC | proline synthetase co-transcribed homolog (bacterial) |  |  | 4,55E-13 | -1,974 |  |  |
| PROSC | proline synthetase co-transcribed homolog (bacterial) |  |  | 5,76E-13 | -1,899 |  |  |
| PROSC | proline synthetase co-transcribed homolog (bacterial) |  |  | 1,53E-13 | -2,462 |  |  |
| PRPF31 | PRP31 pre-mRNA processing factor 31 homolog (S. cerevisiae) |  |  | 3,58E-08 | -1,646 |  |  |
| PRPF4 | PRP4 pre-mRNA processing factor 4 homolog (yeast) |  |  | 3,71E-11 | -1,874 |  |  |
| PRPF40A | PRP40 pre-mRNA processing factor 40 homolog A (S. cerevisiae) |  |  |  |  | 4,78E-09 | -1,689 |
| PRPF4B | PRP4 pre-mRNA processing factor 4 homolog B (yeast) |  |  | 2,05E-08 | -1,382 |  |  |
| PRPF4B | PRP4 pre-mRNA processing factor 4 homolog B (yeast) |  |  | 2,59E-12 | -1,568 | 2,59E-12 | -1,607 |
| PRPF6 | PRP6 pre-mRNA processing factor 6 homolog (S. cerevisiae) |  |  | 9,05E-09 | -1,823 |  |  |
| PRPF8 | PRP8 pre-mRNA processing factor 8 homolog (S. cerevisiae) |  |  | 4,87E-10 | -1,610 |  |  |
| PRPS2 | phosphoribosyl pyrophosphate synthetase 2 |  |  | 5,76E-11 | -1,683 |  |  |
| PRPS2 | phosphoribosyl pyrophosphate synthetase 2 |  |  | 1,69E-14 | -1,540 | 1,69E-14 | -1,993 |
| PRPSAP1 | phosphoribosyl pyrophosphate synthetase-associated protein 1 |  |  | 7,52E-08 | -1,580 |  |  |
| PRPSAP2 | phosphoribosyl pyrophosphate synthetase-associated protein 2 |  |  | 3,71E-09 | -1,594 |  |  |
| PRR14 | proline rich 14 |  |  | 1,54E-07 | -2,082 |  |  |
| PRR5 | proline rich 5 (renal) |  |  | 1,05E-11 | -1,978 |  |  |
| PRSS1 (includes EG:5644) | protease, serine, 1 (trypsin 1) |  |  | 3,71E-09 | -2,223 |  |  |
| PRSS16 | protease, serine, 16 (thymus) | 1,88E-08 | -2,022 | 1,88E-08 | -1,945 |  |  |
| PRSS35 | protease, serine, 35 |  |  | 3,65E-10 | -1,535 |  |  |
| PRUNE | prune homolog (Drosophila) |  |  | 1,89E-10 | -1,982 |  |  |
| PSAP | prosaposin |  |  | 2,52E-11 | -1,827 |  |  |
| PSAT1 | phosphoserine aminotransferase 1 |  |  | 5,45E-09 | -1,554 |  |  |
| PSEN1 | presenilin 1 |  |  | 4,31E-11 | -1,729 | 4,31E-11 | -1,665 |
| PSIP1 | PC4 and SFRS1 interacting protein 1 |  |  | 3,98E-12 | -1,786 |  |  |
| PSMA2 | proteasome (prosome, macropain) subunit, alpha type, 2 |  |  | 4,64E-09 | -1,712 |  |  |
| PSMC3 | proteasome (prosome, macropain) 26S subunit, ATPase, 3 |  |  | 5,66E-09 | -1,604 |  |  |
| PSMC6 | proteasome (prosome, macropain) 26S subunit, ATPase, 6 |  |  | 8,71E-11 | -1,612 |  |  |
| PSMD1 | proteasome (prosome, macropain) 26S subunit, non-ATPase, 1 |  |  | 7,08E-09 | -1,581 |  |  |
| PSMD10 | proteasome (prosome, macropain) 26S subunit, non-ATPase, 10 |  |  | 1,61E-08 | -1,484 |  |  |
| PSMD10 | proteasome (prosome, macropain) 26S subunit, non-ATPase, 10 |  |  | 7,23E-12 | -1,687 |  |  |
| PSMD12 | proteasome (prosome, macropain) 26S subunit, non-ATPase, 12 |  |  | 2,71E-11 | -1,628 | 2,71E-11 | -1,420 |
| PSMD3 | proteasome (prosome, macropain) 26S subunit, non-ATPase, 3 |  |  | 5,22E-11 | -2,019 |  |  |
| PSMD4 | proteasome (prosome, macropain) 26S subunit, non-ATPase, 4 |  |  | 3,79E-12 | -1,695 |  |  |
| PSMD4 | proteasome (prosome, macropain) 26S subunit, non-ATPase, 4 |  |  | 5,13E-09 | -1,682 |  |  |
| PSMD4 | proteasome (prosome, macropain) 26S subunit, non-ATPase, 4 |  |  | 1,64E-07 | -1,561 |  |  |
| PSMD5 | proteasome (prosome, macropain) 26S subunit, non-ATPase, 5 |  |  | 5,27E-08 | -1,697 |  |  |
| PSMD7 | proteasome (prosome, macropain) 26S subunit, non-ATPase, 7 |  |  | 1,69E-14 | -1,913 |  |  |
| PSMD8 | proteasome (prosome, macropain) 26S subunit, non-ATPase, 8 |  |  | 2,10E-08 | -1,675 |  |  |
| PSMD9 | proteasome (prosome, macropain) 26S subunit, non-ATPase, 9 |  |  | 2,92E-08 | -1,607 |  |  |
| PSME2 | proteasome (prosome, macropain) activator subunit 2 (PA28 beta) |  |  | 1,22E-08 | -1,562 |  |  |
| PSME3 | proteasome (prosome, macropain) activator subunit 3 (PA28 gamma; Ki) |  |  | 3,42E-08 | -1,721 |  |  |
| PSME4 | proteasome (prosome, macropain) activator subunit 4 |  |  |  |  | 9,19E-12 | 1,457 |
| PSMF1 | proteasome (prosome, macropain) inhibitor subunit 1 (PI31) |  |  | 2,11E-10 | -1,914 |  |  |
| PSMG2 | proteasome (prosome, macropain) assembly chaperone 2 |  |  | 5,94E-10 | -1,631 |  |  |
| PSRC1 | proline/serine-rich coiled-coil 1 |  |  | 9,96E-12 | -2,040 | 9,96E-12 | -1,600 |
| PTBP1 | polypyrimidine tract binding protein 1 | 5,77E-14 | -1,587 | 5,77E-14 | -2,343 | 5,77E-14 | -2,049 |
| PTDSS1 | phosphatidylserine synthase 1 |  |  | 6,49E-10 | -1,720 |  |  |
| PTPLA | protein tyrosine phosphatase-like (proline instead of catalytic arginine), member A |  |  | 5,75E-10 | -1,801 |  |  |
| PTPLB | protein tyrosine phosphatase-like (proline instead of catalytic arginine), member b |  |  | 8,38E-09 | -1,475 |  |  |
| PTPLB | protein tyrosine phosphatase-like (proline instead of catalytic arginine), member b |  |  | 1,69E-14 | 1,542 | 1,69E-14 | 1,586 |
| PTPMT1 | protein tyrosine phosphatase, mitochondrial 1 |  |  | 3,75E-08 | -1,687 |  |  |
| PTPN18 | protein tyrosine phosphatase, non-receptor type 18 (brain-derived) |  |  | 1,69E-14 | -2,684 | 1,69E-14 | -1,929 |
| PTPRF | protein tyrosine phosphatase, receptor type, F |  |  |  |  | 9,98E-10 | 1,728 |
| PTPRF | protein tyrosine phosphatase, receptor type, F |  |  | 3,49E-13 | -1,943 |  |  |
| PTRH2 | peptidyl-tRNA hydrolase 2 |  |  | 7,44E-09 | -1,555 |  |  |
| PTS | 6-pyruvoyltetrahydropterin synthase |  |  | 1,47E-08 | -1,555 |  |  |
| PTTG1 | pituitary tumor-transforming 1 |  |  | 9,27E-10 | -1,525 |  |  |
| PWP1 | PWP1 homolog (S. cerevisiae) |  |  | 2,71E-08 | -1,519 |  |  |
| PWWP2A | PWWP domain containing 2A |  |  | 1,13E-10 | -1,768 |  |  |
| PXMP3 | peroxisomal membrane protein 3, 35kDa |  |  | 1,58E-09 | -1,707 | 1,58E-09 | -1,573 |
| PXMP4 | peroxisomal membrane protein 4, 24kDa |  |  | 6,95E-12 | -1,850 | 6,95E-12 | -1,653 |
| PYGL | phosphorylase, glycogen, liver |  |  | 8,74E-10 | -1,642 |  |  |
| QKI | quaking homolog, KH domain RNA binding (mouse) | 4,02E-09 | -1,422 |  |  |  |  |
| QKI | quaking homolog, KH domain RNA binding (mouse) |  |  | 2,84E-11 | -1,495 |  |  |
| QPRT | quinolinate phosphoribosyltransferase |  |  | 1,97E-07 | -1,521 |  |  |
| QRICH1 | glutamine-rich 1 |  |  | 5,22E-12 | -1,873 |  |  |
| QSOX2 | quiescin Q6 sulfhydryl oxidase 2 | 7,84E-08 | -1,845 |  |  |  |  |
| RAB11FIP1 | RAB11 family interacting protein 1 (class I) |  |  | 1,07E-12 | -1,953 |  |  |
| RAB11FIP2 | RAB11 family interacting protein 2 (class I) |  |  | 9,63E-09 | -1,609 |  |  |
| RAB11FIP2 | RAB11 family interacting protein 2 (class I) |  |  | 3,29E-10 | -1,748 |  |  |
| RAB12 | RAB12, member RAS oncogene family |  |  | 1,69E-14 | -2,317 |  |  |
| RAB13 | RAB13, member RAS oncogene family |  |  | 3,95E-10 | -1,755 |  |  |
| RAB1A | RAB1A, member RAS oncogene family |  |  | 1,47E-09 | -1,445 | 1,47E-09 | -1,447 |
| RAB21 | RAB21, member RAS oncogene family |  |  | 6,22E-10 | -1,670 |  |  |
| RAB22A | RAB22A, member RAS oncogene family |  |  | 6,69E-08 | -1,599 |  |  |
| RAB23 | RAB23, member RAS oncogene family |  |  |  |  | 1,11E-08 | -2,189 |
| RAB25 | RAB25, member RAS oncogene family |  |  | 4,77E-12 | -1,818 |  |  |
| RAB4A | RAB4A, member RAS oncogene family |  |  | 7,33E-13 | -1,782 | 7,33E-13 | -1,518 |
| RAB5A | RAB5A, member RAS oncogene family |  |  |  |  | 2,39E-10 | -1,880 |
| RAB5A | RAB5A, member RAS oncogene family |  |  | 5,69E-08 | -1,515 |  |  |
| RAB5C | RAB5C, member RAS oncogene family |  |  | 7,58E-11 | -1,898 |  |  |
| RAB5C | RAB5C, member RAS oncogene family |  |  | 9,43E-13 | -1,828 | 9,43E-13 | -1,575 |
| RAB7A | RAB7A, member RAS oncogene family |  |  | 1,56E-12 | -1,678 | 1,56E-12 | -1,394 |
| RAB7A | RAB7A, member RAS oncogene family |  |  | 7,65E-10 | -1,672 |  |  |
| RABAC1 | Rab acceptor 1 (prenylated) |  |  | 3,14E-08 | -1,905 |  |  |
| RABEP2 | rabaptin, RAB GTPase binding effector protein 2 |  |  | 2,63E-09 | -1,651 |  |  |
| RABGAP1 | RAB GTPase activating protein 1 |  |  | 1,69E-14 | -2,151 |  |  |
| RAC1 | ras-related C3 botulinum toxin substrate 1 (rho family, small GTP binding protein Rac1) |  |  | 6,48E-11 | -1,584 |  |  |
| RAC1 | ras-related C3 botulinum toxin substrate 1 (rho family, small GTP binding protein Rac1) |  |  | 2,43E-08 | -1,611 |  |  |
| RAD17 | RAD17 homolog (S. pombe) |  |  | 1,29E-12 | -1,731 |  |  |
| RAD23B | RAD23 homolog B (S. cerevisiae) |  |  | 3,05E-09 | -1,620 |  |  |
| RAD51 | RAD51 homolog (RecA homolog, E. coli) (S. cerevisiae) |  |  | 3,24E-09 | -1,950 |  |  |
| RAD51AP1 | RAD51 associated protein 1 |  |  | 1,08E-09 | -1,533 |  |  |
| RAE1 | RAE1 RNA export 1 homolog (S. pombe) |  |  | 1,36E-09 | -1,583 |  |  |
| RAG1AP1 | recombination activating gene 1 activating protein 1 | 5,47E-12 | -1,997 | 5,47E-12 | -1,707 | 5,47E-12 | -2,010 |
| RALA | v-ral simian leukemia viral oncogene homolog A (ras related) |  |  | 2,95E-10 | -1,645 |  |  |
| RALB | v-ral simian leukemia viral oncogene homolog B (ras related; GTP binding protein) |  |  | 4,17E-12 | -1,931 |  |  |
| RALY | RNA binding protein, autoantigenic (hnRNP-associated with lethal yellow homolog (mouse)) |  |  | 7,67E-11 | -1,741 |  |  |
| RAN | RAN, member RAS oncogene family |  |  | 1,67E-10 | -1,564 |  |  |
| RAN | RAN, member RAS oncogene family |  |  | 7,13E-08 | -1,446 |  |  |
| RANBP2 | RAN binding protein 2 |  |  |  |  | 1,42E-11 | 1,469 |
| RANBP2 | RAN binding protein 2 |  |  | 1,47E-12 | -1,650 |  |  |
| RANBP9 | RAN binding protein 9 |  |  | 1,72E-11 | -1,783 |  |  |
| RANGAP1 | Ran GTPase activating protein 1 |  |  | 6,94E-09 | -2,476 |  |  |
| RAP1A | RAP1A, member of RAS oncogene family |  |  | 1,69E-14 | -1,857 | 1,69E-14 | -1,648 |
| RAP2B | RAP2B, member of RAS oncogene family |  |  | 1,80E-11 | -1,734 | 1,80E-11 | -1,961 |
| RAPGEF2 | Rap guanine nucleotide exchange factor (GEF) 2 |  |  | 4,24E-08 | -1,508 |  |  |
| RASA1 | RAS p21 protein activator (GTPase activating protein) 1 |  |  | 1,99E-09 | -1,532 | 1,99E-09 | -1,465 |
| RASGRF2 | Ras protein-specific guanine nucleotide-releasing factor 2 |  |  | 1,06E-08 | -2,098 |  |  |
| RB1CC1 | RB1-inducible coiled-coil 1 |  |  | 2,29E-11 | -1,531 |  |  |
| RBBP8 (includes EG:5932) | retinoblastoma binding protein 8 |  |  | 4,84E-12 | -1,737 |  |  |
| RBM10 | RNA binding motif protein 10 |  |  | 3,44E-10 | -2,099 |  |  |
| RBM14 | RNA binding motif protein 14 | 8,58E-09 | -1,719 | 8,58E-09 | -2,069 |  |  |
| RBM16 | RNA binding motif protein 16 |  |  | 1,69E-14 | -1,766 |  |  |
| RBM17 | RNA binding motif protein 17 |  |  | 1,04E-08 | -2,145 |  |  |
| RBM22 | RNA binding motif protein 22 |  |  | 1,68E-08 | -1,710 |  |  |
| RBM22 | RNA binding motif protein 22 |  |  | 1,69E-14 | -2,011 |  |  |
| RBM25 (includes EG:58517) | RNA binding motif protein 25 |  |  |  |  | 1,69E-14 | 1,754 |
| RBM25 (includes EG:58517) | RNA binding motif protein 25 |  |  |  |  | 1,69E-14 | 1,740 |
| RBM27 | RNA binding motif protein 27 |  |  | 2,91E-13 | -1,764 |  |  |
| RBM3 | RNA binding motif (RNP1, RRM) protein 3 |  |  | 4,55E-13 | -1,827 |  |  |
| RBM33 | RNA binding motif protein 33 |  |  | 6,56E-13 | -1,895 |  |  |
| RBM35A | RNA binding motif protein 35A |  |  | 1,62E-10 | -1,604 | 1,62E-10 | -1,575 |
| RBM39 | RNA binding motif protein 39 |  |  | 2,90E-09 | -1,455 |  |  |
| RBM39 | RNA binding motif protein 39 |  |  |  |  | 1,69E-14 | 2,235 |
| RBM42 | RNA binding motif protein 42 |  |  | 1,19E-08 | -1,974 |  |  |
| RBM47 | RNA binding motif protein 47 |  |  | 2,18E-09 | -1,652 |  |  |
| RBM47 | RNA binding motif protein 47 |  |  | 3,43E-09 | -1,708 |  |  |
| RBM6 | RNA binding motif protein 6 |  |  | 1,42E-09 | -1,878 |  |  |
| RBM8A | RNA binding motif protein 8A |  |  |  |  | 1,55E-07 | 2,530 |
| RBM8A | RNA binding motif protein 8A |  |  | 1,66E-09 | -1,752 |  |  |
| RBM9 | RNA binding motif protein 9 | 3,14E-14 | -1,522 | 3,14E-14 | -2,222 | 3,14E-14 | -1,377 |
| RBMS1 | RNA binding motif, single stranded interacting protein 1 |  |  | 4,90E-08 | -1,644 |  |  |
| RBMS1 | RNA binding motif, single stranded interacting protein 1 |  |  | 1,57E-09 | -1,679 |  |  |
| RBMX | RNA binding motif protein, X-linked |  |  | 1,49E-12 | -1,788 |  |  |
| RBMX2 | RNA binding motif protein, X-linked 2 |  |  | 2,28E-12 | -2,375 |  |  |
| RBP4 | retinol binding protein 4, plasma |  |  | 1,78E-09 | -1,735 |  |  |
| RBPMS | RNA binding protein with multiple splicing |  |  | 3,87E-12 | -1,657 | 3,87E-12 | -1,672 |
| RBX1 (includes EG:9978) | ring-box 1 |  |  | 1,02E-09 | -1,582 |  |  |
| RC3H2 | ring finger and CCCH-type zinc finger domains 2 |  |  | 1,15E-11 | -1,934 |  |  |
| RCN2 | reticulocalbin 2, EF-hand calcium binding domain |  |  | 3,97E-09 | -1,656 |  |  |
| RCOR3 (includes EG:55758) | REST corepressor 3 |  |  | 3,66E-09 | -1,697 |  |  |
| RDH10 | retinol dehydrogenase 10 (all-trans) |  |  | 1,72E-11 | -2,010 |  |  |
| RDX | radixin |  |  | 1,03E-10 | -1,765 | 1,03E-10 | -1,515 |
| RECQL | RecQ protein-like (DNA helicase Q1-like) | 1,69E-14 | -1,313 | 1,69E-14 | -1,989 | 1,69E-14 | -1,420 |
| RECQL | RecQ protein-like (DNA helicase Q1-like) |  |  | 2,39E-09 | -1,630 |  |  |
| REEP4 | receptor accessory protein 4 |  |  | 8,05E-09 | -2,134 |  |  |
| REEP5 | receptor accessory protein 5 |  |  | 1,69E-14 | -1,707 | 1,69E-14 | -1,387 |
| REEP5 | receptor accessory protein 5 |  |  | 7,67E-09 | -1,548 |  |  |
| REEP6 | receptor accessory protein 6 |  |  | 4,17E-08 | -1,831 |  |  |
| REG4 | regenerating islet-derived family, member 4 |  |  | 5,09E-08 | -2,152 |  |  |
| REPIN1 | replication initiator 1 | 1,18E-09 | -1,373 | 1,18E-09 | -1,694 |  |  |
| REPS1 | RALBP1 associated Eps domain containing 1 |  |  | 2,16E-09 | -1,667 |  |  |
| RERE | arginine-glutamic acid dipeptide (RE) repeats |  |  | 8,20E-10 | -1,992 |  |  |
| REXO2 | REX2, RNA exonuclease 2 homolog (S. cerevisiae) |  |  | 1,82E-11 | -1,751 | 1,82E-11 | -2,016 |
| REXO4 | REX4, RNA exonuclease 4 homolog (S. cerevisiae) |  |  | 3,55E-11 | -2,088 |  |  |
| RFC2 | replication factor C (activator 1) 2, 40kDa |  |  | 3,31E-10 | -1,718 |  |  |
| RFC3 | replication factor C (activator 1) 3, 38kDa |  |  | 1,15E-08 | -1,548 |  |  |
| RFC3 | replication factor C (activator 1) 3, 38kDa |  |  | 1,21E-11 | -1,552 | 1,21E-11 | -1,770 |
| RFC5 | replication factor C (activator 1) 5, 36.5kDa |  |  | 1,60E-08 | -1,534 |  |  |
| RFC5 | replication factor C (activator 1) 5, 36.5kDa |  |  | 8,15E-10 | -1,723 | 8,15E-10 | -1,513 |
| RFFL | ring finger and FYVE-like domain containing 1 |  |  | 3,86E-09 | -1,540 |  |  |
| RFT1 | RFT1 homolog (S. cerevisiae) |  |  | 4,56E-09 | -1,848 |  |  |
| RFWD2 | ring finger and WD repeat domain 2 |  |  | 2,32E-08 | -1,719 |  |  |
| RFX7 | regulatory factor X, 7 |  |  | 7,61E-09 | -1,617 |  |  |
| RGN | regucalcin (senescence marker protein-30) | 3,79E-12 | -1,596 | 3,79E-12 | -2,097 | 3,79E-12 | -1,967 |
| RHBDD2 | rhomboid domain containing 2 |  |  | 1,59E-10 | -2,689 | 1,59E-10 | -1,993 |
| RHEB | Ras homolog enriched in brain | 3,00E-13 | -1,364 | 3,00E-13 | -1,928 |  |  |
| RHOA | ras homolog gene family, member A | 1,69E-14 | -1,369 | 1,69E-14 | -1,670 | 1,69E-14 | -1,656 |
| RHOBTB3 | Rho-related BTB domain containing 3 |  |  | 7,78E-10 | -1,935 |  |  |
| RHOBTB3 | Rho-related BTB domain containing 3 |  |  | 2,88E-11 | -1,626 | 2,88E-11 | -1,404 |
| RHOC | ras homolog gene family, member C |  |  | 5,30E-11 | -1,789 |  |  |
| RHOF | ras homolog gene family, member F (in filopodia) |  |  | 2,81E-10 | -2,205 |  |  |
| RIF1 | RAP1 interacting factor homolog (yeast) |  |  |  |  | 1,69E-14 | 2,078 |
| RIN2 | Ras and Rab interactor 2 |  |  | 2,10E-08 | -1,527 |  |  |
| RING1 | ring finger protein 1 |  |  | 6,46E-09 | -1,793 |  |  |
| RMND1 | required for meiotic nuclear division 1 homolog (S. cerevisiae) | 2,75E-07 | -1,521 |  |  |  |  |
| RMND5B | required for meiotic nuclear division 5 homolog B (S. cerevisiae) |  |  | 3,94E-09 | -1,910 |  |  |
| RNASEH2A | ribonuclease H2, subunit A |  |  | 4,65E-08 | -1,601 |  |  |
| RNASEK | ribonuclease, RNase K |  |  | 4,43E-09 | -1,660 |  |  |
| RNASEN | ribonuclease type III, nuclear |  |  | 7,10E-10 | -1,692 |  |  |
| RNASET2 | ribonuclease T2 |  |  | 1,41E-09 | -1,748 |  |  |
| RNASET2 | ribonuclease T2 |  |  | 4,42E-09 | -1,735 |  |  |
| RNF10 | ring finger protein 10 |  |  | 3,99E-09 | -1,675 |  |  |
| RNF114 | ring finger protein 114 |  |  | 1,74E-07 | -1,518 |  |  |
| RNF114 | ring finger protein 114 |  |  | 3,35E-08 | -1,480 |  |  |
| RNF14 | ring finger protein 14 |  |  |  |  | 2,16E-08 | -1,390 |
| RNF167 | ring finger protein 167 |  |  | 1,03E-08 | -1,996 |  |  |
| RNF170 (includes EG:81790) | ring finger protein 170 |  |  | 2,69E-11 | -1,997 |  |  |
| RNF181 | ring finger protein 181 |  |  | 5,34E-09 | -1,604 |  |  |
| RNF216 | ring finger protein 216 |  |  | 2,72E-08 | -3,017 |  |  |
| RNF26 | ring finger protein 26 |  |  | 1,80E-08 | -1,757 |  |  |
| RNF4 | ring finger protein 4 |  |  | 1,98E-11 | -1,907 |  |  |
| RNF40 | ring finger protein 40 |  |  | 2,04E-09 | -2,304 |  |  |
| RNF5 (includes EG:6048) | ring finger protein 5 |  |  | 1,69E-14 | -1,976 | 1,69E-14 | -1,615 |
| RNF6 | ring finger protein (C3H2C3 type) 6 |  |  | 7,07E-14 | -1,645 | 7,07E-14 | -1,413 |
| RNF7 | ring finger protein 7 |  |  | 5,22E-13 | -1,775 |  |  |
| RNF7 | ring finger protein 7 | 6,81E-13 | -1,344 | 6,81E-13 | -1,909 | 6,81E-13 | -1,523 |
| RNGTT | RNA guanylyltransferase and 5'-phosphatase |  |  | 2,27E-08 | -1,549 |  |  |
| RNMT | RNA (guanine-7-) methyltransferase |  |  | 1,36E-08 | -1,717 |  |  |
| RNPEP | arginyl aminopeptidase (aminopeptidase B) |  |  | 4,14E-11 | -1,702 |  |  |
| ROCK2 | Rho-associated, coiled-coil containing protein kinase 2 |  |  | 2,01E-07 | -1,494 |  |  |
| RP3-402G11.5 | selenoprotein O |  |  | 9,63E-08 | -1,908 |  |  |
| RP5-1077B9.4 | invasion inhibitory protein 45 |  |  | 3,72E-09 | -1,681 |  |  |
| RP6-213H19.1 | serine/threonine protein kinase MST4 |  |  | 1,26E-11 | -1,633 | 1,26E-11 | -2,168 |
| RP9 | retinitis pigmentosa 9 (autosomal dominant) |  |  | 1,66E-11 | -2,278 |  |  |
| RP9 | retinitis pigmentosa 9 (autosomal dominant) |  |  | 8,05E-08 | -1,605 |  |  |
| RPA2 | replication protein A2, 32kDa |  |  | 2,93E-11 | -1,696 |  |  |
| RPA3 | replication protein A3, 14kDa |  |  | 7,15E-11 | -1,603 |  |  |
| RPAIN | RPA interacting protein |  |  | 1,08E-09 | -1,766 |  |  |
| RPE | ribulose-5-phosphate-3-epimerase | 1,90E-10 | -1,333 | 1,90E-10 | -1,621 | 1,90E-10 | -1,630 |
| RPIA | ribose 5-phosphate isomerase A |  |  | 2,95E-10 | -1,652 |  |  |
| RPL22 | ribosomal protein L22 |  |  | 1,01E-10 | -1,662 |  |  |
| RPL22 | ribosomal protein L22 |  |  | 6,77E-08 | -1,516 |  |  |
| RPL26L1 | ribosomal protein L26-like 1 |  |  | 5,61E-08 | -1,555 |  |  |
| RPL31 | ribosomal protein L31 |  |  | 7,07E-10 | -1,511 |  |  |
| RPL35A | ribosomal protein L35a |  |  | 1,84E-10 | -1,485 |  |  |
| RPL36 (includes EG:25873) | ribosomal protein L36 |  |  | 1,83E-10 | -1,570 |  |  |
| RPL38 (includes EG:6169) | ribosomal protein L38 |  |  | 5,64E-13 | 1,796 |  |  |
| RPL7L1 | ribosomal protein L7-like 1 |  |  | 8,07E-10 | -1,675 |  |  |
| RPS10 | ribosomal protein S10 |  |  | 4,55E-13 | -2,538 | 4,55E-13 | -2,423 |
| RPS17L4 | ribosomal protein S17-like 4 |  |  | 1,69E-14 | -1,688 | 1,69E-14 | -2,148 |
| RPS19BP1 | ribosomal protein S19 binding protein 1 |  |  | 9,56E-12 | -1,840 |  |  |
| RPS23 | ribosomal protein S23 |  |  | 4,95E-09 | -1,959 | 4,95E-09 | -1,773 |
| RPS27A | ribosomal protein S27a | 1,86E-10 | -1,987 | 1,86E-10 | -4,163 | 1,86E-10 | -2,747 |
| RPS27L (includes EG:51065) | ribosomal protein S27-like |  |  | 3,41E-09 | -1,589 |  |  |
| RPS6KA4 | ribosomal protein S6 kinase, 90kDa, polypeptide 4 |  |  | 8,78E-09 | -2,325 |  |  |
| RQCD1 | RCD1 required for cell differentiation1 homolog (S. pombe) |  |  | 1,62E-12 | -1,831 |  |  |
| RRBP1 | ribosome binding protein 1 homolog 180kDa (dog) |  |  | 1,69E-14 | -2,120 |  |  |
| RRM1 | ribonucleotide reductase M1 |  |  |  |  | 3,47E-08 | -1,576 |
| RRM1 | ribonucleotide reductase M1 |  |  | 1,85E-12 | -1,677 |  |  |
| RRP1 | ribosomal RNA processing 1 homolog (S. cerevisiae) |  |  | 2,61E-09 | -2,413 |  |  |
| RRP7A | ribosomal RNA processing 7 homolog A (S. cerevisiae) |  |  | 5,80E-09 | -1,762 |  |  |
| RSF1 | remodeling and spacing factor 1 |  |  |  |  | 1,69E-14 | 2,074 |
| RSPRY1 | ring finger and SPRY domain containing 1 |  |  | 2,39E-09 | -1,717 |  |  |
| RTF1 | Rtf1, Paf1/RNA polymerase II complex component, homolog (S. cerevisiae) |  |  | 2,23E-08 | -1,646 |  |  |
| RTN3 | reticulon 3 |  |  | 2,08E-12 | -1,806 |  |  |
| RTN4 | reticulon 4 |  |  | 3,69E-09 | -1,463 |  |  |
| RUVBL2 | RuvB-like 2 (E. coli) |  |  | 3,64E-10 | -1,636 |  |  |
| RWDD1 (includes EG:51389) | RWD domain containing 1 |  |  | 4,55E-13 | -1,755 |  |  |
| RWDD3 | RWD domain containing 3 |  |  | 1,36E-09 | -1,762 |  |  |
| S100A11 | S100 calcium binding protein A11 |  |  | 1,72E-07 | -1,626 |  |  |
| S100A13 | S100 calcium binding protein A13 |  |  | 1,08E-10 | -1,952 |  |  |
| S100A14 | S100 calcium binding protein A14 |  |  | 6,46E-08 | -1,895 |  |  |
| S100A6 | S100 calcium binding protein A6 |  |  | 2,36E-13 | -1,978 |  |  |
| SAAL1 | serum amyloid A-like 1 |  |  | 2,28E-08 | -1,514 |  |  |
| SAE1 | SUMO1 activating enzyme subunit 1 | 1,69E-14 | -1,425 | 1,69E-14 | -2,023 | 1,69E-14 | -1,508 |
| SAFB2 | scaffold attachment factor B2 |  |  | 3,02E-09 | -1,535 |  |  |
| SAPS1 | SAPS domain family, member 1 |  |  | 8,20E-09 | -1,835 |  |  |
| SAPS3 | SAPS domain family, member 3 |  |  | 1,02E-12 | -1,843 |  |  |
| SAPS3 | SAPS domain family, member 3 |  |  | 4,49E-11 | -1,615 |  |  |
| SAR1B | SAR1 homolog B (S. cerevisiae) |  |  | 1,04E-12 | -1,619 | 1,04E-12 | -1,511 |
| SARS | seryl-tRNA synthetase |  |  | 9,20E-10 | -1,794 |  |  |
| SAT1 | spermidine/spermine N1-acetyltransferase 1 |  |  | 1,49E-08 | -1,495 |  |  |
| SAT2 | spermidine/spermine N1-acetyltransferase family member 2 |  |  | 4,29E-10 | -2,096 |  |  |
| SC4MOL | sterol-C4-methyl oxidase-like |  |  | 1,41E-07 | -1,464 |  |  |
| SCAND1 | SCAN domain containing 1 |  |  | 1,21E-08 | -1,690 |  |  |
| SCAND1 | SCAN domain containing 1 |  |  | 3,90E-08 | -1,685 |  |  |
| SCAND1 | SCAN domain containing 1 |  |  | 2,36E-13 | -2,033 |  |  |
| SCARA3 | scavenger receptor class A, member 3 |  |  | 1,35E-08 | -2,656 |  |  |
| SCARB1 | scavenger receptor class B, member 1 |  |  | 8,89E-11 | -1,957 |  |  |
| SCARB2 | scavenger receptor class B, member 2 |  |  | 4,00E-11 | -1,658 |  |  |
| SCD | stearoyl-CoA desaturase (delta-9-desaturase) |  |  | 1,87E-08 | -1,747 |  |  |
| SCD | stearoyl-CoA desaturase (delta-9-desaturase) |  |  | 2,39E-10 | -1,967 | 2,39E-10 | -2,462 |
| SCML1 | sex comb on midleg-like 1 (Drosophila) |  |  | 1,69E-14 | -1,746 |  |  |
| SCO1 | SCO cytochrome oxidase deficient homolog 1 (yeast) |  |  | 1,89E-10 | -2,054 |  |  |
| SCOC | short coiled-coil protein |  |  | 1,69E-14 | -1,525 | 1,69E-14 | -2,009 |
| SCOC | short coiled-coil protein |  |  | 2,16E-10 | -1,481 | 2,16E-10 | -1,391 |
| SCRN2 | secernin 2 |  |  | 2,98E-08 | -1,825 |  |  |
| SCRN3 | secernin 3 |  |  | 1,88E-11 | -1,642 | 1,88E-11 | -1,439 |
| SCYE1 | small inducible cytokine subfamily E, member 1 (endothelial monocyte-activating) |  |  | 2,15E-09 | -1,547 |  |  |
| SCYE1 | small inducible cytokine subfamily E, member 1 (endothelial monocyte-activating) |  |  | 1,73E-13 | -1,715 |  |  |
| SCYL2 | SCY1-like 2 (S. cerevisiae) |  |  | 8,35E-14 | -1,588 | 8,35E-14 | -1,521 |
| SCYL2 | SCY1-like 2 (S. cerevisiae) |  |  | 4,49E-14 | -1,821 |  |  |
| SDC1 | syndecan 1 |  |  | 1,64E-11 | -2,440 |  |  |
| SDC4 | syndecan 4 |  |  | 1,69E-14 | -1,535 | 1,69E-14 | 1,296 |
| SDF4 | stromal cell derived factor 4 |  |  | 3,62E-09 | -1,794 |  |  |
| SDF4 | stromal cell derived factor 4 | 3,17E-12 | -1,643 | 3,17E-12 | -1,994 |  |  |
| SDHB | succinate dehydrogenase complex, subunit B, iron sulfur (Ip) | 1,72E-08 | -1,412 |  |  | 1,72E-08 | -1,722 |
| SDHC | succinate dehydrogenase complex, subunit C, integral membrane protein, 15kDa | 1,69E-14 | -1,683 | 1,69E-14 | -2,743 | 1,69E-14 | -1,864 |
| SDHC | succinate dehydrogenase complex, subunit C, integral membrane protein, 15kDa | 1,69E-14 | -2,180 | 1,69E-14 | -3,735 | 1,69E-14 | -2,382 |
| SDHC | succinate dehydrogenase complex, subunit C, integral membrane protein, 15kDa |  |  | 7,95E-13 | -2,016 |  |  |
| SDHC | succinate dehydrogenase complex, subunit C, integral membrane protein, 15kDa | 1,69E-14 | -2,849 | 1,69E-14 | -5,238 | 1,69E-14 | -4,179 |
| SEC11A | SEC11 homolog A (S. cerevisiae) |  |  | 1,48E-07 | -1,446 |  |  |
| SEC11C | SEC11 homolog C (S. cerevisiae) |  |  | 1,09E-07 | -1,809 |  |  |
| SEC14L1 | SEC14-like 1 (S. cerevisiae) |  |  |  |  | 5,76E-12 | 1,763 |
| SEC16A | SEC16 homolog A (S. cerevisiae) |  |  | 7,55E-10 | -1,619 |  |  |
| SEC22A | SEC22 vesicle trafficking protein homolog A (S. cerevisiae) |  |  | 1,97E-10 | -1,964 |  |  |
| SEC22B | SEC22 vesicle trafficking protein homolog B (S. cerevisiae) |  |  |  |  | 9,58E-10 | -2,055 |
| SEC23B | Sec23 homolog B (S. cerevisiae) |  |  | 7,59E-10 | -1,640 |  |  |
| SEC23IP | SEC23 interacting protein |  |  | 3,14E-14 | -1,830 |  |  |
| SEC24A | SEC24 family, member A (S. cerevisiae) |  |  | 7,98E-09 | -1,657 |  |  |
| SEC24C (includes EG:9632) | SEC24 family, member C (S. cerevisiae) |  |  | 1,69E-14 | -2,518 |  |  |
| SEC31A | SEC31 homolog A (S. cerevisiae) |  |  | 1,95E-13 | -1,780 |  |  |
| SEC31A | SEC31 homolog A (S. cerevisiae) |  |  | 2,56E-08 | -1,677 |  |  |
| SEC61A1 | Sec61 alpha 1 subunit (S. cerevisiae) |  |  | 1,69E-14 | -2,044 |  |  |
| SEC61A1 | Sec61 alpha 1 subunit (S. cerevisiae) |  |  | 3,49E-08 | -1,938 |  |  |
| SEC63 | SEC63 homolog (S. cerevisiae) |  |  | 3,14E-14 | -1,785 |  |  |
| SEL1L | sel-1 suppressor of lin-12-like (C. elegans) |  |  | 4,67E-11 | -1,649 |  |  |
| SELK | selenoprotein K |  |  | 3,49E-11 | -1,633 | 3,49E-11 | -1,433 |
| SELS | selenoprotein S |  |  | 2,73E-08 | -1,523 |  |  |
| SEMA3A | sema domain, immunoglobulin domain (Ig), short basic domain, secreted, (semaphorin) 3A |  |  | 2,13E-07 | -1,977 |  |  |
| SENP3 | SUMO1/sentrin/SMT3 specific peptidase 3 |  |  | 8,39E-11 | -2,204 |  |  |
| SENP3 | SUMO1/sentrin/SMT3 specific peptidase 3 |  |  | 1,59E-08 | -2,223 |  |  |
| SEP15 | 15 kDa selenoprotein | 1,69E-14 | -1,390 | 1,69E-14 | -1,764 | 1,69E-14 | -1,545 |
| SEPT11 | septin 11 |  |  | 1,75E-09 | -1,601 |  |  |
| SEPT2 | septin 2 |  |  | 5,77E-14 | -1,697 | 5,77E-14 | -1,901 |
| SEPT2 | septin 2 |  |  | 1,69E-14 | -1,696 | 1,69E-14 | -1,668 |
| SEPT9 | septin 9 |  |  | 4,72E-13 | -3,409 | 4,72E-13 | -1,595 |
| SERBP1 | SERPINE1 mRNA binding protein 1 |  |  | 4,33E-09 | -1,523 |  |  |
| SERBP1 | SERPINE1 mRNA binding protein 1 |  |  | 7,92E-12 | -1,650 | 7,92E-12 | -1,449 |
| SERINC1 | serine incorporator 1 |  |  | 2,34E-08 | -1,503 |  |  |
| SERINC3 | serine incorporator 3 |  |  | 1,69E-10 | -1,668 |  |  |
| SERPINA1 | serpin peptidase inhibitor, clade A (alpha-1 antiproteinase, antitrypsin), member 1 |  |  | 2,81E-12 | -1,685 |  |  |
| SERPINB1 | serpin peptidase inhibitor, clade B (ovalbumin), member 1 |  |  | 2,45E-12 | -1,881 |  |  |
| SERPINH1 | serpin peptidase inhibitor, clade H (heat shock protein 47), member 1, (collagen binding protein 1) |  |  | 1,71E-10 | -1,769 |  |  |
| SET | SET nuclear oncogene |  |  |  |  | 3,00E-13 | 1,643 |
| SETD5 | SET domain containing 5 | 5,14E-07 | 2,631 |  |  |  |  |
| SETDB1 | SET domain, bifurcated 1 |  |  | 4,18E-09 | -2,495 |  |  |
| SF3B3 | splicing factor 3b, subunit 3, 130kDa |  |  | 8,11E-10 | -1,705 |  |  |
| SFN | stratifin |  |  | 9,78E-11 | -1,466 |  |  |
| SFN | stratifin |  |  | 3,40E-13 | -1,441 |  |  |
| SFRS1 | splicing factor, arginine/serine-rich 1 |  |  | 7,52E-11 | -1,438 | 7,52E-11 | -1,555 |
| SFRS12 | splicing factor, arginine/serine-rich 12 |  |  | 3,97E-10 | -1,507 |  |  |
| SFRS2 | splicing factor, arginine/serine-rich 2 |  |  | 1,69E-14 | -1,670 | 1,69E-14 | -1,609 |
| SFRS2B | splicing factor, arginine/serine-rich 2B |  |  | 5,22E-09 | -1,509 |  |  |
| SFRS2IP | splicing factor, arginine/serine-rich 2, interacting protein |  |  | 3,35E-12 | -1,707 |  |  |
| SFRS4 | splicing factor, arginine/serine-rich 4 |  |  | 7,03E-09 | -1,864 |  |  |
| SFRS5 | splicing factor, arginine/serine-rich 5 |  |  | 3,47E-08 | -1,782 |  |  |
| SFRS5 | splicing factor, arginine/serine-rich 5 | 1,69E-14 | -1,451 | 1,69E-14 | -2,293 | 1,69E-14 | -1,423 |
| SFRS6 | splicing factor, arginine/serine-rich 6 |  |  |  |  | 1,31E-13 | 1,990 |
| SFRS6 | splicing factor, arginine/serine-rich 6 |  |  | 3,66E-12 | -1,554 |  |  |
| SFT2D1 | SFT2 domain containing 1 |  |  | 1,21E-10 | -1,555 |  |  |
| SFXN4 | sideroflexin 4 |  |  | 4,27E-12 | -1,783 |  |  |
| SGCE | sarcoglycan, epsilon |  |  | 2,28E-09 | -1,638 | 2,28E-09 | -1,547 |
| SGEF | Src homology 3 domain-containing guanine nucleotide exchange factor |  |  |  |  | 6,57E-08 | -1,570 |
| SGOL2 | shugoshin-like 2 (S. pombe) |  |  | 1,34E-10 | -1,623 |  |  |
| SGPL1 | sphingosine-1-phosphate lyase 1 |  |  | 2,03E-12 | -1,573 |  |  |
| SH3BGRL3 | SH3 domain binding glutamic acid-rich protein like 3 |  |  | 2,88E-10 | -2,050 |  |  |
| SH3BP5 | SH3-domain binding protein 5 (BTK-associated) |  |  |  |  | 6,70E-08 | -1,515 |
| SH3GLB1 | SH3-domain GRB2-like endophilin B1 |  |  | 4,64E-13 | -1,700 |  |  |
| SH3GLB1 | SH3-domain GRB2-like endophilin B1 |  |  | 8,63E-12 | -1,839 |  |  |
| SH3KBP1 | SH3-domain kinase binding protein 1 |  |  | 2,58E-12 | -1,865 |  |  |
| SH3RF1 | SH3 domain containing ring finger 1 |  |  |  |  | 4,15E-11 | 2,642 |
| SH3RF2 | SH3 domain containing ring finger 2 |  |  | 1,17E-07 | -1,887 |  |  |
| SHC1 | SHC (Src homology 2 domain containing) transforming protein 1 |  |  | 1,34E-08 | -1,644 |  |  |
| SHISA5 | shisa homolog 5 (Xenopus laevis) |  |  | 6,10E-11 | -1,759 |  |  |
| SHMT1 | serine hydroxymethyltransferase 1 (soluble) |  |  | 4,64E-13 | -2,045 | 4,64E-13 | -1,635 |
| SHPRH | SNF2 histone linker PHD RING helicase |  |  | 7,70E-09 | -1,533 |  |  |
| SIGIRR | single immunoglobulin and toll-interleukin 1 receptor (TIR) domain |  |  | 7,45E-08 | -1,960 |  |  |
| SIPA1L3 (includes EG:23094) | signal-induced proliferation-associated 1 like 3 |  |  | 9,98E-13 | -2,440 |  |  |
| SIRT1 | sirtuin (silent mating type information regulation 2 homolog) 1 (S. cerevisiae) |  |  | 1,35E-12 | -1,865 | 1,35E-12 | -1,587 |
| SIRT7 | sirtuin (silent mating type information regulation 2 homolog) 7 (S. cerevisiae) |  |  | 5,18E-08 | -1,894 |  |  |
| SIVA1 | SIVA1, apoptosis-inducing factor |  |  | 3,00E-10 | -1,807 |  |  |
| SIVA1 | SIVA1, apoptosis-inducing factor |  |  | 6,28E-08 | -1,856 |  |  |
| SKAP2 | src kinase associated phosphoprotein 2 |  |  | 1,62E-12 | -1,867 |  |  |
| SKAP2 | src kinase associated phosphoprotein 2 |  |  | 3,22E-09 | -1,663 | 3,22E-09 | -1,709 |
| SKAP2 | src kinase associated phosphoprotein 2 |  |  | 5,88E-10 | -1,658 |  |  |
| SKP2 | S-phase kinase-associated protein 2 (p45) |  |  |  |  | 2,81E-10 | -1,839 |
| SLBP | stem-loop binding protein |  |  | 1,56E-12 | -1,674 | 1,56E-12 | -1,420 |
| SLC15A4 | solute carrier family 15, member 4 |  |  | 1,77E-11 | -2,036 |  |  |
| SLC16A1 | solute carrier family 16, member 1 (monocarboxylic acid transporter 1) | 3,31E-12 | -1,734 | 3,31E-12 | -2,204 | 3,31E-12 | -2,514 |
| SLC17A5 | solute carrier family 17 (anion/sugar transporter), member 5 |  |  | 8,35E-14 | -1,970 |  |  |
| SLC1A5 | solute carrier family 1 (neutral amino acid transporter), member 5 |  |  | 1,69E-14 | -2,970 | 1,69E-14 | -1,470 |
| SLC20A1 | solute carrier family 20 (phosphate transporter), member 1 |  |  |  |  | 9,76E-09 | 1,523 |
| SLC25A1 | solute carrier family 25 (mitochondrial carrier; citrate transporter), member 1 |  |  | 6,62E-11 | -2,018 |  |  |
| SLC25A11 | solute carrier family 25 (mitochondrial carrier; oxoglutarate carrier), member 11 |  |  | 3,25E-09 | -1,805 |  |  |
| SLC25A13 | solute carrier family 25, member 13 (citrin) |  |  | 2,25E-12 | -1,744 |  |  |
| SLC25A32 | solute carrier family 25, member 32 |  |  | 2,26E-08 | -1,581 |  |  |
| SLC25A39 | solute carrier family 25, member 39 |  |  | 4,60E-11 | -2,029 |  |  |
| SLC25A43 | solute carrier family 25, member 43 |  |  | 4,56E-09 | -1,643 |  |  |
| SLC25A6 | solute carrier family 25 (mitochondrial carrier; adenine nucleotide translocator), member 6 |  |  | 1,22E-10 | -1,653 |  |  |
| SLC2A4RG | SLC2A4 regulator |  |  | 5,95E-10 | -1,865 |  |  |
| SLC2A4RG | SLC2A4 regulator |  |  |  |  | 4,61E-08 | -1,926 |
| SLC30A5 | solute carrier family 30 (zinc transporter), member 5 |  |  | 8,38E-08 | -1,533 |  |  |
| SLC33A1 | solute carrier family 33 (acetyl-CoA transporter), member 1 |  |  | 7,19E-10 | -1,810 |  |  |
| SLC35A2 | solute carrier family 35 (UDP-galactose transporter), member A2 |  |  | 7,70E-11 | -1,835 |  |  |
| SLC35A4 | solute carrier family 35, member A4 |  |  | 1,43E-10 | -1,928 |  |  |
| SLC35B3 | solute carrier family 35, member B3 |  |  | 1,46E-08 | -1,988 |  |  |
| SLC37A4 | solute carrier family 37 (glucose-6-phosphate transporter), member 4 |  |  | 6,87E-08 | -1,764 |  |  |
| SLC37A4 | solute carrier family 37 (glucose-6-phosphate transporter), member 4 | 1,69E-14 | -1,717 | 1,69E-14 | -2,665 | 1,69E-14 | -2,106 |
| SLC38A1 | solute carrier family 38, member 1 |  |  | 6,56E-09 | -1,526 |  |  |
| SLC38A1 | solute carrier family 38, member 1 |  |  | 8,31E-13 | -1,649 |  |  |
| SLC38A10 | solute carrier family 38, member 10 |  |  | 9,90E-12 | -2,106 |  |  |
| SLC38A2 | solute carrier family 38, member 2 |  |  | 2,83E-09 | -1,558 |  |  |
| SLC39A1 | solute carrier family 39 (zinc transporter), member 1 |  |  | 7,17E-11 | -1,994 |  |  |
| SLC39A10 | solute carrier family 39 (zinc transporter), member 10 |  |  | 4,49E-14 | -1,787 |  |  |
| SLC39A14 | solute carrier family 39 (zinc transporter), member 14 |  |  | 1,69E-14 | -2,024 | 1,69E-14 | -3,589 |
| SLC39A6 | solute carrier family 39 (zinc transporter), member 6 |  |  | 1,37E-09 | -1,531 |  |  |
| SLC40A1 | solute carrier family 40 (iron-regulated transporter), member 1 |  |  | 3,69E-11 | -1,453 | 3,69E-11 | -1,552 |
| SLC44A1 | solute carrier family 44, member 1 |  |  | 1,31E-10 | -1,612 |  |  |
| SLC44A1 | solute carrier family 44, member 1 |  |  | 2,53E-06 | -1,565 |  |  |
| SLC44A2 | solute carrier family 44, member 2 |  |  | 9,68E-13 | -2,218 | 9,68E-13 | -1,554 |
| SLC44A3 | solute carrier family 44, member 3 | 7,94E-10 | -1,403 | 7,94E-10 | -1,828 |  |  |
| SLC46A1 | solute carrier family 46 (folate transporter), member 1 |  |  | 2,37E-09 | -1,759 |  |  |
| SLC4A1AP | solute carrier family 4 (anion exchanger), member 1, adaptor protein |  |  | 2,81E-10 | -2,006 |  |  |
| SLC4A2 | solute carrier family 4, anion exchanger, member 2 (erythrocyte membrane protein band 3-like 1) |  |  | 7,38E-10 | -1,972 |  |  |
| SLC9A3 | solute carrier family 9 (sodium/hydrogen exchanger), member 3 | 3,87E-13 | -1,523 | 3,87E-13 | -2,517 | 3,87E-13 | -1,848 |
| SLC9A6 | solute carrier family 9 (sodium/hydrogen exchanger), member 6 |  |  | 2,58E-09 | -1,783 |  |  |
| SLU7 | SLU7 splicing factor homolog (S. cerevisiae) | 3,30E-13 | -1,389 | 3,30E-13 | -2,029 | 3,30E-13 | -1,542 |
| SMA4 | glucuronidase, beta pseudogene | 4,34E-11 | -1,953 | 4,34E-11 | -2,041 | 4,34E-11 | -1,605 |
| SMAD4 | SMAD family member 4 |  |  | 1,06E-07 | -1,633 |  |  |
| SMARCA4 | SWI/SNF related, matrix associated, actin dependent regulator of chromatin, subfamily a, member 4 |  |  | 5,72E-11 | -1,830 |  |  |
| SMARCC1 | SWI/SNF related, matrix associated, actin dependent regulator of chromatin, subfamily c, member 1 |  |  | 1,69E-14 | 2,020 | 1,69E-14 | 3,185 |
| SMARCC1 | SWI/SNF related, matrix associated, actin dependent regulator of chromatin, subfamily c, member 1 |  |  | 5,03E-11 | -1,890 |  |  |
| SMARCD1 | SWI/SNF related, matrix associated, actin dependent regulator of chromatin, subfamily d, member 1 |  |  | 5,94E-09 | -1,910 |  |  |
| SMC2 | structural maintenance of chromosomes 2 |  |  | 1,08E-10 | -1,597 | 1,08E-10 | -1,484 |
| SMC3 | structural maintenance of chromosomes 3 |  |  |  |  | 1,69E-14 | 1,590 |
| SMC4 | structural maintenance of chromosomes 4 |  |  | 4,98E-09 | -1,471 | 4,98E-09 | -1,533 |
| SMCHD1 | structural maintenance of chromosomes flexible hinge domain containing 1 |  |  | 1,69E-14 | 2,448 | 1,69E-14 | 2,924 |
| SMCHD1 | structural maintenance of chromosomes flexible hinge domain containing 1 |  |  |  |  | 2,44E-07 | 2,212 |
| SMCR8 | Smith-Magenis syndrome chromosome region, candidate 8 |  |  | 9,31E-11 | -1,957 |  |  |
| SMCR8 | Smith-Magenis syndrome chromosome region, candidate 8 |  |  | 1,25E-09 | -1,644 |  |  |
| SMEK2 | SMEK homolog 2, suppressor of mek1 (Dictyostelium) | 1,69E-14 | -1,588 | 1,69E-14 | -2,535 | 1,69E-14 | -2,025 |
| SMN1 | survival of motor neuron 1, telomeric |  |  | 1,69E-14 | -1,957 | 1,69E-14 | -1,612 |
| SMS | spermine synthase |  |  | 5,50E-11 | -1,766 |  |  |
| SMUG1 | single-strand-selective monofunctional uracil-DNA glycosylase 1 |  |  | 5,64E-08 | -1,772 |  |  |
| SNAP23 | synaptosomal-associated protein, 23kDa | 1,69E-14 | -1,773 | 1,69E-14 | -1,504 | 1,69E-14 | -2,013 |
| SNHG12 | small nucleolar RNA host gene 12 (non-protein coding) |  |  | 7,49E-13 | -2,022 |  |  |
| SNRNP25 | small nuclear ribonucleoprotein 25kDa (U11/U12) |  |  | 3,05E-09 | -1,672 |  |  |
| SNRPA | small nuclear ribonucleoprotein polypeptide A |  |  | 6,07E-11 | -1,765 |  |  |
| SNRPA1 | small nuclear ribonucleoprotein polypeptide A' |  |  | 1,21E-12 | -1,505 |  |  |
| SNRPA1 | small nuclear ribonucleoprotein polypeptide A' |  |  | 5,23E-10 | -1,827 |  |  |
| SNRPB2 | small nuclear ribonucleoprotein polypeptide B'' |  |  | 5,93E-08 | -1,520 |  |  |
| SNRPG | small nuclear ribonucleoprotein polypeptide G |  |  |  |  | 8,42E-10 | -1,372 |
| SNRPN | small nuclear ribonucleoprotein polypeptide N |  |  | 1,69E-14 | -1,897 | 1,69E-14 | -1,822 |
| SNRPN | small nuclear ribonucleoprotein polypeptide N |  |  | 1,49E-10 | -1,937 |  |  |
| SNTB2 | syntrophin, beta 2 (dystrophin-associated protein A1, 59kDa, basic component 2) |  |  | 7,93E-09 | -1,875 |  |  |
| SNTB2 | syntrophin, beta 2 (dystrophin-associated protein A1, 59kDa, basic component 2) |  |  | 1,92E-08 | -1,618 |  |  |
| SNUPN | snurportin 1 |  |  | 5,23E-08 | -1,643 |  |  |
| SNW1 | SNW domain containing 1 |  |  | 1,43E-08 | -1,538 |  |  |
| SNW1 | SNW domain containing 1 |  |  | 7,47E-12 | -1,779 |  |  |
| SNX1 | sorting nexin 1 |  |  | 8,41E-11 | -1,806 |  |  |
| SNX1 | sorting nexin 1 |  |  | 1,03E-10 | -1,867 |  |  |
| SNX12 | sorting nexin 12 |  |  | 8,48E-11 | -1,849 |  |  |
| SNX13 | sorting nexin 13 |  |  | 3,50E-09 | -1,866 |  |  |
| SNX2 | sorting nexin 2 |  |  | 2,41E-09 | -1,485 | 2,41E-09 | -1,413 |
| SNX24 | sorting nexin 24 |  |  | 7,06E-10 | -1,905 |  |  |
| SNX3 | sorting nexin 3 |  |  | 2,32E-12 | -1,618 | 2,32E-12 | -1,621 |
| SNX9 (includes EG:51429) | sorting nexin 9 |  |  | 1,80E-12 | -1,801 |  |  |
| SOCS2 | suppressor of cytokine signaling 2 |  |  | 1,56E-10 | -1,777 |  |  |
| SOCS2 | suppressor of cytokine signaling 2 |  |  | 5,45E-09 | -1,532 |  |  |
| SOLH | small optic lobes homolog (Drosophila) |  |  | 1,07E-08 | -2,084 |  |  |
| SON | SON DNA binding protein | 4,55E-13 | -1,813 | 4,55E-13 | -1,689 |  |  |
| SORBS2 | sorbin and SH3 domain containing 2 |  |  | 2,35E-09 | -2,117 |  |  |
| SOX2 | SRY (sex determining region Y)-box 2 |  |  | 1,69E-14 | -2,107 | 1,69E-14 | -2,041 |
| SOX9 | SRY (sex determining region Y)-box 9 |  |  |  |  | 1,95E-13 | 1,669 |
| SP2 | Sp2 transcription factor |  |  | 1,54E-10 | -1,614 | 1,54E-10 | -1,781 |
| SPAG1 | sperm associated antigen 1 |  |  | 5,17E-10 | -1,812 |  |  |
| SPAG9 | sperm associated antigen 9 |  |  |  |  | 5,07E-08 | -1,618 |
| SPAG9 | sperm associated antigen 9 |  |  |  |  | 1,22E-08 | 1,700 |
| SPATA20 | spermatogenesis associated 20 |  |  | 1,69E-14 | -2,152 |  |  |
| SPC24 | SPC24, NDC80 kinetochore complex component, homolog (S. cerevisiae) |  |  | 1,69E-14 | -2,613 |  |  |
| SPCS1 (includes EG:28972) | signal peptidase complex subunit 1 homolog (S. cerevisiae) |  |  | 2,63E-13 | -1,619 | 2,63E-13 | -1,455 |
| SPEN | spen homolog, transcriptional regulator (Drosophila) |  |  |  |  | 1,69E-14 | 2,167 |
| SPG11 (includes EG:80208) | spastic paraplegia 11 (autosomal recessive) |  |  | 2,48E-11 | -1,710 |  |  |
| SPG7 | spastic paraplegia 7 (pure and complicated autosomal recessive) |  |  | 2,81E-08 | -1,662 |  |  |
| SPG7 | spastic paraplegia 7 (pure and complicated autosomal recessive) |  |  |  |  | 3,21E-13 | 1,577 |
| SPINT1 | serine peptidase inhibitor, Kunitz type 1 |  |  | 7,25E-12 | -1,960 |  |  |
| SPON1 | spondin 1, extracellular matrix protein |  |  | 1,08E-09 | -1,874 |  |  |
| SPPL2A | signal peptide peptidase-like 2A |  |  | 8,06E-10 | -1,619 |  |  |
| SPR | sepiapterin reductase (7,8-dihydrobiopterin:NADP+ oxidoreductase) |  |  | 3,56E-08 | -1,763 |  |  |
| SPSB2 | splA/ryanodine receptor domain and SOCS box containing 2 | 4,29E-13 | -2,330 | 4,29E-13 | -2,683 | 4,29E-13 | -1,821 |
| SPTBN1 | spectrin, beta, non-erythrocytic 1 |  |  |  |  | 4,21E-13 | 2,398 |
| SPTBN1 | spectrin, beta, non-erythrocytic 1 |  |  |  |  | 1,07E-11 | 1,840 |
| SPTLC2 | serine palmitoyltransferase, long chain base subunit 2 |  |  | 8,65E-09 | -1,863 |  |  |
| SR140 | U2-associated SR140 protein |  |  | 1,95E-09 | -1,495 |  |  |
| SR140 | U2-associated SR140 protein |  |  | 5,00E-10 | -1,555 |  |  |
| SRA1 | steroid receptor RNA activator 1 |  |  | 1,11E-09 | -1,697 |  |  |
| SRD5A1 | steroid-5-alpha-reductase, alpha polypeptide 1 (3-oxo-5 alpha-steroid delta 4-dehydrogenase alpha 1) |  |  | 7,15E-08 | -1,981 |  |  |
| SRI | sorcin |  |  | 3,94E-13 | -1,780 |  |  |
| SRP72 | signal recognition particle 72kDa |  |  | 1,98E-11 | -1,661 | 1,98E-11 | -1,468 |
| SRP72 | signal recognition particle 72kDa |  |  | 5,52E-08 | -1,515 |  |  |
| SRP9 | signal recognition particle 9kDa |  |  | 1,92E-10 | -1,501 |  |  |
| SRPK2 | SFRS protein kinase 2 |  |  | 5,13E-09 | -1,660 |  |  |
| SRRM1 | serine/arginine repetitive matrix 1 |  |  | 7,48E-10 | -1,535 |  |  |
| SRRM2 | serine/arginine repetitive matrix 2 |  |  | 1,51E-11 | -1,691 |  |  |
| SRRM2 | serine/arginine repetitive matrix 2 |  |  |  |  | 1,69E-14 | 1,648 |
| SS18 | synovial sarcoma translocation, chromosome 18 | 7,35E-11 | -1,709 | 7,35E-11 | -1,622 |  |  |
| SS18L2 | synovial sarcoma translocation gene on chromosome 18-like 2 |  |  | 1,07E-08 | -1,598 |  |  |
| SSB | Sjogren syndrome antigen B (autoantigen La) |  |  | 5,94E-09 | -1,516 |  |  |
| SSBP1 | single-stranded DNA binding protein 1 |  |  | 6,62E-11 | -1,572 |  |  |
| SSH3 | slingshot homolog 3 (Drosophila) |  |  | 1,69E-14 | -2,547 | 1,69E-14 | -2,308 |
| SSRP1 | structure specific recognition protein 1 |  |  | 1,48E-09 | -1,726 |  |  |
| ST13 | suppression of tumorigenicity 13 (colon carcinoma) (Hsp70 interacting protein) |  |  | 1,56E-08 | -1,485 |  |  |
| ST13 | suppression of tumorigenicity 13 (colon carcinoma) (Hsp70 interacting protein) |  |  | 4,49E-14 | -1,908 |  |  |
| ST14 | suppression of tumorigenicity 14 (colon carcinoma) |  |  | 5,80E-11 | -1,905 |  |  |
| STAG2 | stromal antigen 2 |  |  | 2,49E-11 | -1,646 |  |  |
| STAM | signal transducing adaptor molecule (SH3 domain and ITAM motif) 1 |  |  | 1,58E-09 | -1,595 |  |  |
| STARD10 | StAR-related lipid transfer (START) domain containing 10 |  |  | 2,44E-13 | -2,104 |  |  |
| STARD10 | StAR-related lipid transfer (START) domain containing 10 |  |  | 1,95E-13 | -2,032 |  |  |
| STARD3NL | STARD3 N-terminal like |  |  | 3,34E-12 | -1,775 |  |  |
| STAU1 | staufen, RNA binding protein, homolog 1 (Drosophila) |  |  | 1,23E-10 | -1,572 |  |  |
| STEAP1 | six transmembrane epithelial antigen of the prostate 1 |  |  | 1,15E-11 | -1,547 |  |  |
| STIP1 | stress-induced-phosphoprotein 1 | 1,69E-14 | -3,106 | 1,69E-14 | -3,591 | 1,69E-14 | -2,493 |
| STIP1 | stress-induced-phosphoprotein 1 | 1,69E-14 | -2,539 | 1,69E-14 | -3,699 | 1,69E-14 | -1,627 |
| STK19 | serine/threonine kinase 19 |  |  | 2,65E-08 | -1,817 |  |  |
| STK35 | serine/threonine kinase 35 |  |  | 1,80E-09 | -1,835 |  |  |
| STMN3 | stathmin-like 3 | 9,83E-12 | -1,581 | 9,83E-12 | -1,932 |  |  |
| STOM | stomatin |  |  |  |  | 4,51E-08 | -1,897 |
| STOM | stomatin |  |  | 3,87E-09 | -1,948 |  |  |
| STOML2 | stomatin (EPB72)-like 2 |  |  | 3,42E-09 | -1,639 |  |  |
| STRA13 | stimulated by retinoic acid 13 homolog (mouse) |  |  | 1,69E-14 | -2,100 |  |  |
| STRN3 | striatin, calmodulin binding protein 3 |  |  |  |  | 1,29E-10 | 2,942 |
| STT3B | STT3, subunit of the oligosaccharyltransferase complex, homolog B (S. cerevisiae) |  |  | 5,05E-13 | -1,666 |  |  |
| STX16 | syntaxin 16 |  |  | 5,96E-09 | -1,637 |  |  |
| STX3 | syntaxin 3 |  |  | 5,82E-09 | -1,512 | 5,82E-09 | -1,637 |
| STX6 | syntaxin 6 |  |  | 3,68E-10 | -1,732 |  |  |
| STYX | serine/threonine/tyrosine interacting protein |  |  | 1,69E-14 | -2,140 |  |  |
| STYX | serine/threonine/tyrosine interacting protein |  |  |  |  | 1,69E-14 | 1,669 |
| SUCLA2 | succinate-CoA ligase, ADP-forming, beta subunit |  |  | 1,69E-14 | -1,742 | 1,69E-14 | -1,493 |
| SUCLG2 | succinate-CoA ligase, GDP-forming, beta subunit |  |  | 8,91E-10 | -1,523 |  |  |
| SUCLG2 | succinate-CoA ligase, GDP-forming, beta subunit |  |  | 1,55E-12 | -1,647 |  |  |
| SUCLG2 | succinate-CoA ligase, GDP-forming, beta subunit |  |  | 1,45E-09 | -1,585 |  |  |
| SUDS3 | suppressor of defective silencing 3 homolog (S. cerevisiae) |  |  | 2,54E-11 | -1,630 |  |  |
| SULT1A1 | sulfotransferase family, cytosolic, 1A, phenol-preferring, member 1 |  |  | 1,67E-11 | -2,139 | 1,67E-11 | -1,702 |
| SULT1A3 | sulfotransferase family, cytosolic, 1A, phenol-preferring, member 3 |  |  | 1,27E-08 | -1,747 |  |  |
| SUMO1 | SMT3 suppressor of mif two 3 homolog 1 (S. cerevisiae) |  |  | 1,52E-08 | -1,529 |  |  |
| SUMO1 | SMT3 suppressor of mif two 3 homolog 1 (S. cerevisiae) |  |  | 6,64E-13 | -1,978 | 6,64E-13 | -1,788 |
| SUMO3 | SMT3 suppressor of mif two 3 homolog 3 (S. cerevisiae) |  |  | 5,21E-12 | -1,618 |  |  |
| SUOX | sulfite oxidase |  |  | 6,82E-10 | -2,099 |  |  |
| SUPT6H | suppressor of Ty 6 homolog (S. cerevisiae) |  |  | 3,27E-10 | -2,154 |  |  |
| SURF6 | surfeit 6 |  |  | 8,17E-10 | -2,098 |  |  |
| SVIP (includes EG:258010) | small VCP/p97-interacting protein |  |  | 3,97E-08 | -2,036 |  |  |
| SYNCRIP | synaptotagmin binding, cytoplasmic RNA interacting protein |  |  | 3,64E-08 | -1,494 |  |  |
| SYNCRIP | synaptotagmin binding, cytoplasmic RNA interacting protein |  |  | 3,48E-09 | -1,554 |  |  |
| SYNGR2 | synaptogyrin 2 |  |  | 2,63E-08 | -1,721 |  |  |
| SYNJ2 | synaptojanin 2 |  |  | 6,58E-10 | -2,035 |  |  |
| SYNJ2BP | synaptojanin 2 binding protein |  |  |  |  | 2,46E-10 | 1,652 |
| SYPL1 | synaptophysin-like 1 |  |  | 6,87E-13 | -1,527 | 6,87E-13 | -1,687 |
| TACC1 | transforming, acidic coiled-coil containing protein 1 |  |  | 1,69E-14 | -2,202 | 1,69E-14 | -1,530 |
| TACC3 | transforming, acidic coiled-coil containing protein 3 |  |  | 6,88E-09 | -2,038 |  |  |
| TAF10 | TAF10 RNA polymerase II, TATA box binding protein (TBP)-associated factor, 30kDa |  |  | 1,02E-09 | -1,696 |  |  |
| TAF11 | TAF11 RNA polymerase II, TATA box binding protein (TBP)-associated factor, 28kDa |  |  | 8,26E-09 | -1,670 |  |  |
| TAF4B | TAF4b RNA polymerase II, TATA box binding protein (TBP)-associated factor, 105kDa |  |  | 3,80E-08 | -1,969 |  |  |
| TAF6 | TAF6 RNA polymerase II, TATA box binding protein (TBP)-associated factor, 80kDa |  |  | 1,41E-09 | -1,816 |  |  |
| TAF7 | TAF7 RNA polymerase II, TATA box binding protein (TBP)-associated factor, 55kDa |  |  | 5,00E-09 | -1,474 |  |  |
| TAF9B | TAF9B RNA polymerase II, TATA box binding protein (TBP)-associated factor, 31kDa |  |  |  |  | 7,41E-13 | -2,010 |
| TAGLN | transgelin |  |  | 5,81E-09 | -1,708 |  |  |
| TAGLN | transgelin |  |  | 8,35E-14 | -1,725 |  |  |
| TAGLN2 | transgelin 2 |  |  | 1,78E-11 | -1,825 |  |  |
| TANK | TRAF family member-associated NFKB activator |  |  | 3,34E-12 | -1,768 |  |  |
| TATDN1 (includes EG:83940) | TatD DNase domain containing 1 |  |  | 5,12E-08 | -1,538 |  |  |
| TAX1BP1 | Tax1 (human T-cell leukemia virus type I) binding protein 1 |  |  | 5,43E-08 | -1,494 |  |  |
| TAX1BP3 | Tax1 (human T-cell leukemia virus type I) binding protein 3 |  |  | 1,59E-10 | -1,648 |  |  |
| TBC1D20 | TBC1 domain family, member 20 |  |  | 1,17E-07 | -1,995 |  |  |
| TBC1D22A | TBC1 domain family, member 22A |  |  | 2,79E-08 | -1,818 |  |  |
| TBCB | tubulin folding cofactor B |  |  | 1,74E-10 | -1,834 |  |  |
| TBK1 | TANK-binding kinase 1 |  |  | 5,90E-10 | -1,640 |  |  |
| TBL1XR1 | transducin (beta)-like 1 X-linked receptor 1 |  |  |  |  | 1,69E-14 | 2,529 |
| TBPL1 | TBP-like 1 |  |  | 6,00E-09 | -1,631 |  |  |
| TBRG1 (includes EG:84897) | transforming growth factor beta regulator 1 |  |  | 3,66E-08 | -1,887 |  |  |
| TCAG7.1228 | ubinuclein 2 |  |  |  |  | 8,91E-10 | 2,902 |
| TCEAL1 | transcription elongation factor A (SII)-like 1 |  |  | 5,41E-10 | -1,764 | 5,41E-10 | -1,603 |
| TCERG1 | transcription elongation regulator 1 |  |  | 6,01E-10 | -1,486 |  |  |
| TCF25 | transcription factor 25 (basic helix-loop-helix) |  |  |  |  | 4,88E-10 | 1,466 |
| TCF4 | transcription factor 4 |  |  | 1,69E-14 | -2,419 | 1,69E-14 | -1,781 |
| TCF7L2 (includes EG:6934) | transcription factor 7-like 2 (T-cell specific, HMG-box) |  |  | 9,15E-13 | -1,848 |  |  |
| TCF7L2 (includes EG:6934) | transcription factor 7-like 2 (T-cell specific, HMG-box) |  |  | 6,41E-08 | -1,584 |  |  |
| TCF7L2 (includes EG:6934) | transcription factor 7-like 2 (T-cell specific, HMG-box) |  |  | 3,11E-09 | -1,659 |  |  |
| TCFL5 | transcription factor-like 5 (basic helix-loop-helix) |  |  | 1,69E-14 | -2,221 | 1,69E-14 | -1,734 |
| TCTEX1D2 | Tctex1 domain containing 2 |  |  | 4,31E-10 | -1,653 | 4,31E-10 | -1,578 |
| TCTN3 | tectonic family member 3 |  |  | 3,95E-08 | -1,661 |  |  |
| TDRD7 | tudor domain containing 7 |  |  | 4,94E-08 | -1,594 |  |  |
| TDRKH | tudor and KH domain containing |  |  | 1,41E-10 | -2,060 |  |  |
| TEAD3 | TEA domain family member 3 |  |  | 5,27E-09 | -2,099 |  |  |
| TELO2 | TEL2, telomere maintenance 2, homolog (S. cerevisiae) |  |  | 2,71E-08 | -2,699 |  |  |
| TERF2 | telomeric repeat binding factor 2 |  |  | 8,95E-09 | -1,672 |  |  |
| TES | testis derived transcript (3 LIM domains) |  |  |  |  | 3,80E-09 | 1,382 |
| TEX261 | testis expressed 261 |  |  | 2,84E-12 | -1,974 | 2,84E-12 | -1,350 |
| TFDP1 | transcription factor Dp-1 |  |  | 1,80E-12 | -1,666 |  |  |
| TFDP1 | transcription factor Dp-1 |  |  | 1,69E-14 | -3,141 | 1,69E-14 | -2,280 |
| TFDP2 | transcription factor Dp-2 (E2F dimerization partner 2) |  |  | 1,18E-10 | -2,157 |  |  |
| TFE3 | transcription factor binding to IGHM enhancer 3 | 2,16E-13 | -1,588 | 2,16E-13 | -2,398 | 2,16E-13 | -1,640 |
| TFEC | transcription factor EC | 1,69E-14 | -1,362 | 1,69E-14 | -2,211 | 1,69E-14 | -2,436 |
| TFEC | transcription factor EC |  |  | 2,35E-12 | -2,439 | 2,35E-12 | -2,692 |
| TFRC | transferrin receptor (p90, CD71) | 1,69E-14 | -1,689 | 1,69E-14 | -2,189 | 1,69E-14 | -2,845 |
| TGDS | TDP-glucose 4,6-dehydratase |  |  | 3,14E-14 | -1,758 | 3,14E-14 | -1,484 |
| TGFBR1 | transforming growth factor, beta receptor 1 |  |  | 2,63E-10 | -1,674 |  |  |
| TGFBR2 | transforming growth factor, beta receptor II (70/80kDa) |  |  | 4,33E-09 | -1,603 |  |  |
| TGM2 | transglutaminase 2 (C polypeptide, protein-glutamine-gamma-glutamyltransferase) |  |  | 4,49E-14 | -2,778 | 4,49E-14 | -1,809 |
| TGS1 | trimethylguanosine synthase homolog (S. cerevisiae) | 7,16E-10 | -1,433 | 7,16E-10 | -1,837 |  |  |
| TGS1 | trimethylguanosine synthase homolog (S. cerevisiae) |  |  |  |  | 1,74E-07 | 1,417 |
| TH1L | TH1-like (Drosophila) |  |  | 1,09E-09 | -1,633 |  |  |
| TH1L | TH1-like (Drosophila) |  |  | 6,76E-09 | -1,589 |  |  |
| TH1L | TH1-like (Drosophila) |  |  | 1,05E-09 | -1,662 |  |  |
| TH1L | TH1-like (Drosophila) |  |  | 2,15E-09 | -1,608 |  |  |
| THAP5 | THAP domain containing 5 |  |  | 2,13E-10 | -1,803 |  |  |
| THAP7 | THAP domain containing 7 |  |  | 1,84E-08 | -2,339 |  |  |
| THBS1 | thrombospondin 1 |  |  |  |  | 7,37E-09 | 1,805 |
| THEM2 | thioesterase superfamily member 2 |  |  | 7,09E-11 | -1,538 | 7,09E-11 | -1,565 |
| THOC2 | THO complex 2 |  |  |  |  | 3,91E-10 | 1,837 |
| THOC4 | THO complex 4 |  |  |  |  | 3,33E-08 | 1,301 |
| THOC4 | THO complex 4 |  |  |  |  | 1,11E-06 | 1,407 |
| THOC5 | THO complex 5 |  |  | 3,69E-10 | -2,096 |  |  |
| THOC7 | THO complex 7 homolog (Drosophila) |  |  | 1,69E-14 | -1,925 |  |  |
| THRA | thyroid hormone receptor, alpha (erythroblastic leukemia viral (v-erb-a) oncogene homolog, avian) |  |  |  |  | 4,03E-13 | 3,605 |
| THRAP3 | thyroid hormone receptor associated protein 3 |  |  | 1,69E-14 | 2,360 | 1,69E-14 | 2,921 |
| THUMPD1 | THUMP domain containing 1 |  |  | 3,14E-14 | -1,989 |  |  |
| THUMPD3 | THUMP domain containing 3 |  |  | 1,78E-09 | -1,857 |  |  |
| THYN1 | thymocyte nuclear protein 1 |  |  | 2,56E-08 | -1,553 |  |  |
| TIMM10 | translocase of inner mitochondrial membrane 10 homolog (yeast) |  |  | 3,30E-13 | -1,875 |  |  |
| TIMM10 | translocase of inner mitochondrial membrane 10 homolog (yeast) |  |  | 2,09E-10 | -1,836 |  |  |
| TIMM23 | translocase of inner mitochondrial membrane 23 homolog (yeast) |  |  | 1,28E-08 | -1,612 |  |  |
| TIMM23 | translocase of inner mitochondrial membrane 23 homolog (yeast) |  |  | 3,16E-09 | -1,523 |  |  |
| TIMM23B | translocase of inner mitochondrial membrane 23 homolog B (yeast) |  |  | 1,00E-08 | -1,581 |  |  |
| TIMM44 | translocase of inner mitochondrial membrane 44 homolog (yeast) |  |  | 1,35E-08 | -2,437 |  |  |
| TIMP2 | TIMP metallopeptidase inhibitor 2 |  |  | 4,19E-08 | -1,800 |  |  |
| TIMP2 | TIMP metallopeptidase inhibitor 2 |  |  | 3,38E-08 | -1,724 |  |  |
| TINP1 | TGF beta-inducible nuclear protein 1 |  |  | 1,40E-08 | -1,457 |  |  |
| TIPARP | TCDD-inducible poly(ADP-ribose) polymerase |  |  |  |  | 6,66E-09 | 1,743 |
| TK1 | thymidine kinase 1, soluble | 1,69E-14 | -1,323 | 1,69E-14 | -2,152 |  |  |
| TK1 | thymidine kinase 1, soluble |  |  | 5,86E-08 | -1,640 |  |  |
| TKT | transketolase |  |  | 5,65E-11 | -1,827 |  |  |
| TKT | transketolase |  |  | 6,28E-11 | -1,656 |  |  |
| TM2D2 | TM2 domain containing 2 |  |  | 1,25E-12 | -1,839 | 1,25E-12 | -1,363 |
| TM2D3 | TM2 domain containing 3 |  |  | 1,81E-08 | -1,627 |  |  |
| TM4SF1 | transmembrane 4 L six family member 1 |  |  |  |  | 4,79E-13 | 1,369 |
| TM4SF1 | transmembrane 4 L six family member 1 |  |  |  |  | 2,26E-12 | 1,374 |
| TM7SF2 | transmembrane 7 superfamily member 2 |  |  | 2,16E-08 | -2,130 |  |  |
| TM7SF3 | transmembrane 7 superfamily member 3 |  |  | 4,53E-08 | -1,621 |  |  |
| TM9SF1 | transmembrane 9 superfamily member 1 |  |  | 8,47E-08 | -1,943 |  |  |
| TM9SF1 | transmembrane 9 superfamily member 1 |  |  | 3,66E-09 | -1,794 |  |  |
| TM9SF4 | transmembrane 9 superfamily protein member 4 |  |  | 2,15E-11 | -2,213 |  |  |
| TMBIM1 | transmembrane BAX inhibitor motif containing 1 | 2,23E-11 | -1,363 | 2,23E-11 | -1,906 |  |  |
| TMED10 | transmembrane emp24-like trafficking protein 10 (yeast) |  |  | 9,11E-09 | -1,506 |  |  |
| TMED10 | transmembrane emp24-like trafficking protein 10 (yeast) |  |  | 4,36E-11 | -1,738 |  |  |
| TMED3 | transmembrane emp24 protein transport domain containing 3 |  |  | 2,26E-13 | -2,047 |  |  |
| TMED4 | transmembrane emp24 protein transport domain containing 4 |  |  | 7,09E-11 | -1,712 |  |  |
| TMEM103 | chromosome 3 open reading frame 75 |  |  | 7,85E-09 | -2,149 |  |  |
| TMEM106B | transmembrane protein 106B | 6,81E-11 | 1,705 |  |  | 6,81E-11 | 1,807 |
| TMEM120A | transmembrane protein 120A |  |  | 9,68E-10 | -1,901 |  |  |
| TMEM126B | transmembrane protein 126B |  |  | 6,73E-10 | -1,474 |  |  |
| TMEM134 | transmembrane protein 134 |  |  | 9,51E-14 | -2,240 | 9,51E-14 | -1,583 |
| TMEM135 | transmembrane protein 135 |  |  | 1,90E-08 | -1,555 |  |  |
| TMEM138 | transmembrane protein 138 |  |  | 4,05E-10 | -1,819 |  |  |
| TMEM139 | transmembrane protein 139 |  |  | 2,79E-12 | -2,066 | 2,79E-12 | -1,989 |
| TMEM14A | transmembrane protein 14A |  |  | 4,49E-14 | -1,676 | 4,49E-14 | -1,478 |
| TMEM14B | transmembrane protein 14B |  |  | 1,69E-14 | -1,641 | 1,69E-14 | -1,531 |
| TMEM14B | transmembrane protein 14B |  |  | 2,01E-09 | -1,640 |  |  |
| TMEM14C | transmembrane protein 14C |  |  | 3,24E-09 | -1,490 |  |  |
| TMEM14C | transmembrane protein 14C |  |  | 1,58E-11 | -1,575 |  |  |
| TMEM165 | transmembrane protein 165 |  |  | 3,14E-08 | -1,647 |  |  |
| TMEM167A | transmembrane protein 167A |  |  | 2,89E-11 | -1,580 | 2,89E-11 | -1,373 |
| TMEM188 | transmembrane protein 188 |  |  | 2,70E-10 | -1,495 | 2,70E-10 | -1,630 |
| TMEM19 | transmembrane protein 19 |  |  | 8,06E-11 | -1,706 | 8,06E-11 | -1,549 |
| TMEM19 | transmembrane protein 19 |  |  | 6,90E-10 | -1,777 |  |  |
| TMEM199 | transmembrane protein 199 |  |  | 8,45E-11 | -1,854 |  |  |
| TMEM203 | transmembrane protein 203 |  |  | 1,29E-10 | -1,678 | 1,29E-10 | -1,485 |
| TMEM205 | transmembrane protein 205 |  |  | 2,28E-08 | -1,848 |  |  |
| TMEM209 | transmembrane protein 209 |  |  | 6,53E-12 | -1,904 |  |  |
| TMEM30A | transmembrane protein 30A |  |  | 4,90E-10 | -1,464 | 4,90E-10 | -1,532 |
| TMEM30A | transmembrane protein 30A |  |  | 1,69E-14 | -1,554 | 1,69E-14 | -1,990 |
| TMEM33 | transmembrane protein 33 | 2,47E-11 | -1,481 | 2,47E-11 | -1,832 | 2,47E-11 | -1,349 |
| TMEM37 | transmembrane protein 37 |  |  | 3,77E-09 | -2,012 |  |  |
| TMEM37 | transmembrane protein 37 |  |  | 2,38E-08 | -2,045 |  |  |
| TMEM39A | transmembrane protein 39A |  |  | 3,58E-08 | -1,710 |  |  |
| TMEM43 | transmembrane protein 43 |  |  | 1,00E-10 | -1,894 |  |  |
| TMEM45B | transmembrane protein 45B |  |  | 3,14E-14 | -1,801 | 3,14E-14 | -1,613 |
| TMEM45B | transmembrane protein 45B |  |  | 6,28E-09 | -1,587 |  |  |
| TMEM54 | transmembrane protein 54 |  |  | 2,19E-08 | -1,910 |  |  |
| TMEM55B | transmembrane protein 55B | 3,14E-14 | -1,556 | 3,14E-14 | -2,422 | 3,14E-14 | -1,828 |
| TMEM64 | transmembrane protein 64 |  |  | 1,69E-14 | -1,843 | 1,69E-14 | -1,615 |
| TMEM68 | transmembrane protein 68 |  |  | 4,16E-11 | -2,028 |  |  |
| TMEM77 | transmembrane protein 77 |  |  | 2,97E-10 | -1,740 | 2,97E-10 | -1,486 |
| TMEM8 | transmembrane protein 8 (five membrane-spanning domains) | 1,69E-14 | -1,311 | 1,69E-14 | -2,002 |  |  |
| TMEM85 | transmembrane protein 85 |  |  | 2,20E-11 | -1,685 | 2,20E-11 | -1,515 |
| TMEM85 | transmembrane protein 85 |  |  |  |  | 6,53E-09 | -1,518 |
| TMEM87A | transmembrane protein 87A |  |  | 2,15E-08 | -1,764 |  |  |
| TMEM9 | transmembrane protein 9 | 1,69E-14 | -1,590 | 1,69E-14 | -2,193 | 1,69E-14 | -1,884 |
| TMEM9 | transmembrane protein 9 | 1,69E-14 | -1,528 | 1,69E-14 | -2,061 | 1,69E-14 | -1,323 |
| TMEM97 | transmembrane protein 97 | 1,69E-14 | -1,352 | 1,69E-14 | -2,372 | 1,69E-14 | -1,522 |
| TMEM97 | transmembrane protein 97 |  |  | 8,38E-08 | -1,604 |  |  |
| TMEM98 | transmembrane protein 98 |  |  | 4,03E-12 | -1,770 | 4,03E-12 | -1,461 |
| TMOD3 | tropomodulin 3 (ubiquitous) |  |  | 8,34E-09 | -1,542 |  |  |
| TMPO | thymopoietin |  |  | 1,01E-09 | -1,560 |  |  |
| TMSL8 | thymosin-like 8 |  |  | 4,32E-10 | -2,027 |  |  |
| TMTC3 | transmembrane and tetratricopeptide repeat containing 3 |  |  | 2,18E-07 | -1,393 |  |  |
| TMTC3 | transmembrane and tetratricopeptide repeat containing 3 |  |  | 2,22E-08 | -1,526 |  |  |
| TNFAIP8 | tumor necrosis factor, alpha-induced protein 8 |  |  |  |  | 4,27E-12 | 1,535 |
| TNFAIP8L1 | tumor necrosis factor, alpha-induced protein 8-like 1 |  |  | 5,46E-12 | -2,361 |  |  |
| TNFRSF10D | tumor necrosis factor receptor superfamily, member 10d, decoy with truncated death domain |  |  | 1,70E-08 | -1,748 |  |  |
| TNFRSF11B | tumor necrosis factor receptor superfamily, member 11b |  |  |  |  | 4,18E-10 | 1,551 |
| TNIP1 | TNFAIP3 interacting protein 1 |  |  | 2,02E-09 | -1,687 |  |  |
| TNPO1 | transportin 1 |  |  | 2,24E-08 | -1,557 |  |  |
| TNPO2 | transportin 2 (importin 3, karyopherin beta 2b) |  |  | 9,33E-08 | -1,638 |  |  |
| TNRC6A | trinucleotide repeat containing 6A |  |  | 3,78E-09 | -1,634 |  |  |
| TOB1 | transducer of ERBB2, 1 | 1,69E-14 | -1,469 | 1,69E-14 | -1,820 | 1,69E-14 | -2,496 |
| TOLLIP | toll interacting protein |  |  | 1,22E-07 | -2,076 |  |  |
| TOM1L1 | target of myb1 (chicken)-like 1 |  |  | 1,57E-09 | -1,612 |  |  |
| TOMM20 | translocase of outer mitochondrial membrane 20 homolog (yeast) |  |  | 2,77E-11 | -1,653 |  |  |
| TOMM20 | translocase of outer mitochondrial membrane 20 homolog (yeast) |  |  | 2,65E-11 | -1,624 | 2,65E-11 | -1,370 |
| TOMM22 | translocase of outer mitochondrial membrane 22 homolog (yeast) |  |  | 3,54E-09 | -1,548 |  |  |
| TOMM34 | translocase of outer mitochondrial membrane 34 |  |  | 1,40E-11 | -1,854 |  |  |
| TOMM5 (includes EG:401505) | translocase of outer mitochondrial membrane 5 homolog (yeast) |  |  | 7,30E-10 | -1,629 |  |  |
| TOMM7 | translocase of outer mitochondrial membrane 7 homolog (yeast) |  |  | 3,44E-09 | -1,544 |  |  |
| TOMM70A | translocase of outer mitochondrial membrane 70 homolog A (S. cerevisiae) |  |  | 4,79E-13 | -1,925 |  |  |
| TOP1 | topoisomerase (DNA) I |  |  |  |  | 1,69E-14 | 1,782 |
| TOP2A | topoisomerase (DNA) II alpha 170kDa |  |  | 1,69E-14 | -1,966 | 1,69E-14 | -1,743 |
| TOR1B | torsin family 1, member B (torsin B) |  |  | 8,44E-09 | -1,737 |  |  |
| TP53BP2 | tumor protein p53 binding protein, 2 |  |  | 4,51E-11 | -2,056 |  |  |
| TPD52 | tumor protein D52 |  |  | 2,18E-11 | -1,646 | 2,18E-11 | -1,708 |
| TPD52 | tumor protein D52 |  |  | 1,69E-14 | -1,718 | 1,69E-14 | -1,717 |
| TPD52L1 | tumor protein D52-like 1 |  |  | 1,74E-11 | -1,705 | 1,74E-11 | -1,454 |
| TPD52L1 | tumor protein D52-like 1 |  |  | 2,16E-13 | -1,740 | 2,16E-13 | -1,932 |
| TPD52L2 | tumor protein D52-like 2 |  |  | 7,11E-13 | -1,906 |  |  |
| TPI1 | triosephosphate isomerase 1 |  |  | 3,90E-09 | -1,570 |  |  |
| TPK1 | thiamin pyrophosphokinase 1 |  |  | 3,49E-13 | -1,817 | 3,49E-13 | -1,468 |
| TPK1 | thiamin pyrophosphokinase 1 |  |  | 6,87E-13 | -1,998 | 6,87E-13 | -1,583 |
| TPM1 | tropomyosin 1 (alpha) |  |  | 1,13E-09 | -1,477 |  |  |
| TPP1 | tripeptidyl peptidase I |  |  | 1,69E-14 | -2,385 | 1,69E-14 | -1,833 |
| TPP1 | tripeptidyl peptidase I |  |  | 2,63E-09 | -1,674 | 2,63E-09 | -1,359 |
| TPR | translocated promoter region (to activated MET oncogene) |  |  | 1,90E-10 | 1,618 | 1,90E-10 | 1,764 |
| TPR | translocated promoter region (to activated MET oncogene) |  |  |  |  | 3,67E-11 | 1,571 |
| TPRKB | TP53RK binding protein |  |  | 2,71E-13 | -1,767 |  |  |
| TPX2 | TPX2, microtubule-associated, homolog (Xenopus laevis) |  |  | 1,36E-10 | -1,613 | 1,36E-10 | -1,458 |
| TRADD | TNFRSF1A-associated via death domain |  |  | 1,51E-10 | -1,874 |  |  |
| TRAM1 | translocation associated membrane protein 1 |  |  | 2,61E-09 | -1,434 | 2,61E-09 | -1,509 |
| TRAM2 (includes EG:9697) | translocation associated membrane protein 2 |  |  | 6,30E-08 | -1,762 |  |  |
| TRAM2 (includes EG:9697) | translocation associated membrane protein 2 |  |  | 2,01E-07 | -1,610 |  |  |
| TRAP1 | TNF receptor-associated protein 1 |  |  | 2,33E-08 | -1,640 |  |  |
| TRAPPC2L | trafficking protein particle complex 2-like |  |  | 5,02E-08 | -1,673 |  |  |
| TRIB1 | tribbles homolog 1 (Drosophila) |  |  |  |  | 1,69E-14 | 1,493 |
| TRIM13 | tripartite motif-containing 13 |  |  | 4,19E-09 | -1,678 |  |  |
| TRIM25 | tripartite motif-containing 25 |  |  | 3,08E-08 | -1,708 |  |  |
| TRIM28 | tripartite motif-containing 28 |  |  | 7,53E-09 | -1,790 |  |  |
| TRIM41 | tripartite motif-containing 41 |  |  | 3,96E-10 | -2,251 |  |  |
| TRIM8 | tripartite motif-containing 8 |  |  | 5,76E-10 | -2,010 |  |  |
| TRMT5 | TRM5 tRNA methyltransferase 5 homolog (S. cerevisiae) |  |  | 2,38E-10 | -1,610 |  |  |
| TROAP | trophinin associated protein (tastin) |  |  | 2,68E-10 | -2,061 |  |  |
| TRPC4AP | transient receptor potential cation channel, subfamily C, member 4 associated protein |  |  | 1,17E-09 | -1,901 |  |  |
| TRRAP | transformation/transcription domain-associated protein |  |  | 1,72E-09 | -1,858 |  |  |
| TRUB1 | TruB pseudouridine (psi) synthase homolog 1 (E. coli) |  |  |  |  | 3,54E-11 | -4,420 |
| TSEN15 | tRNA splicing endonuclease 15 homolog (S. cerevisiae) |  |  | 4,24E-10 | -1,927 | 4,24E-10 | -1,895 |
| TSEN15 | tRNA splicing endonuclease 15 homolog (S. cerevisiae) |  |  | 7,09E-11 | -1,896 | 7,09E-11 | -1,744 |
| TSG101 | tumor susceptibility gene 101 |  |  | 2,20E-10 | -1,697 |  |  |
| TSN | translin |  |  |  |  | 8,80E-08 | -1,568 |
| TSN | translin |  |  | 3,36E-08 | -1,792 |  |  |
| TSNAX | translin-associated factor X |  |  | 2,91E-13 | -1,826 |  |  |
| TSPAN15 | tetraspanin 15 |  |  | 1,19E-13 | -2,228 |  |  |
| TSPAN6 | tetraspanin 6 |  |  | 3,52E-10 | -1,561 | 3,52E-10 | -1,394 |
| TSPAN6 | tetraspanin 6 |  |  | 7,98E-11 | -1,567 |  |  |
| TSPAN8 | tetraspanin 8 |  |  | 4,65E-08 | -1,522 |  |  |
| TSR1 | TSR1, 20S rRNA accumulation, homolog (S. cerevisiae) | 1,69E-14 | -2,296 | 1,69E-14 | -2,412 |  |  |
| TSR2 | TSR2, 20S rRNA accumulation, homolog (S. cerevisiae) |  |  | 2,14E-09 | -2,014 |  |  |
| TSTA3 | tissue specific transplantation antigen P35B |  |  | 1,55E-10 | -1,786 |  |  |
| TTC3 | tetratricopeptide repeat domain 3 |  |  | 4,08E-10 | -1,600 |  |  |
| TTC3 | tetratricopeptide repeat domain 3 |  |  |  |  | 1,15E-10 | 1,785 |
| TTC3 | tetratricopeptide repeat domain 3 |  |  | 5,22E-09 | -1,578 |  |  |
| TTC32 | tetratricopeptide repeat domain 32 |  |  |  |  | 8,82E-09 | -1,772 |
| TTC8 | tetratricopeptide repeat domain 8 |  |  | 1,78E-09 | -1,823 |  |  |
| TTF2 | transcription termination factor, RNA polymerase II |  |  | 5,31E-10 | -1,547 |  |  |
| TTK | TTK protein kinase |  |  | 4,42E-09 | -1,572 |  |  |
| TTL | tubulin tyrosine ligase |  |  | 1,08E-11 | -1,808 |  |  |
| TTR | transthyretin |  |  | 1,69E-14 | -2,090 | 1,69E-14 | -1,502 |
| TUBA1A | tubulin, alpha 1a |  |  |  |  | 5,64E-13 | 1,465 |
| TUBA4A | tubulin, alpha 4a |  |  | 1,48E-10 | -1,735 |  |  |
| TUBD1 | tubulin, delta 1 |  |  | 1,54E-10 | -1,875 |  |  |
| TUBG1 | tubulin, gamma 1 |  |  | 4,54E-11 | -1,760 |  |  |
| TUG1 (includes EG:55000) | taurine upregulated 1 (non-protein coding) |  |  | 9,66E-10 | -1,480 |  |  |
| TUSC2 | tumor suppressor candidate 2 |  |  | 1,15E-09 | -2,033 |  |  |
| TWF1 | twinfilin, actin-binding protein, homolog 1 (Drosophila) |  |  |  |  | 1,69E-14 | -2,028 |
| TWF1 | twinfilin, actin-binding protein, homolog 1 (Drosophila) |  |  | 1,09E-08 | 2,175 |  |  |
| TWSG1 | twisted gastrulation homolog 1 (Drosophila) | 7,19E-13 | -1,325 | 7,19E-13 | -1,778 | 7,19E-13 | -1,416 |
| TXN | thioredoxin |  |  | 8,68E-10 | 1,875 |  |  |
| TXN2 | thioredoxin 2 |  |  | 1,06E-09 | -1,989 |  |  |
| TXNDC14 | thioredoxin domain containing 14 |  |  | 3,15E-08 | -1,546 |  |  |
| TXNDC9 | thioredoxin domain containing 9 |  |  | 4,43E-11 | -1,691 |  |  |
| TXNIP | thioredoxin interacting protein |  |  | 1,04E-09 | -1,585 |  |  |
| TXNL1 | thioredoxin-like 1 |  |  | 1,09E-10 | -1,644 |  |  |
| TXNL4A (includes EG:10907) | thioredoxin-like 4A |  |  | 5,17E-09 | -1,600 |  |  |
| TXNL4B | thioredoxin-like 4B |  |  | 1,22E-08 | -1,922 |  |  |
| TYMS | thymidylate synthetase |  |  | 3,10E-12 | -1,999 |  |  |
| TYRO3 | TYRO3 protein tyrosine kinase |  |  | 1,57E-10 | -2,269 |  |  |
| U2AF2 (includes EG:11338) | U2 small nuclear RNA auxiliary factor 2 |  |  | 8,05E-12 | -1,910 | 8,05E-12 | -1,529 |
| UBA2 | ubiquitin-like modifier activating enzyme 2 |  |  | 7,95E-08 | -1,774 |  |  |
| UBA5 | ubiquitin-like modifier activating enzyme 5 |  |  | 1,69E-14 | -1,892 |  |  |
| UBAP2L | ubiquitin associated protein 2-like |  |  | 5,15E-10 | -1,764 |  |  |
| UBAP2L | ubiquitin associated protein 2-like |  |  | 2,20E-08 | -1,809 |  |  |
| UBC | ubiquitin C |  |  | 1,14E-10 | -1,638 |  |  |
| UBE2A | ubiquitin-conjugating enzyme E2A (RAD6 homolog) |  |  | 8,33E-09 | -1,530 |  |  |
| UBE2B | ubiquitin-conjugating enzyme E2B (RAD6 homolog) |  |  | 4,49E-14 | -1,604 | 4,49E-14 | -1,306 |
| UBE2D3 | ubiquitin-conjugating enzyme E2D 3 (UBC4/5 homolog, yeast) |  |  | 1,69E-14 | -1,673 |  |  |
| UBE2E2 | ubiquitin-conjugating enzyme E2E 2 (UBC4/5 homolog, yeast) |  |  | 2,49E-09 | -1,963 |  |  |
| UBE2E3 | ubiquitin-conjugating enzyme E2E 3 (UBC4/5 homolog, yeast) |  |  | 6,85E-11 | -1,689 |  |  |
| UBE2F | ubiquitin-conjugating enzyme E2F (putative) |  |  | 1,14E-08 | -1,933 |  |  |
| UBE2G1 | ubiquitin-conjugating enzyme E2G 1 (UBC7 homolog, yeast) |  |  | 3,11E-12 | -1,566 | 3,11E-12 | -1,508 |
| UBE2G2 | ubiquitin-conjugating enzyme E2G 2 (UBC7 homolog, yeast) |  |  | 5,33E-09 | -1,817 |  |  |
| UBE2I | ubiquitin-conjugating enzyme E2I (UBC9 homolog, yeast) |  |  | 1,07E-09 | -1,637 |  |  |
| UBE2J1 | ubiquitin-conjugating enzyme E2, J1 (UBC6 homolog, yeast) | 2,48E-09 | -1,382 | 2,48E-09 | -1,751 |  |  |
| UBE2K | ubiquitin-conjugating enzyme E2K (UBC1 homolog, yeast) | 1,69E-14 | -1,609 | 1,69E-14 | -2,465 |  |  |
| UBE2L3 | ubiquitin-conjugating enzyme E2L 3 |  |  | 7,92E-12 | -1,596 | 7,92E-12 | -1,361 |
| UBE2L3 | ubiquitin-conjugating enzyme E2L 3 |  |  | 6,67E-11 | -1,782 |  |  |
| UBE2M | ubiquitin-conjugating enzyme E2M (UBC12 homolog, yeast) |  |  | 8,09E-08 | -1,753 |  |  |
| UBE2Q1 | ubiquitin-conjugating enzyme E2Q family member 1 |  |  | 6,25E-09 | -1,764 |  |  |
| UBE2Q2 | ubiquitin-conjugating enzyme E2Q family member 2 |  |  | 1,69E-14 | -1,835 | 1,69E-14 | -1,459 |
| UBE2R2 (includes EG:54926) | ubiquitin-conjugating enzyme E2R 2 |  |  | 1,54E-11 | -1,816 |  |  |
| UBE2T | ubiquitin-conjugating enzyme E2T (putative) |  |  | 1,33E-09 | -1,665 |  |  |
| UBE2V1 | ubiquitin-conjugating enzyme E2 variant 1 |  |  | 1,69E-14 | -2,246 |  |  |
| UBE2V2 | ubiquitin-conjugating enzyme E2 variant 2 |  |  | 4,72E-13 | -1,767 |  |  |
| UBE3A | ubiquitin protein ligase E3A |  |  | 4,02E-10 | -1,876 |  |  |
| UBE3C | ubiquitin protein ligase E3C |  |  | 2,58E-09 | -2,407 |  |  |
| UBE3C | ubiquitin protein ligase E3C |  |  | 1,16E-10 | -1,653 |  |  |
| UBL3 | ubiquitin-like 3 |  |  | 2,48E-08 | -1,621 |  |  |
| UBLCP1 | ubiquitin-like domain containing CTD phosphatase 1 |  |  | 3,98E-12 | -1,748 |  |  |
| UBN1 | ubinuclein 1 |  |  | 1,69E-14 | 1,674 | 1,69E-14 | 2,033 |
| UBP1 | upstream binding protein 1 (LBP-1a) |  |  | 2,97E-09 | -1,591 |  |  |
| UBQLN1 | ubiquilin 1 |  |  | 1,80E-09 | -1,564 |  |  |
| UBXN2A | UBX domain protein 2A |  |  | 6,07E-11 | -1,810 |  |  |
| UBXN4 | UBX domain protein 4 |  |  |  |  | 1,69E-14 | 1,829 |
| UBXN4 | UBX domain protein 4 |  |  | 1,40E-11 | -1,755 | 1,40E-11 | -1,468 |
| UBXN6 | UBX domain protein 6 |  |  | 4,00E-10 | -2,159 |  |  |
| UBXN7 | UBX domain protein 7 |  |  |  |  | 6,82E-12 | 1,487 |
| UCHL3 | ubiquitin carboxyl-terminal esterase L3 (ubiquitin thiolesterase) |  |  | 2,10E-07 | -1,479 |  |  |
| UCK2 | uridine-cytidine kinase 2 |  |  | 9,08E-08 | -1,667 |  |  |
| UCP2 | uncoupling protein 2 (mitochondrial, proton carrier) |  |  | 4,17E-08 | -2,151 |  |  |
| UCP2 | uncoupling protein 2 (mitochondrial, proton carrier) | 1,69E-14 | -1,356 | 1,69E-14 | -2,133 | 1,69E-14 | -1,448 |
| UEVLD | UEV and lactate/malate dehyrogenase domains |  |  |  |  | 6,11E-09 | -1,937 |
| UFD1L | ubiquitin fusion degradation 1 like (yeast) |  |  | 7,16E-11 | -1,622 |  |  |
| UGCG | UDP-glucose ceramide glucosyltransferase |  |  |  |  | 1,69E-14 | 2,592 |
| UGCG | UDP-glucose ceramide glucosyltransferase |  |  | 4,49E-14 | -1,431 |  |  |
| UGCGL2 | UDP-glucose ceramide glucosyltransferase-like 2 |  |  | 3,81E-11 | -1,770 |  |  |
| UGP2 | UDP-glucose pyrophosphorylase 2 |  |  | 1,95E-10 | -1,562 |  |  |
| UHMK1 | U2AF homology motif (UHM) kinase 1 |  |  | 5,76E-13 | 1,637 | 5,76E-13 | 2,252 |
| UHMK1 | U2AF homology motif (UHM) kinase 1 |  |  |  |  | 9,89E-09 | 1,961 |
| UHRF1 | ubiquitin-like with PHD and ring finger domains 1 |  |  | 1,11E-08 | -1,513 |  |  |
| UNC119B | unc-119 homolog B (C. elegans) |  |  | 2,16E-08 | -1,617 |  |  |
| UNC50 | unc-50 homolog (C. elegans) |  |  | 1,69E-14 | -1,748 | 1,69E-14 | -1,592 |
| UNC84A | unc-84 homolog A (C. elegans) |  |  | 8,34E-10 | -1,505 |  |  |
| UNK | unkempt homolog (Drosophila) |  |  | 2,22E-10 | -1,717 | 2,22E-10 | -1,608 |
| UNQ1887 | signal peptide peptidase 3 |  |  | 2,04E-10 | -1,738 |  |  |
| UPF3A | UPF3 regulator of nonsense transcripts homolog A (yeast) |  |  | 1,70E-12 | -1,779 |  |  |
| UPF3B | UPF3 regulator of nonsense transcripts homolog B (yeast) |  |  | 3,32E-11 | -1,794 | 3,32E-11 | -1,441 |
| UQCR | ubiquinol-cytochrome c reductase, 6.4kDa subunit |  |  | 4,49E-14 | -1,733 |  |  |
| UQCRB | ubiquinol-cytochrome c reductase binding protein | 2,84E-10 | -1,298 | 2,84E-10 | -1,619 |  |  |
| UQCRB | ubiquinol-cytochrome c reductase binding protein |  |  | 1,80E-11 | -1,667 |  |  |
| UQCRC2 | ubiquinol-cytochrome c reductase core protein II |  |  | 6,12E-11 | -1,545 | 6,12E-11 | -1,430 |
| UQCRH | ubiquinol-cytochrome c reductase hinge protein |  |  | 8,73E-11 | -1,650 |  |  |
| USP10 | ubiquitin specific peptidase 10 |  |  |  |  | 1,69E-14 | 1,583 |
| USP10 | ubiquitin specific peptidase 10 |  |  | 2,33E-09 | -1,844 |  |  |
| USP14 | ubiquitin specific peptidase 14 (tRNA-guanine transglycosylase) |  |  | 3,87E-13 | -1,860 |  |  |
| USP16 | ubiquitin specific peptidase 16 |  |  | 1,63E-10 | -1,885 |  |  |
| USP38 | ubiquitin specific peptidase 38 |  |  | 5,14E-12 | -1,720 |  |  |
| USP39 | ubiquitin specific peptidase 39 | 4,79E-12 | -1,383 | 4,79E-12 | -1,957 |  |  |
| USP46 | ubiquitin specific peptidase 46 |  |  | 4,93E-09 | -1,800 |  |  |
| USP47 | ubiquitin specific peptidase 47 |  |  |  |  | 2,48E-10 | 1,699 |
| USP9X | ubiquitin specific peptidase 9, X-linked |  |  | 2,04E-11 | -1,795 |  |  |
| UTP18 | UTP18, small subunit (SSU) processome component, homolog (yeast) |  |  | 8,78E-09 | -1,536 |  |  |
| UTP20 | UTP20, small subunit (SSU) processome component, homolog (yeast) |  |  |  |  | 3,78E-13 | 2,180 |
| UVRAG | UV radiation resistance associated gene |  |  | 8,11E-08 | -1,771 |  |  |
| UXT | ubiquitously-expressed transcript |  |  | 1,25E-10 | -1,741 |  |  |
| VAMP3 | vesicle-associated membrane protein 3 (cellubrevin) |  |  | 1,69E-14 | -1,948 | 1,69E-14 | -2,193 |
| VAMP3 | vesicle-associated membrane protein 3 (cellubrevin) |  |  | 1,63E-13 | -1,689 | 1,63E-13 | -1,604 |
| VAMP7 | vesicle-associated membrane protein 7 |  |  | 7,98E-09 | -1,471 |  |  |
| VAMP8 | vesicle-associated membrane protein 8 (endobrevin) |  |  | 2,37E-09 | -1,640 |  |  |
| VAPB | VAMP (vesicle-associated membrane protein)-associated protein B and C |  |  |  |  | 4,86E-12 | 3,950 |
| VAPB | VAMP (vesicle-associated membrane protein)-associated protein B and C |  |  | 1,56E-11 | -1,678 |  |  |
| VARS | valyl-tRNA synthetase |  |  | 5,13E-13 | -2,232 |  |  |
| VCL | vinculin |  |  | 1,58E-08 | -1,591 |  |  |
| VCP | valosin-containing protein |  |  | 1,24E-07 | -1,725 |  |  |
| VDAC3 | voltage-dependent anion channel 3 |  |  | 1,53E-13 | -1,697 | 1,53E-13 | -1,496 |
| VEGFA | vascular endothelial growth factor A |  |  | 1,79E-08 | -1,965 |  |  |
| VEZF1 | vascular endothelial zinc finger 1 |  |  | 1,26E-09 | -1,688 |  |  |
| VEZF1 | vascular endothelial zinc finger 1 |  |  | 9,63E-09 | -1,665 |  |  |
| VIL1 | villin 1 | 4,98E-11 | -2,079 | 4,98E-11 | -2,179 |  |  |
| VIL1 | villin 1 | 4,79E-13 | -2,289 | 4,79E-13 | -2,162 |  |  |
| VKORC1 | vitamin K epoxide reductase complex, subunit 1 |  |  | 3,65E-08 | -1,715 |  |  |
| VPS24 | vacuolar protein sorting 24 homolog (S. cerevisiae) |  |  | 1,69E-14 | -1,770 | 1,69E-14 | -1,429 |
| VPS25 | vacuolar protein sorting 25 homolog (S. cerevisiae) |  |  | 5,53E-10 | -1,690 |  |  |
| VPS29 | vacuolar protein sorting 29 homolog (S. cerevisiae) |  |  | 1,29E-08 | -1,479 |  |  |
| VPS35 | vacuolar protein sorting 35 homolog (S. cerevisiae) |  |  | 4,22E-09 | -1,555 |  |  |
| VPS54 | vacuolar protein sorting 54 homolog (S. cerevisiae) |  |  | 4,21E-08 | -1,535 |  |  |
| VTA1 | Vps20-associated 1 homolog (S. cerevisiae) |  |  | 7,19E-13 | -1,540 | 7,19E-13 | -1,592 |
| VTA1 | Vps20-associated 1 homolog (S. cerevisiae) |  |  | 1,40E-09 | -1,661 |  |  |
| WAC | WW domain containing adaptor with coiled-coil |  |  | 7,90E-12 | -1,661 |  |  |
| WAPAL | wings apart-like homolog (Drosophila) |  |  | 5,22E-10 | -1,727 |  |  |
| WASF2 | WAS protein family, member 2 |  |  |  |  | 1,53E-13 | 4,118 |
| WASL (includes EG:8976) | Wiskott-Aldrich syndrome-like |  |  |  |  | 2,84E-11 | 1,643 |
| WBP11 | WW domain binding protein 11 |  |  | 9,98E-13 | -1,800 | 9,98E-13 | -1,574 |
| WBP11 | WW domain binding protein 11 |  |  | 6,49E-10 | -1,581 |  |  |
| WBSCR22 | Williams Beuren syndrome chromosome region 22 |  |  | 3,14E-14 | -1,745 |  |  |
| WDR1 | WD repeat domain 1 |  |  | 9,77E-13 | -1,772 |  |  |
| WDR1 | WD repeat domain 1 | 1,69E-14 | -1,431 | 1,69E-14 | -2,141 | 1,69E-14 | -1,832 |
| WDR26 | WD repeat domain 26 |  |  | 1,12E-09 | -1,660 |  |  |
| WDR33 | WD repeat domain 33 |  |  | 2,90E-10 | -3,869 |  |  |
| WDR34 | WD repeat domain 34 |  |  | 1,69E-14 | -2,229 |  |  |
| WDR5 | WD repeat domain 5 |  |  | 6,89E-12 | -2,176 |  |  |
| WDR5 | WD repeat domain 5 |  |  | 6,60E-10 | -2,052 |  |  |
| WDR55 | WD repeat domain 55 |  |  | 9,11E-09 | -1,686 |  |  |
| WDR61 | WD repeat domain 61 |  |  | 2,44E-08 | -1,619 |  |  |
| WDR61 | WD repeat domain 61 |  |  | 2,32E-12 | -1,676 |  |  |
| WDR72 | WD repeat domain 72 |  |  | 1,63E-08 | -1,580 |  |  |
| WDR74 | WD repeat domain 74 |  |  | 2,17E-12 | -2,063 |  |  |
| WDR79 | WD repeat domain 79 |  |  | 2,53E-10 | -1,793 |  |  |
| WDR82 | WD repeat domain 82 |  |  | 4,32E-10 | -1,610 |  |  |
| WDR92 | WD repeat domain 92 |  |  |  |  | 1,63E-10 | 1,724 |
| WHSC2 | Wolf-Hirschhorn syndrome candidate 2 |  |  | 2,28E-10 | -1,739 |  |  |
| WIPF2 | WAS/WASL interacting protein family, member 2 |  |  | 9,32E-09 | -1,852 |  |  |
| WIPF2 | WAS/WASL interacting protein family, member 2 |  |  | 2,82E-09 | -1,714 |  |  |
| WIPI2 | WD repeat domain, phosphoinositide interacting 2 |  |  | 5,56E-12 | -1,907 |  |  |
| WRB | tryptophan rich basic protein |  |  | 1,19E-10 | -1,560 | 1,19E-10 | -1,486 |
| WSB1 | WD repeat and SOCS box-containing 1 |  |  | 1,77E-12 | -1,769 |  |  |
| WSB2 | WD repeat and SOCS box-containing 2 |  |  | 7,34E-12 | -1,813 |  |  |
| WTAP | Wilms tumor 1 associated protein |  |  | 3,90E-08 | -1,419 |  |  |
| WTAP | Wilms tumor 1 associated protein |  |  | 2,30E-10 | -1,422 |  |  |
| WWC1 | WW and C2 domain containing 1 |  |  |  |  | 4,07E-09 | 1,643 |
| WWP1 | WW domain containing E3 ubiquitin protein ligase 1 |  |  | 2,27E-10 | -1,954 |  |  |
| WWTR1 | WW domain containing transcription regulator 1 |  |  | 1,40E-08 | -1,524 |  |  |
| XPNPEP1 | X-prolyl aminopeptidase (aminopeptidase P) 1, soluble |  |  | 1,35E-09 | -1,770 |  |  |
| XPO5 | exportin 5 |  |  | 4,80E-09 | -2,209 |  |  |
| XPO6 | exportin 6 |  |  | 2,54E-11 | -1,950 |  |  |
| XPO7 | exportin 7 |  |  | 3,04E-12 | -1,948 |  |  |
| XRCC5 | X-ray repair complementing defective repair in Chinese hamster cells 5 (double-strand-break rejoining) |  |  | 8,23E-08 | -1,453 |  |  |
| XRCC5 | X-ray repair complementing defective repair in Chinese hamster cells 5 (double-strand-break rejoining) |  |  | 3,10E-13 | -1,671 |  |  |
| XRCC6BP1 | XRCC6 binding protein 1 |  |  | 8,50E-08 | -1,702 |  |  |
| YAF2 | YY1 associated factor 2 |  |  | 4,79E-12 | -2,198 | 4,79E-12 | -2,005 |
| YAP1 | Yes-associated protein 1, 65kDa |  |  | 1,69E-14 | -1,737 | 1,69E-14 | -1,400 |
| YARS | tyrosyl-tRNA synthetase |  |  | 5,71E-13 | -1,973 |  |  |
| YDD19 | YDD19 protein |  |  |  |  | 3,45E-09 | -1,961 |
| YIPF3 | Yip1 domain family, member 3 | 1,69E-14 | -1,931 | 1,69E-14 | -2,782 | 1,69E-14 | -1,623 |
| YIPF5 | Yip1 domain family, member 5 | 2,81E-13 | -1,805 | 2,81E-13 | -1,866 |  |  |
| YLPM1 | YLP motif containing 1 |  |  |  |  | 2,17E-09 | 2,035 |
| YPEL5 | yippee-like 5 (Drosophila) |  |  | 5,49E-09 | -1,724 |  |  |
| YTHDF3 | YTH domain family, member 3 |  |  | 5,73E-10 | -1,700 | 5,73E-10 | -1,869 |
| YWHAQ (includes EG:10971) | tyrosine 3-monooxygenase/tryptophan 5-monooxygenase activation protein, theta polypeptide |  |  | 1,17E-08 | -1,458 |  |  |
| YWHAZ | tyrosine 3-monooxygenase/tryptophan 5-monooxygenase activation protein, zeta polypeptide |  |  | 7,47E-11 | -1,626 |  |  |
| ZAK | sterile alpha motif and leucine zipper containing kinase AZK |  |  | 1,50E-08 | -1,592 |  |  |
| ZBED1 | zinc finger, BED-type containing 1 |  |  | 1,83E-08 | -1,935 |  |  |
| ZBTB2 | zinc finger and BTB domain containing 2 |  |  | 1,47E-07 | -1,570 |  |  |
| ZBTB44 | zinc finger and BTB domain containing 44 |  |  | 3,28E-08 | -1,549 |  |  |
| ZBTB44 | zinc finger and BTB domain containing 44 |  |  | 1,89E-08 | -1,573 |  |  |
| ZC3H11A | zinc finger CCCH-type containing 11A |  |  |  |  | 6,11E-13 | 2,084 |
| ZC3H15 | zinc finger CCCH-type containing 15 |  |  | 3,91E-10 | -1,557 |  |  |
| ZC3H8 | zinc finger CCCH-type containing 8 |  |  | 8,97E-11 | -1,763 |  |  |
| ZC3HAV1L | zinc finger CCCH-type, antiviral 1-like |  |  |  |  | 1,44E-09 | -1,471 |
| ZCCHC17 | zinc finger, CCHC domain containing 17 |  |  | 3,30E-13 | -2,253 |  |  |
| ZCCHC7 | zinc finger, CCHC domain containing 7 |  |  | 2,18E-09 | -1,724 |  |  |
| ZCCHC8 | zinc finger, CCHC domain containing 8 |  |  | 2,33E-12 | -1,727 |  |  |
| ZCCHC9 | zinc finger, CCHC domain containing 9 |  |  | 2,12E-11 | -1,710 |  |  |
| ZCRB1 | zinc finger CCHC-type and RNA binding motif 1 |  |  | 5,17E-08 | -1,552 |  |  |
| ZCRB1 | zinc finger CCHC-type and RNA binding motif 1 | 2,16E-13 | -1,373 | 2,16E-13 | -1,899 |  |  |
| ZDHHC12 | zinc finger, DHHC-type containing 12 |  |  | 9,06E-09 | -2,816 |  |  |
| ZDHHC16 | zinc finger, DHHC-type containing 16 |  |  | 6,64E-09 | -1,629 |  |  |
| ZDHHC4 | zinc finger, DHHC-type containing 4 |  |  | 2,72E-11 | -2,053 |  |  |
| ZDHHC5 | zinc finger, DHHC-type containing 5 |  |  | 7,63E-10 | -1,846 |  |  |
| ZFAND2A | zinc finger, AN1-type domain 2A |  |  | 7,29E-09 | -1,819 |  |  |
| ZFP36L2 | zinc finger protein 36, C3H type-like 2 |  |  | 6,57E-08 | -2,081 |  |  |
| ZFP91 | zinc finger protein 91 homolog (mouse) |  |  |  |  | 5,76E-13 | 1,837 |
| ZFR | zinc finger RNA binding protein |  |  |  |  | 9,66E-10 | 1,926 |
| ZFR | zinc finger RNA binding protein |  |  | 1,69E-14 | 1,809 | 1,69E-14 | 2,193 |
| ZFYVE21 | zinc finger, FYVE domain containing 21 | 6,98E-12 | -1,418 | 6,98E-12 | -2,021 | 6,98E-12 | -1,799 |
| ZFYVE21 | zinc finger, FYVE domain containing 21 |  |  | 7,82E-11 | -2,000 |  |  |
| ZGPAT | zinc finger, CCCH-type with G patch domain |  |  | 2,13E-08 | -1,726 |  |  |
| ZMAT2 | zinc finger, matrin type 2 |  |  | 9,56E-12 | -1,729 |  |  |
| ZMIZ1 | zinc finger, MIZ-type containing 1 |  |  | 9,15E-12 | -2,160 | 9,15E-12 | -1,714 |
| ZMYND8 | zinc finger, MYND-type containing 8 |  |  | 2,00E-12 | -1,788 |  |  |
| ZNF12 | zinc finger protein 12 |  |  | 3,65E-08 | -1,695 |  |  |
| ZNF146 | zinc finger protein 146 |  |  | 1,65E-08 | -1,870 |  |  |
| ZNF207 | zinc finger protein 207 |  |  | 7,55E-10 | -1,527 | 7,55E-10 | -1,492 |
| ZNF281 | zinc finger protein 281 |  |  |  |  | 3,94E-13 | 1,504 |
| ZNF294 | ring finger protein 160 |  |  |  |  | 7,74E-10 | 1,918 |
| ZNF302 | zinc finger protein 302 |  |  | 2,72E-11 | -1,579 | 2,72E-11 | -1,413 |
| ZNF320 | zinc finger protein 320 |  |  | 9,51E-14 | -2,534 |  |  |
| ZNF367 | zinc finger protein 367 | 8,51E-10 | -1,347 | 8,51E-10 | -1,730 | 8,51E-10 | -1,493 |
| ZNF45 | zinc finger protein 45 |  |  | 1,35E-08 | -1,803 |  |  |
| ZNF462 | zinc finger protein 462 | 3,66E-08 | -1,615 |  |  |  |  |
| ZNF511 | zinc finger protein 511 |  |  | 3,86E-10 | -1,709 |  |  |
| ZNF512B | zinc finger protein 512B | 1,69E-14 | -1,288 | 1,69E-14 | -1,998 |  |  |
| ZNF593 | zinc finger protein 593 |  |  | 3,09E-10 | -1,830 |  |  |
| ZNF638 | zinc finger protein 638 |  |  | 1,01E-10 | -1,763 |  |  |
| ZNF641 | zinc finger protein 641 |  |  | 1,74E-07 | -1,636 |  |  |
| ZNF664 | zinc finger protein 664 |  |  | 1,69E-14 | -1,856 | 1,69E-14 | -1,460 |
| ZNF706 | zinc finger protein 706 |  |  | 1,07E-13 | -1,840 |  |  |
| ZNF711 | zinc finger protein 711 |  |  | 3,02E-09 | -1,664 |  |  |
| ZNF720 | zinc finger protein 720 |  |  | 3,31E-09 | -1,854 |  |  |
| ZNHIT3 | zinc finger, HIT type 3 |  |  | 3,74E-10 | -1,675 |  |  |
| ZNRD1 | zinc ribbon domain containing 1 |  |  | 2,99E-12 | -1,915 | 2,99E-12 | -1,566 |
| ZNRD1 | zinc ribbon domain containing 1 |  |  | 4,56E-09 | -1,728 |  |  |
| ZNRF1 | zinc and ring finger 1 |  |  | 2,39E-08 | -1,904 |  |  |
| ZNRF1 | zinc and ring finger 1 |  |  | 1,38E-08 | -2,025 |  |  |
| ZNRF3 | zinc and ring finger 3 |  |  | 4,10E-09 | -1,809 |  |  |
| ZRANB1 | zinc finger, RAN-binding domain containing 1 |  |  | 6,29E-09 | -1,823 |  |  |
| ZW10 | ZW10, kinetochore associated, homolog (Drosophila) |  |  | 5,23E-09 | -1,672 |  |  |
| ZXDC | ZXD family zinc finger C |  |  |  |  | 2,36E-08 | 1,866 |
