# Supplementary material for: AS601245, an Anti-Inflammatory JNK Inhibitor, and Clofibrate Have a Synergistic Effect in Inducing Cell Responses and in Affecting the Gene Expression Profile in CaCo-2 Colon Cancer Cells
Source: PPAR Res. 2012 Feb 29;2012:269751. doi: 10.1155/2012/269751 (PMC3349252; doi:10.1155/2012/269751)
Supplement: Supplementary file 2 [file 269751.f2.doc]

**Tab B** **GENE RELATIVE EXPRESSION DETECTED BY AFFYMETRIX AND QUANTITATIVE REAL-TIME PCR^a^**

|  | **Clofibrate** | | **AS601245** | | **Clofibrate+AS601245** | |
| --- | --- | --- | --- | --- | --- | --- |
| Gene | Affymetrix | qPCR (TaqMan) | Affymetrix | qPCR (TaqMan) | Affymetrix | qPCR (TaqMan) |
| **GANAB** | **3.8** | **2.8** | **2.3** | **2.8** | **3.6** | **3.7** |
| **FGFR2** | **unchanged** | **-1.5** | **-2.1** | **-1.7** | **unchanged** | **-1.6** |
| **IL6ST** | **2** | **2.1** | **2.8** | **2.4** | **6.5** | **6.4** |
| **CCNG2** | **unchanged** | **-2.7** | **-1.9** | **-2.7** | **-3.2** | **-2.4** |

**^a^**Gene relative expression detected by affymetrix and quantitative real-time reverse transcription PCR in Caco-2 cells treated with 5 µM clofibrate, 0.1 µM AS601245 and combined treatment.
