# Supplementary material for: AS601245, an Anti-Inflammatory JNK Inhibitor, and Clofibrate Have a Synergistic Effect in Inducing Cell Responses and in Affecting the Gene Expression Profile in CaCo-2 Colon Cancer Cells
Source: PPAR Res. 2012 Feb 29;2012:269751. doi: 10.1155/2012/269751 (PMC3349252; doi:10.1155/2012/269751)
Supplement: Supplementary file 3 [file 269751.f3.doc]

**Tab C** **GENES AFFECTED BY CLOFIBRATO, BY AS601245, AND BY THE COMBINED TREATMENT WITH CLOFIBRATE AND AS601245, ARRANGED WITH RESPECT TO THE RELATIVE BIOLOGICAL FUNCTIONS^b^**

| **CLOFIBRATO** | | |
| --- | --- | --- |
| **Biofunction** | **Genes p-value range** | **#Genes** |
| **Cancer** | 4.10E-06 - 4.20E-02 | 52 |
| **Cell Morphology** | 6.06E-06 - 4.20E-02 | 12 |
| **Cardiovascular System Development and Function** | 6.06E-06 - 3.38E-02 | 4 |
| **Skeletal and Muscular System Development and Function** | 6.06E-06 - 4.20E-02 | 9 |
| **RNA Post-Transcriptional Modification** | 1.45E-05 - 3.38E-02 | 10 |
| **Cellular Development** | 6.36E-05 - 4.20E-02 | 23 |
| **Gene Expression** | 1.96E-04 - 3.38E-02 | 28 |
| **Protein Synthesis** | 1.96E-04 - 2.54E-02 | 5 |
| **Hypersensitivity Response** | 1.07E-03 - 4.20E-02 | 3 |
| **Gastrointestinal Disease** | 1.47E-03 - 4.08E-02 | 16 |

| **AS601245** | | |
| --- | --- | --- |
| **Biofunction** | **Genes p-value range** | **#Genes** |
| **Cellular Compromise** | 8.79E-17 - 1.10E-02 | 207 |
| **Infectious Disease** | 8.79E-17 - 1.48E-06 | 231 |
| **RNA Post-Transcriptional Modification** | 1.23E-15 - 9.81E-03 | 95 |
| **Cell Death** | 2.71E-14 - 1.10E-02 | 466 |
| **Cell Cycle** | 5.01E-14 - 1.11E-02 | 262 |
| **Cellular Growth and Proliferation** | 2.55E-13 - 1.09E-02 | 495 |
| **Cancer** | 1.88E-12 - 1.11E-02 | 721 |
| **Reproductive System Disease** | 3.40E-10 - 1.11E-02 | 380 |
| **Genetic Disorder** | 3.50E-10 - 1.11E-02 | 643 |
| **Neurological Disease** | 5.74E-10 - 9.86E-03 | 378 |

| **CLOFIBRATO +AS601245** | | |
| --- | --- | --- |
| **Biofunction** | **Genes p-value range** | **#Genes** |
| **Cancer** | 4.85E-18 - 8.20E-03 | 270 |
| **Genetic Disorder** | 3.22E-12 - 4.99E-03 | 239 |
| **Cellular Growth and Proliferation** | 3.69E-09 - 7.17E-03 | 185 |
| **Cell Death** | 2.29E-08 - 8.20E-03 | 170 |
| **RNA Post-Transcriptional Modification** | 4.30E-08 - 7.17E-03 | 31 |
| **Cell Cycle** | 5.92E-08 - 8.12E-03 | 91 |
| **Neurological Disease** | 1.42E-07 - 7.17E-03 | 140 |
| **Reproductive System Disease** | 2.13E-07 - 8.20E-03 | 179 |
| **Gastrointestinal Disease** | 2.16E-06 - 6.92E-03 | 78 |
| **Gene Expression** | 4.66E-06 - 7.17E-03 | 120 |

**^b^**Genes affected by 5 µM clofibrato, by 0.1 µM AS601245, and by the combined treatment with clofibrato and AS601245, with respect to DMSO treated Caco-2 cells at 24h and arranged with respect to the relative biological functions
